# Comparative transcriptomics analysis on Senecavirus A-infected and non-infected cells

**DOI:** 10.3389/fvets.2024.1431879

**Published:** 2024-06-25

**Authors:** Yan Li, Huanhuan Chu, Yujia Jiang, Ziwei Li, Jie Wang, Fuxiao Liu

**Affiliations:** ^1^College of Veterinary Medicine, Qingdao Agricultural University, Qingdao, China; ^2^Qingdao Center for Animal Disease Control and Prevention, Qingdao, China; ^3^College of Veterinary Medicine, Northwest A&F University, Yangling, China; ^4^Qingdao Zhongren-OLand Bioengineering Co., Ltd., Qingdao, China

**Keywords:** Senecavirus A, RNA-seq, transcriptomics, differentially expressed gene, enrichment analysis, immunity, pathway

## Abstract

Senecavirus A (SVA) is an emerging virus that causes the vesicular disease in pigs, clinically indistinguishable from other high consequence vesicular diseases. This virus belongs to the genus *Senecavirus* in the family *Picornaviridae*. Its genome is a positive-sense, single-stranded RNA, approximately 7,300 nt in length, with a 3′ poly(A) tail but without 5′-end capped structure. SVA can efficiently propagate in different cells, including some non-pig-derived cell lines. A wild-type SVA was previously rescued from its cDNA clone using reverse genetics in our laboratory. In the present study, the BSR-T7/5 cell line was inoculated with the passage-5 SVA. At 12 h post-inoculation, SVA-infected and non-infected cells were independently collected for the analysis on comparative transcriptomics. The results totally showed 628 differentially expressed genes, including 565 upregulated and 63 downregulated ones, suggesting that SVA infection significantly stimulated the transcription initiation in cells. GO and KEGG enrichment analyses demonstrated that SVA exerted multiple effects on immunity-related pathways in cells. Furthermore, the RNA sequencing data were subjected to other in-depth analyses, such as the single-nucleotide polymorphism, transcription factors, and protein–protein interactions. The present study, along with our previous proteomics and metabolomics researches, provides a multi-omics insight into the interaction between SVA and its hosts.

## 1 Introduction

Senecavirus A (SVA), as an emerging virus, has been demonstrated to be a causative agent for vesicular disease in swine (1–5). SVA-infected pigs develop vesicular lesions mainly on the snout, dewclaw or (and) coronary band. Other signs include lameness, anorexia, lethargy, cutaneous hyperemia, and fever (6, 7). The SVA-induced signs are clinically indistinguishable from those of other vesicular diseases in pigs (8). The outbreak of SVA infection has been recently reported in several countries, including Canada, the United States, Brazil, China, Thailand, Vietnam and Chile. The transmission risk of SVA has attracted a great deal of attention from the pig industry around the world.

SVA is the only member of the genus *Senecavirus* in the family *Picornaviridae* (9). The virion is a typical icosahedral particle without envelope. It harbors a positive-sense, single-stranded RNA genome, approximately 7,300 nt in length, composed of 5′ untranslated region (UTR), long encoding region and 3’ UTR. Like those of other picornaviruses, the 5′ terminus of SVA genome does not contain a cap structure. In contrast, a short peptide (VPg) is covalently linked to the 5′ terminus, and plays an essential role in synthesizing the SVA genome. The 5’ UTR bears a type-IV internal ribosome entry site (10), structurally and functionally similar to those of pestiviruses (11), allowing for the initiation of polyprotein translation in a cap-independent manner. The 3’ UTR is approximately 70 nt in length, followed by a variable-length poly(A) tail (12). The encoding region of SVA polyprotein follows the standard “L–VP4–VP2–VP3–VP1–2A–2B–2C–3A–3B–3C–3D” layout. After SVA infection, the viral polyprotein will be translated in cytoplasm, and then gradually cleaved into 12 proteins, namely, L, VP4, VP2, VP3, VP1, 2A, 2B, 2C, 3A, 3B, 3C and 3D (13). The VP4, VP2, VP3 and VP1 as structural proteins participate in viral morphogenesis. The others are nonstructural proteins, albeit uninvolved in the package of virion, playing a crucial role in viral replication (14–16).

RNA sequencing (RNA-seq) is a technique that uses next-generation sequencing to reveal the presence and quantity of RNA molecules in a biological sample, providing a snapshot of gene expression in the sample, also known as transcriptome. A transcriptome is the full range of mRNA molecules expressed by an organism. The RNA-seq technique contributes to identifying a transcriptome in a given population, even in a single cell (17). Comparative transcriptomics facilitates the elucidation of differentiation between two groups (populations, species and so on) in their alternative gene spliced transcripts, post-transcriptional modifications, gene fusion, single-nucleotide polymorphism (SNP) and changes in gene expression over time (18). Large DNA viruses, such as human cytomegalovirus and African swine fever virus, contained very long genomes. Each of these viruses itself has a complicated transcriptome (19, 20) in virus-infected cells. In contrast, some small RNA viruses, such as picornavirus, only have a simple “transcriptome,” i.e., one single RNA genome. Therefore, it is meaningless to uncover a picornaviral “transcriptome” only based on a given picornavirus itself.

SVA can trigger a variety of metabolic and biochemical changes in cells through virus-specific or -nonspecific mechanisms (21–23). For example, SVA 2C protein can target mitochondria and cause release of cytochrome C into cytoplasm, which activates caspase-9 and -3 in a series of signaling cascades to induce the onset of apoptosis (24). In addition, SVA infection is able to affect the level of transcription in hosts. For example, SVA 2C protein can block the transcription of interferon-stimulated gene 56 and interferon-β to weaken the innate immunity in hosts (21). We have demonstrated that SVA infection can lead to significant changes in cellular proteome and metabolome, even at an early stage of infection (25, 26). Virus-caused differentiation of cellular proteome is closely related to the change in cellular transcriptome. Therefore, a comparative transcriptomics analysis was conducted here to uncover a profile of SVA-induced changes in cellular transcriptome at the early stage of infection.

## 2 Materials and methods

### 2.1 Cell line and virus

The BSR-T7/5 cell line, derived from the baby hamster kidney cell, was kindly provided by the China Animal Health and Epidemiology Center. This cell line was cultured at 37°C with 5% CO_2_ in Dulbecco’s modified Eagle’s medium (DMEM), supplemented with 4% fetal bovine serum (VivaCell, Shanghai, China), penicillin (100 U/mL), streptomycin (100 μg/mL) and amphotericin B (0.25 μg/mL). The wild-type SVA was rescued previously from a full-length cDNA clone (27), genetically derived from an SVA isolate, CH-LX-01-2016 (28).

### 2.2 Sample preparation

BSR-T7/5 cells were seeded into six T25 flasks for culture at 37°C. When the cells were 90% confluent, three flasks were randomly selected for incubation with the passage-5 SVA at an MOI (multiplicity of infection) of 2.5. The other flasks, as non-infected controls, were not treated. There were three SVA-infected samples (S1, S2 and S3) and three non-infected controls (C1, C2 and C3). Supernatants were separately removed from the six flasks at 12 h post-inoculation (hpi). Cell monolayers were gently washed with PBS three times, followed by the extraction of total RNAs using the TRIzol reagent (Thermo Fisher, Waltham, MA, United States), *as per* the manufacturer’s instructions. The concentration, quality and integrity of total RNAs were determined using the NanoDrop spectrophotometer (Thermo Fisher, Waltham, MA, United States). Three μg of RNA was used as input material to prepare RNA sample for each group.

### 2.3 RNA-seq analysis

The preparation of sequencing libraries was carried out as described previously with modifications (29). The mRNAs were purified from total RNAs using poly-T oligo-attached magnetic beads, further fragmented, and then used as templates to produce cDNAs. The first strand cDNA was synthesized using a system with random hexamer primers and the reverse transcriptase. The second strand cDNA was synthesized via the first strand with dNTP, buffer solution, DNA polymerase I and RNase H. Remaining overhangs were converted into blunt ends through exonuclease/polymerase activities. After adenylation of the 3′ ends of DNA fragments, Illumina paired-end adapter oligonucleotides were ligated to prepare for hybridization. The cDNA fragments of 400 to 500 bp were preferentially size-selected using the AMPure XP system (Beckman Coulter, Beverly, United States). DNA fragments with ligated adaptor molecules on both ends were selectively enriched using Illumina PCR Primer Cocktail in a 15-cycle PCR reaction. Products were purified using the AMPure XP system, and then quantified by the Agilent high sensitivity DNA assay on the Agilent 2,100 Bioanalyzer (Agilent Technologies, CA, United States). The sequencing libraries were subjected to sequencing on the NovaSeq 6,000 platform (Illumina, CA, United States) for obtaining image files.

### 2.4 Quality control and reads mapping

The image files were transformed by the software of sequencing platform. The original data was generated in a FASTQ format (raw data). Sequencing data contained a number of connectors and low-quality reads. The Cutadapt (v1.15) software was used to filter the sequencing data (30), subsequently obtaining high-quality sequences (clean data) for further analysis. Two reference genomes were those of the golden hamster (Genbank No.: PRJNA77669) and the SVA CH-LX-01-2016 (Genbank No.: KX751945). The filtered reads were separately mapped to both reference genomes using the HISAT2 (v2.0.5) program (31).

### 2.5 Analysis of differential expression

The analysis of differentially expressed genes (DEGs) was performed as described previously with modifications (32). The HTSeq (v0.9.1) was used to compare the Read Count values on each gene as the original gene expression (33). Gene expression was standardized through the FPKM (Fragments Per Kilobase of transcript per Million mapped reads). DEGs were determined by the DESeq (v1.30.0) with screening parameters as follows: the fold change (FC) > 2 (or <0.5) and the significant *p* value <0.05 (34). The bi-directional clustering analysis of all DEGs was performed by the Pheatmap (v1.0.8) package. The heatmap was drawn according to the expression level of the same gene in different groups and the expression patterns of different genes in the same group, with the Euclidean method for calculating the distance, and the complete linkage method for clustering.

### 2.6 Analyses of GO and KEGG enrichments

All genes were mapped to terms in the database of gene ontology (GO). Differentially enriched genes were calculated for each term. The topGO package was designed to perform the GO enrichment analysis on the DEGs. The *p* value was calculated by the hypergeometric distribution method. The *p* value <0.05 was determined as the standard of significant enrichment. The GO terms were found with significantly differentially enriched genes, all of which were further classified to determine the main biological functions. The ClusterProfiler (v3.4.4) software was used to carry out the enrichment analysis of DEGs on the KEGG (Kyoto Encyclopedia of Genes and Genomes) pathways. The *p* value <0.05 was determined as the standard of significant enrichment (35).

### 2.7 Other analyses on RNA-seq data

#### 2.7.1 Analysis of new transcripts

On the basis of the existing reference genome, the software StringTie (http://ccb.jhu.edu/software/stringtie/) was used to assemble the mapped reads (36). The assembling results were compared with the known transcripts for obtaining unannotated transcripts.

#### 2.7.2 Analysis of alternative splicing events

The rMATS (v3.2.5) software was used to uncover alternative splicing events (37). The main types of alternative splicing events included skipped exon (SE), retained intron (RI), alternative 5′ splice site (A5SS), alternative 3′ splice site (A3SS), and mutually exclusive exons (MXE).

#### 2.7.3 Analysis of SNP sites

The Varscan program was used to obtain SNP sites (38). The filtering criteria were: (i) SNP site base Q > 20, (ii) the number of reads covering the site >8, (iii) the number of reads supporting the mutation site >2, and (4) the *p* value of SNP locus <0.01.

#### 2.7.4 Prediction of transcription factors

Transcription factors and their own families were predicted via the comparison with the Animal Transcription Factor Database (AnimalTFDB) (39), a comprehensive database including classification and annotation of genome-wide transcription factors, transcription co-factors and chromatin remodeling factors in numerous animal genomes.

#### 2.7.5 Analysis of differential exon usage

The DEXSeq package was used to analyze the RNA-seq data for identifying the differential exon usage, as described previously (40).

#### 2.7.6 Interaction analysis in protein network

The STRING database (https://string-db.org/) was used to unveil putative protein–protein interactions (PPI) (41), contributing to clarifying the relationship among genes of interest.

### 2.8 Validation of gene expression by RT-qPCR

Four representative genes, namely, SVA genome, Nfkbia, Phlda2 and Txnip, were selected for validating the profile of gene expression. The GAPDH (glyceraldehyde-3-phosphate dehydrogenase) gene was used as an internal reference control. Gene-specific primers were listed in Supplementary file 18 for RT-qPCR validation. The RT-qPCR analysis was performed with three technical repeats, using the AceQ qPCR SYBR Green Master Mix (Vazyme, Nanjing, China) based on the LightCycler 480® Real-time PCR System (Roche, Rotkreuz, Switzerland), *as per* the manufacturer’s instructions. The RT-qPCR results were analyzed through the 2^−ΔΔCt^ method for relatively quantifying the four genes of interest (42). The GraphPad Prism (v8.0) was used for statistical analysis by two-tailed Student′s *t*-test with Welch′s correction. Data were shown as means ± standard deviations of three independent experiments.

## 3 Results

### 3.1 Sequencing for *de novo* transcriptome assembly

**Figure 1:**
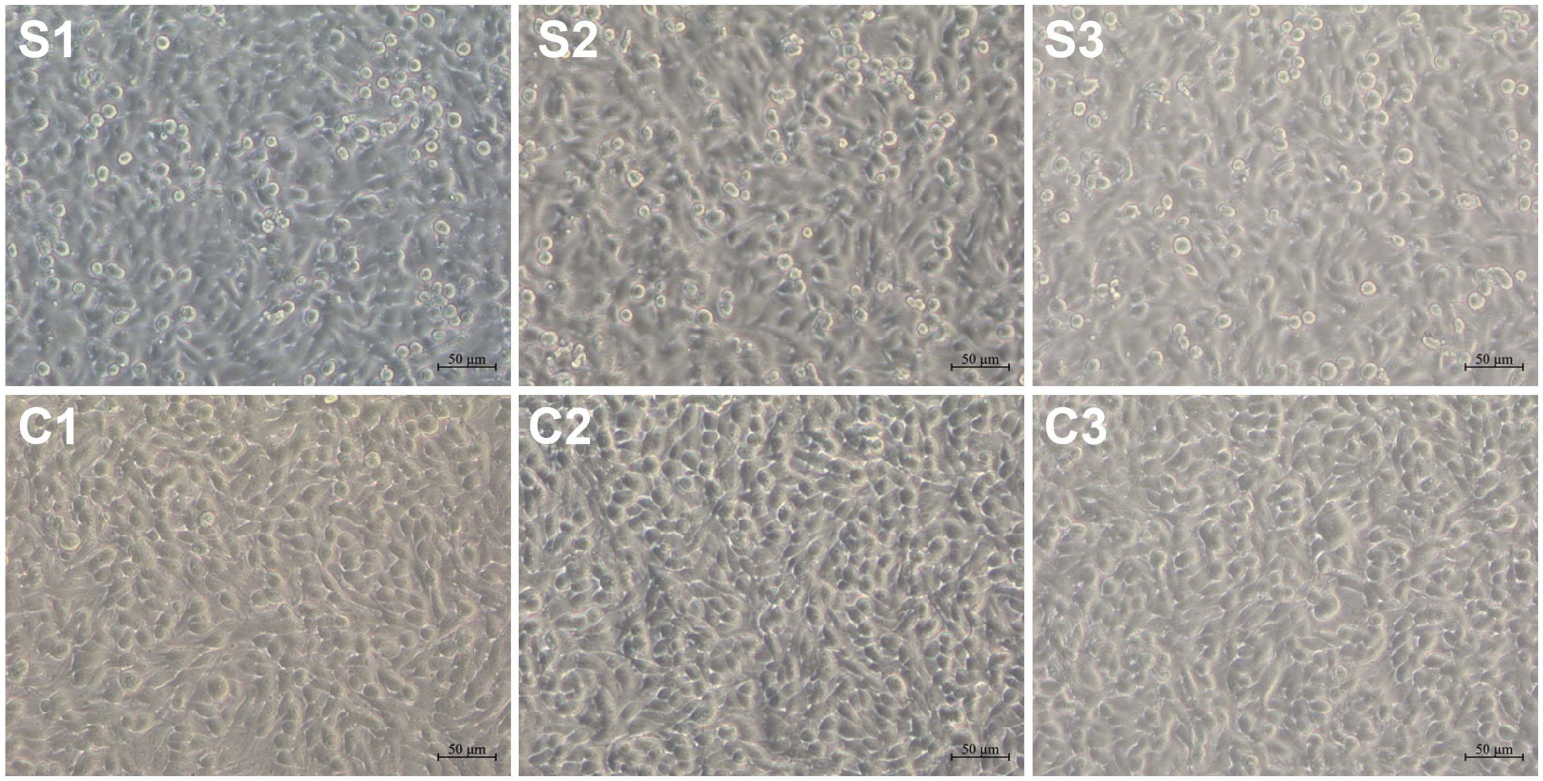
SVA-infected and non-infected cell monolayers at 12 hpi. S1, S2, and S3: SVA-infected groups; C1, C2 and C3: non-infected controls.

The BSR-T7/5 cell monolayers showed no obvious cytopathic effect (CPE) at 12 hpi (Figure 1). Cells were separately collected from SVA-infected and non-infected groups to extract total RNAs for the construction of high-quality cDNA libraries. The primary library-related data were listed in Supplementary file 1. The cDNA libraries were subjected to sequencing to obtain image files, subsequently transformed into raw data for statistical classification, as shown in Table 1. Because the raw data contained a number of connectors and low-quality reads, the Cutadapt (v1.15) software was used to filter the raw data for obtaining high-quality clean sequences, as listed in Supplementary file 2. There were three key parameters, namely, base mass, base content, and average mass of reads. Their distributions were independently shown in Supplementary 3, 4 and 5.

**Table 1:** Statistic data of RNA-seq for all six groups.

Sample name	Reads No.	Bases (bp) No.	Q30 (bp)	*N* (%)	Q20 (%)	Q30 (%)
S1	45,327,558	6,844,461,258	6,647,043,638	0.012777	99.00	97.12
S2	53,263,406	8,042,774,306	7,823,495,397	0.012671	99.06	97.27
S3	39,688,814	5,993,010,914	5,820,866,532	0.012943	99.02	97.13
C1	42,486,048	6,415,393,248	6,235,364,335	0.012489	99.01	97.19
C2	53,252,482	8,041,124,782	7,800,962,620	0.012589	98.95	97.01
C3	57,220,586	8,640,308,486	8,385,089,387	0.013141	98.96	97.05

Reads No.: the total number of reads; Bases (bp) No.: the total number of bases; Q30 (bp): the total number of bases with base recognition accuracy more than 99.9%; *N* (%): the percentage of ambiguous bases; Q20 (%) or Q30 (%): the percentage of Q20 or Q30 bases with base recognition accuracy more than 99%.

### 3.2 Transcriptomic mapping

The filtered reads were mapped to both reference genomes, those of the golden hamster and the SVA CH-LX-01-2016, using the HISAT2 (v2.0.5) program. The results of RNA-seq mapping were listed in Table 2. The global profile of reads was subjected to the further statistical analysis on the distribution of reads mapped to both genomes, in which genetic elements included coding sequence, intron, intergenic spacer, and UTR. The mapping results were listed in Table 3, and shown in Supplementary file 6. Supplementary file 7 exhibited the coverage distributions of reads mapped to genes. To sum up, a high-quality dataset of RNA-Seq was harvested here, meeting a standard for the further bioinformatics analysis.

**Table 2:** Statistical data of RNA-seq mapping.

Sample name	Clean reads	Total mapped (Rate)	Multiple mapped (Rate)	Uniquely mapped (Rate)
S1	44,738,448	40,444,421 (90.40%)	770,019 (1.90%)	39,674,402 (98.10%)
S2	52,647,920	47,570,136 (90.36%)	904,976 (1.90%)	46,665,160 (98.10%)
S3	39,219,782	35,252,668 (89.88%)	643,266 (1.82%)	34,609,402 (98.18%)
C1	41,942,238	36,798,807 (87.74%)	902,042 (2.45%)	35,896,765 (97.55%)
C2	52,501,164	45,940,744 (87.50%)	1,092,548 (2.38%)	44,848,196 (97.62%)
C3	56,437,286	49,541,887 (87.78%)	1,183,403 (2.39%)	48,358,484 (97.61%)

Clean reads: the number of sequences used for mapping; Total mapped (Rate): the number of sequences successfully mapped (Total mapped/Clean reads); Multiple Mapped (Rate): the number of sequences mapped to multiple regions (Multiple mapped/Total mapped); Uniquely Mapped (Rate): the number of sequences mapped to a single region (Uniquely mapped/Total mapped).

**Table 3:** Distribution of read-mapped regions.

Sample name	Map events	Mapped to gene (Rate)	Mapped to intergene (Rate)	Mapped to exon (Rate)
S1	39,674,402	36,731,507 (92.58%)	2,942,895 (7.42%)	34,599,182 (94.19%)
S2	46,665,160	42,794,501 (91.71%)	3,870,659 (8.29%)	39,814,657 (93.04%)
S3	34,609,402	31,712,144 (91.63%)	2,897,258 (8.37%)	29,532,363 (93.13%)
C1	35,896,765	32,161,772 (89.60%)	3,734,993 (10.40%)	29,444,837 (91.55%)
C2	44,848,196	39,799,780 (88.74%)	5,048,416 (11.26%)	35,909,925 (90.23%)
C3	48,358,484	43,583,502 (90.13%)	4,774,982 (9.87%)	40,133,590 (92.08%)

Map events: the number of mapping events; Mapped to gene (Rate): the number of reads mapped to genetic regions (Mapped to gene/Map events); Mapped to InterGene: the number of reads mapped to intergenic spacers (Mapped to intergene/Map events); Mapped to exon (Rate): the number of reads mapped to exons (Mapped to exon/Mapped to gene).

### 3.3 Profile of gene expression

**Figure 2:**
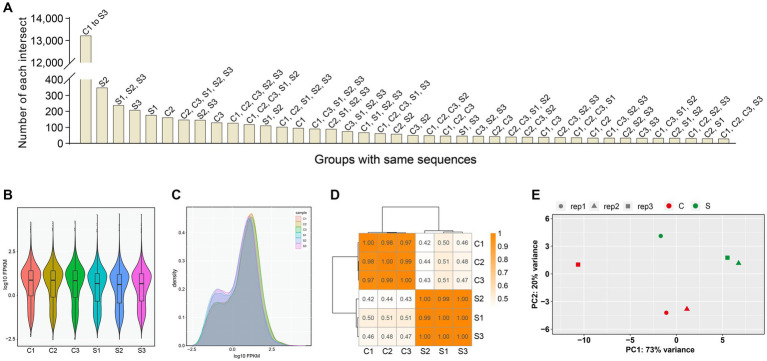
Profile of gene expression in all groups. The numbers of genes that are either co-identified in different groups or recognized in a single group **(A)**. Violin plot of FPKM distributions in all groups **(B)**. Distributions of FPKM densities in all groups **(C)**. The correlation analysis of gene expression via the calculation of Pearson’s correlation coefficients **(D)**. Principal component analysis on all groups **(E)**. PC1: principal component 1. PC2: principal component 2.

A total of 20,374 genes were identified in all six groups (Supplementary file 8), but these genes contained more than 3,000 components with FPKM value = 0. FPKM was a simple method for normalizing the read count data, based on gene length and the total number of mapped reads. The FPKM-normalized expression level was divided into different intervals (Supplementary file 9) for the six groups, as shown in Supplementary file 10. The number of genes, either co-identified in different groups or recognized in a single group, was shown in Figure 2A. The density distribution of FPKM values was displayed in Figures 2B,C, as two different forms. To validate whether the sequencing depth of RNA-seq was sufficient for the analysis of gene expression, the saturation analysis was performed for all six groups, as shown in Supplementary file 11. The correlation analysis of gene expression, based on calculation of Pearson’s correlation coefficients, was carried out among the six groups (Figure 2D). The closer to 1.0 the correlation coefficient was, the higher the similarity of expression pattern was among the six groups. Principal component analysis made it possible to project a high-dimensional dataset onto two or three dimensions, as shown in Figure 2E. The closer the distance was, the higher the similarity was among groups.

### 3.4 Analysis of differential expression

**Figure 3:**
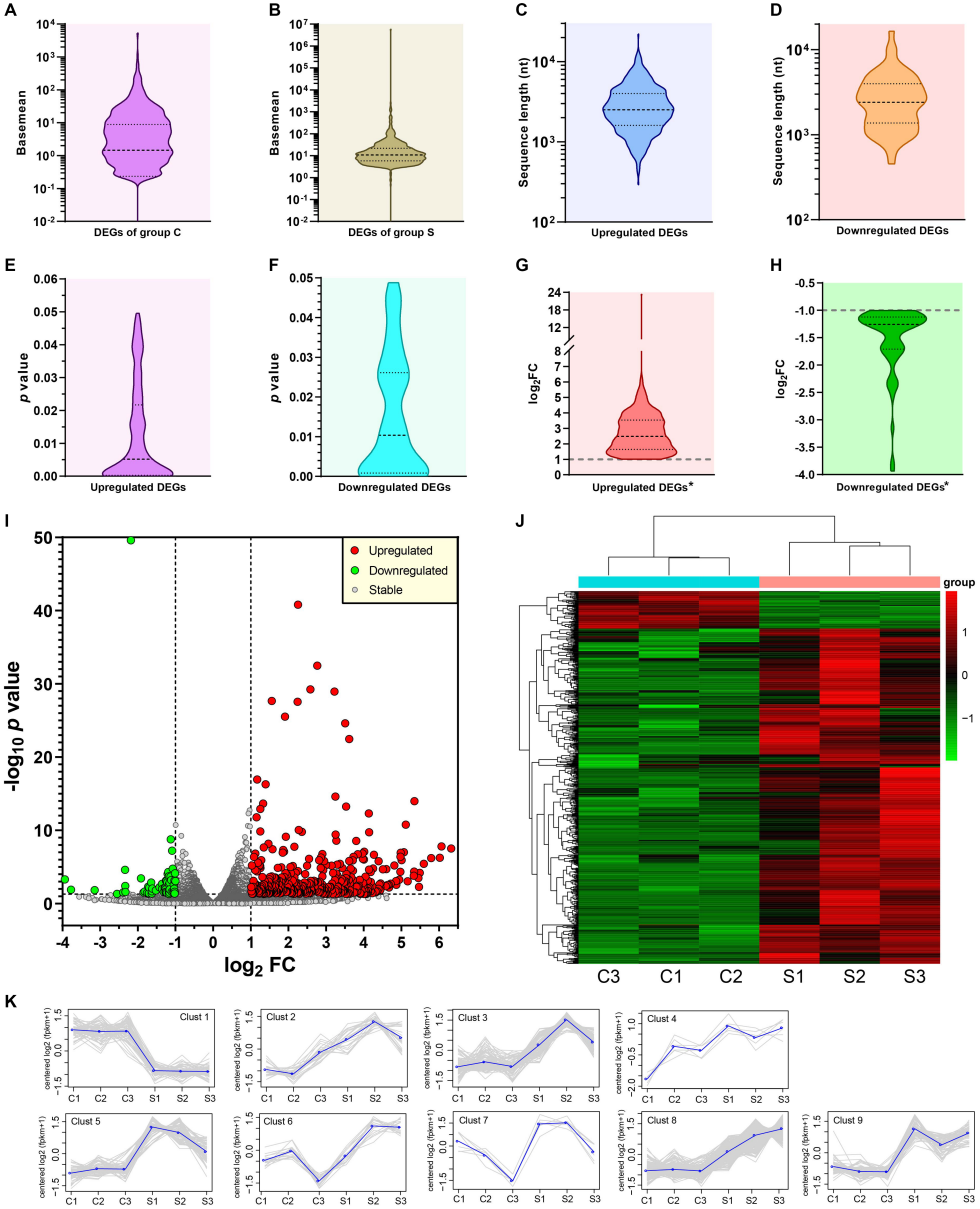
Profile and analysis of DEGs. Distribution of basemean values in group C **(A)** and S **(B)**. Distributions of sequence lengths of upregulated **(C)** and downregulated **(D)** DEGs. Distributions of *p* values of upregulated **(E)** and downregulated **(F)** DEGs. Distributions of log_2_FC values of upregulated **(G)** and downregulated **(H)** DEGs. *Excluding the SVA-related data. Volcano plot of *p* value versus FC for all identified genes but excluding outliers **(I)**. The threshold values are set as |log_2_FC| > 1 and *p* value <0.05. Heatmap based on bi-directional clustering analysis of all DEGs **(J)**. Clustering analysis on expression patterns of DEGs **(K)**. Grey lines indicate expression patterns. Each blue line represents the average value in each cluster.

DEGs were determined by the DESeq (v1.30.0) with screening parameters, |log_2_FC| > 1 and the significant *p* value <0.05. A total of 628 DEGs, including 565 upregulated (Supplementary file 12) and 63 downregulated (Supplementary file 13) components, were identified here. The basemean was described as the “mean of normalized counts of all samples.” The basemean values of DEGs, corresponding to the group C and S, were exhibited in Figures 3A,B, respectively. The distributions of sequence length, *p* value and log_2_FC were exhibited in Figures 3C–H. A single asterisk in Figures 3G,H indicated the exclusion of positive or negative infinity (“Inf” in Supplementary files 12, 13), respectively. The distribution and degree of differential expression were graphically shown in a volcano plot and a heatmap, respectively.

The volcano plot (Figure 3I), drawn by the GraphPad Prism software, revealed the *p* value versus the FC for all identified genes. The threshold values were set as |log_2_FC| > 1 and *p* value <0.05. The upregulated, downregulated and stably-expressed genes were indicated by red, green and grey circles, respectively. The R language Pheatmap (1.0.8) software package was used for the bi-directional clustering analysis to draw the heatmap (Figure 3J), which provided a visual depiction for hierarchical clustering of all 628 DEGs from the six groups. The red and green labels represented the upregulated and downregulated DEGs, respectively. The intensity of color reflected the degree of differentiation in gene expression. All 628 DEGs, based on their differences in expression patterns, were classified into nine different clusters (Figure 3K), in which grey lines indicated expression patterns, and each blue line represented the average value in each cluster.

### 3.5 GO enrichment analysis

**Figure 4:**
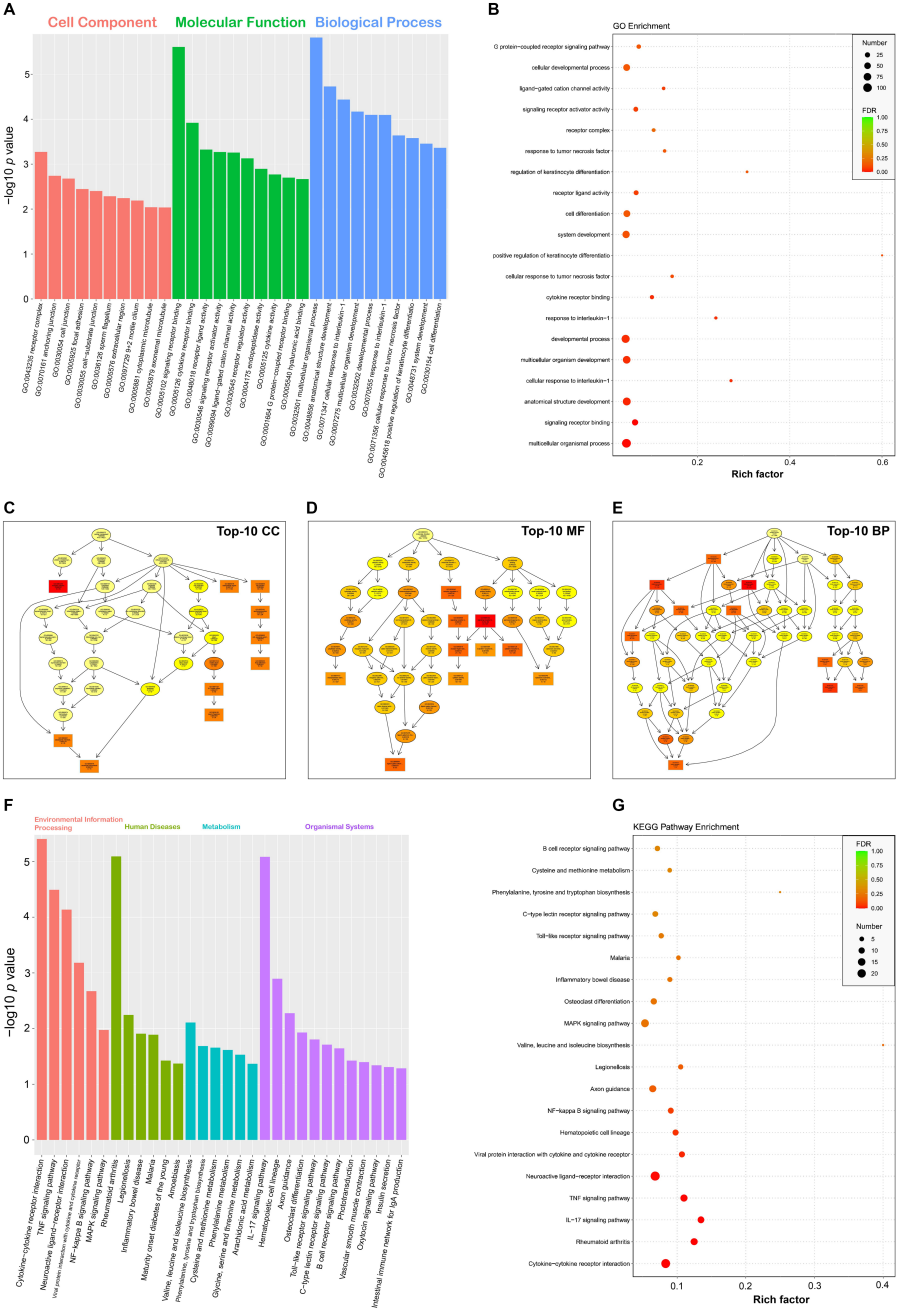
GO and KEGG enrichment analyses of DEGs. Top-10 statistically significant GO terms of three categories **(A)**. Bubble plot of top-20 statistically significant GO terms **(B)**. Directed acyclic graphs of top-10 statistically significant GO categories **(C–E)**. GO terms with the top-10 lowest FDRs are framed with rectangles, and the others are indicated by ellipses. The more statistically significant a GO term is, the darker its color is. Top-30 statistically significant KEGG pathways, classified into four categories **(F)**. Bubble plot of top-20 statistically significant KEGG pathways **(G)**.

The topGO package was used for performing the GO enrichment analysis on DEGs. The GO terms were found with significantly differentially enriched genes. The numbers of category BP, CC and MF were 3,093, 357 and 644, respectively. The complete GO data were listed in Supplementary file 14 in detail. Figure 4A displayed the top-10 statistically significant GO terms for each GO category. The false discovery rate (FDR), ranging from 0 to 1, was associated with the degree of GO enrichment. The lower the FDR was, the more significant the enrichment degree was. The GO terms with the top-20 lowest FDRs were shown in a bubble plot (Figure 4B). Each GO category was organized further as a directed acyclic graph (Figures 4C–E), in which parental terms described more general functional categories than their next-generation terms. GO terms with the top-10 lowest FDRs were framed with rectangles, and the others were indicated by ellipses. The more statistically significant a GO term was, the darker its color was.

### 3.6 KEGG enrichment analysis

The analysis of KEGG pathway enrichment was performed to uncover DEG-related pathways. The result showed that DEGs were enriched in a total of 275 KEGG pathways (Supplementary file 15). Figure 4F displayed the top-30 statistically significant KEGG pathways (*p* value <0.05), classified into four categories, namely, environmental information processing, human diseases, metabolism, and organismal systems. According to the result of KEGG enrichment, the degree of enrichment was evaluated through the rich factor, FDR, and the number of DEGs enriched in a given pathway. The higher the rich factor was, the more significant the enrichment degree was. The lower the FDR was, the more significant the enrichment degree was. The KEGG pathways with the top-20 lowest FDRs were shown in a bubble plot (Figure 4G).

### 3.7 Other analyses on RNA-seq data

**Figure 5:**
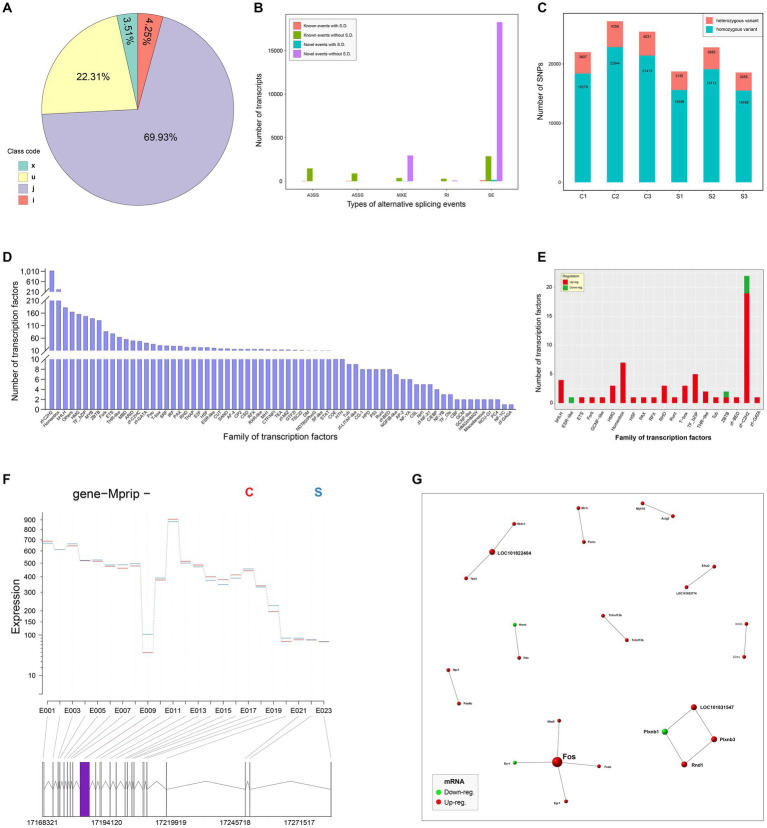
In-depth analysis of RNA-seq data. Pie chart of new transcripts **(A)**. All new transcripts are classified into four categories, x, u, j and i. Analysis of alternative splicing events **(B)**. X and Y axes indicate the types of alternative splicing events, and the number of new transcripts, respectively. S.D.: significant differentiation. The numbers of SNP sites in all groups **(C)**. Profile of transcription factor families **(D)**. The Y axis indicates the number of transcription factors in each family. Transcription factor families with significantly differential transcription factors **(E)**. A representative gene with differential exon usage **(F)**. The differential exon usage is marked with a purple rectangle. PPI network with 26 nodes and 17 edges **(G)**. The score of PPI is set to be more than 0.95. Red and green nodes indicate upregulated and downregulated genes, respectively.

The StringTie was used to assemble the mapped reads. The assembling results were compared with the known transcripts to obtain unannotated transcripts. The transcripts from class codes j, i, u, and x, regarded as new transcripts, were functionally annotated and listed in Supplementary file 16. The proportion of each class code was shown in Figure 5A. Five types of alternative splicing events were analyzed by the rMATS (v3.2.5) software. The SE and RI exhibited the most and the least alternative splicing events, respectively (Figure 5B). The SNP sites were analyzed by the Varscan program. The numbers of heterozygous and homozygous variants were shown in Figure 5C. Transcription factors and their own families were predicted through the comparison with those in the AnimalTFDB. Figure 5D showed the number of transcription factors in each family. Out of the identified families, 20 were demonstrated to contain upregulated, downregulated or both components (Figure 5E). The DEXSeq package was used to analyze the RNA-seq data for identifying the differential exon usage, as shown in Supplementary file 17. Figure 5F revealed a representative gene with differential exon usage. DEGs were comprehensively analyzed in the STRING database to unveil potential PPIs (Score > 0.95) for constructing a PPI network, which including 26 nodes and 17 edges (Figure 5G). Red and green nodes indicated upregulated and downregulated genes, respectively.

### 3.8 Validation of gene expression by RT-qPCR

**Figure 6:**
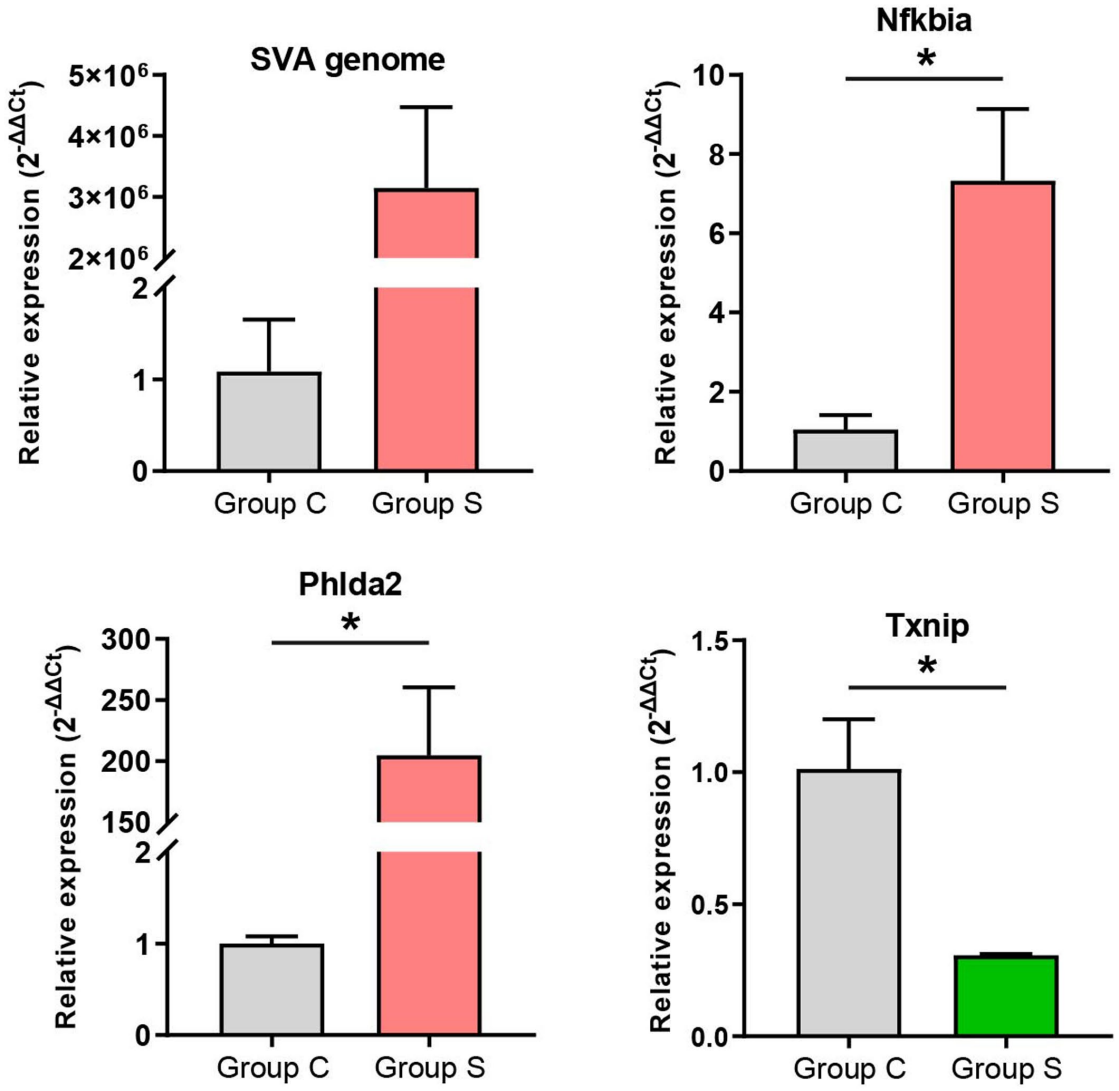
RT-qPCR validation of gene expression. The 2^-ΔΔCt^ method is used for relative quantification. The GAPDH gene is an internal control for normalization. Data are shown as means ± standard deviations of three independent experiments. Statistical significance is determined by two-tailed Student′s *t*-test with Welch′s correction. *p<0.05.

Three upregulated and one downregulated DEGs were selected for validating the profile of gene expression through RT-qPCR. The three upregulated genes included SVA genome, Nfkbia and Phlda2 (Supplementary file 12); the downregulated gene was Txnip (Supplementary file 13). The RT-qPCR detection demonstrated that the expression trend of DEGs was consistent with the result of RNA-seq analysis (Figure 6). Due to the group C without SVA inoculation, the expression differentiation of SVA genome was extremely significant between both groups (Figure 6, Left upper). There was no need for the statistical analysis on it.

## 4 Discussion

The *Picornaviridae* is a well-characterized family within the plus-strand RNA viruses. SVA is a typical picornavirus. Its genome is only a positive-sense, single-stranded mRNA, harboring a 3′ poly(A) tail but no 5′ capped structure. In other words, an SVA virion has a single mRNA, which however is not the viral transcript. Picornaviruses, albeit structurally simple, possibly have significant effects on physiological functions in their hosts. After entrance of virion into a cell, a picornaviral genome will be released into cytosol. This genome either relies on the host translation machinery to initiate the translation of polyprotein, or serves as a template to synthesize an antigenome, which will be used as a template for synthesizing another genome. The nascent genome can be used as a template for the next round of translation or replication, and alternatively is packaged into a virion (43). Therefore, although SVA as such has no concept of viral transcriptome, its infection may exert a significant impact on the cellular transcriptome. This prompted us to conduct the present study for uncovering the transcriptomic change in SVA-infected cells.

A replication-competent SVA was previously rescued from its cDNA clone in our laboratory (27). The passage-5 SVA was used here as a model virus. Despite SVA inoculation with MOI of 2.5, three cell monolayers showed no visible CPE at 12 hpi (Figure 1). Because we demonstrated previously that SVA infection led to significant cellular changes both in proteomic and in metabolomic profiles at 12 hpi, it could be postulated that the cellular transcriptome would be also affected at 12 hpi to some extent. The RNA-seq recognized totally 20,374 genes in the six groups, but containing more than 3,000 genes with FPKM value = 0. The correlation of gene expression is an important indicator to demonstrate the reliability of experiment, and the reasonability of samples. A certain correlation coefficient, if between 0.8 and 1.0, would indicate the extremely strong correlation between two groups. The current correlation analysis displayed the extremely strong intra-group correlation, but the weak inter-group correlation (Figure 2D), implying the RNA-seq data that were reliable.

RNA-seq data were subjected to the further analysis on the differentiation expression. The result totally recognized 565 upregulated and 63 downregulated DEGs here. Out of these DEGs, five representative genes (three upregulated and two downregulated genes) were selected out for RT-qPCR analysis to validate preliminarily the profile of DEGs. Except the downregulated DEG, Tcta gene (data not shown), the other four showed their expression trends consistent with the result of RNA-seq analysis (Figure 6). In our previous study on comparative proteomics between SVA-infected and non-infected cells, we identified totally 305 upregulated and 56 downregulated DEPs (differentially expressed proteins) (25). Regardless of the present or the previous study, the number of upregulated components was much higher than that of downregulated ones. Such a result was consistent with our postulation that DEGs shared a similar regulation trend with DEPs between SVA-infected and non-infected groups. Out of the identified DEGs in group S, the SVA genome was most statistically significant in the expression level (Supplementary file 12). Due to the group C without SVA inoculation, both GO and KEGG enrichment analyses excluded the data of SVA genome, and others with positive or negative infinity (Supplementary files 12, 13). The KEGG enrichment analysis showed that several DEGs were significantly enriched in many immunity-related pathways, such as TNF signaling pathway, IL-17 signaling pathway, Toll-like receptor signaling pathway, and B cell receptor signaling pathway (Figure 4F). The GO enrichment analysis also revealed a few statistically significant terms associated with immune responses, e.g., the response to interleukin-1 and the cellular response to tumor necrosis factor (Figure 4A). Like the conclusion drawn in a previous report (44), the current results also suggest that SVA infection may be able to induce significantly immune responses, especially the innate immune response, in hosts at an early stage of infection.

Further, RNA-seq data were subjected to the in-depth analyses, concerning SNPs, transcription factors, PPI and so on. The analysis of SNP events indicated that there was no significant differentiation in the number of SNP events between group C and S (Figure 5C), implying that SVA infection had no ability of inducing the occurrence of SNP events in the host genome. It is worth noting that out of the 20 statistically significant families of transcription factors, most of them only contain upregulated components (Figure 5E). This result implies that SVA infection can notably stimulate multiple transcriptional pathways, resulting in upregulated DEGs far more than downregulated DEGs. The STRING database was used here to unravel putative PPIs in SVA-infected cells. The result revealed no formation of complicated interaction network among 26 putative DEPs (Figure 5G). Although the 26 DEPs include no SVA-related protein, the possibility that SVA-related proteins interact with cellular proteins cannot be ruled out, because the information of SVA proteins has not been deposited in the STRING database.

SVA emerged in many countries and regions over the past 20 years. It has been still considered as an emerging virus. Natural selection has been a primary evolutionary force affecting SVA codon usage bias (45). Multi-omics analysis provides an integrated approach to facilitate in-depth studies on the virology, especially on the interaction of viruses with their hosts. Based on our previous researches on proteomics and metabolomics, it was demonstrated that SVA infection could lead to significant changes in cellular intrinsic components even at an early stage of infection (25, 26). In order to comparatively analyze transcritpomic profiles between SVA-infected and non-infected cells, we conducted the present study. To sum up, the current results revealed that most of the DEGs were upregulated genes, indicating that SVA infection positively stimulated the transcription initiation in cells. GO and KEGG enrichment analyses demonstrated that SVA could markedly affect immunity-related pathways in cells, whereas the mechanism remained to be elucidated.

## Data availability statement

The data presented in the study are deposited in the NCBI repository, accession number PRJNA1100277.

## Author contributions

YL: Writing – original draft, Methodology, Formal analysis. HC: Writing – original draft, Methodology, Investigation, Data curation. YJ: Writing – review & editing, Formal analysis. ZL: Writing – review & editing, Investigation. JW: Writing – review & editing, Software. FL: Writing – review & editing, Writing – original draft, Supervision, Conceptualization.
