# Supplementary material for: Comparative transcriptomics analysis on Senecavirus A-infected and non-infected cells
Source: Front Vet Sci. 2024 Jun 25;11:1431879. doi: 10.3389/fvets.2024.1431879 (PMC11231404; doi:10.3389/fvets.2024.1431879)
Supplement: Supplementary file 1 [file Table_1.DOC]

**Supplementary 1**

**cDNA libraries of six groups**

| **Sample** | **Lib. Name** | **Lib. Insert Size** | **Sequencing Platform** | **Sequencing Mode** | **Exp. Name** |
| --- | --- | --- | --- | --- | --- |
| S1 | LRA186870 | 380bp | NovaSeq | Paired-end, 2×150bp | S1 |
| S2 | LRA186871 | 380bp | NovaSeq | Paired-end, 2×150bp | S2 |
| S3 | LRA186872 | 380bp | NovaSeq | Paired-end, 2×150bp | S3 |
| C1 | LRA186873 | 380bp | NovaSeq | Paired-end, 2×150bp | C1 |
| C2 | LRA186874 | 380bp | NovaSeq | Paired-end, 2×150bp | C2 |
| C3 | LRA186875 | 380bp | NovaSeq | Paired-end, 2×150bp | C3 |
