# Supplementary material for: Comparative transcriptomics analysis on Senecavirus A-infected and non-infected cells
Source: Front Vet Sci. 2024 Jun 25;11:1431879. doi: 10.3389/fvets.2024.1431879 (PMC11231404; doi:10.3389/fvets.2024.1431879)
Supplement: Supplementary file 2 [file Table_2.DOC]

**Supplementary 2** Date of clean sequences

| Sample | Clean Reads No. | Clean Data (bp) | Clean Reads % | Clean Data % |
| --- | --- | --- | --- | --- |
| S1 | 44738448 | 6743097798 | 98.70 | 98.52 |
| S2 | 52647920 | 7936282772 | 98.84 | 98.68 |
| S3 | 39219782 | 5912815859 | 98.82 | 98.66 |
| C1 | 41942238 | 6321565517 | 98.72 | 98.54 |
| C2 | 52501164 | 7912646345 | 98.59 | 98.40 |
| C3 | 56437286 | 8503948473 | 98.63 | 98.42 |
