# Supplementary material for: Comparative transcriptomics analysis on Senecavirus A-infected and non-infected cells
Source: Front Vet Sci. 2024 Jun 25;11:1431879. doi: 10.3389/fvets.2024.1431879 (PMC11231404; doi:10.3389/fvets.2024.1431879)
Supplement: Supplementary file 10 [file Table_10.doc]

**Supplementary Table 10** Primers of RT-qPCR analysis

| Primers | Sequences (5′ to 3′) |
| --- | --- |
| SVA-F | GGCCTGCATTTTCTCTCTCG |
| SVA-R | TCCATGGGTCCTTGCATCTT |
| Nfkbia-F | AGGATGAGGAGAGTTACGA |
| Nfkbia-R | CCTCCAAACACACAGTCA |
| Phlda2-F | CTTCCATTCCATCCTCAA |
| Phlda2-R | AAGTCGATCTCCTTGTAG |
| Txnip-F | AACAGACCTTGGACTACC |
| Txnip-R | ATCACCATCTCGTTCTCA |
| GAPDH-F | TCCACACCTTCTACTGAT |
| GAPDH-R | TTGCTGACAATCTTGAGG |
