# Supplementary figures and images for: Comparative transcriptomics analysis on Senecavirus A-infected and non-infected cells (part 1 of 2)
Source: Front Vet Sci. 2024 Jun 25;11:1431879. doi: 10.3389/fvets.2024.1431879 (PMC11231404; doi:10.3389/fvets.2024.1431879)

gene-ATP6+gene-COX3+gene-ATP8+

S

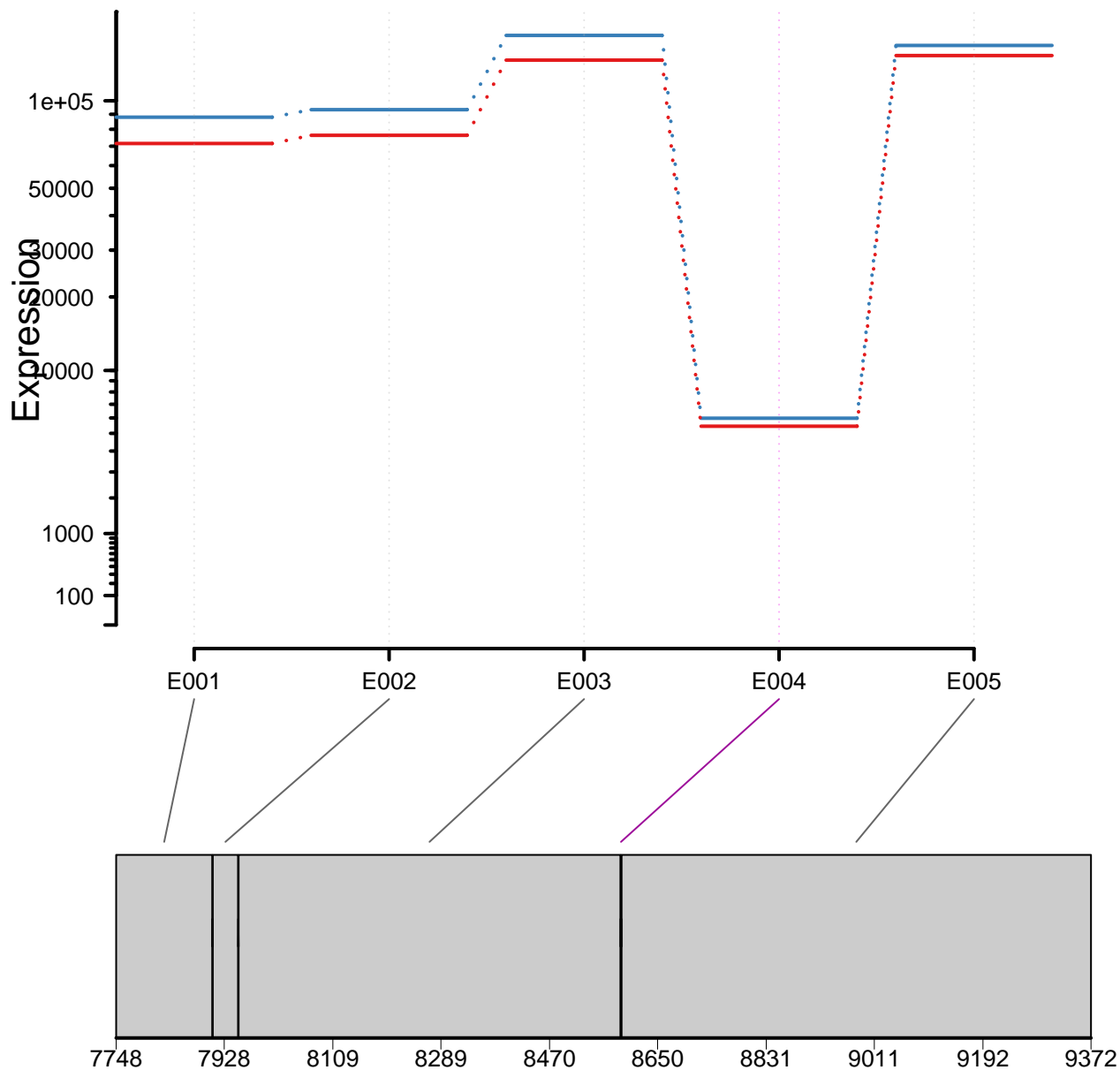

Supplement: Supplementary file 11 [file Data_Sheet_1.ZIP › Supplementary 17/gene-ATP6+gene-COX3+gene-ATP8.pdf]

gene-Ankrd10 -

C

S

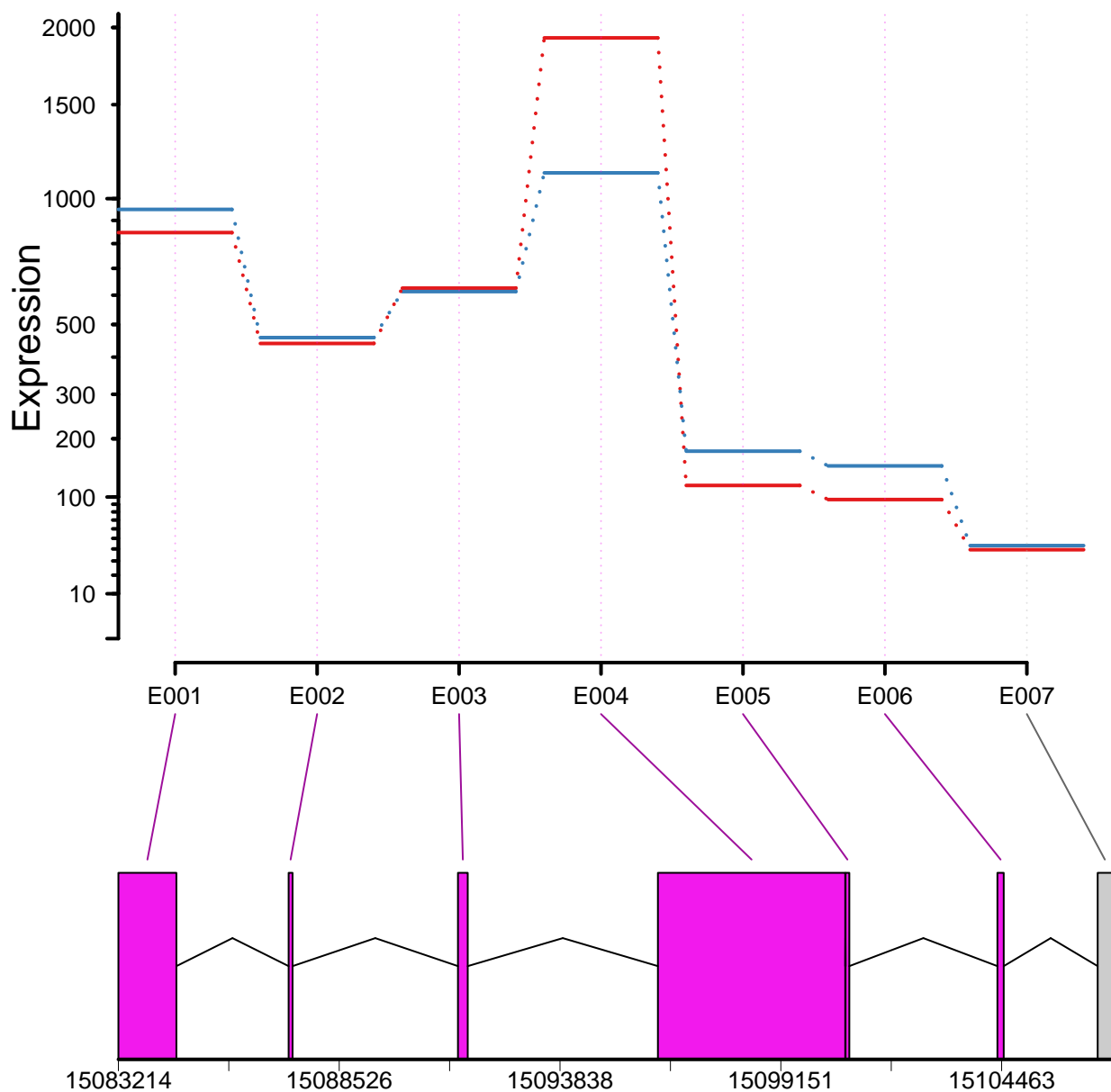

Supplement: Supplementary file 11 [file Data_Sheet_1.ZIP › Supplementary 17/gene-Ankrd10.pdf]

gene-Ankrd11 -

C

S

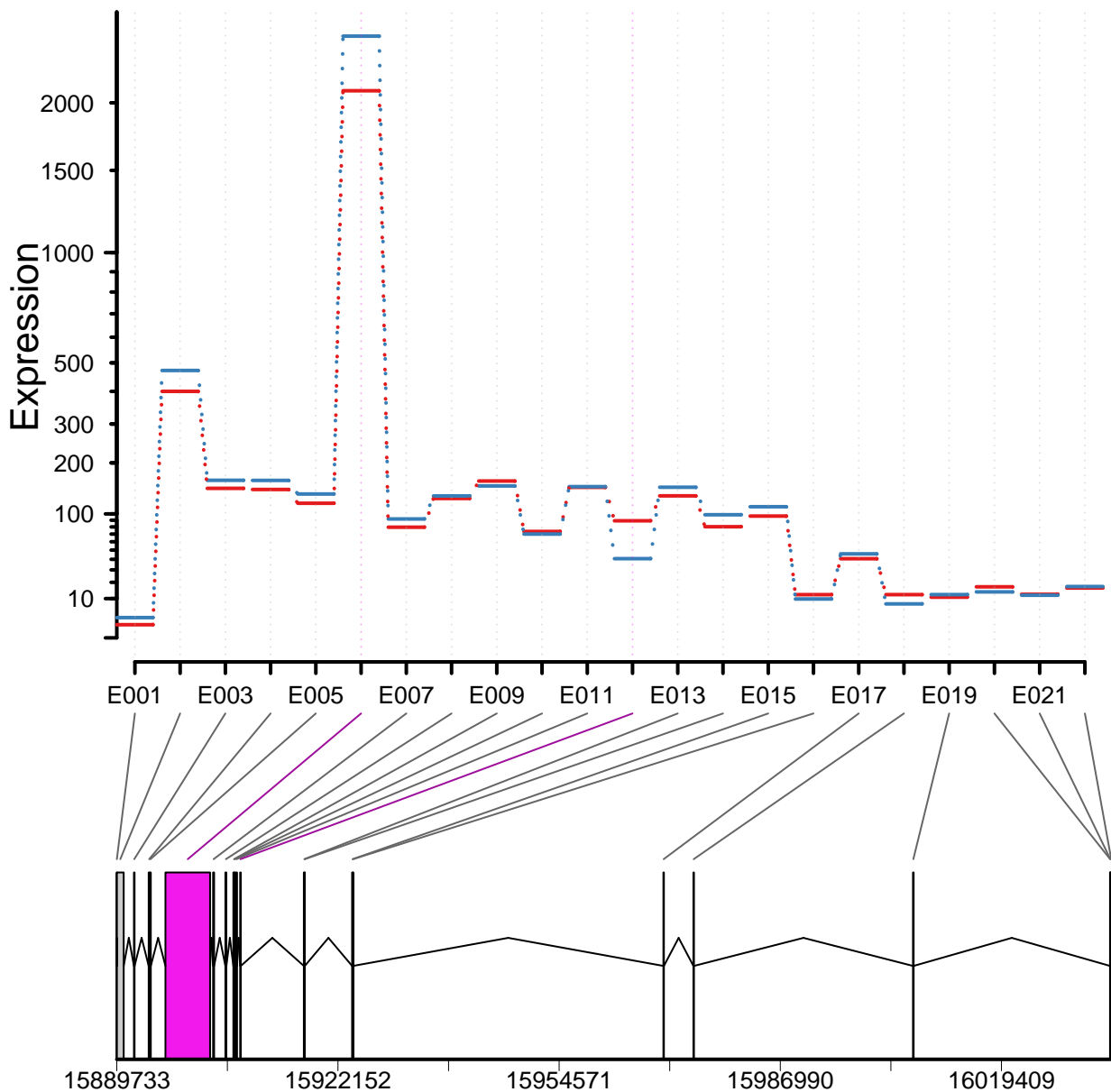

Supplement: Supplementary file 11 [file Data_Sheet_1.ZIP › Supplementary 17/gene-Ankrd11.pdf]

gene-Appl1 -

C

S

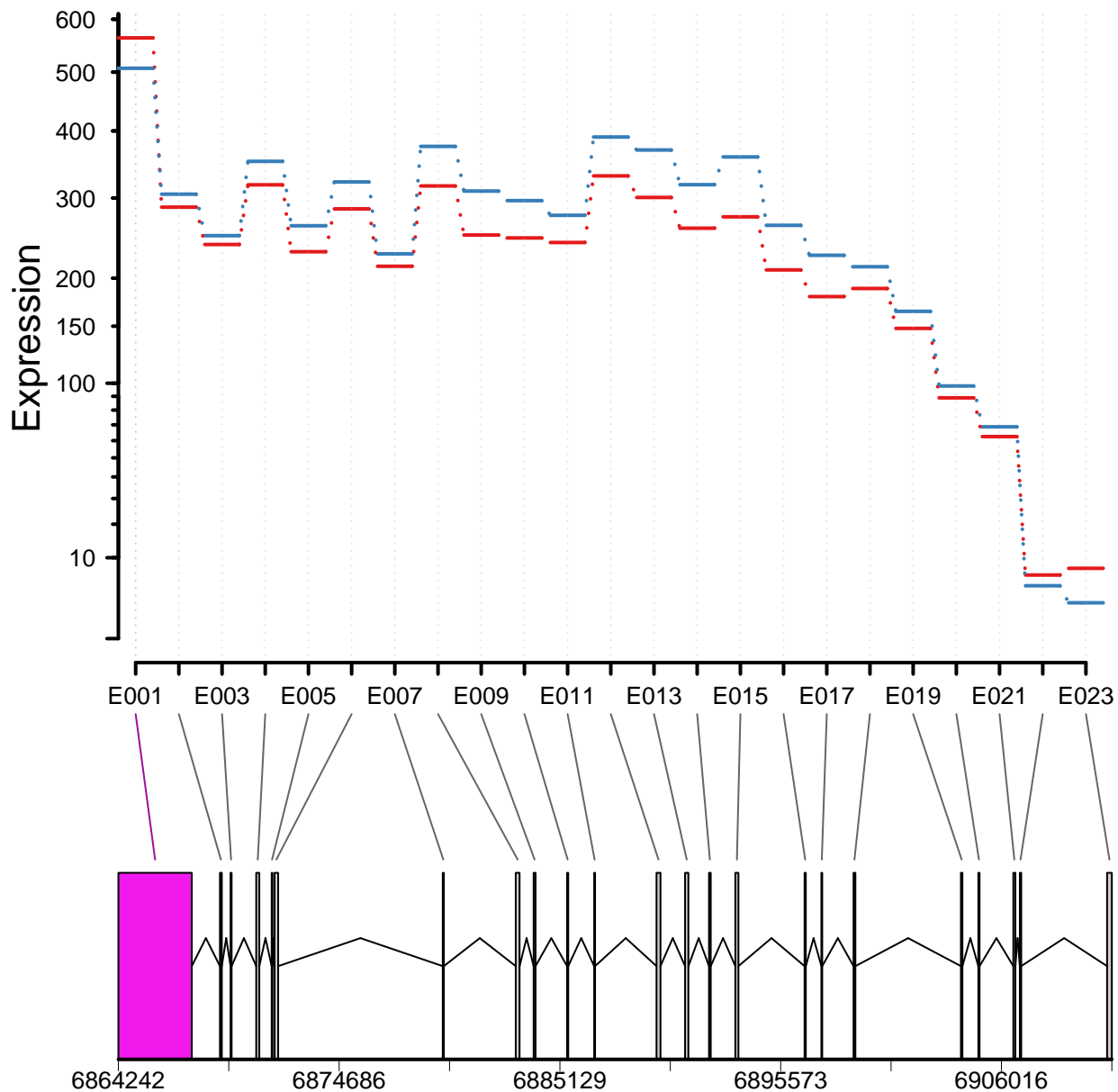

Supplement: Supplementary file 11 [file Data_Sheet_1.ZIP › Supplementary 17/gene-Appl1.pdf]

gene-Arfgef2 +

C

S

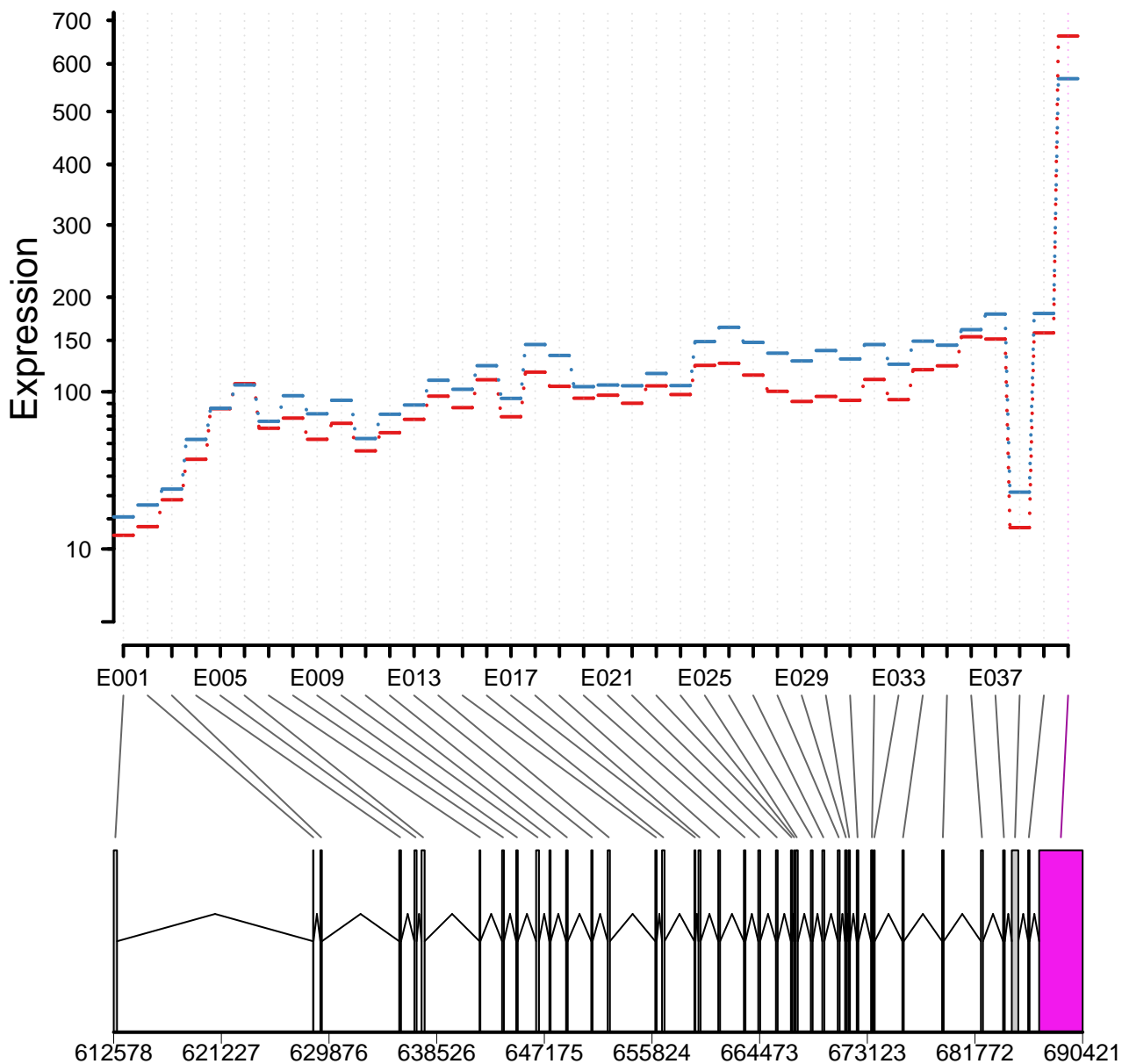

Supplement: Supplementary file 11 [file Data_Sheet_1.ZIP › Supplementary 17/gene-Arfgef2.pdf]

gene-Arhgef12 -

C

S

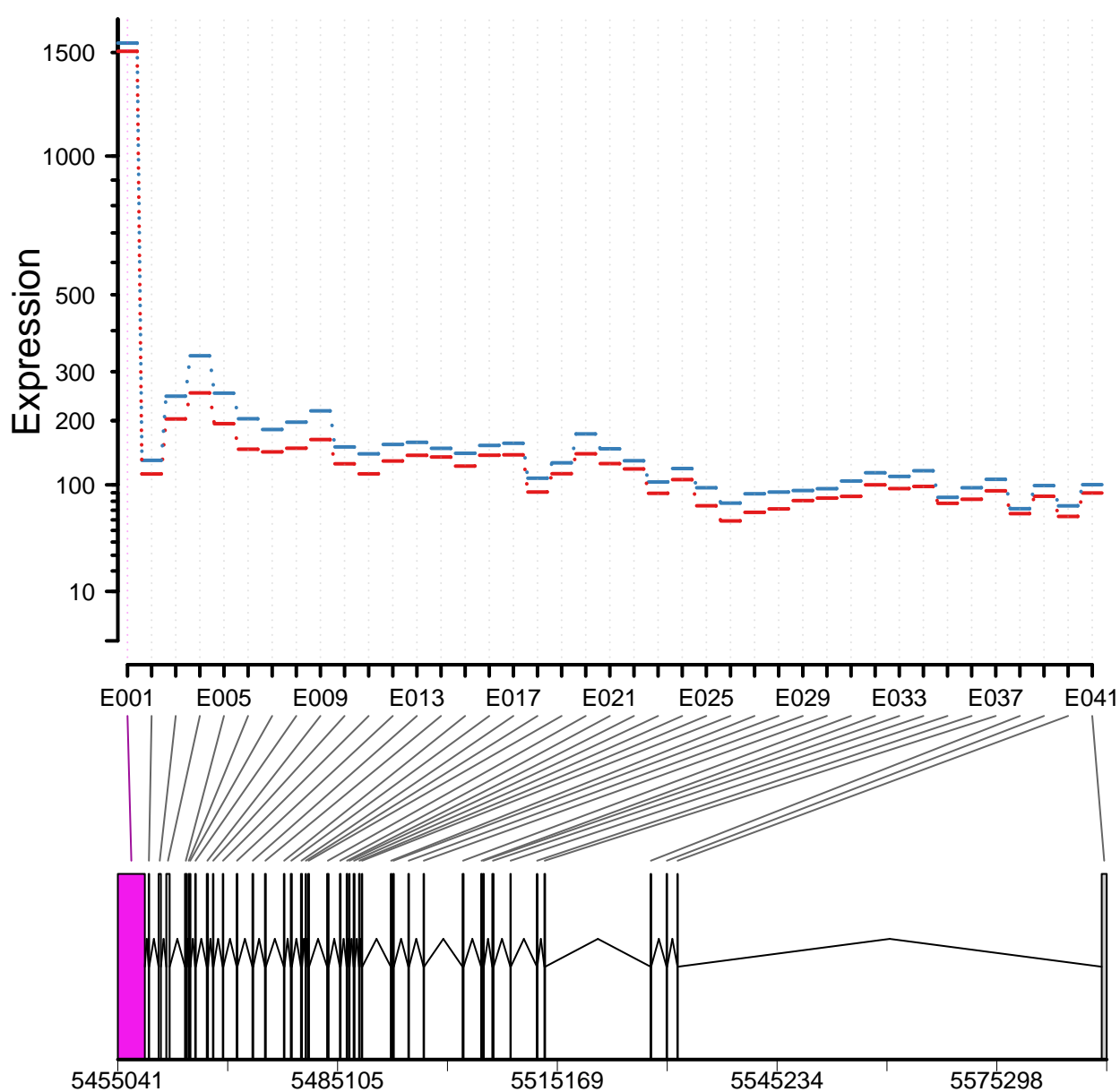

Supplement: Supplementary file 11 [file Data_Sheet_1.ZIP › Supplementary 17/gene-Arhgef12.pdf]

gene-Atp6v1c2 +

C

S

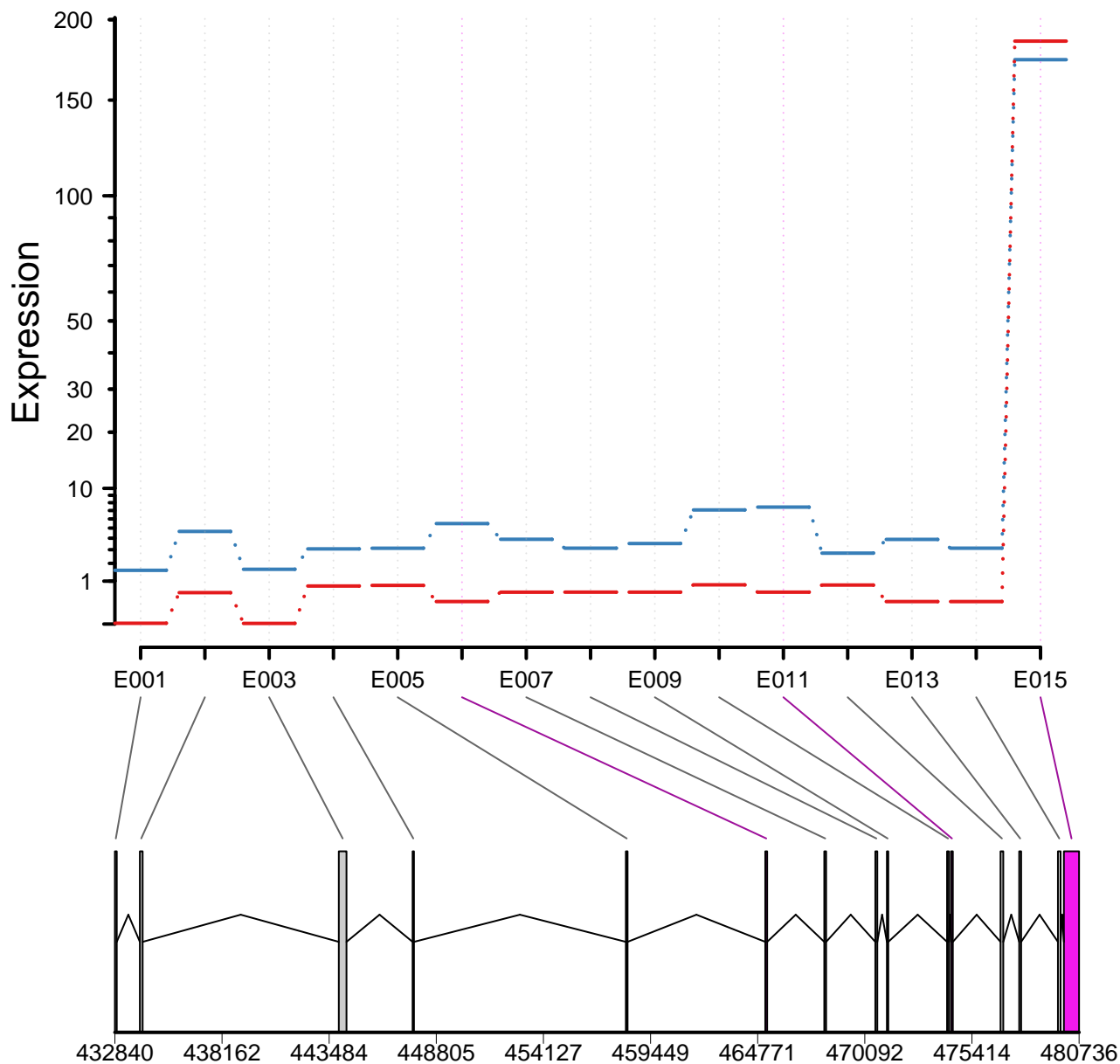

Supplement: Supplementary file 11 [file Data_Sheet_1.ZIP › Supplementary 17/gene-Atp6v1c2.pdf]

gene-Atxn2l +

C

S

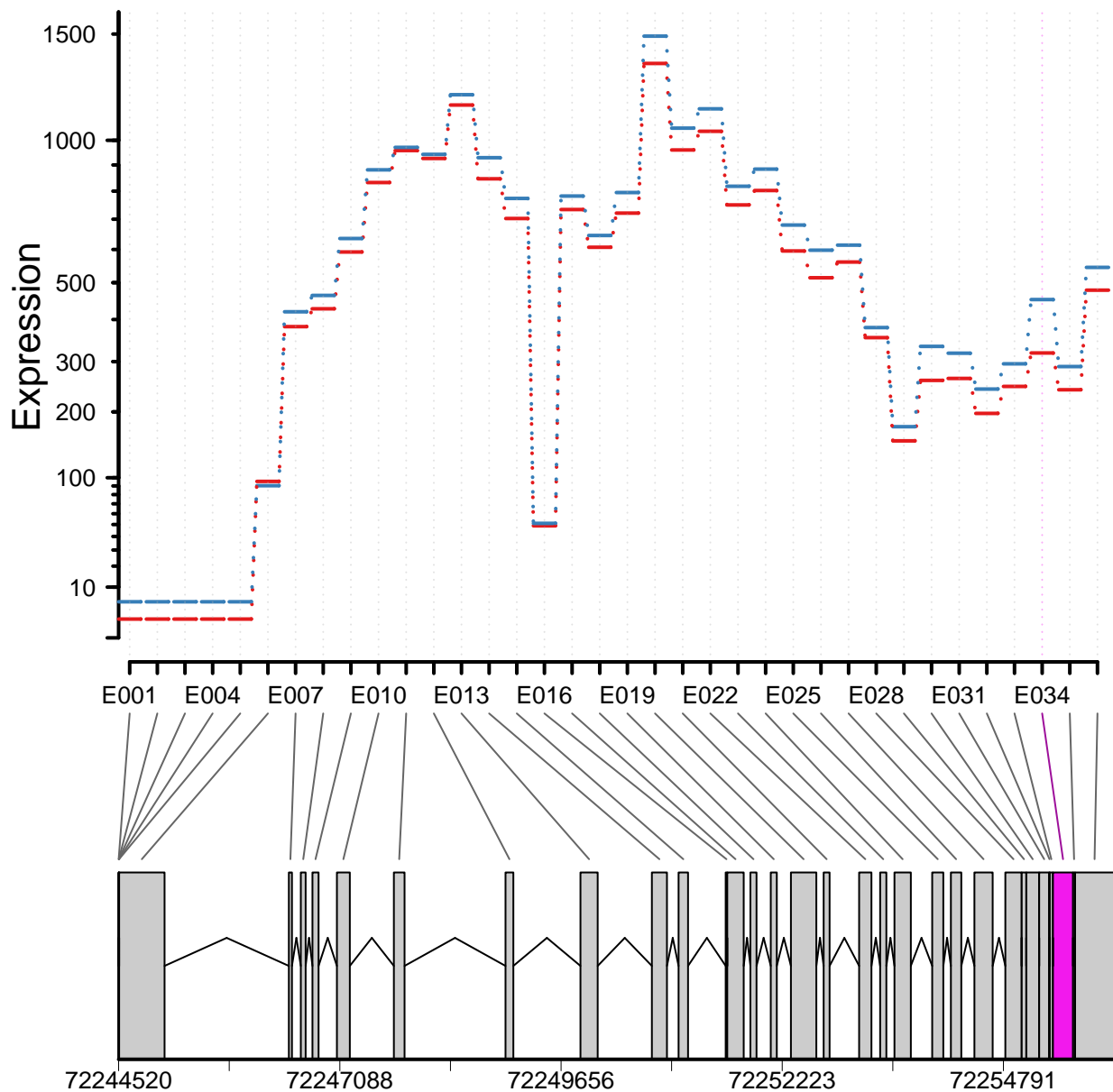

Supplement: Supplementary file 11 [file Data_Sheet_1.ZIP › Supplementary 17/gene-Atxn2l.pdf]

gene-Bclaf1 -

C

S

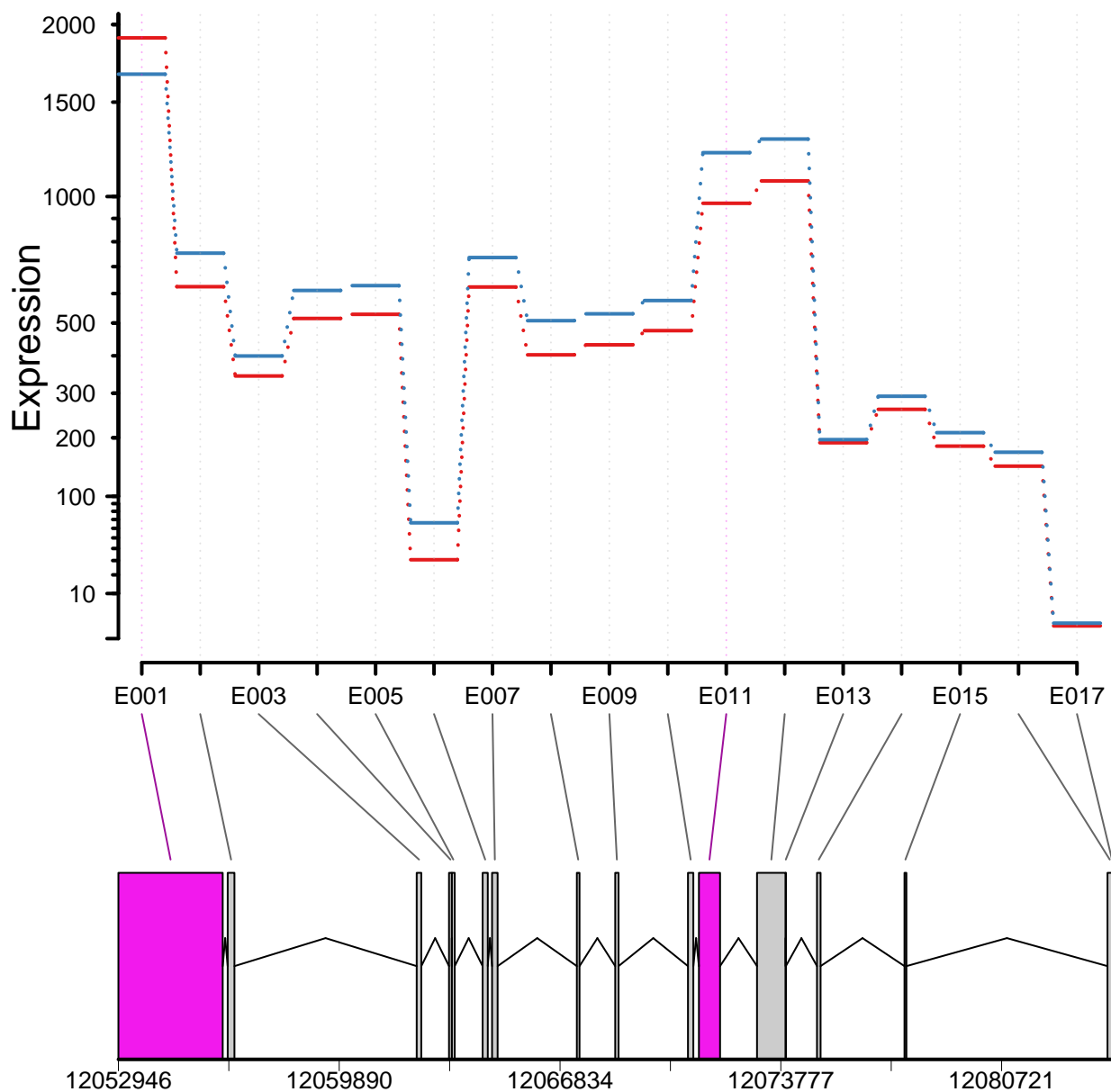

Supplement: Supplementary file 11 [file Data_Sheet_1.ZIP › Supplementary 17/gene-Bclaf1.pdf]

gene-Brd1 -

C

S

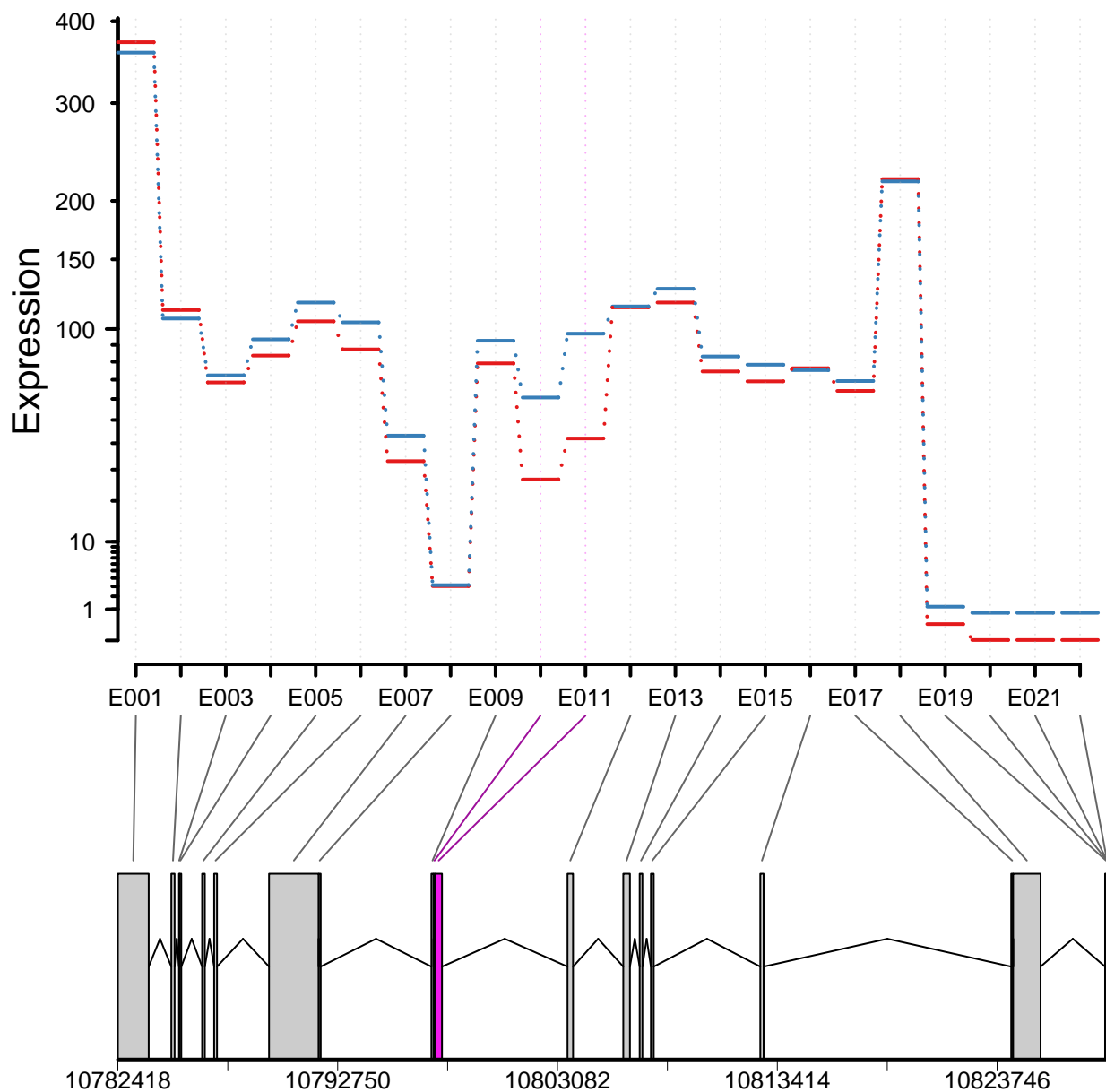

Supplement: Supplementary file 11 [file Data_Sheet_1.ZIP › Supplementary 17/gene-Brd1.pdf]

gene-Brwd1 +

C

S

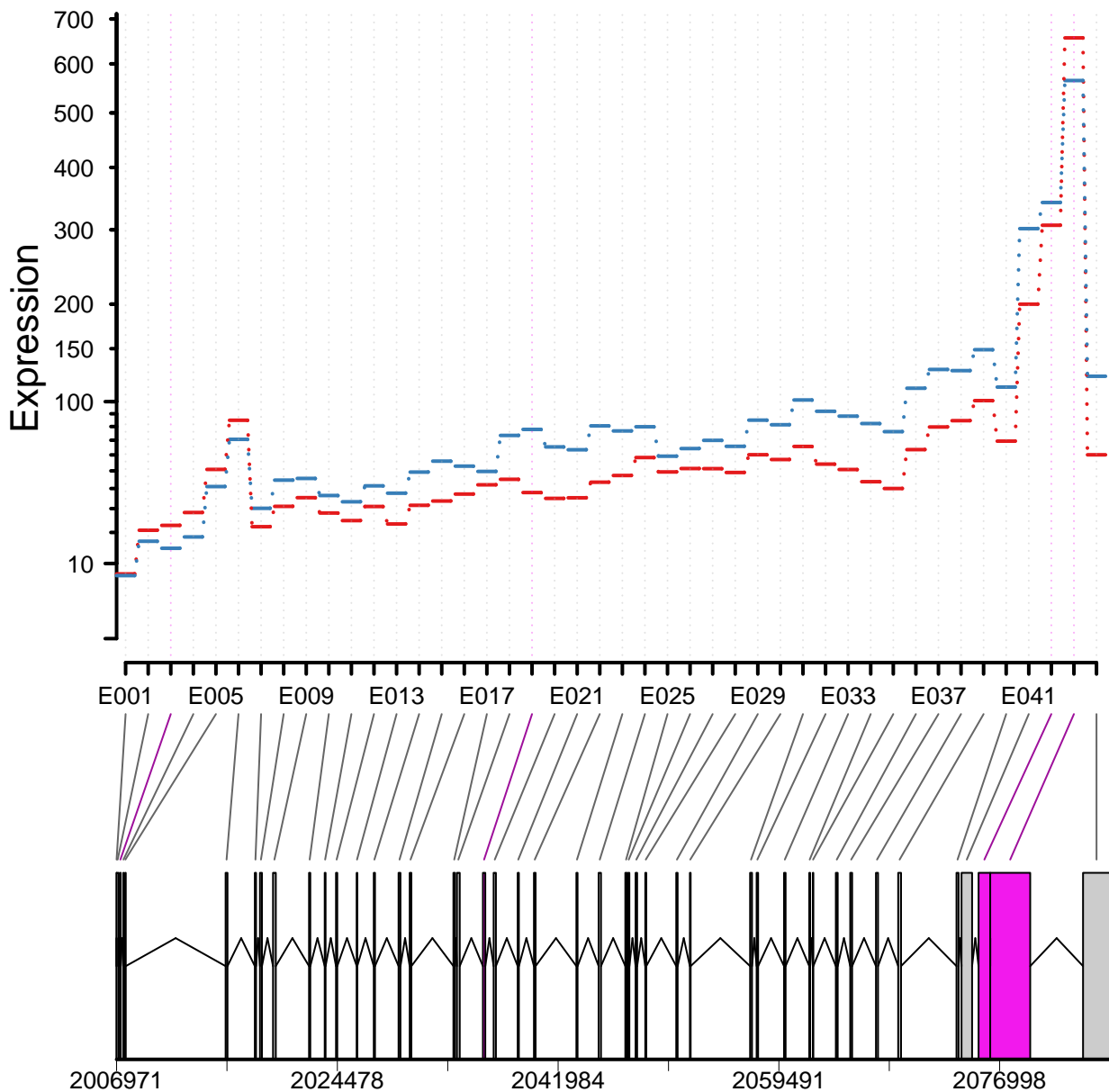

Supplement: Supplementary file 11 [file Data_Sheet_1.ZIP › Supplementary 17/gene-Brwd1.pdf]

gene-Btbd9 -

C

S

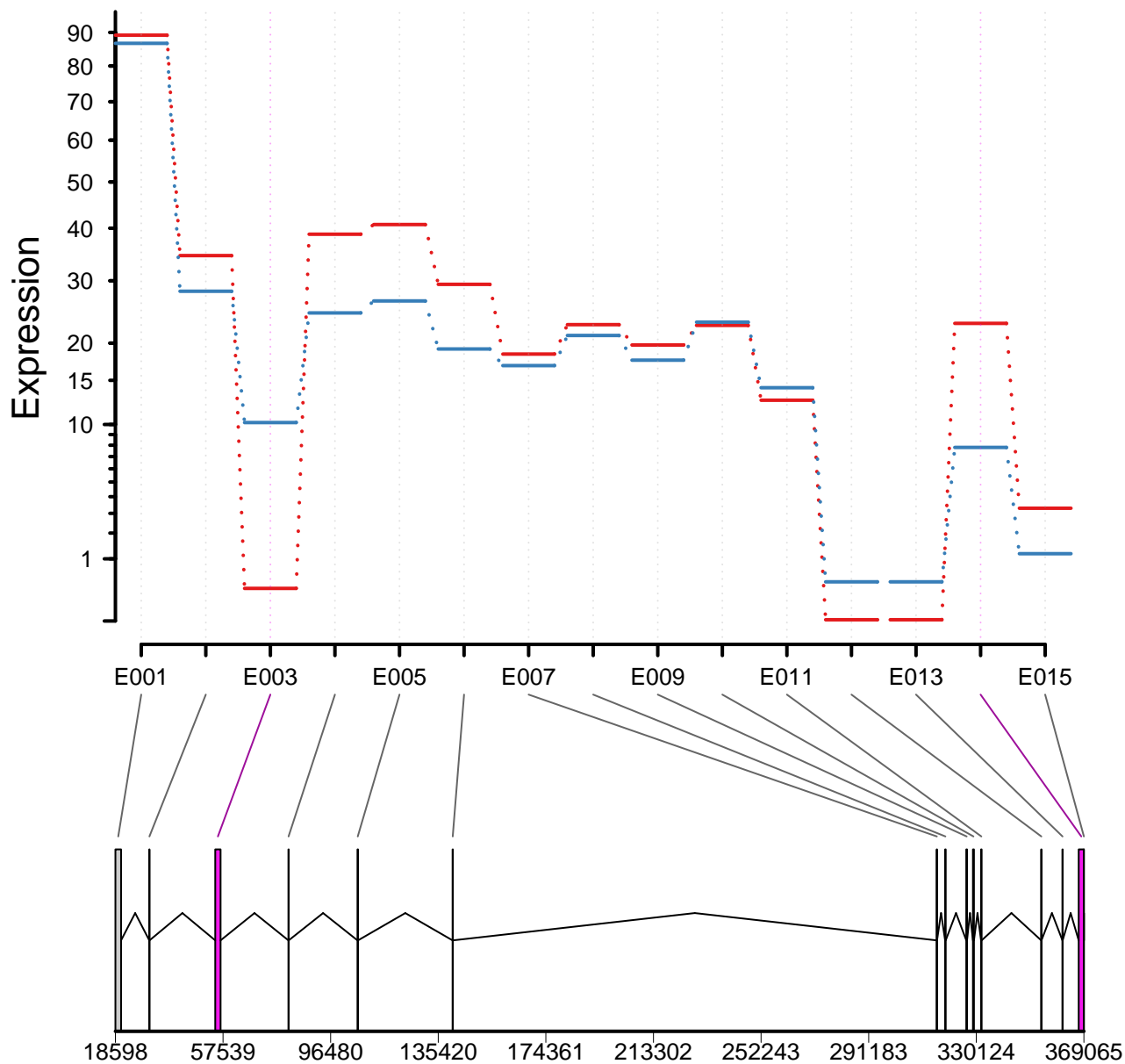

Supplement: Supplementary file 11 [file Data_Sheet_1.ZIP › Supplementary 17/gene-Btbd9.pdf]

gene-Cars +

C

S

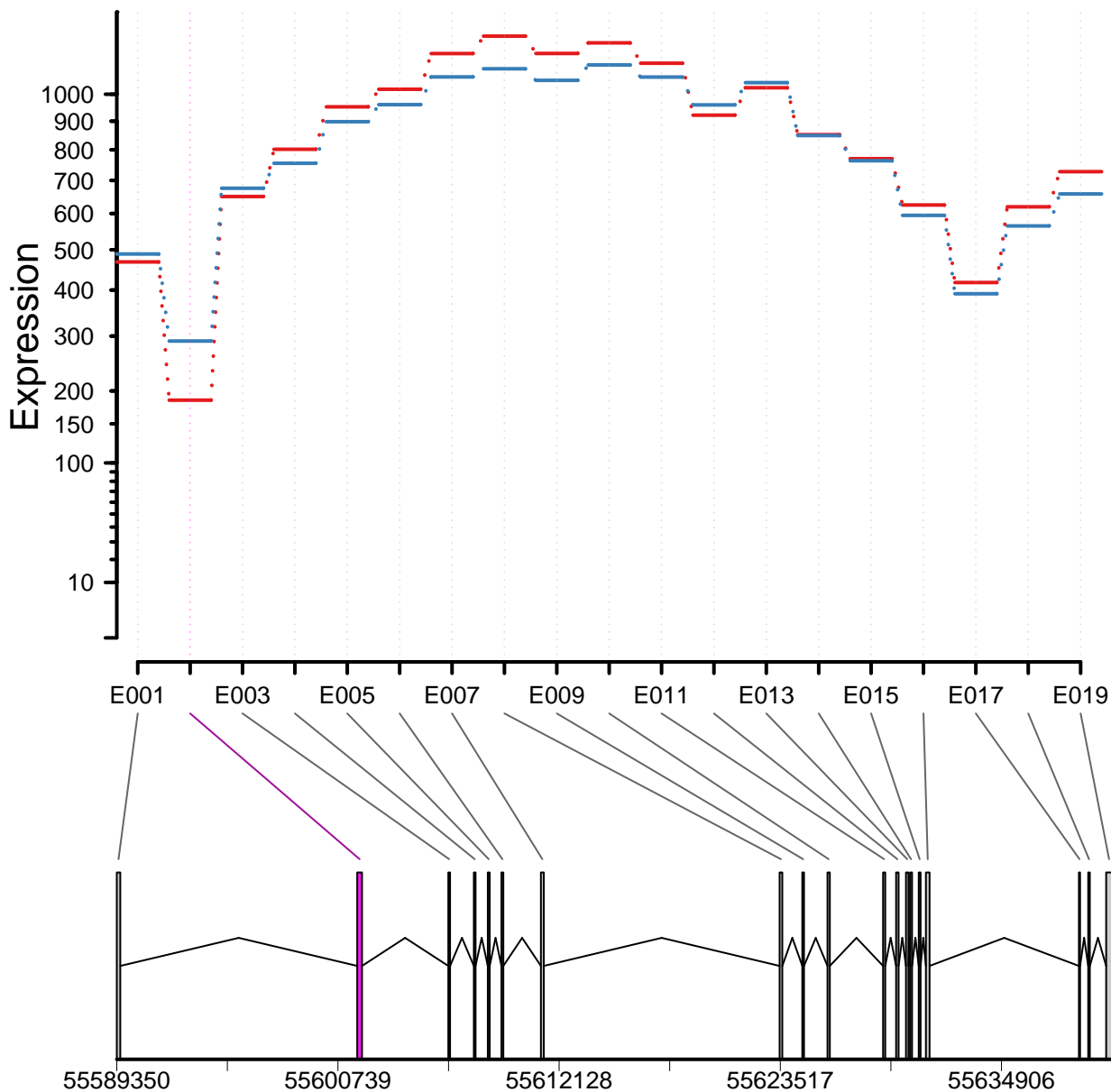

Supplement: Supplementary file 11 [file Data_Sheet_1.ZIP › Supplementary 17/gene-Cars.pdf]

gene-Cd44 +

C

S

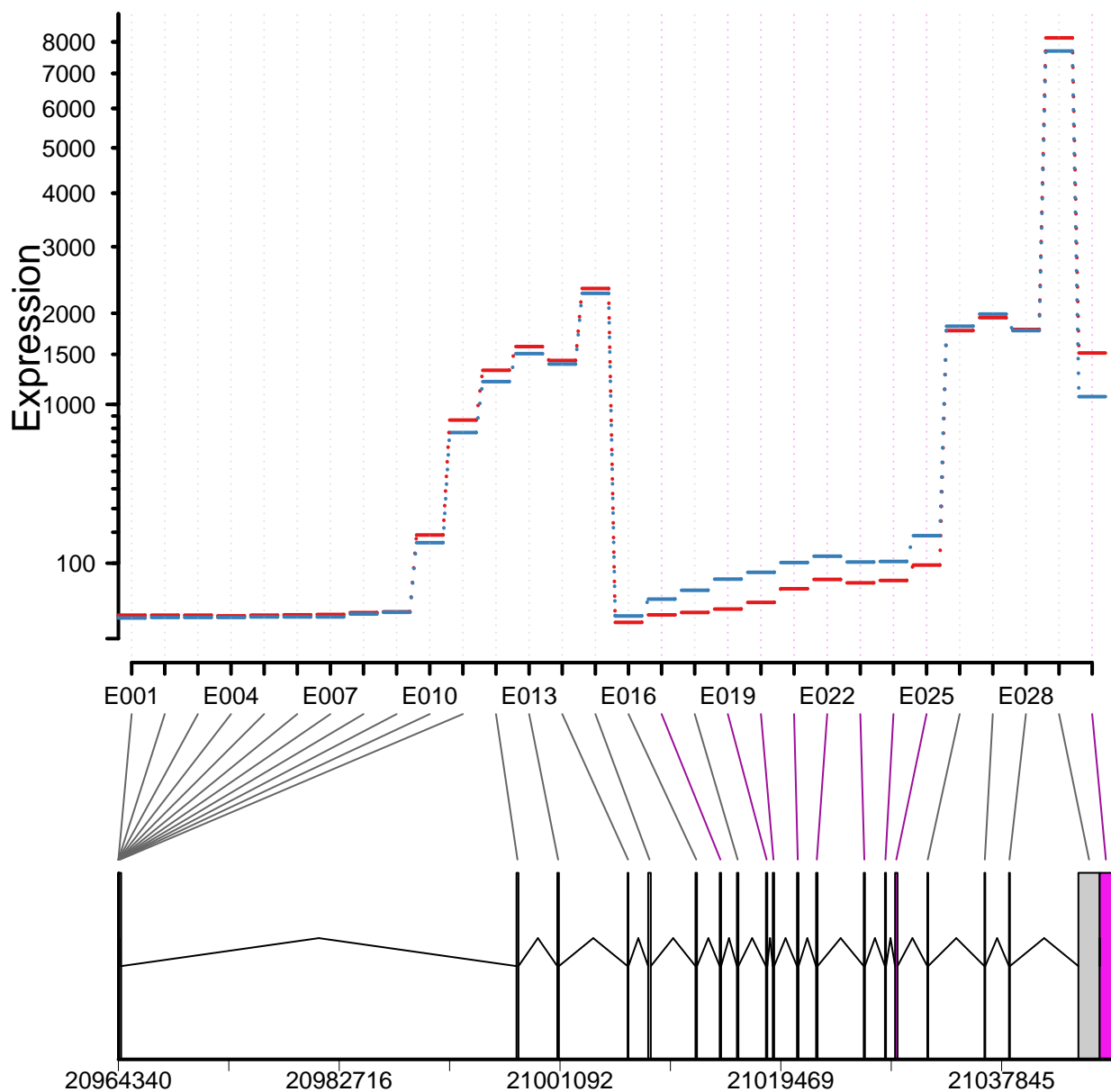

Supplement: Supplementary file 11 [file Data_Sheet_1.ZIP › Supplementary 17/gene-Cd44.pdf]

gene-Cdyl2 -

C

S

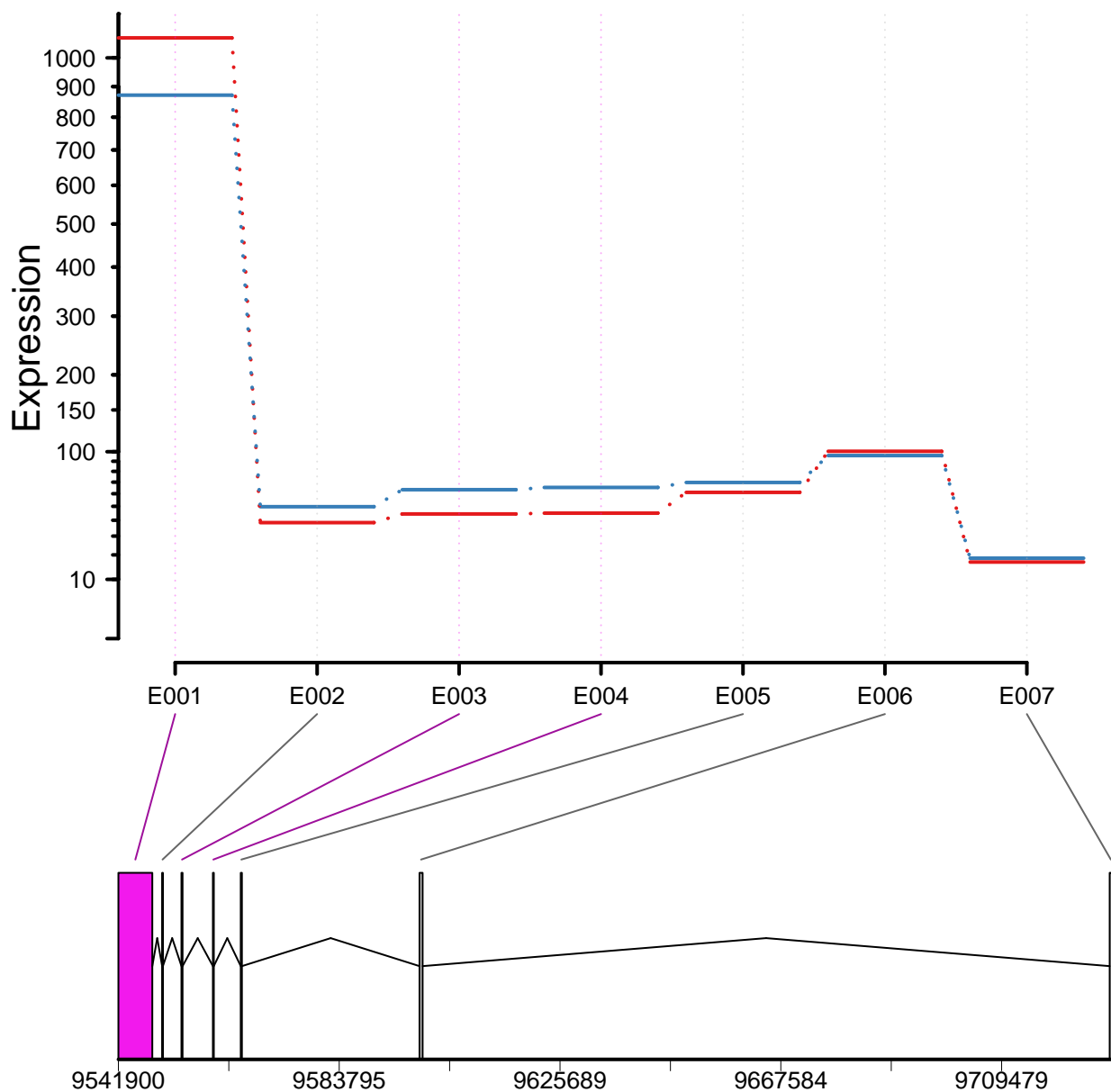

Supplement: Supplementary file 11 [file Data_Sheet_1.ZIP › Supplementary 17/gene-Cdyl2.pdf]

gene-Chka –

C

S

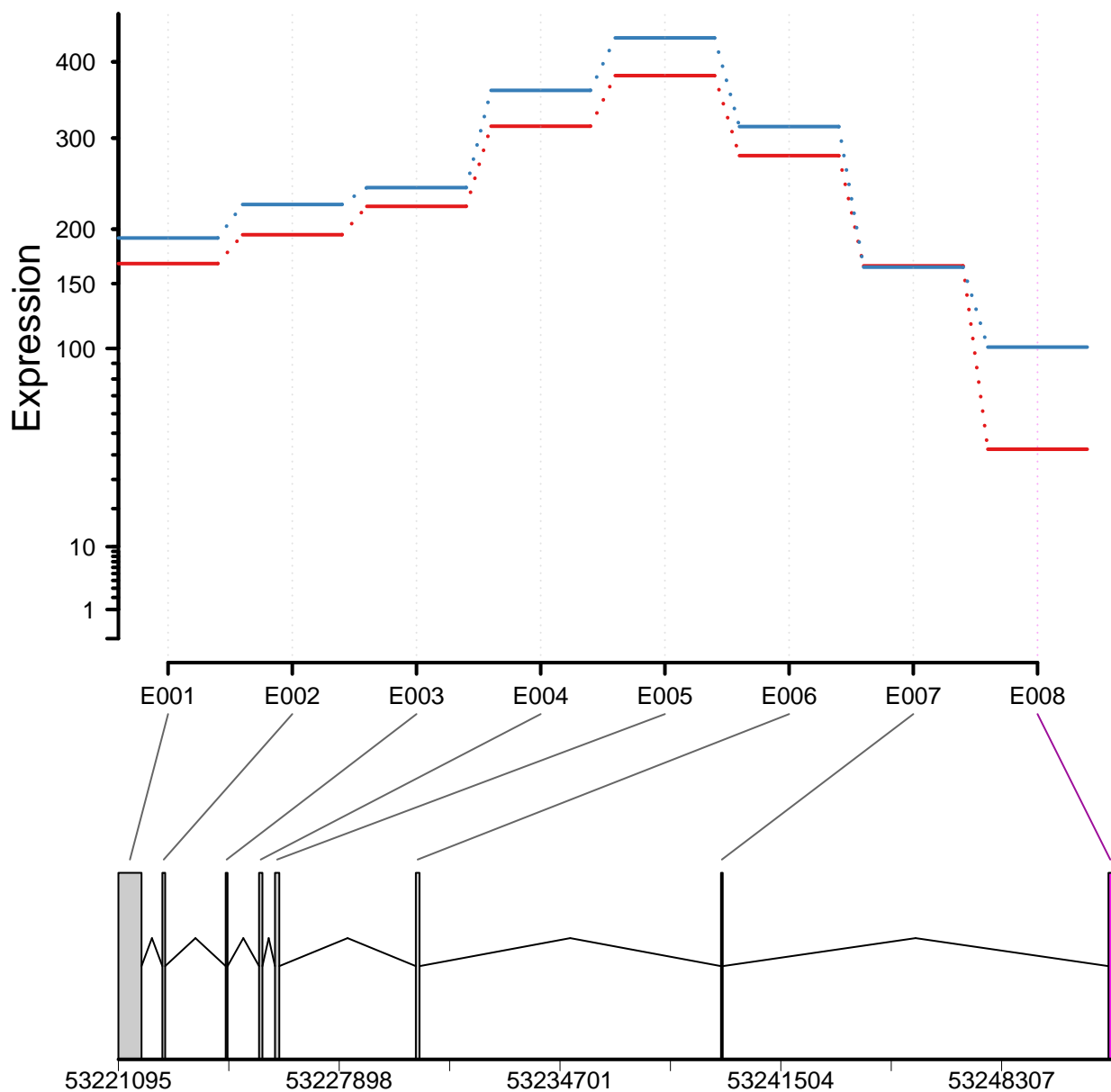

Supplement: Supplementary file 11 [file Data_Sheet_1.ZIP › Supplementary 17/gene-Chka.pdf]

gene-Clk1 +

C

S

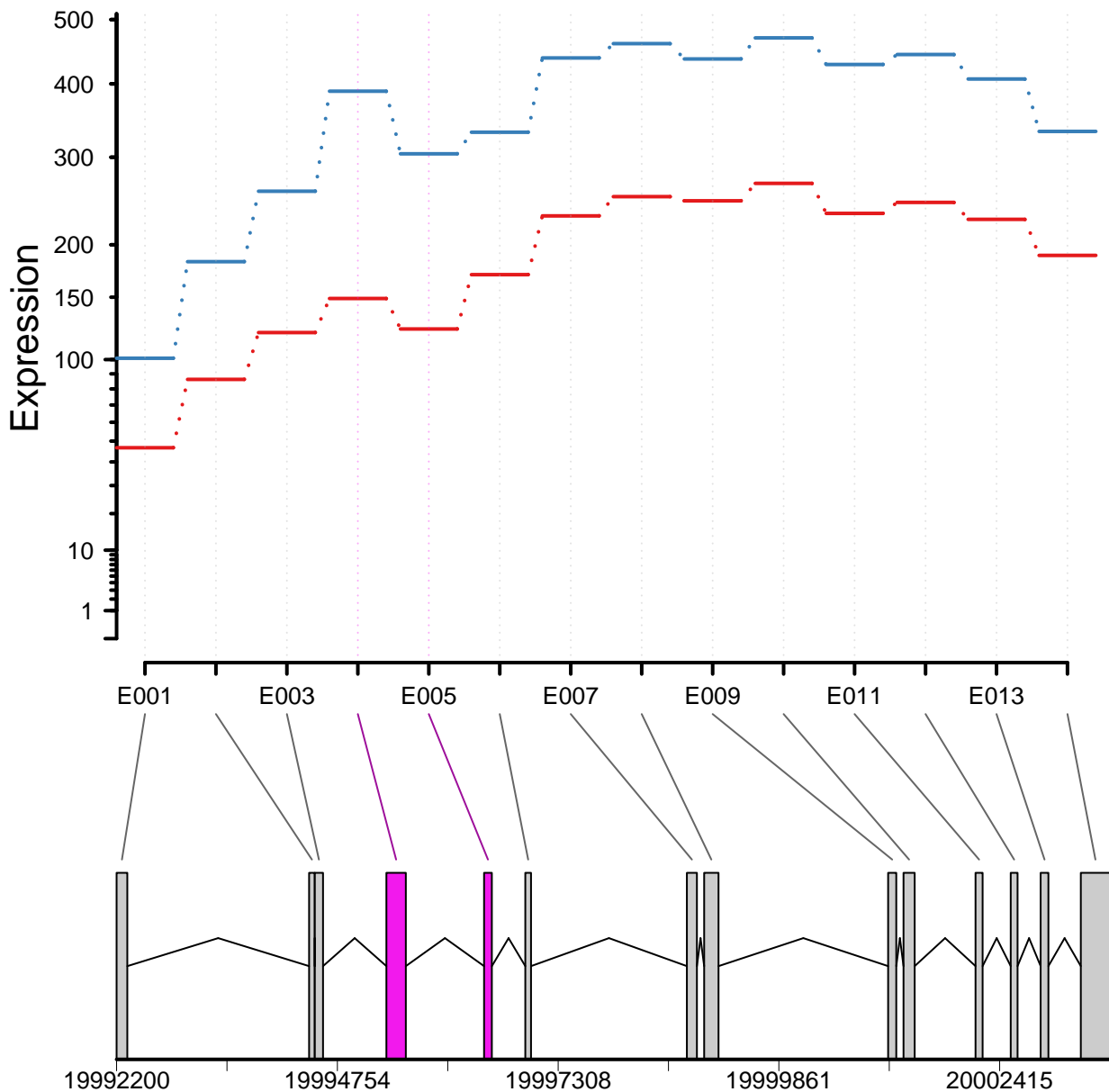

Supplement: Supplementary file 11 [file Data_Sheet_1.ZIP › Supplementary 17/gene-Clk1.pdf]

gene-Clk4 -

C

S

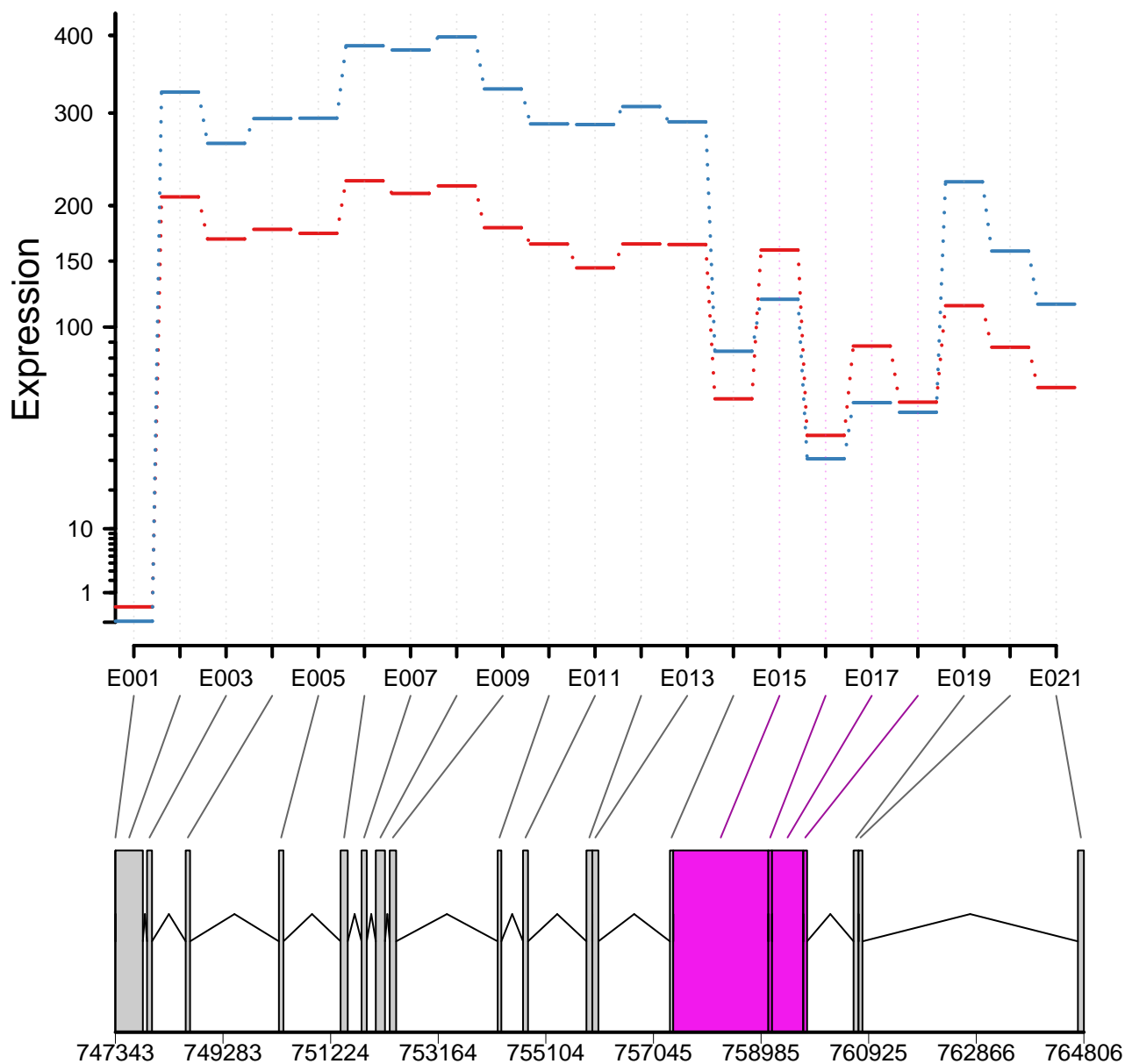

Supplement: Supplementary file 11 [file Data_Sheet_1.ZIP › Supplementary 17/gene-Clk4.pdf]

gene-Cnot4 -

C

S

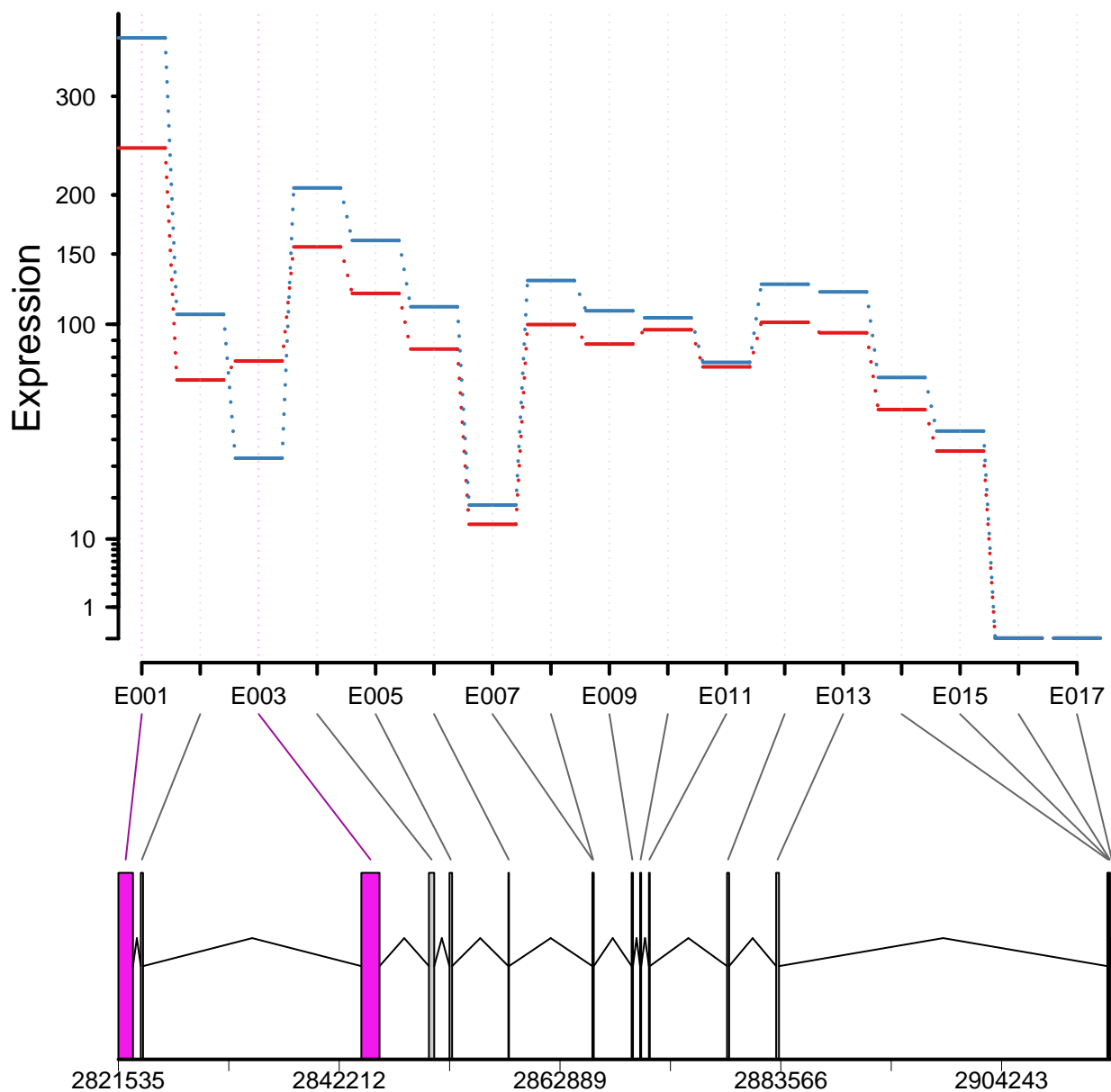

Supplement: Supplementary file 11 [file Data_Sheet_1.ZIP › Supplementary 17/gene-Cnot4.pdf]

S

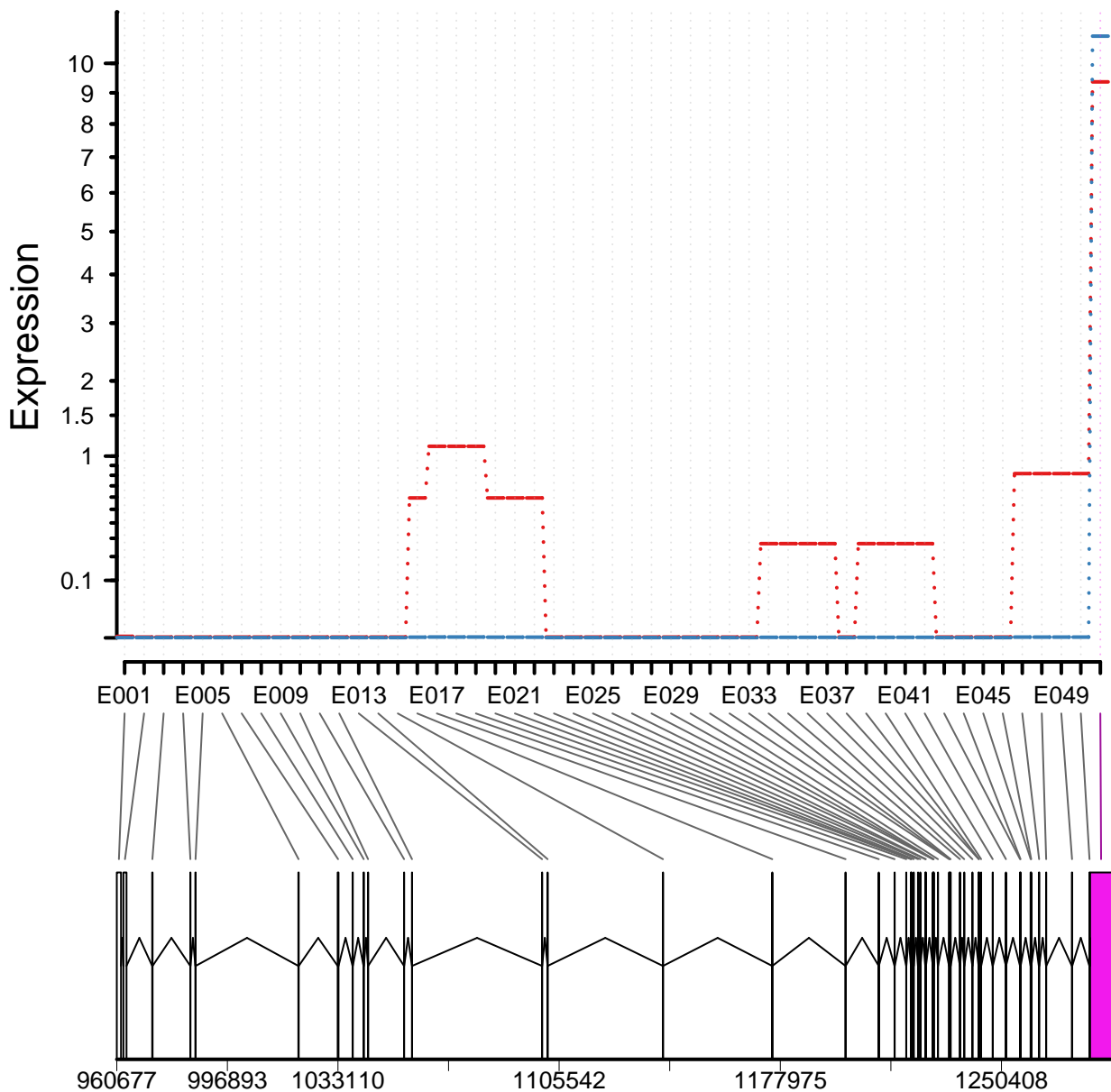

Supplement: Supplementary file 11 [file Data_Sheet_1.ZIP › Supplementary 17/gene-Col19a1.pdf]

gene-Cpne1 +

C

S

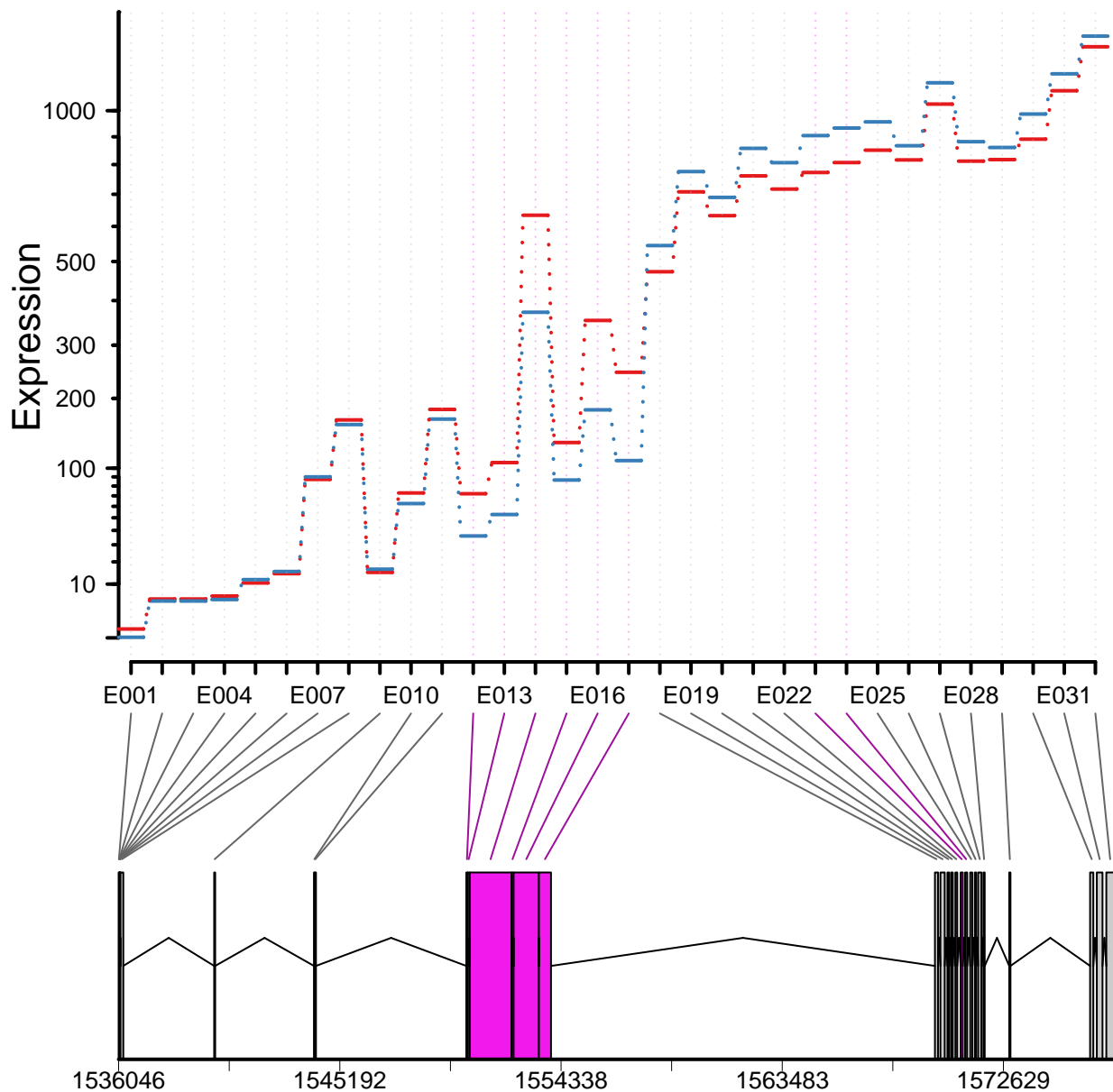

Supplement: Supplementary file 11 [file Data_Sheet_1.ZIP › Supplementary 17/gene-Cpne1.pdf]

gene-Ctnnb1 -

C

S

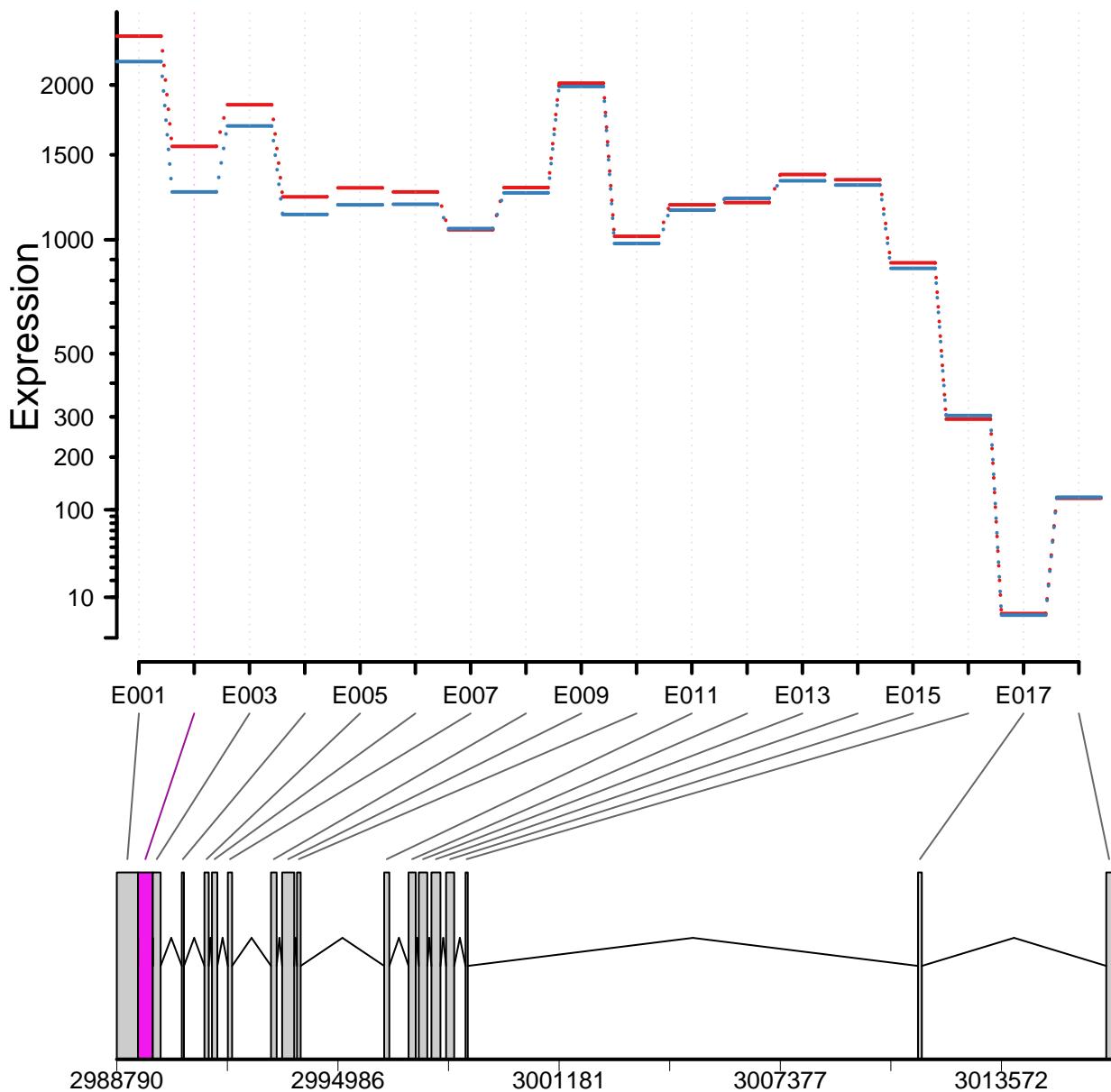

Supplement: Supplementary file 11 [file Data_Sheet_1.ZIP › Supplementary 17/gene-Ctnnb1.pdf]

gene-Cuzd1 +

C

S

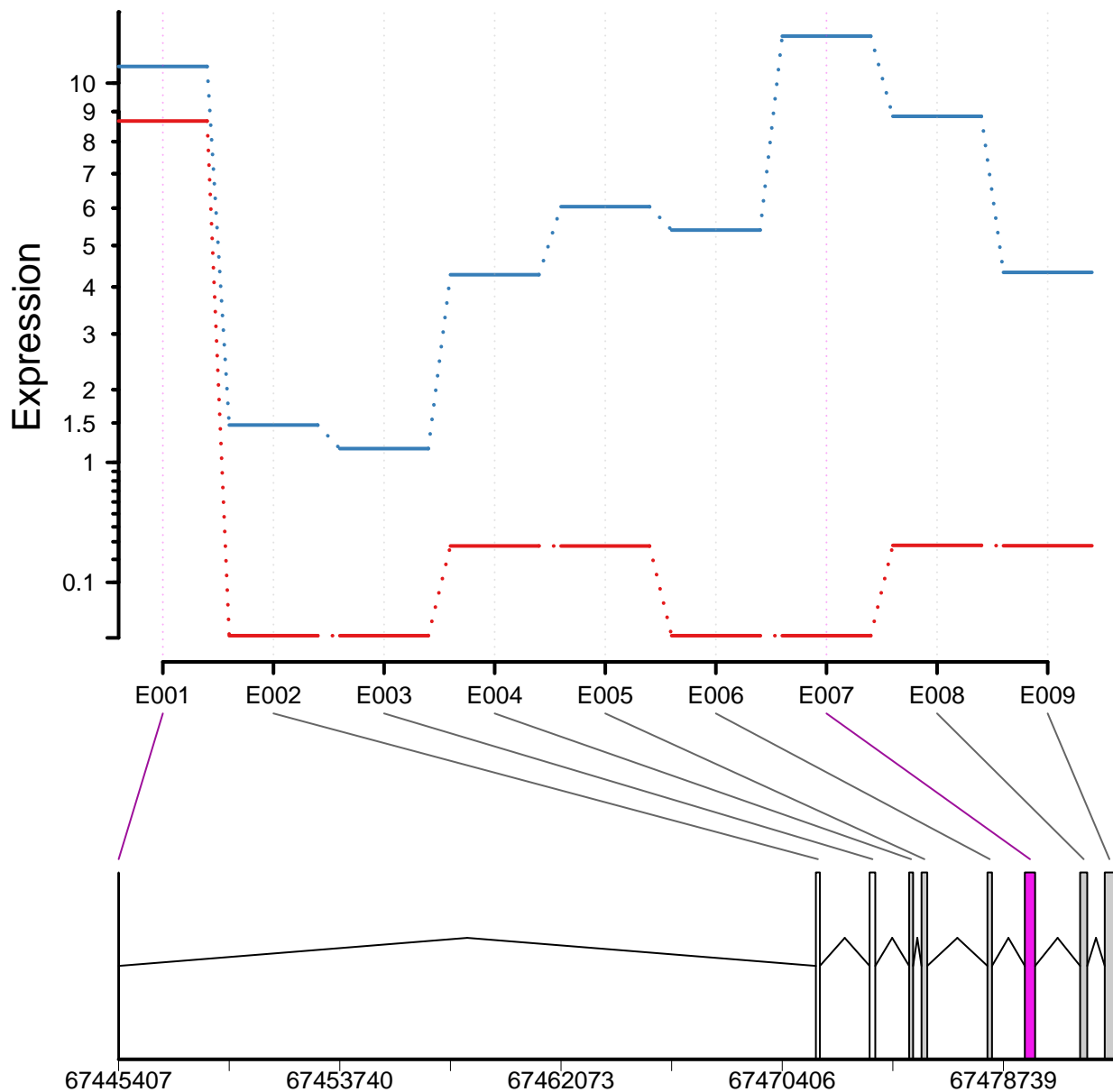

Supplement: Supplementary file 11 [file Data_Sheet_1.ZIP › Supplementary 17/gene-Cuzd1.pdf]

gene-Cyld -

C

S

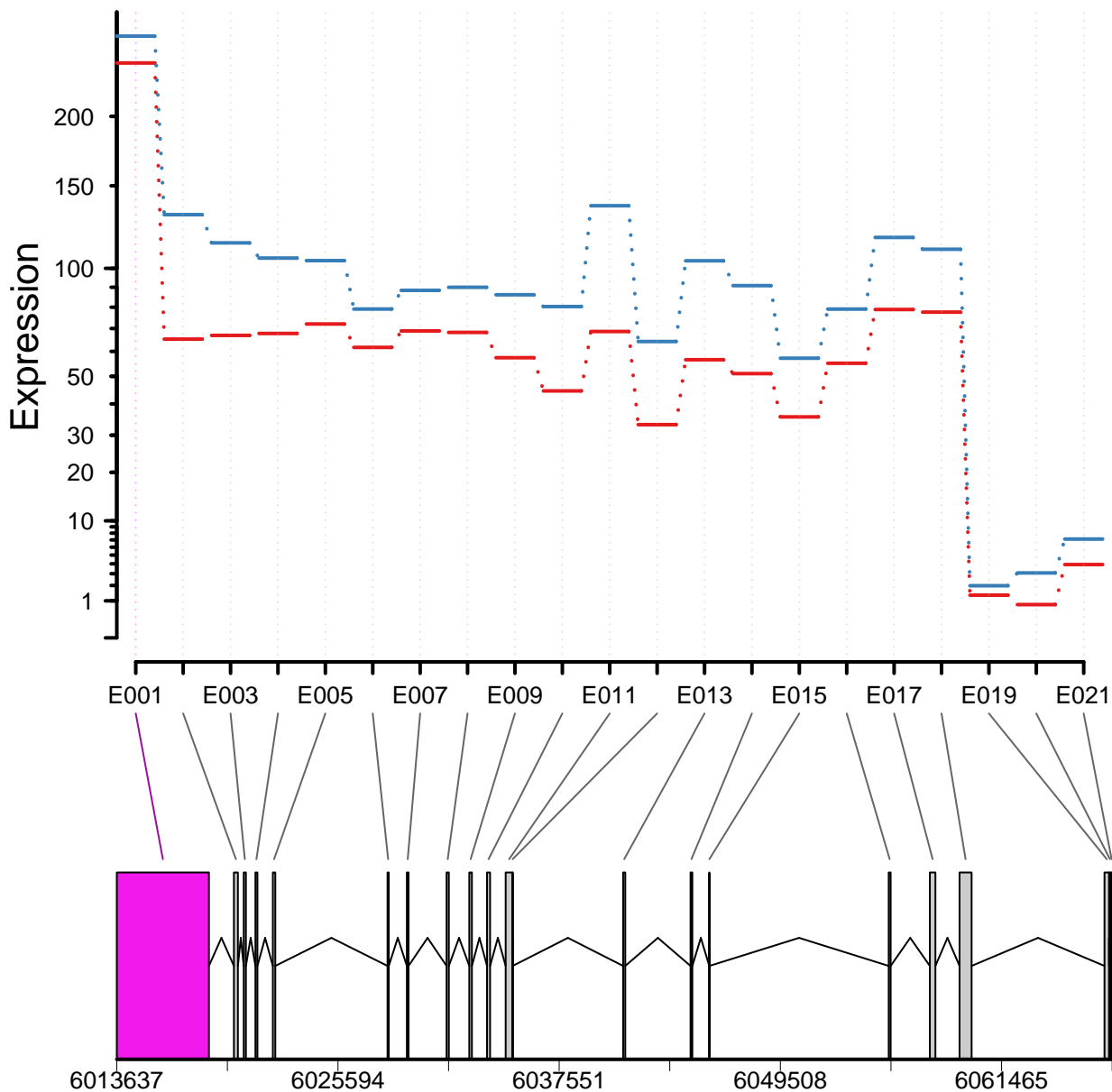

Supplement: Supplementary file 11 [file Data_Sheet_1.ZIP › Supplementary 17/gene-Cyld.pdf]

gene-Dclk1 +

C

S

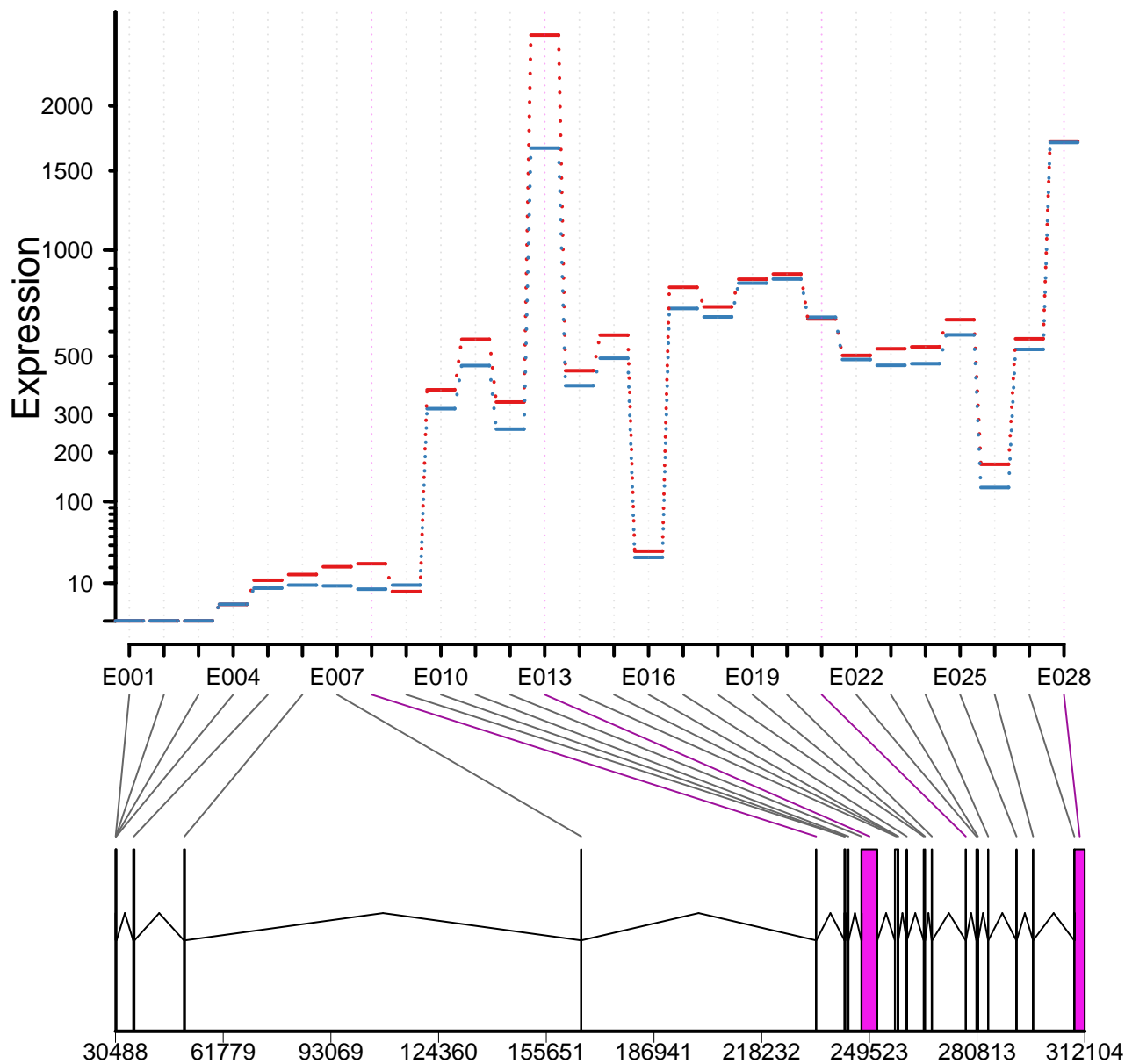

Supplement: Supplementary file 11 [file Data_Sheet_1.ZIP › Supplementary 17/gene-Dclk1.pdf]

gene-Ddr2 -

C

S

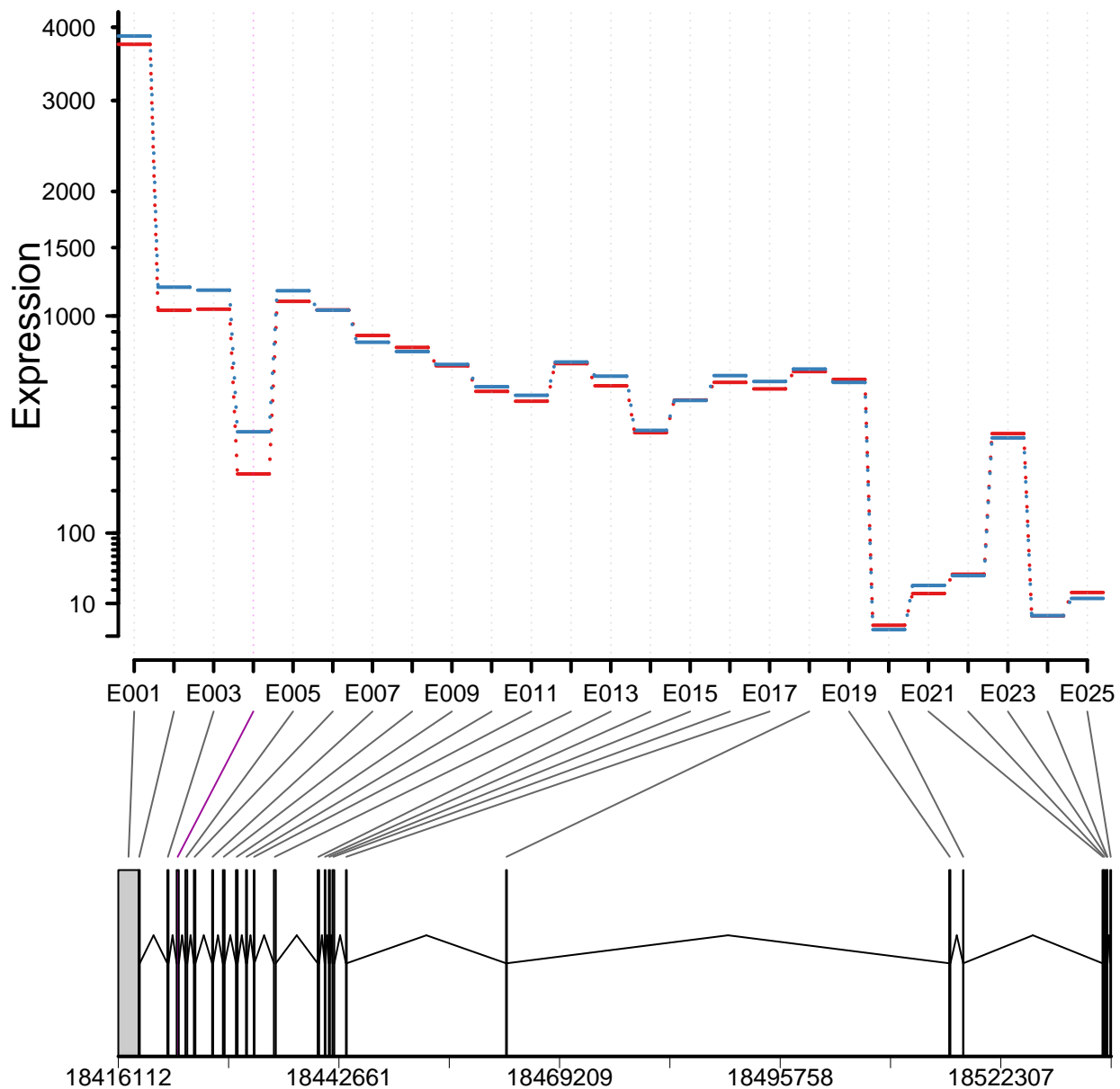

Supplement: Supplementary file 11 [file Data_Sheet_1.ZIP › Supplementary 17/gene-Ddr2.pdf]

gene-Dido1 -

C

S

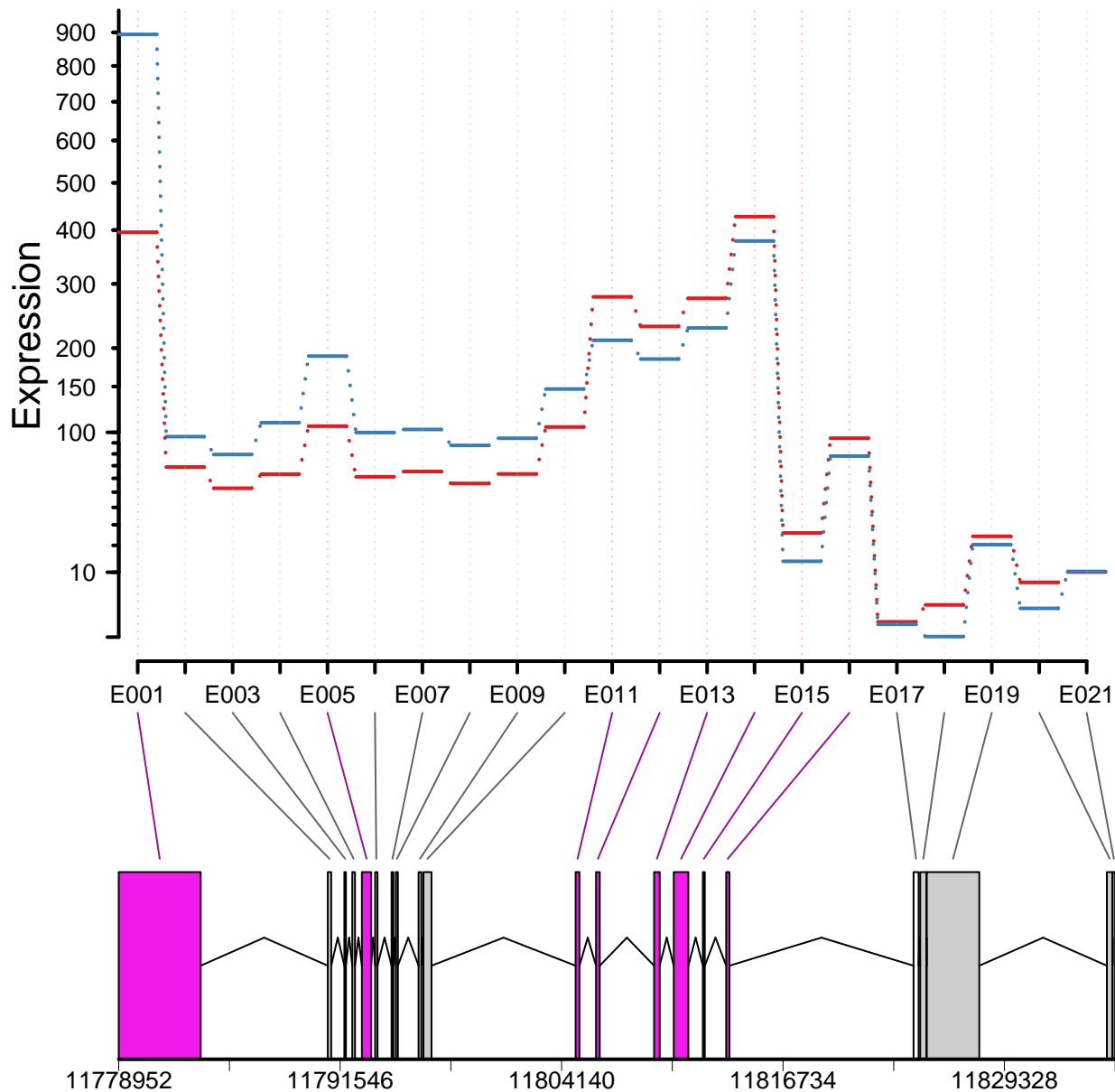

Supplement: Supplementary file 11 [file Data_Sheet_1.ZIP › Supplementary 17/gene-Dido1.pdf]

gene-Eea1 +

C

S

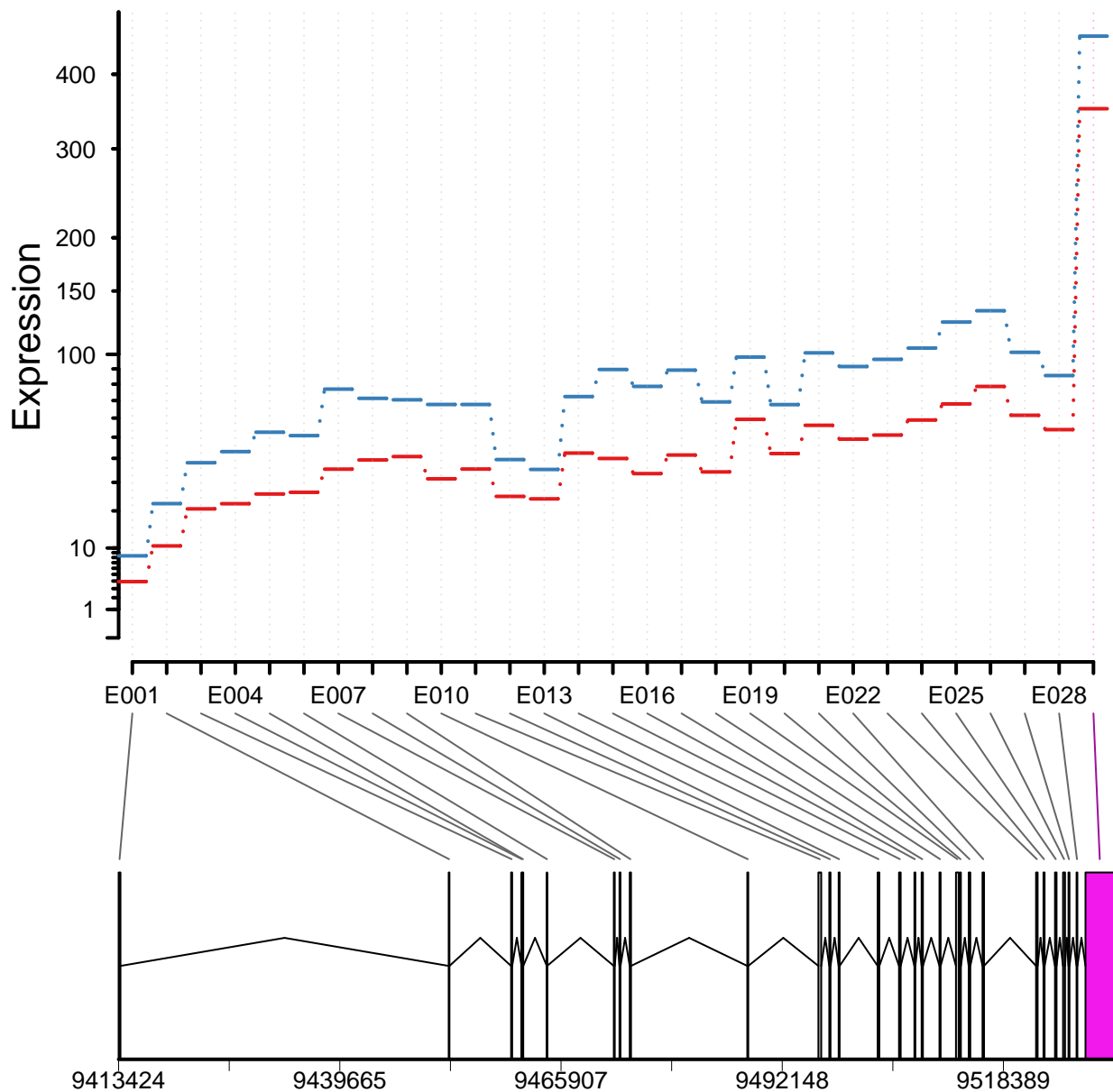

Supplement: Supplementary file 11 [file Data_Sheet_1.ZIP › Supplementary 17/gene-Eea1.pdf]

gene-Ets1 -

C

S

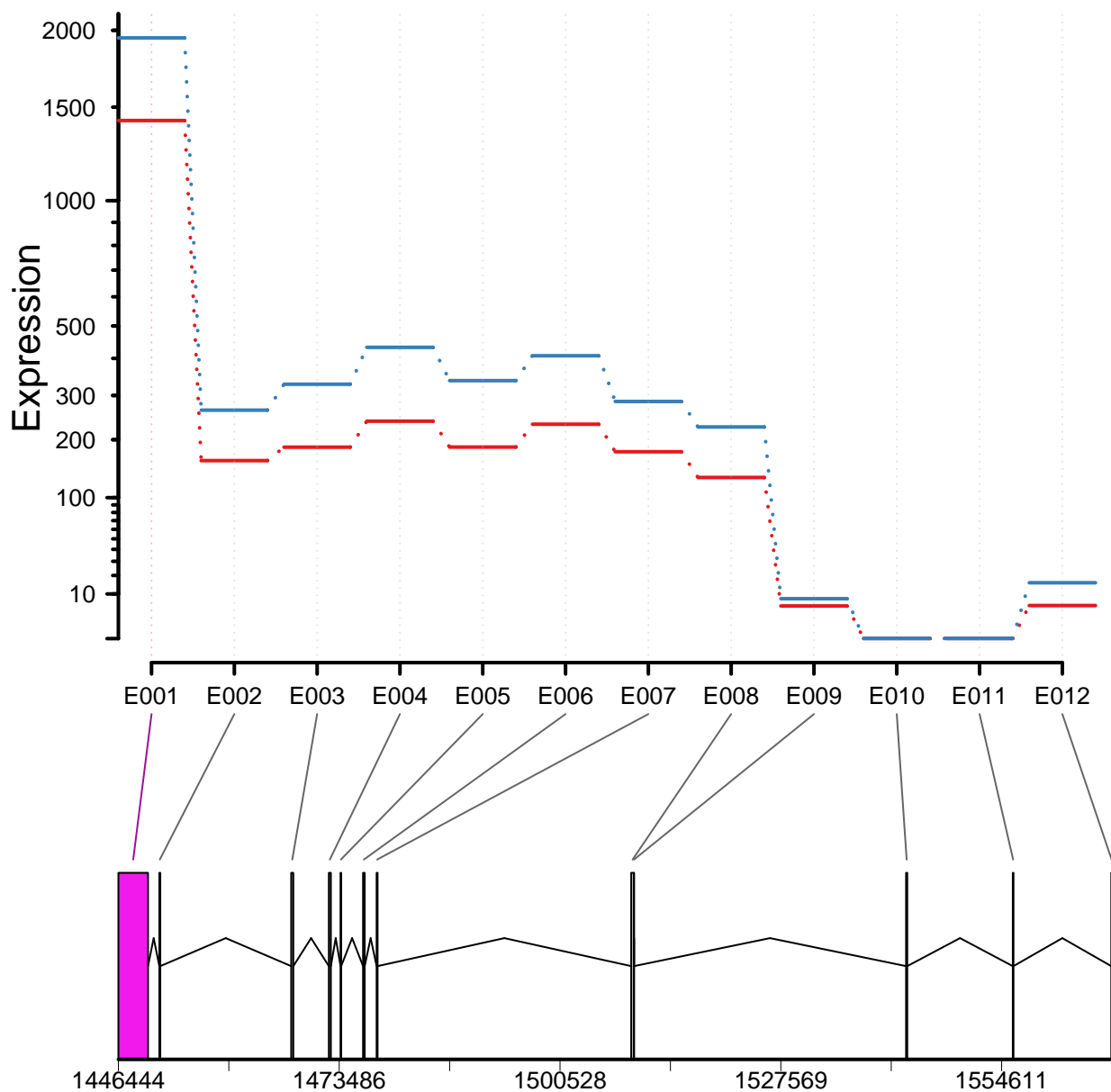

Supplement: Supplementary file 11 [file Data_Sheet_1.ZIP › Supplementary 17/gene-Ets1.pdf]

gene-Fnbp4 -

C

S

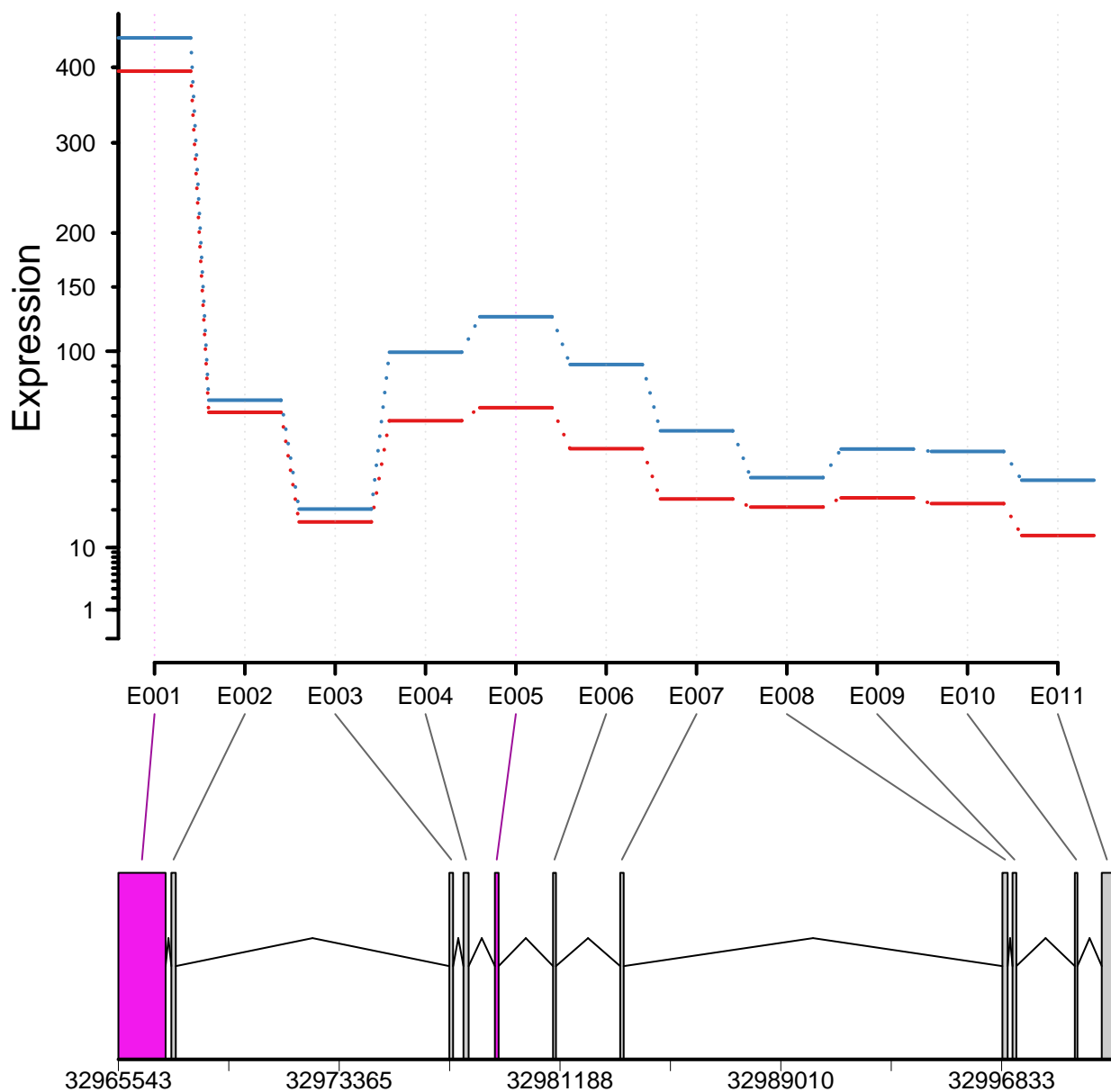

Supplement: Supplementary file 11 [file Data_Sheet_1.ZIP › Supplementary 17/gene-Fnbp4.pdf]

gene-Gapvd1 -

C

S

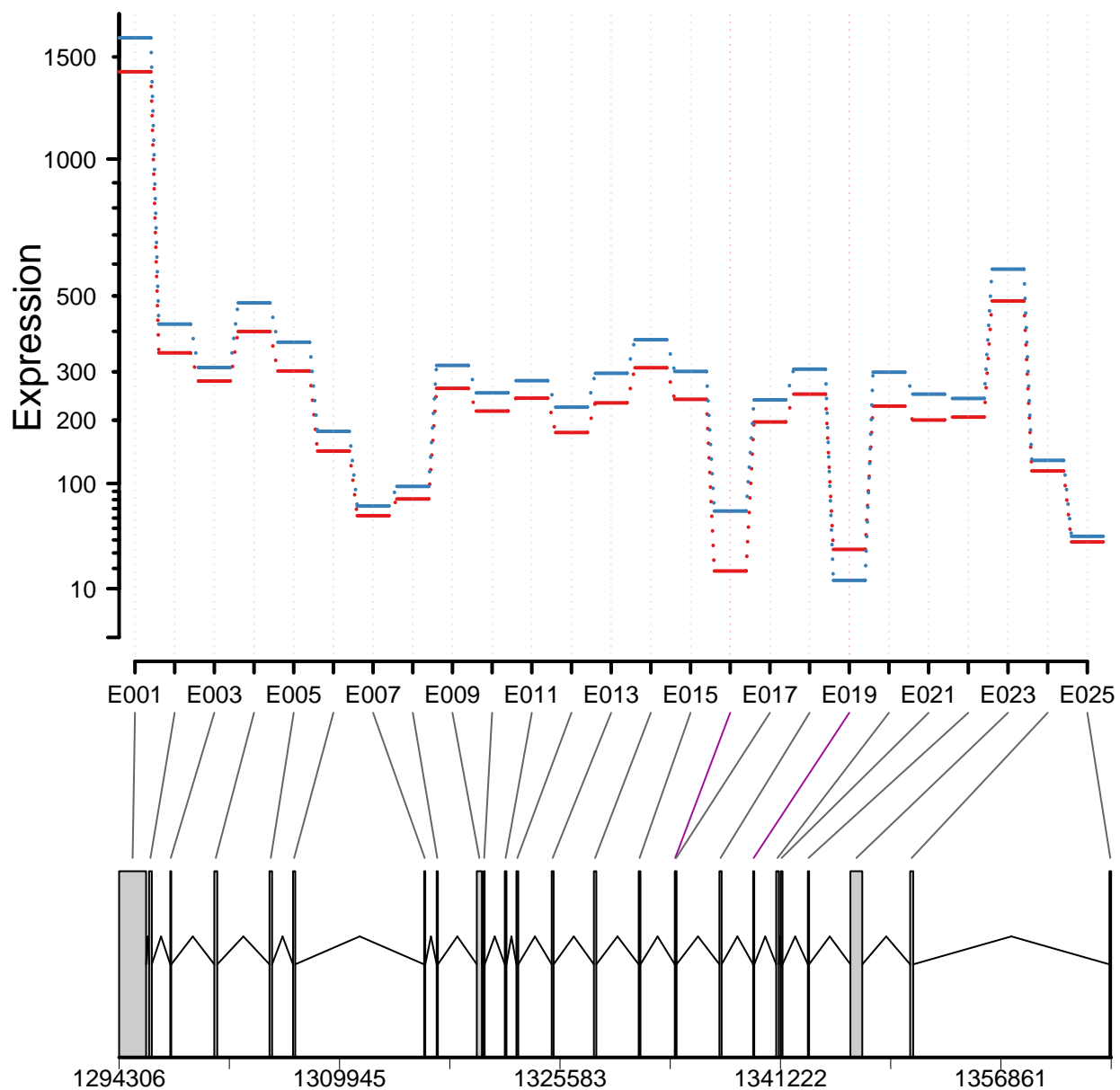

Supplement: Supplementary file 11 [file Data_Sheet_1.ZIP › Supplementary 17/gene-Gapvd1.pdf]

gene-GIs +

C

S

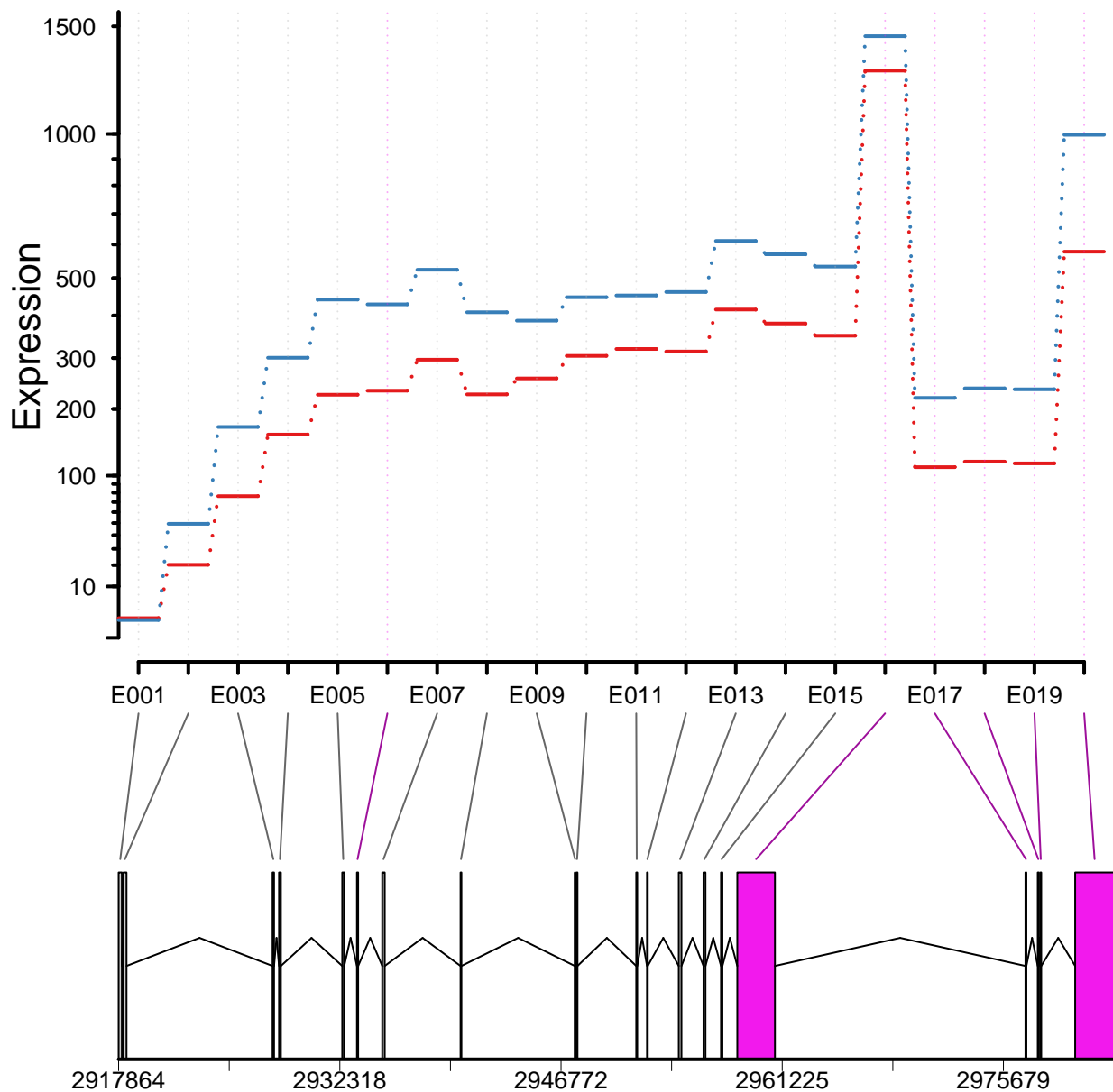

Supplement: Supplementary file 11 [file Data_Sheet_1.ZIP › Supplementary 17/gene-Gls.pdf]

gene-Hnnpa2b1 +

C

S

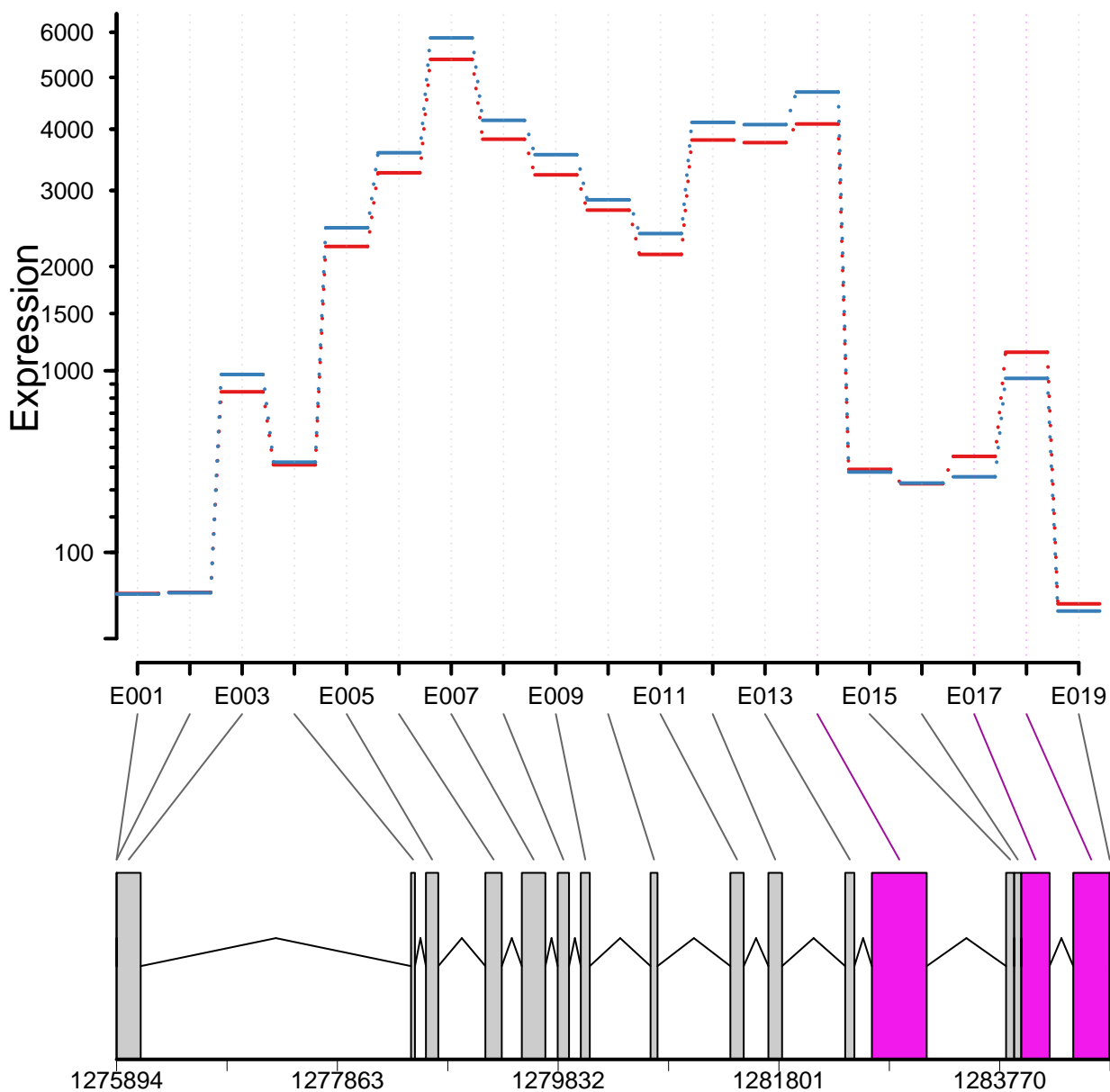

Supplement: Supplementary file 11 [file Data_Sheet_1.ZIP › Supplementary 17/gene-Hnrnpa2b1.pdf]

gene-Hnrnpc +

C

S

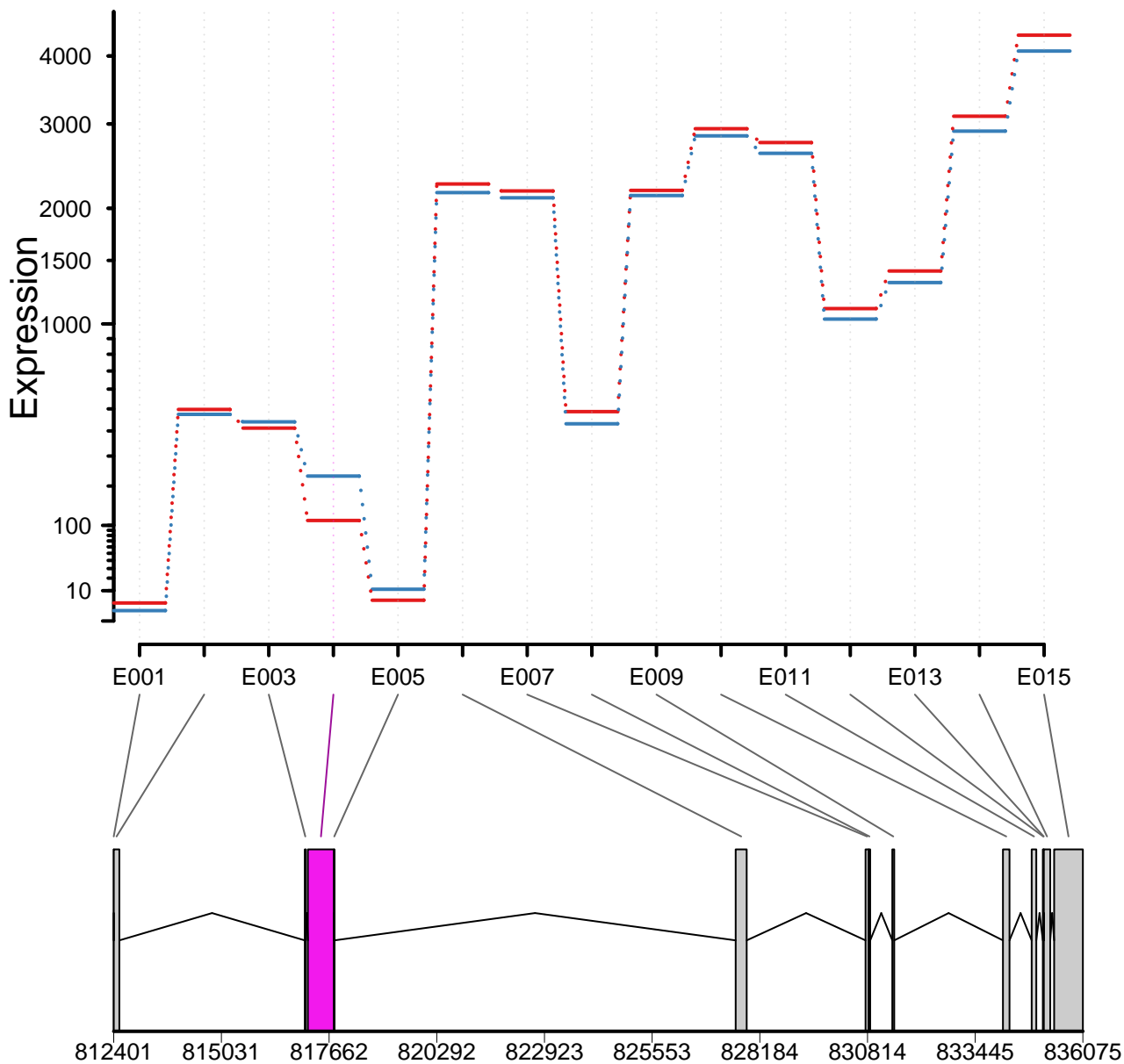

Supplement: Supplementary file 11 [file Data_Sheet_1.ZIP › Supplementary 17/gene-Hnrnpc.pdf]

gene-Huwe1 -

C

S

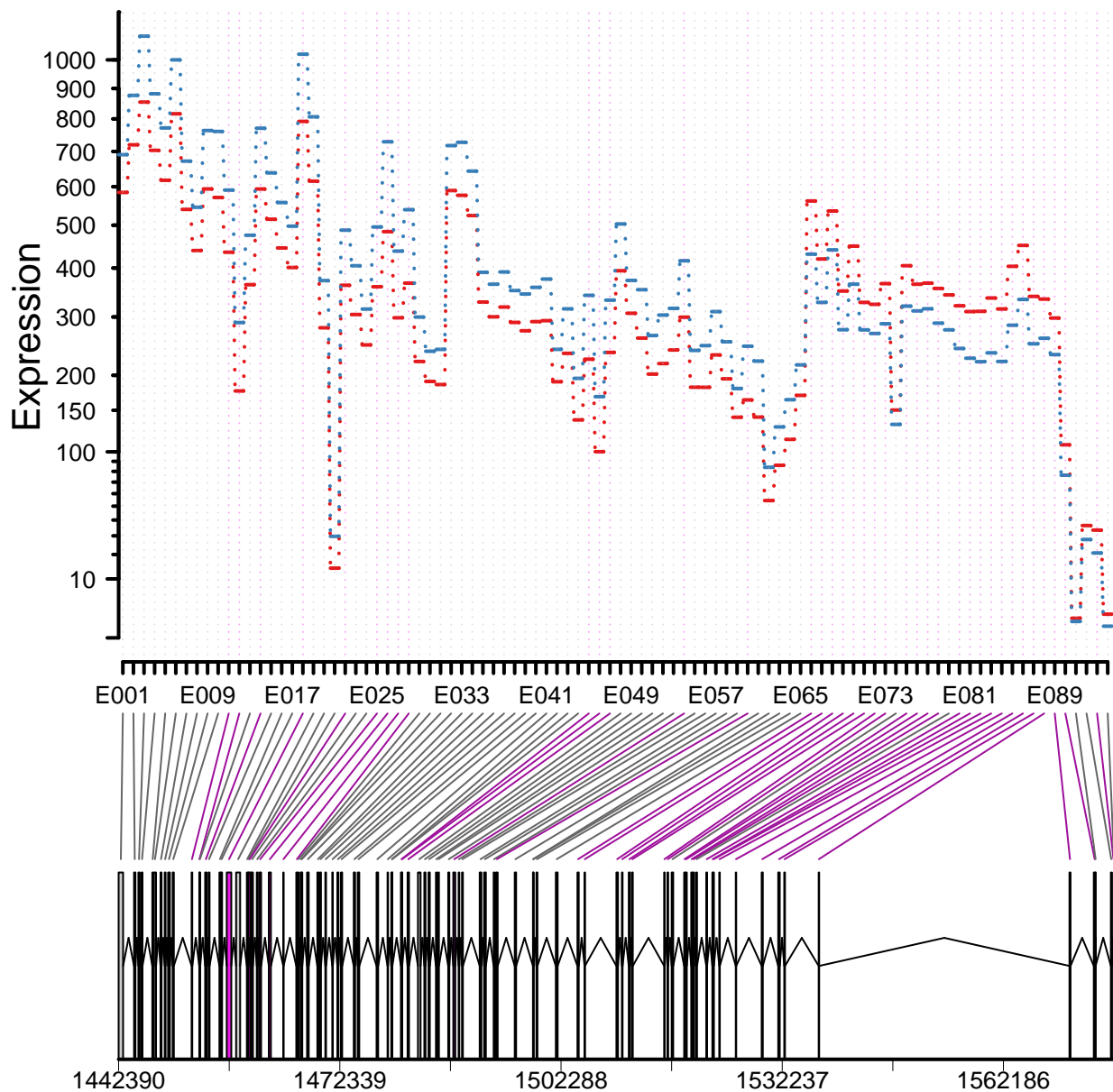

Supplement: Supplementary file 11 [file Data_Sheet_1.ZIP › Supplementary 17/gene-Huwe1.pdf]

gene-Ints10 +

C

S

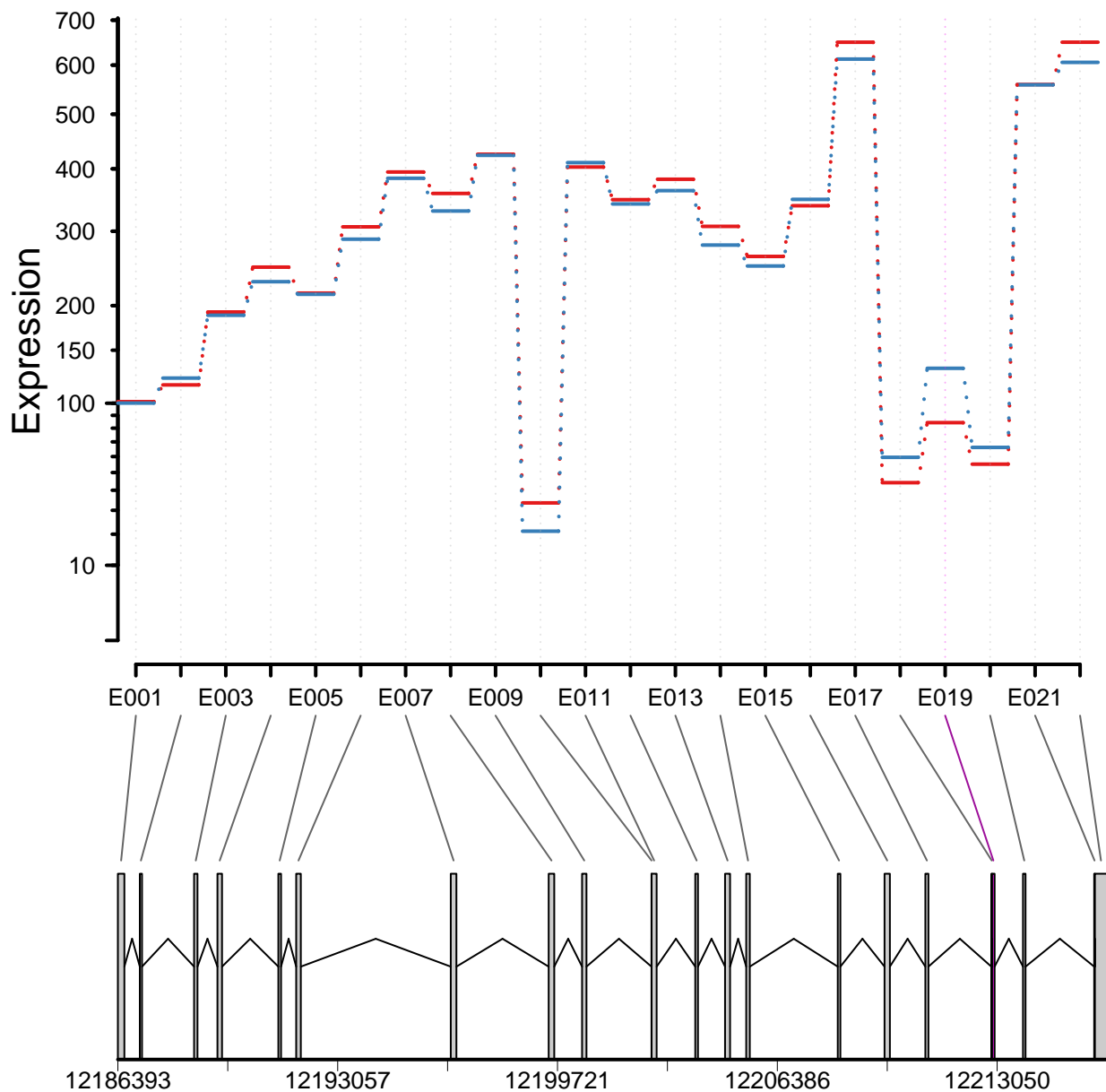

Supplement: Supplementary file 11 [file Data_Sheet_1.ZIP › Supplementary 17/gene-Ints10.pdf]

gene-Itgb6 -

C

S

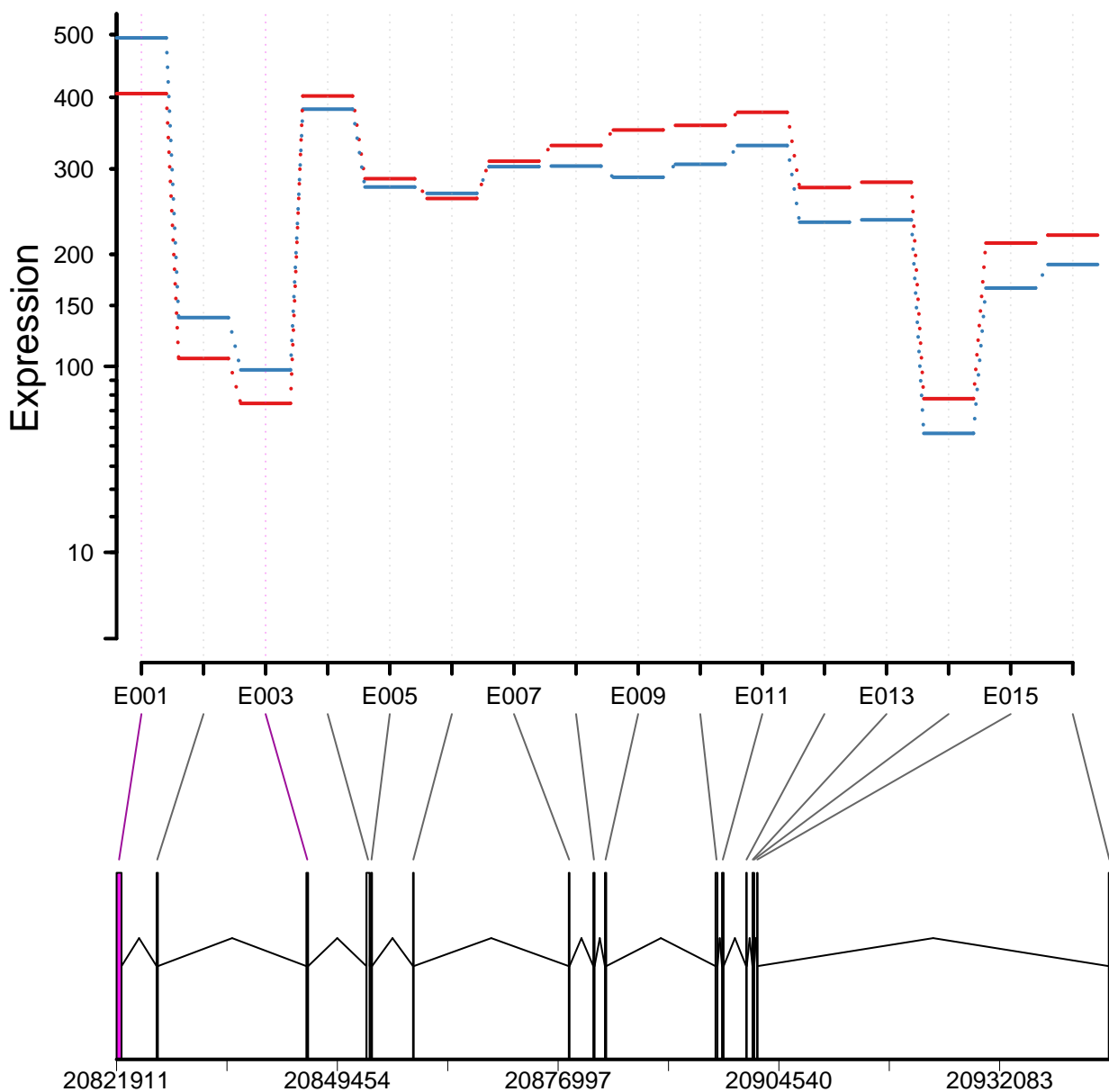

Supplement: Supplementary file 11 [file Data_Sheet_1.ZIP › Supplementary 17/gene-Itgb6.pdf]

gene-lvns1abp +

C

S

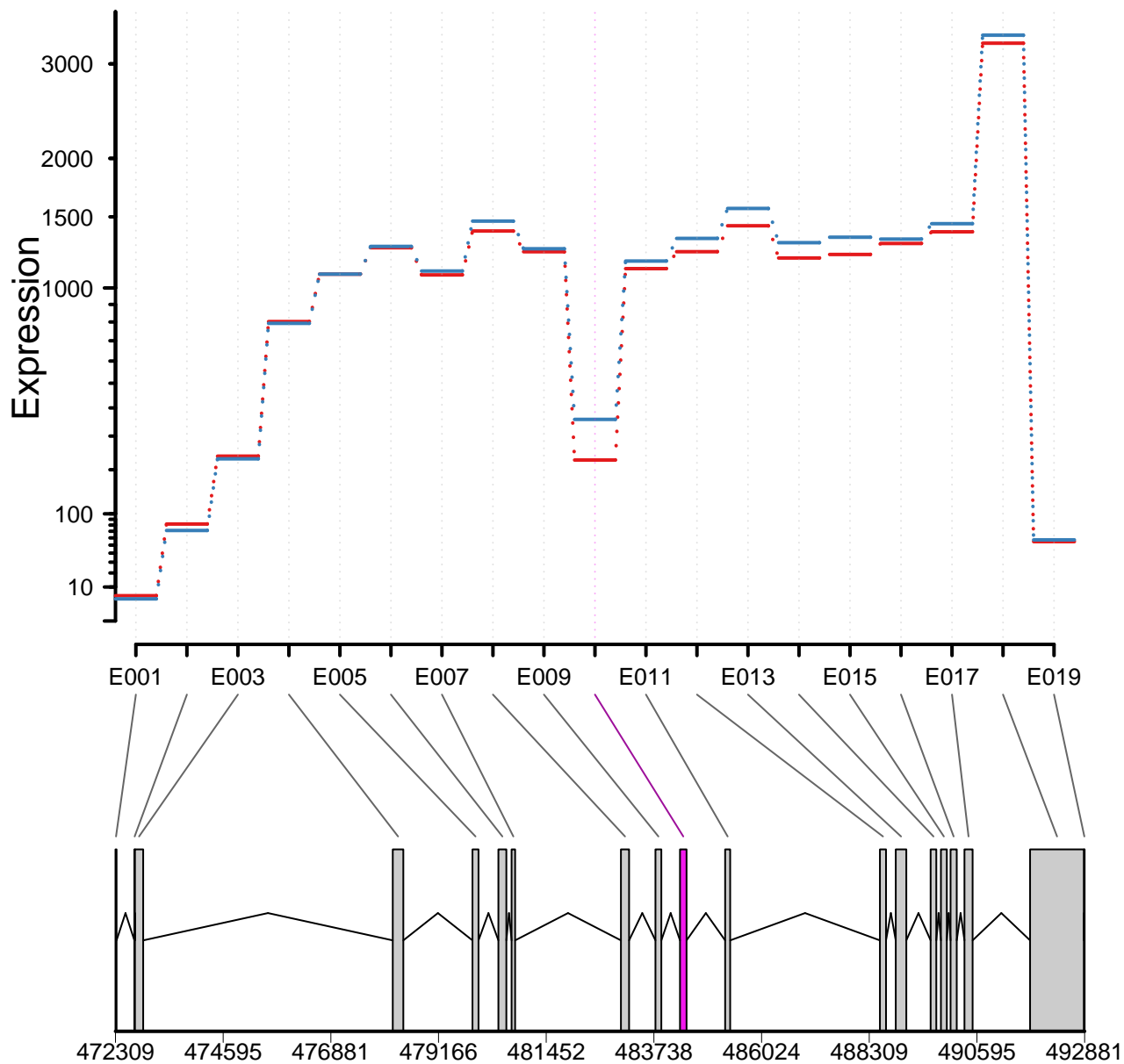

Supplement: Supplementary file 11 [file Data_Sheet_1.ZIP › Supplementary 17/gene-Ivns1abp.pdf]

gene-Jagn1 -

C

S

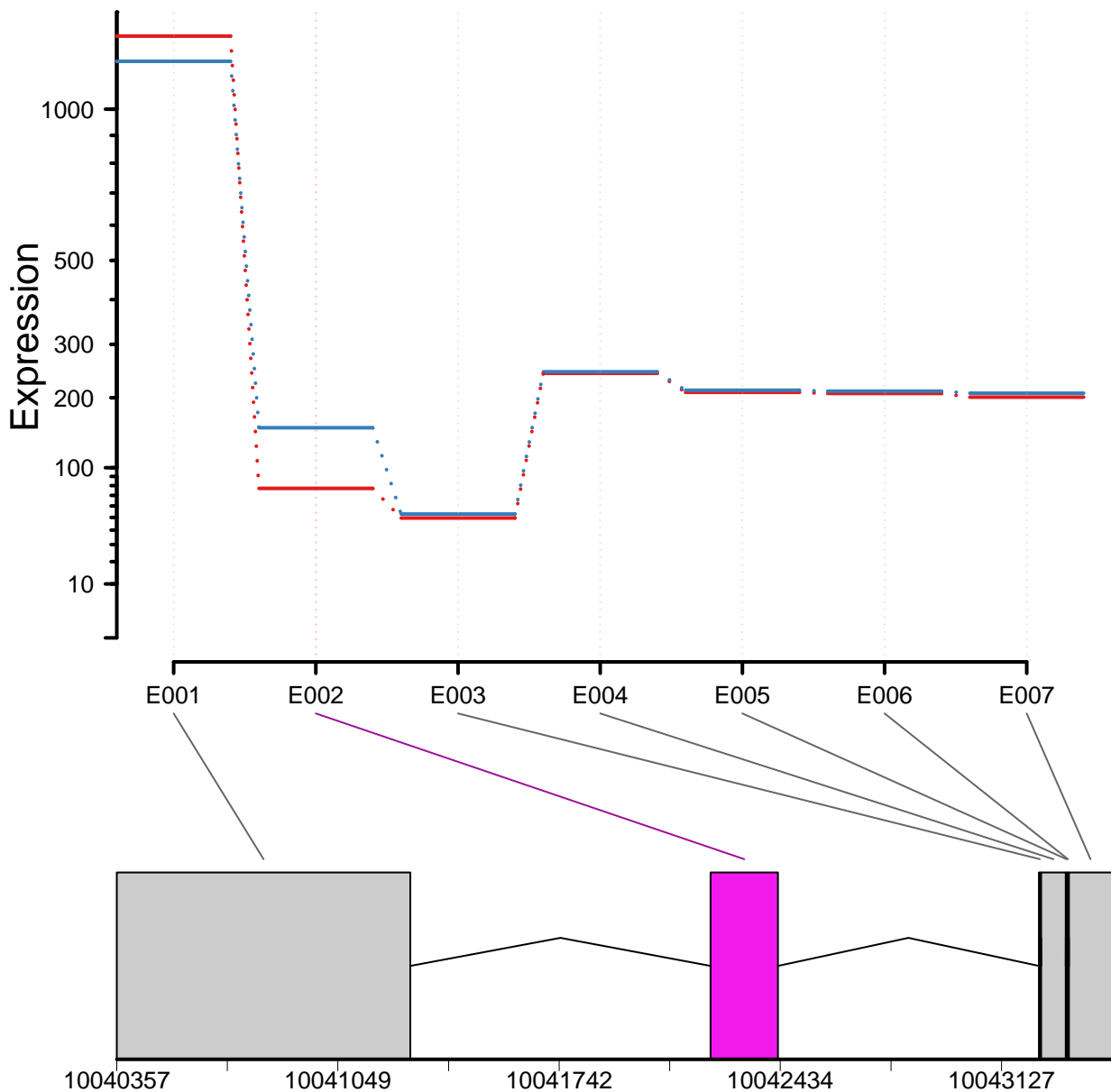

Supplement: Supplementary file 11 [file Data_Sheet_1.ZIP › Supplementary 17/gene-Jagn1.pdf]

gene-Kansl2 -

C

S

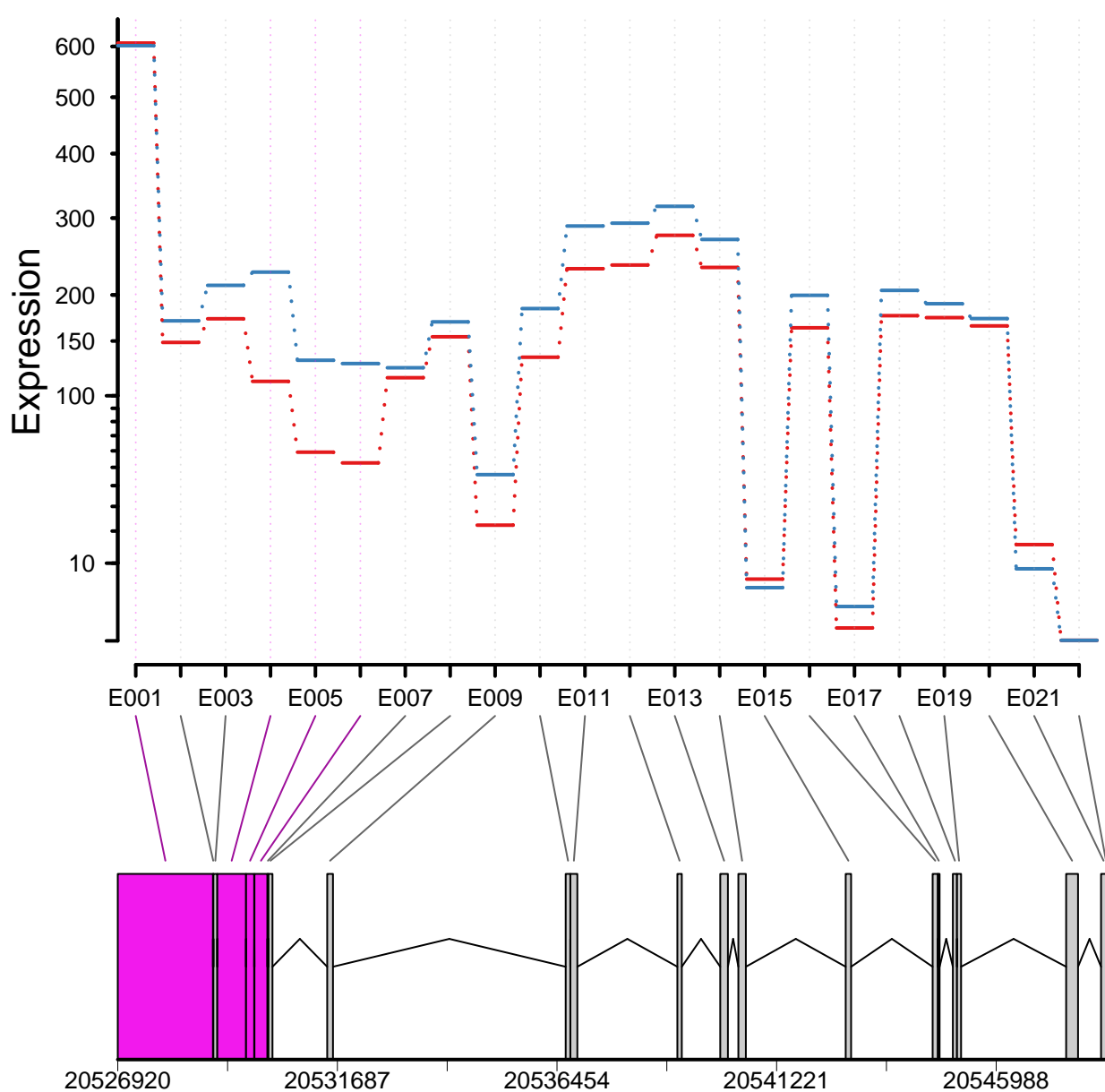

Supplement: Supplementary file 11 [file Data_Sheet_1.ZIP › Supplementary 17/gene-Kansl2.pdf]

gene-Kif23 +

C

S

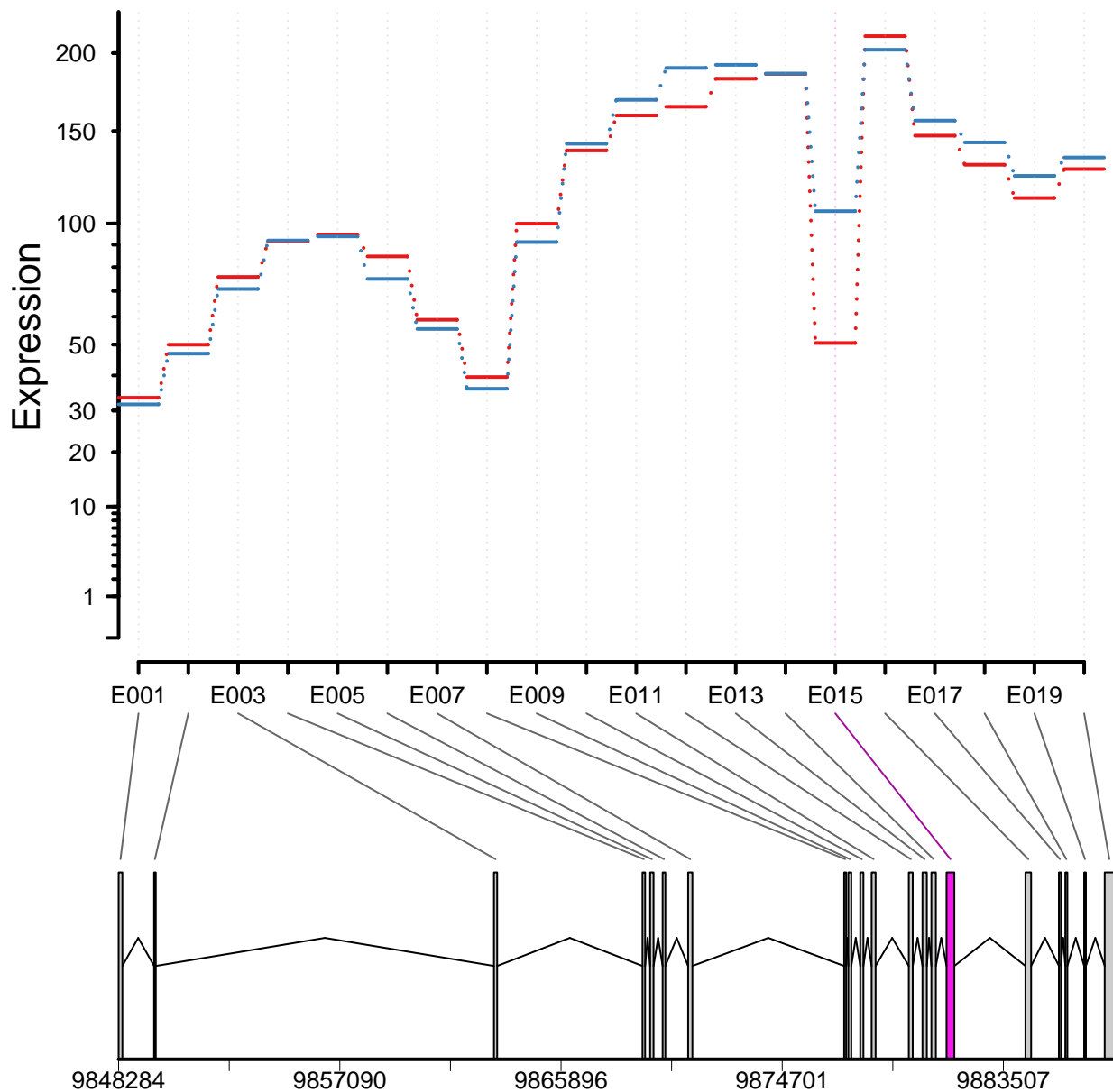

Supplement: Supplementary file 11 [file Data_Sheet_1.ZIP › Supplementary 17/gene-Kif23.pdf]

gene-LOC101823746 +

C

S

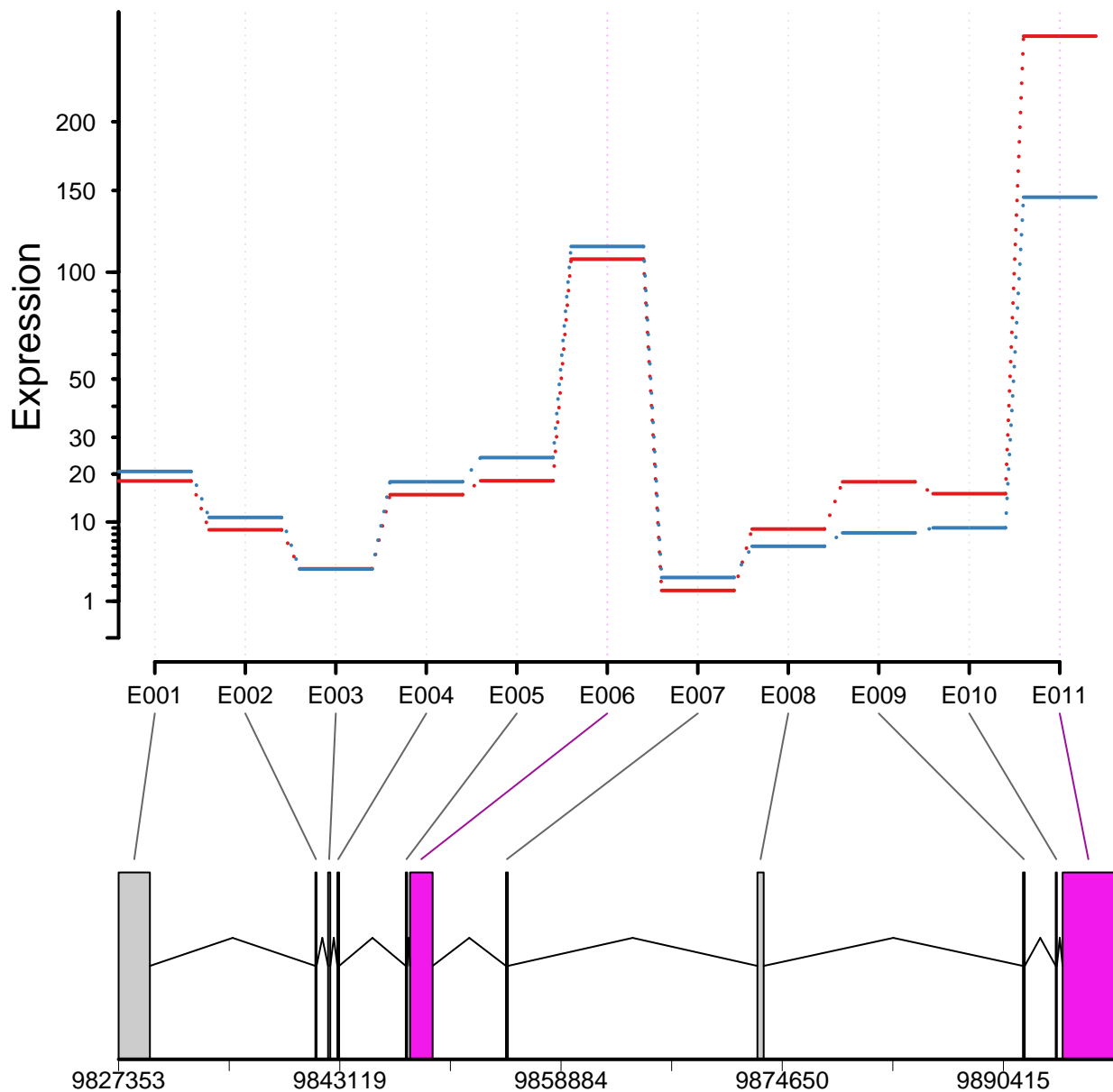

Supplement: Supplementary file 11 [file Data_Sheet_1.ZIP › Supplementary 17/gene-LOC101823746.pdf]

gene-LOC101834096 +

C

S

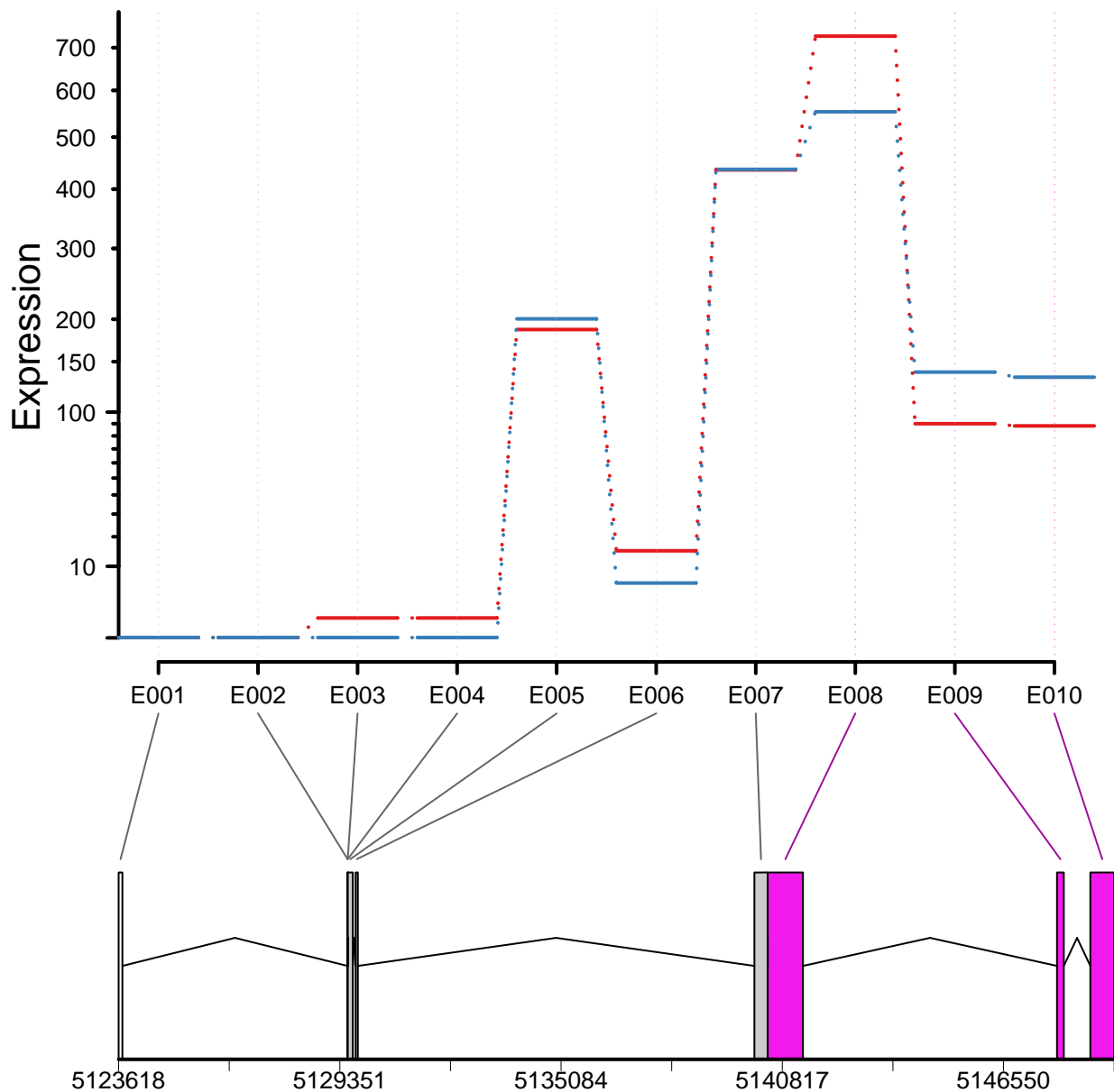

Supplement: Supplementary file 11 [file Data_Sheet_1.ZIP › Supplementary 17/gene-LOC101834096.pdf]

gene-LOC101834606 -

C

S

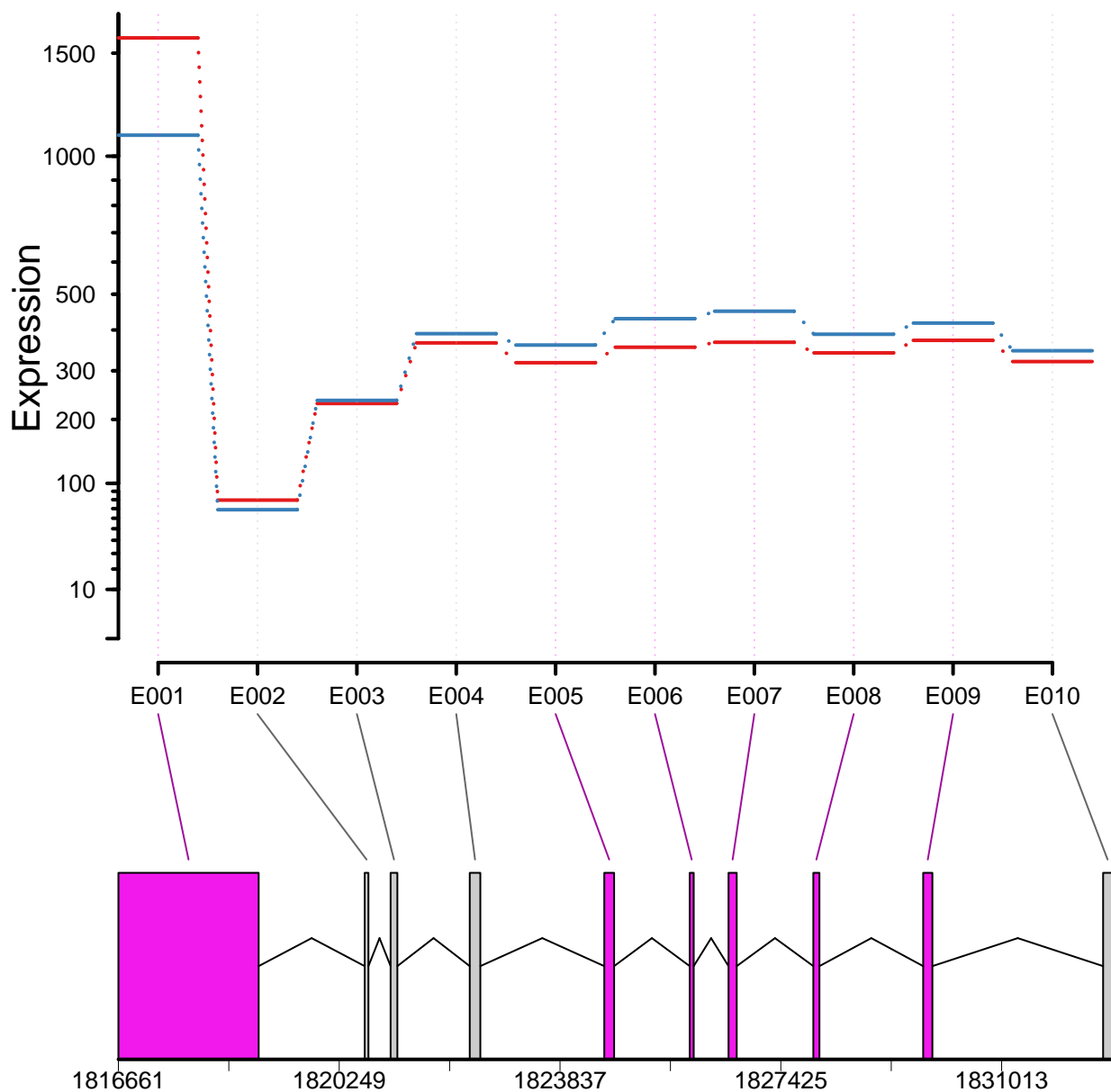

Supplement: Supplementary file 11 [file Data_Sheet_1.ZIP › Supplementary 17/gene-LOC101834606.pdf]

gene-LOC101843260 +

C

S

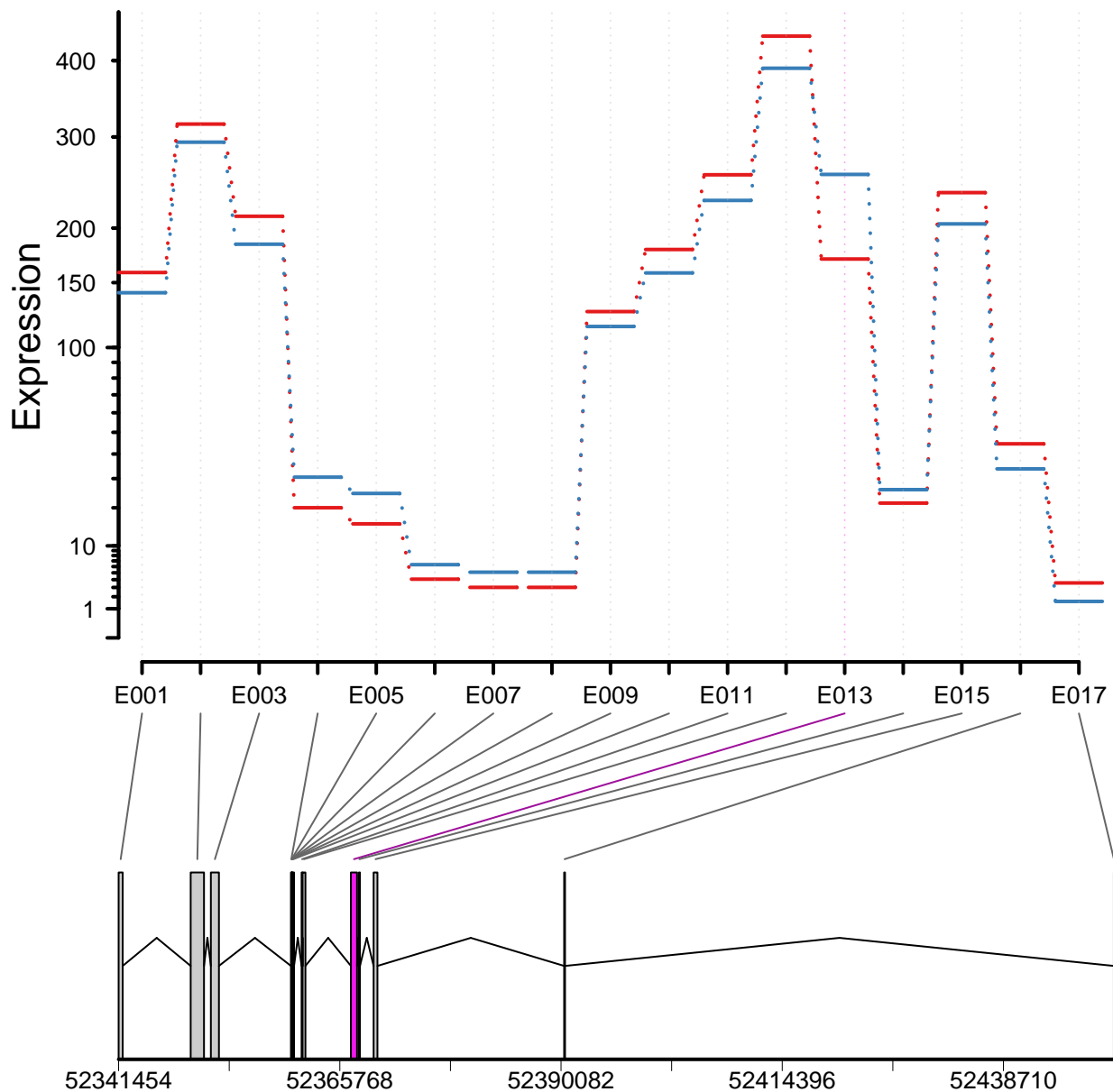

Supplement: Supplementary file 11 [file Data_Sheet_1.ZIP › Supplementary 17/gene-LOC101843260.pdf]

gene-LOC106021065+gene-LOC101832181 – S

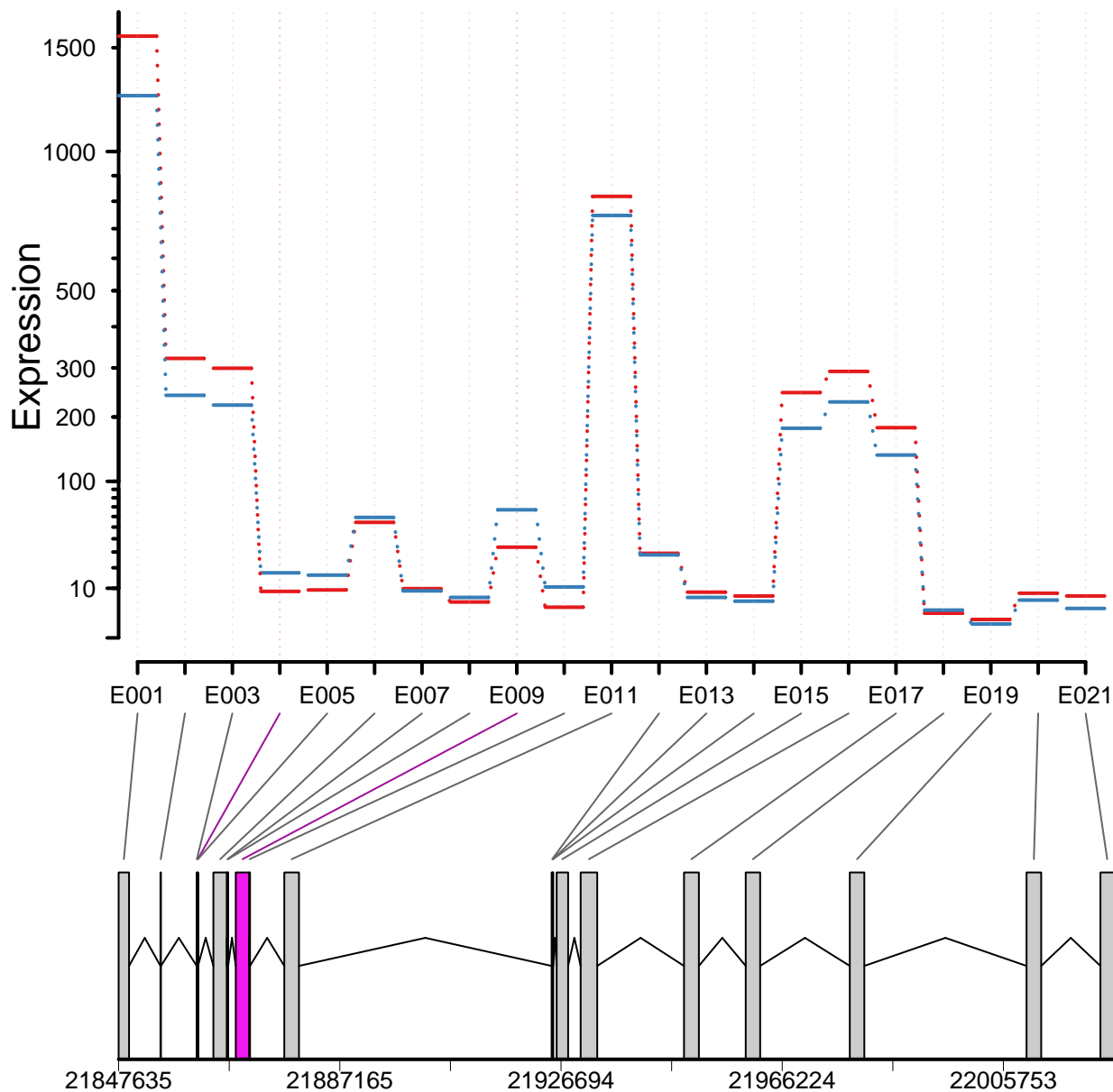

Supplement: Supplementary file 11 [file Data_Sheet_1.ZIP › Supplementary 17/gene-LOC106021065+gene-LOC101832181.pdf]

# gene-LOC106021468+gene-Cdkl5 C S

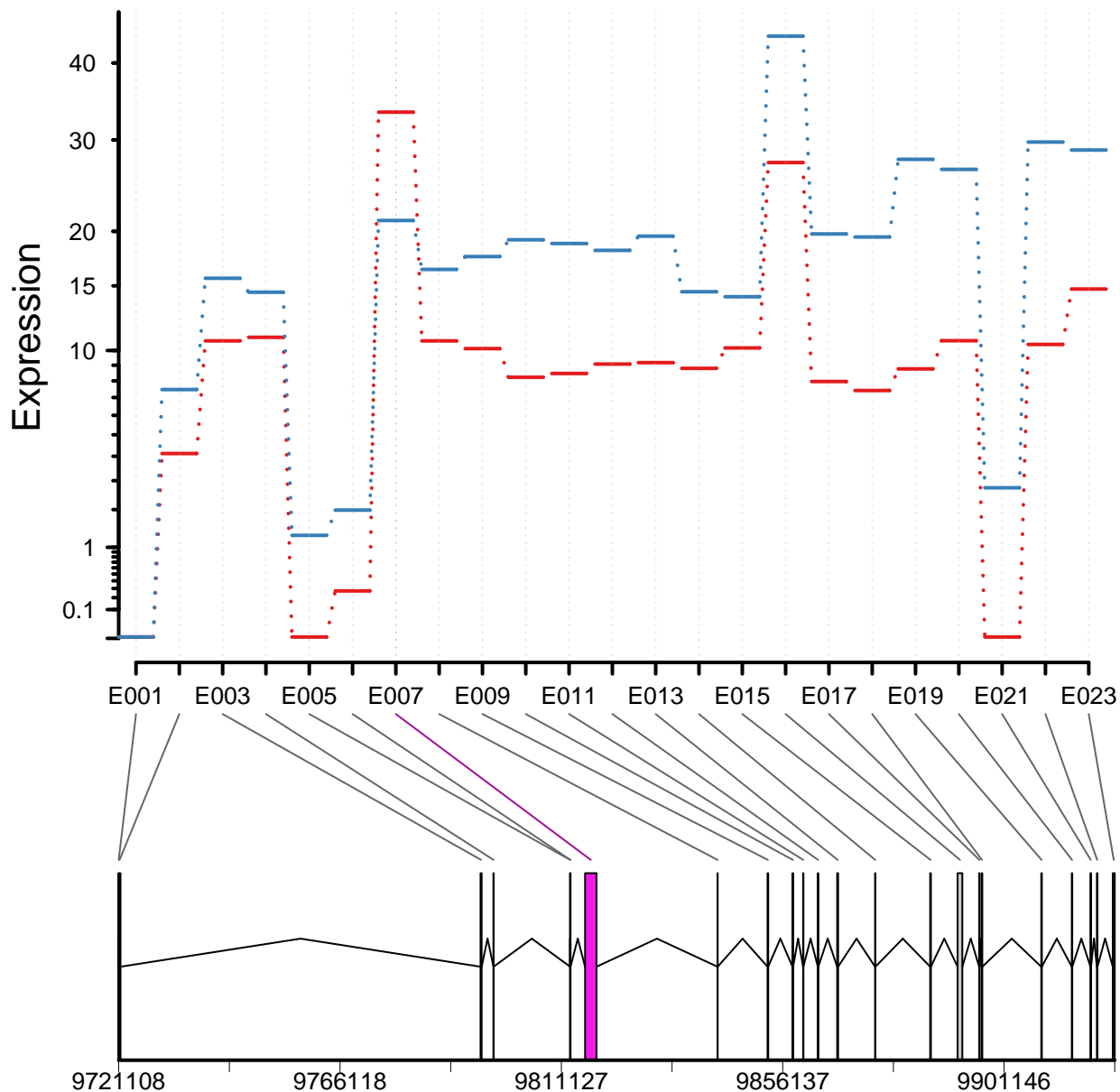

Supplement: Supplementary file 11 [file Data_Sheet_1.ZIP › Supplementary 17/gene-LOC106021468+gene-Cdkl5.pdf]

gene-LOC106022533 -

C

S

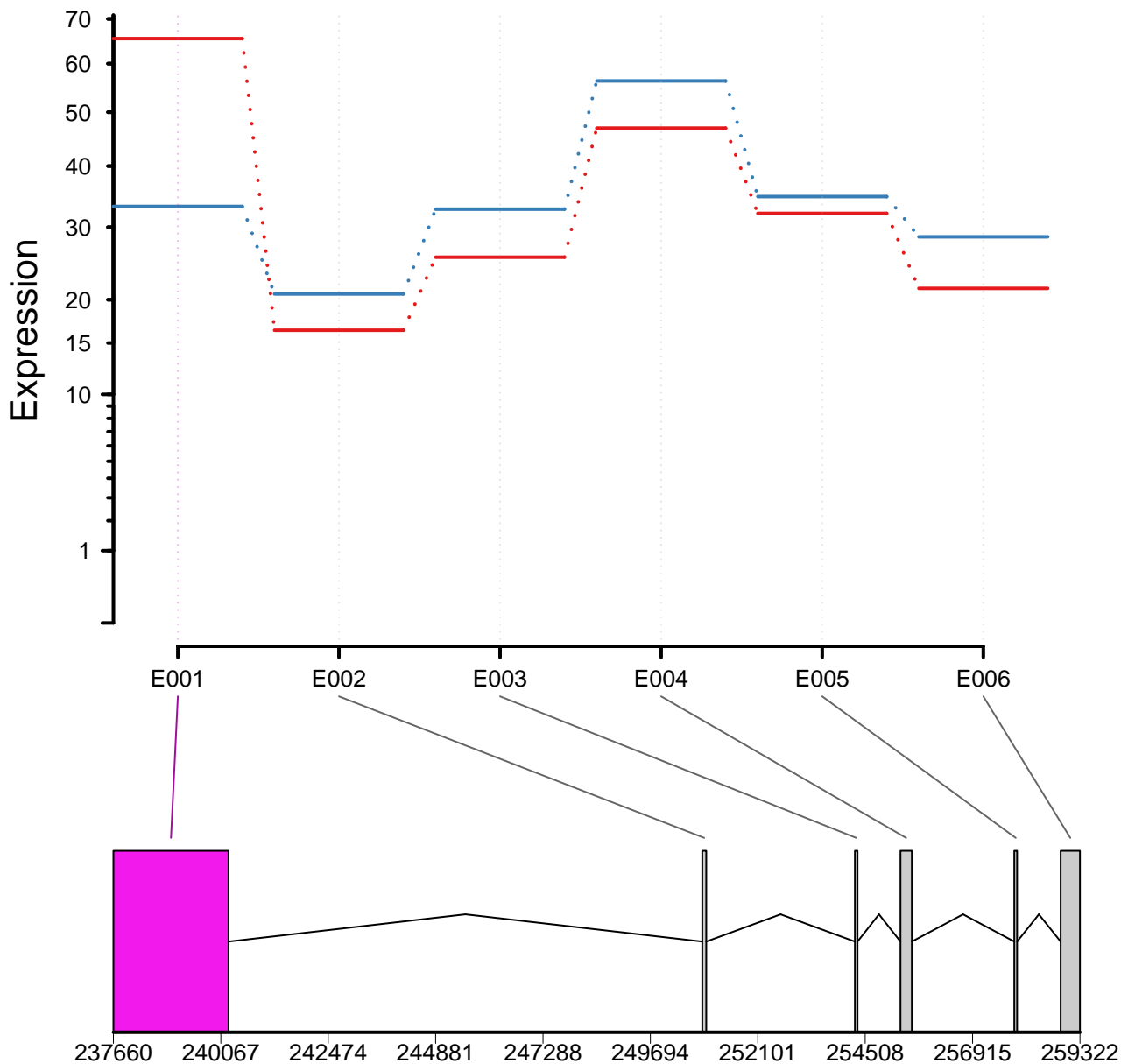

Supplement: Supplementary file 11 [file Data_Sheet_1.ZIP › Supplementary 17/gene-LOC106022533.pdf]

gene-LOC106022537 -

C

S

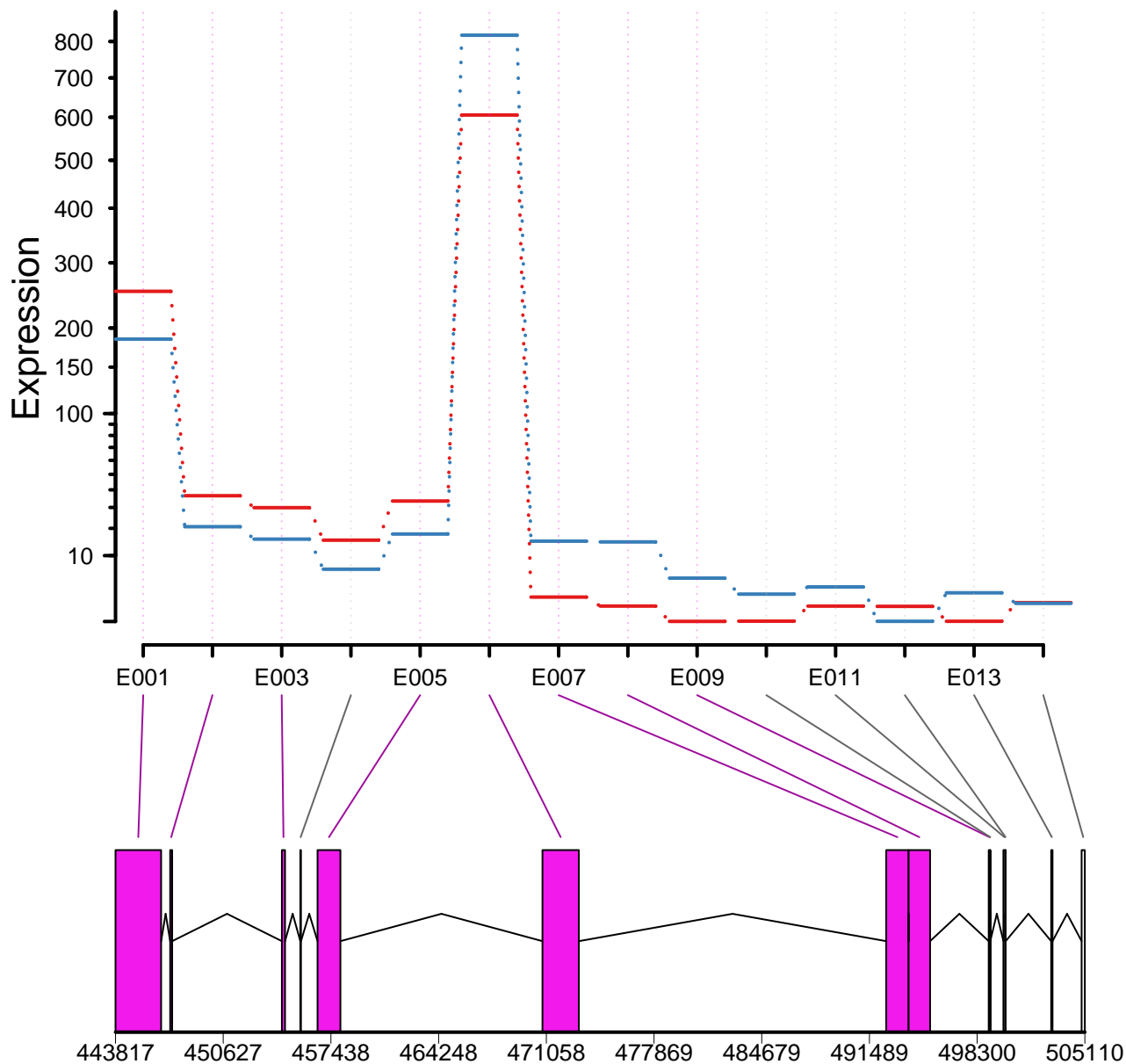

Supplement: Supplementary file 11 [file Data_Sheet_1.ZIP › Supplementary 17/gene-LOC106022537.pdf]

gene-LOC110340397 -

C

S

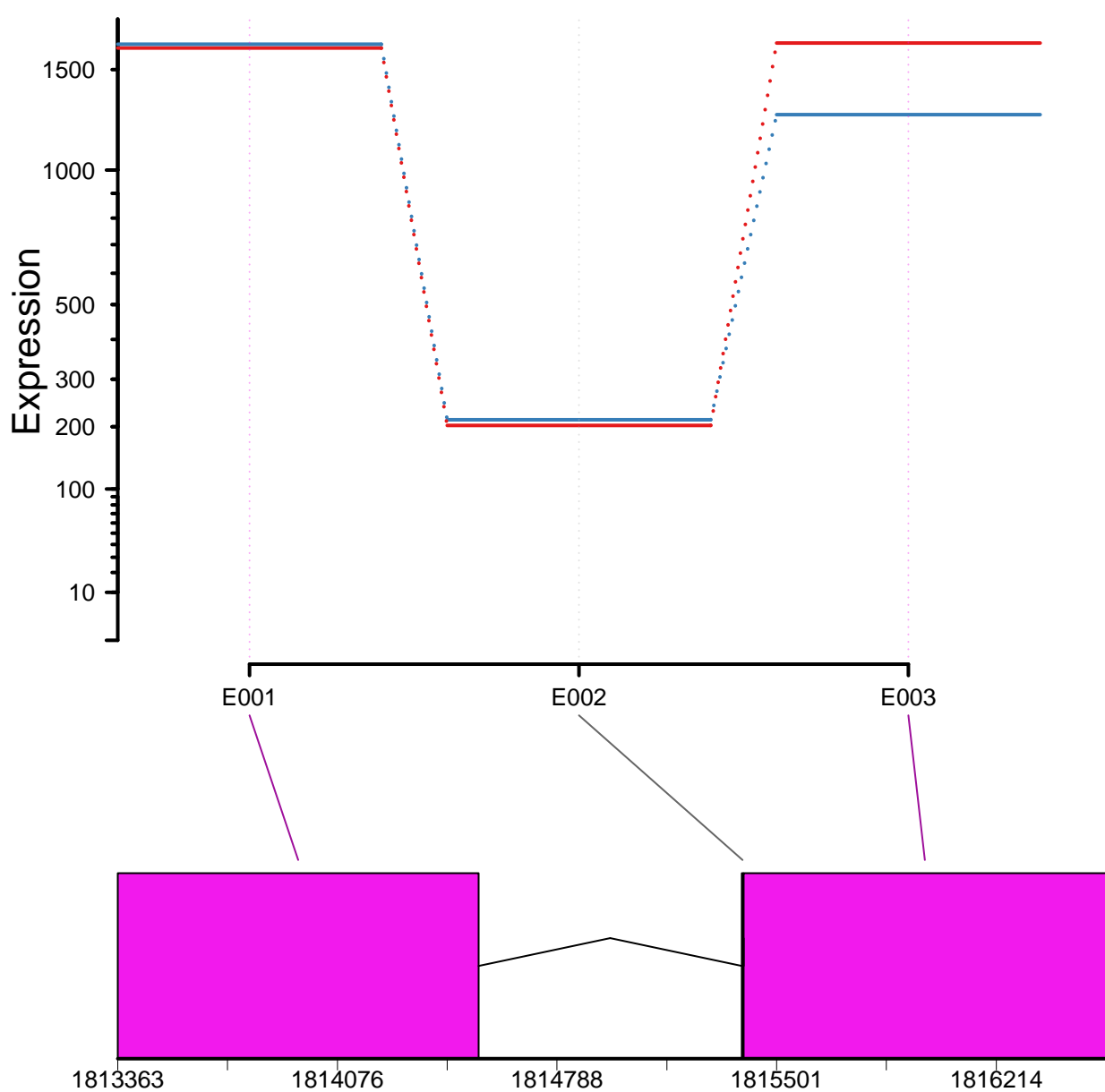

Supplement: Supplementary file 11 [file Data_Sheet_1.ZIP › Supplementary 17/gene-LOC110340397.pdf]

gene-Leng8 -

C

S

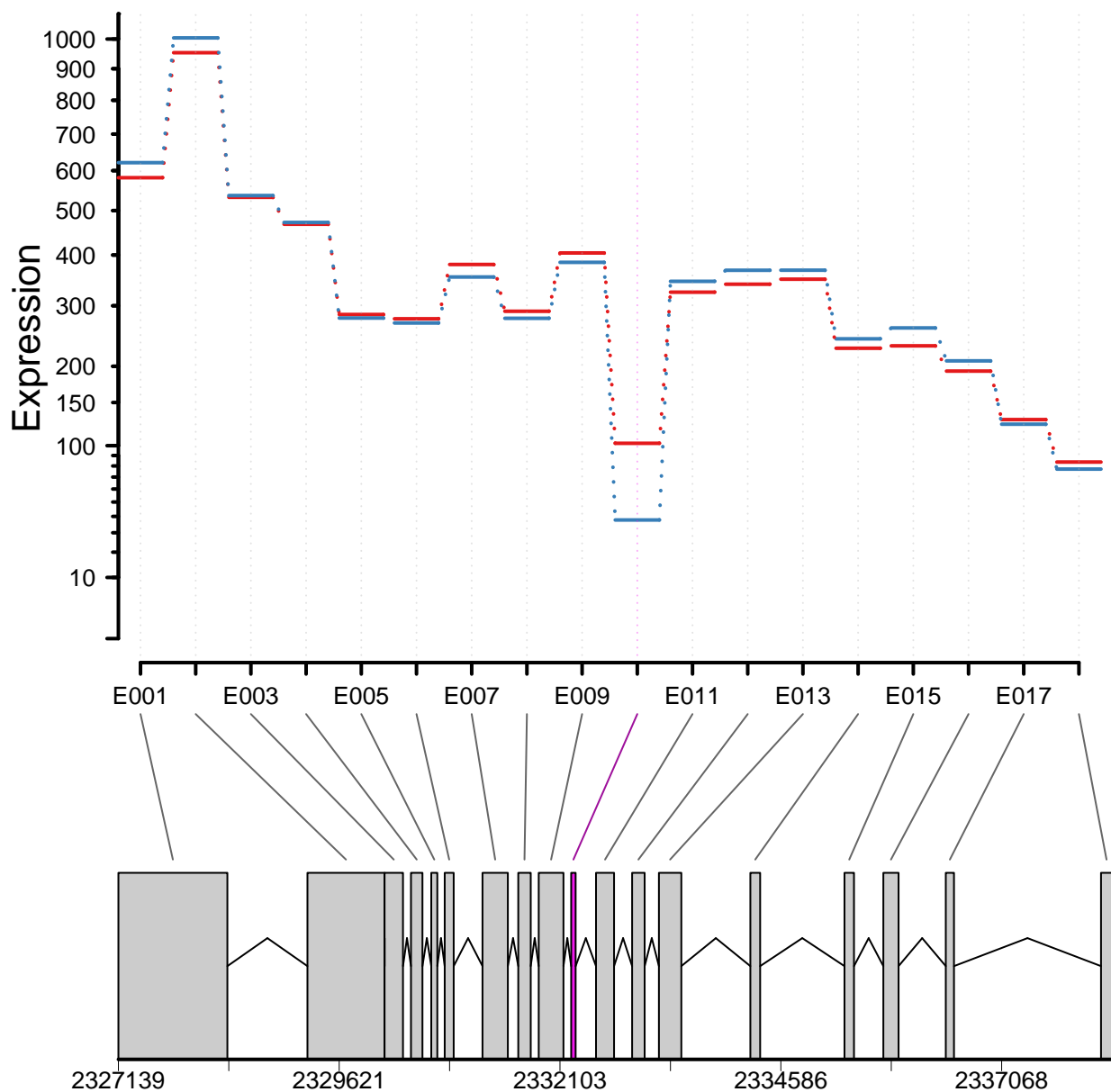

Supplement: Supplementary file 11 [file Data_Sheet_1.ZIP › Supplementary 17/gene-Leng8.pdf]

gene-Lrrfip1 -

C

S

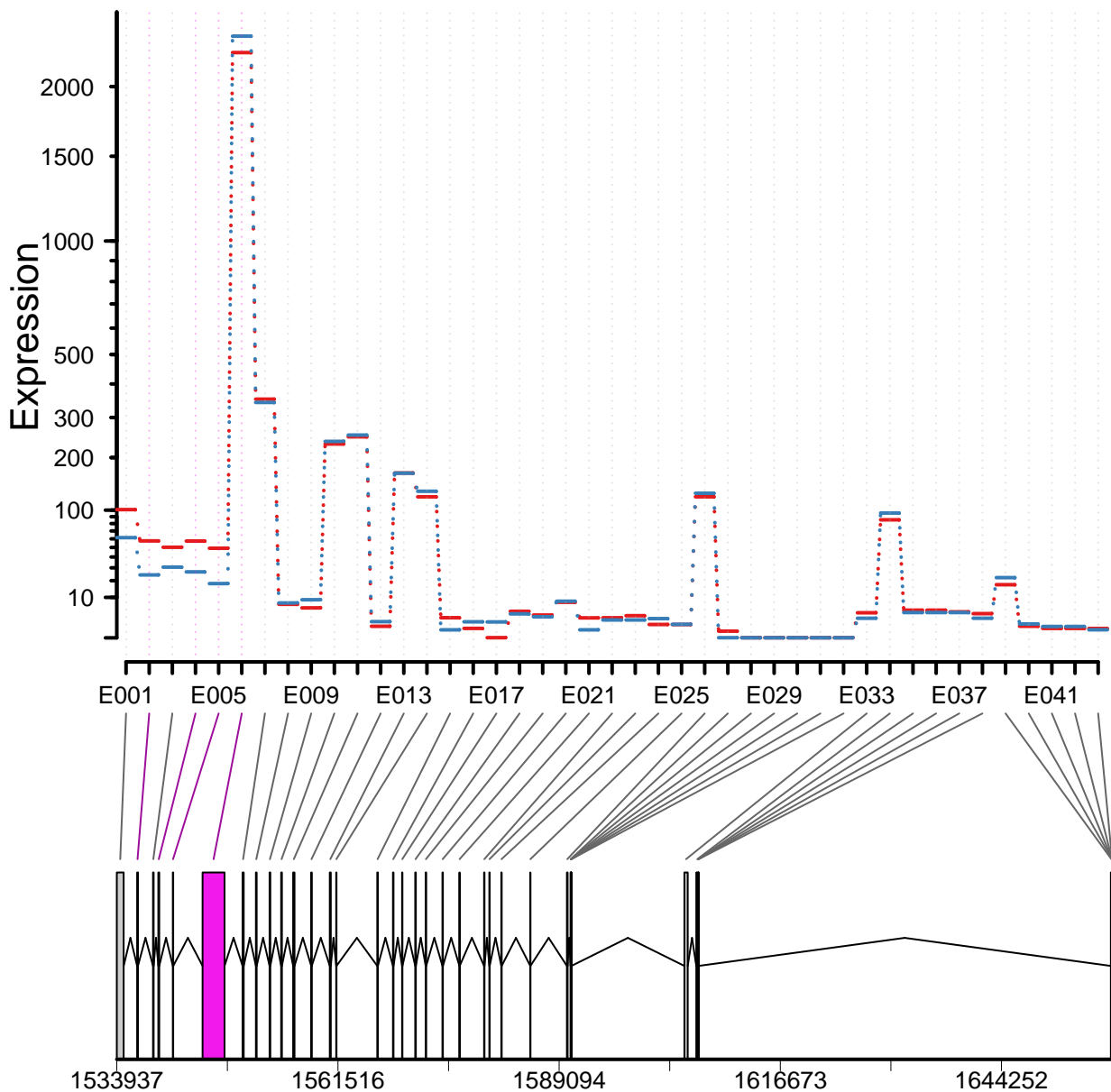

Supplement: Supplementary file 11 [file Data_Sheet_1.ZIP › Supplementary 17/gene-Lrrfip1.pdf]

gene-Mapk9 -

C

S

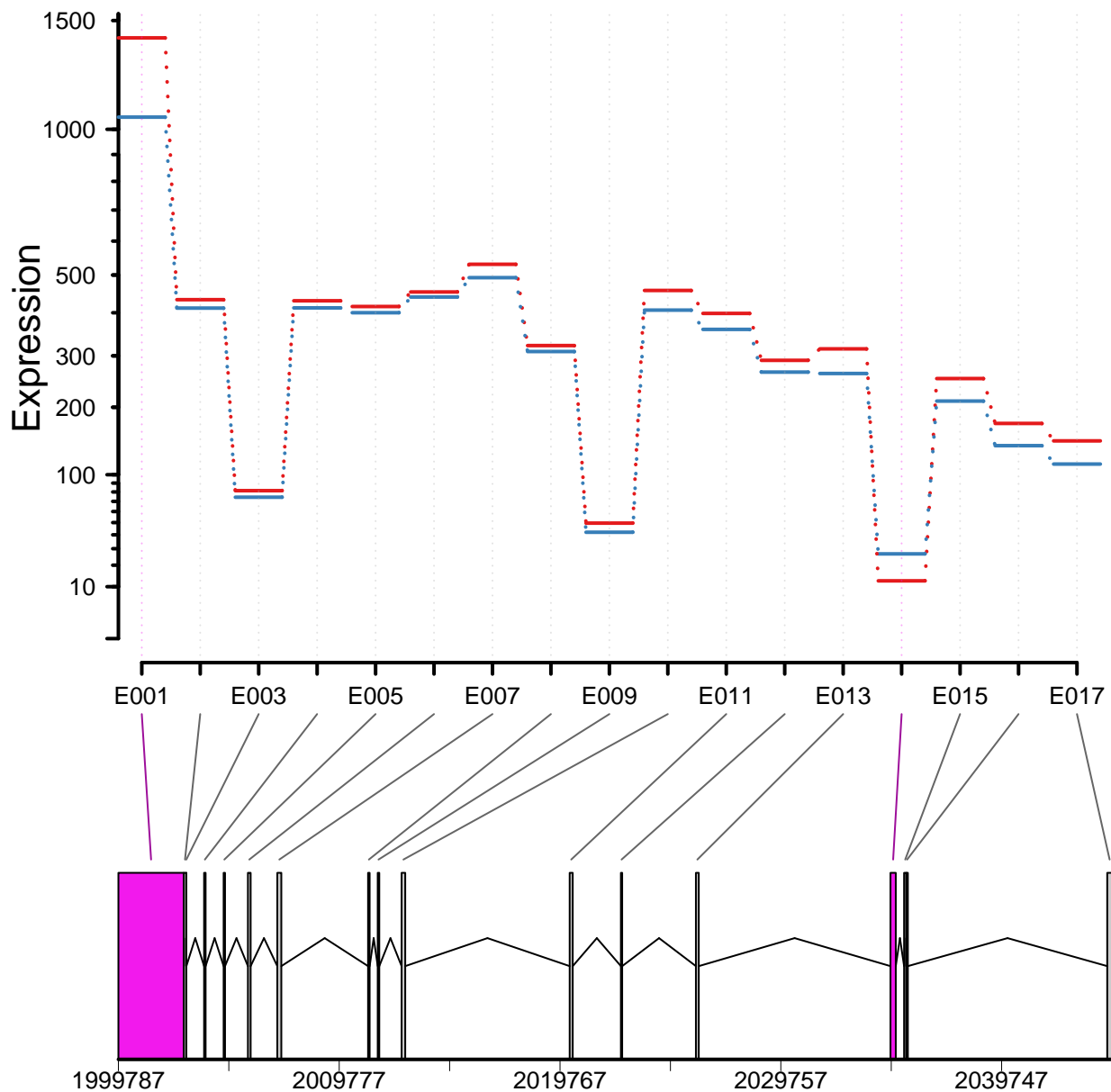

Supplement: Supplementary file 11 [file Data_Sheet_1.ZIP › Supplementary 17/gene-Mapk9.pdf]

gene-Mkln1 +

C

S

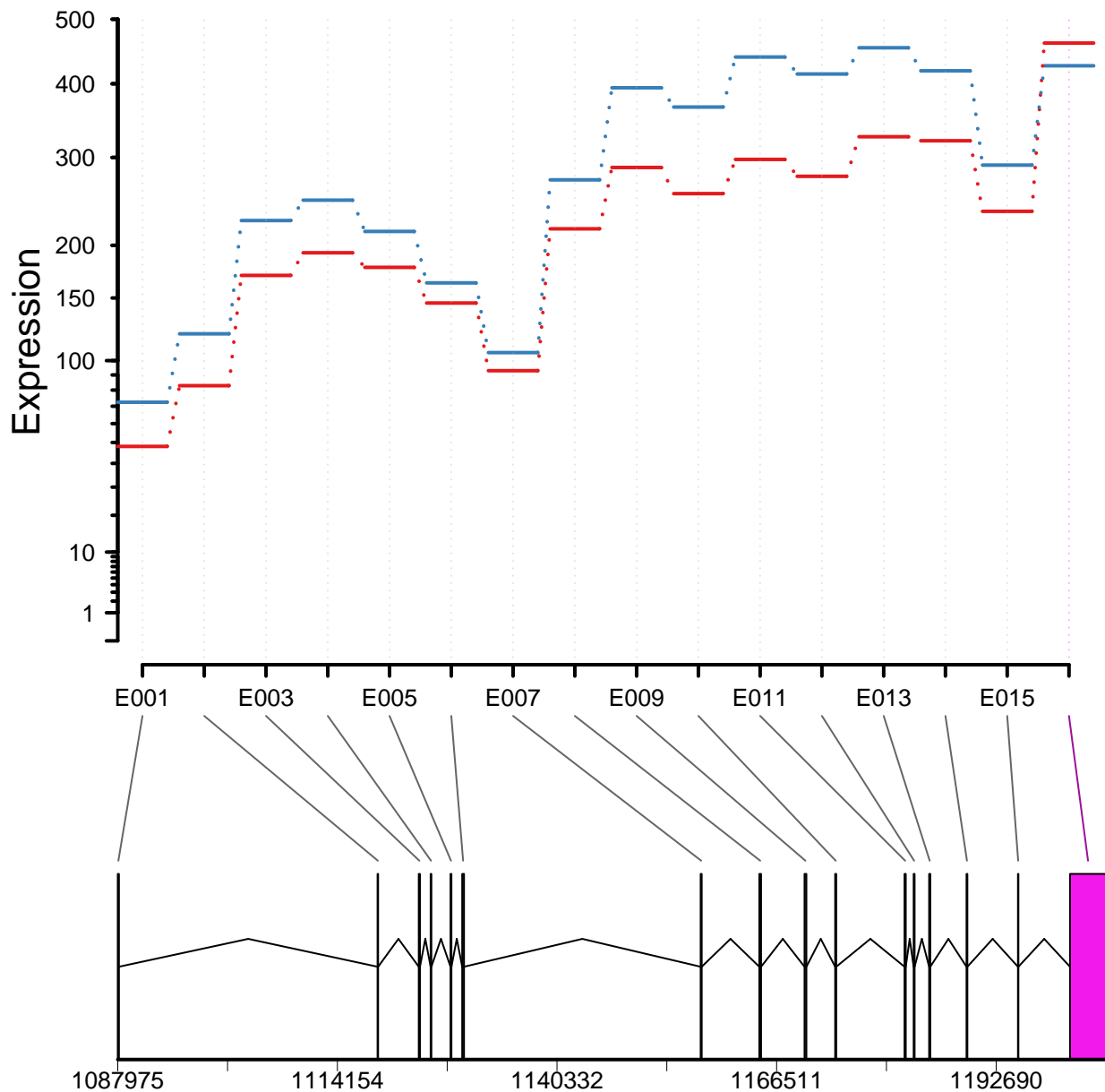

Supplement: Supplementary file 11 [file Data_Sheet_1.ZIP › Supplementary 17/gene-Mkln1.pdf]

gene-Mprip -

C

S

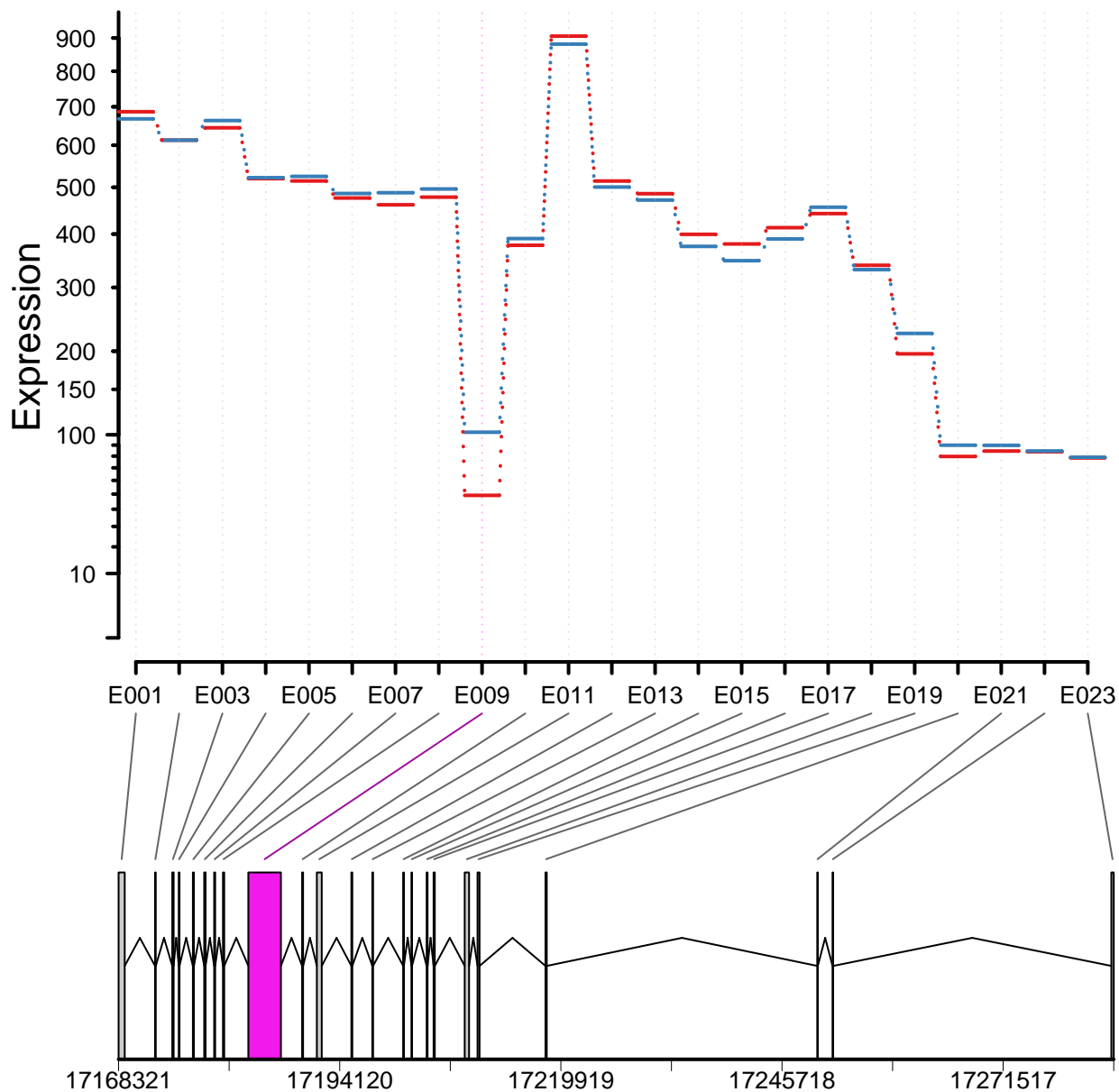

Supplement: Supplementary file 11 [file Data_Sheet_1.ZIP › Supplementary 17/gene-Mprip.pdf]

gene-Mrps30 -

C

S

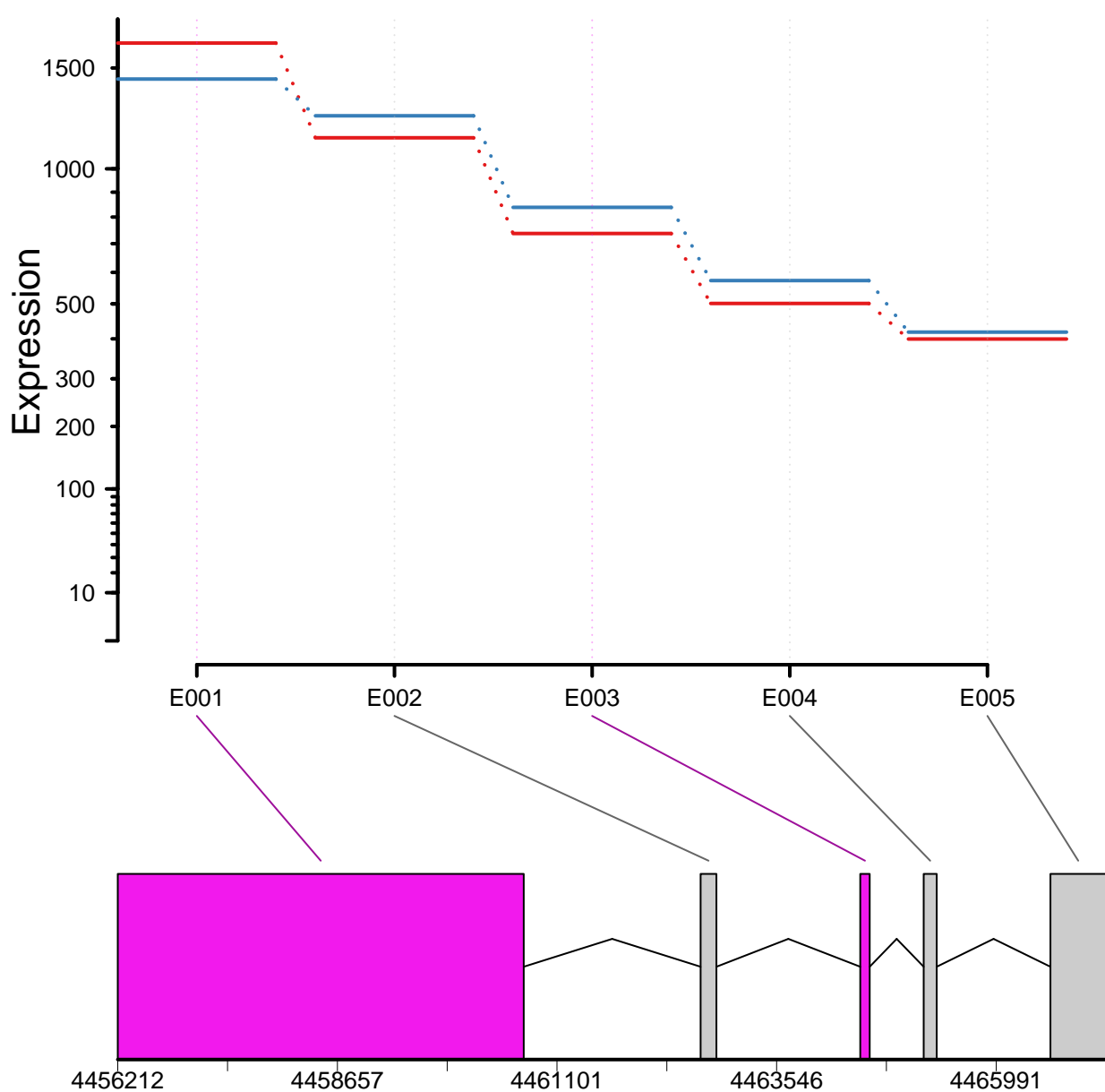

Supplement: Supplementary file 11 [file Data_Sheet_1.ZIP › Supplementary 17/gene-Mrps30.pdf]

gene-Myo5a -

C

S

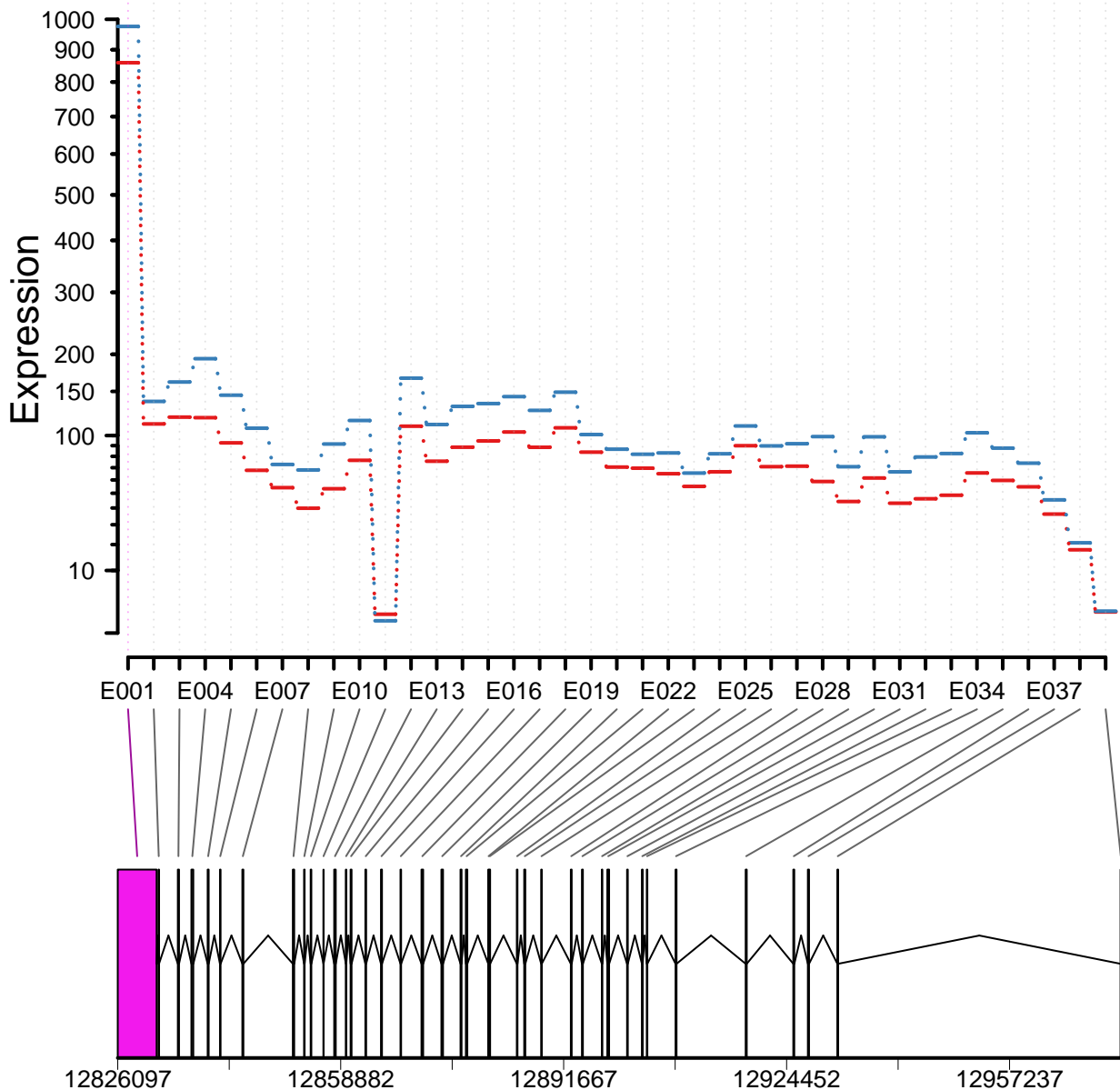

Supplement: Supplementary file 11 [file Data_Sheet_1.ZIP › Supplementary 17/gene-Myo5a.pdf]

gene-Ncoa3 -

C

S

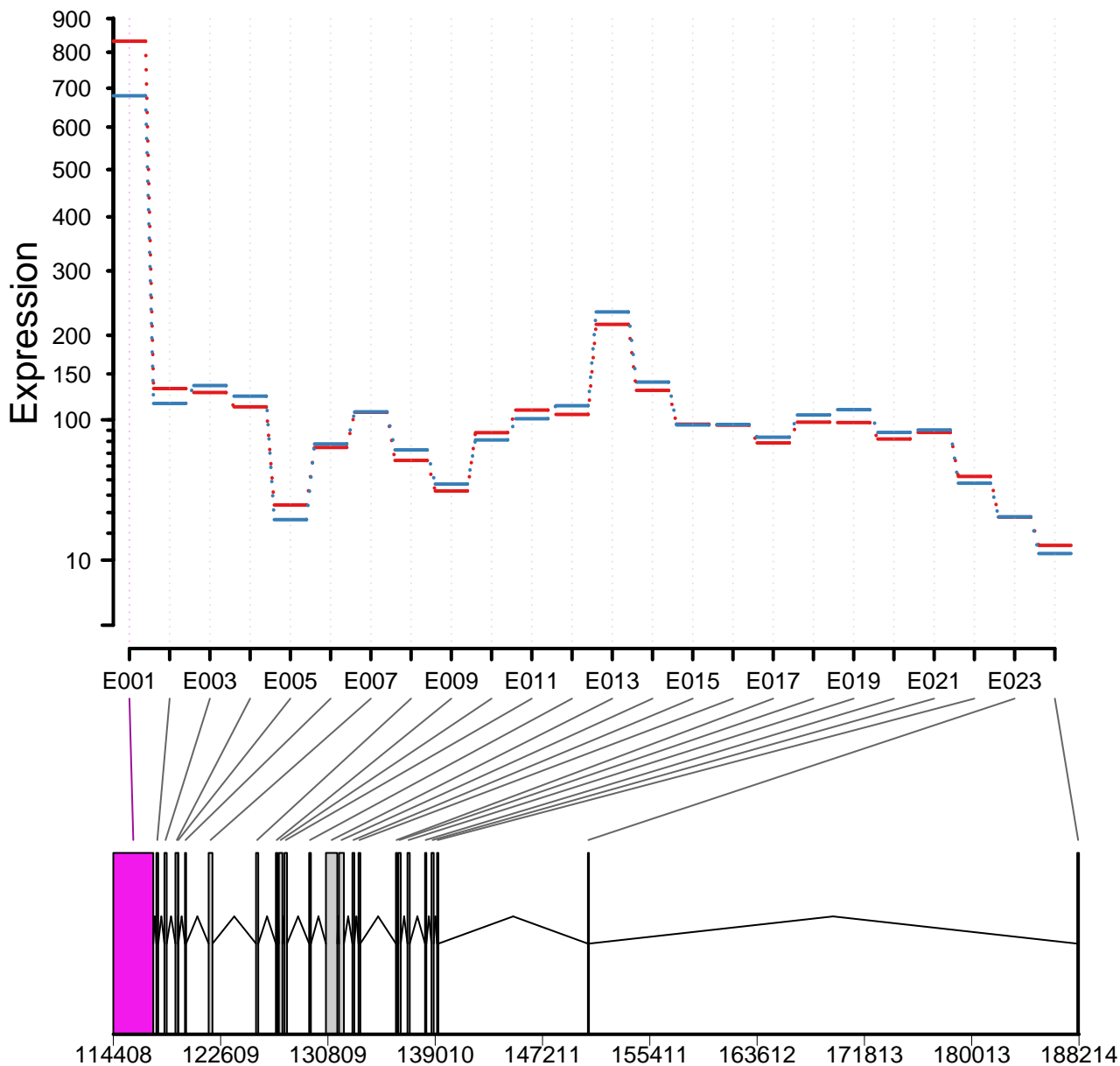

Supplement: Supplementary file 11 [file Data_Sheet_1.ZIP › Supplementary 17/gene-Ncoa3.pdf]

gene-Ndc1 –

C

S

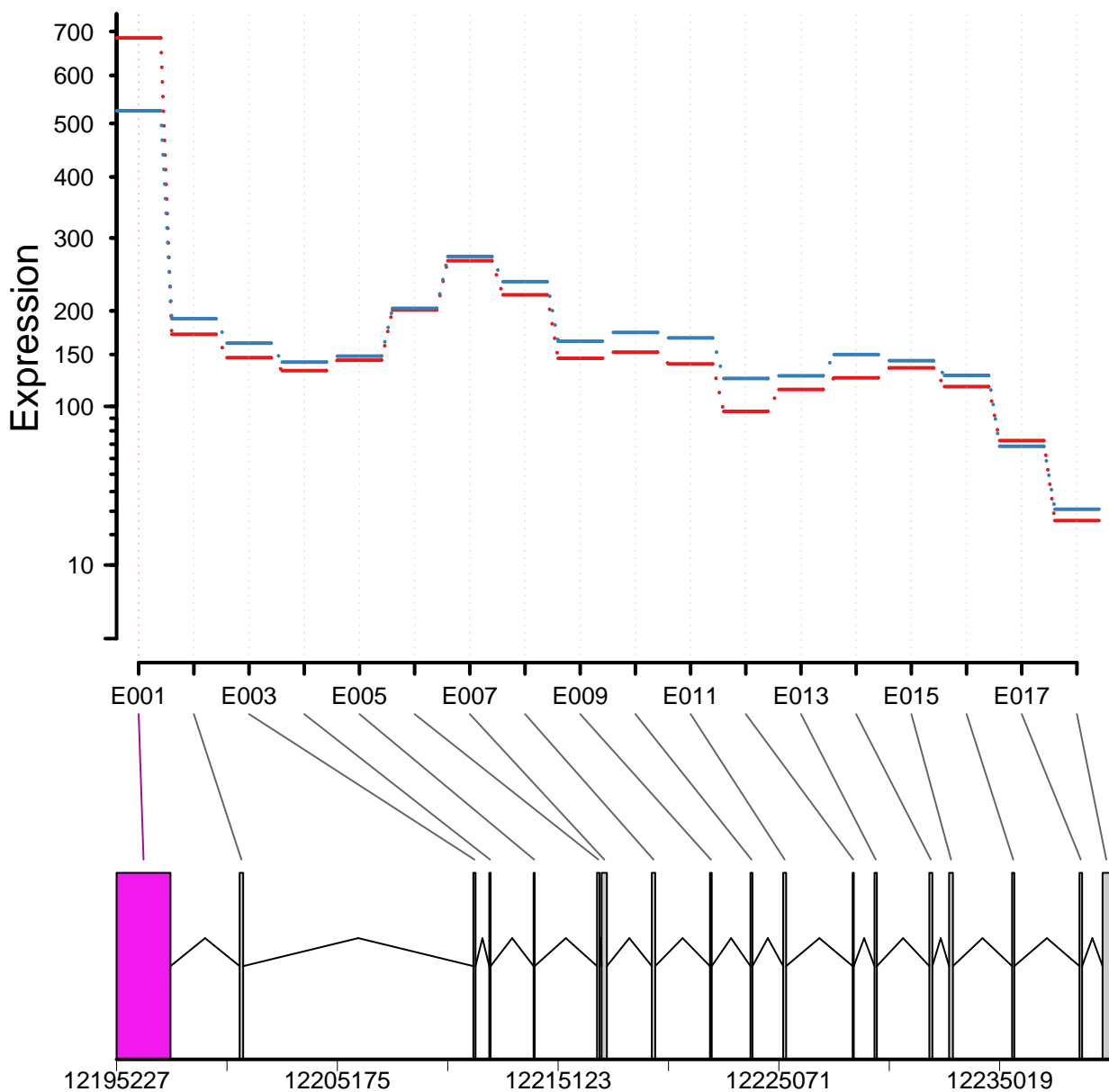

Supplement: Supplementary file 11 [file Data_Sheet_1.ZIP › Supplementary 17/gene-Ndc1.pdf]

gene-Nfkbiz -

C

S

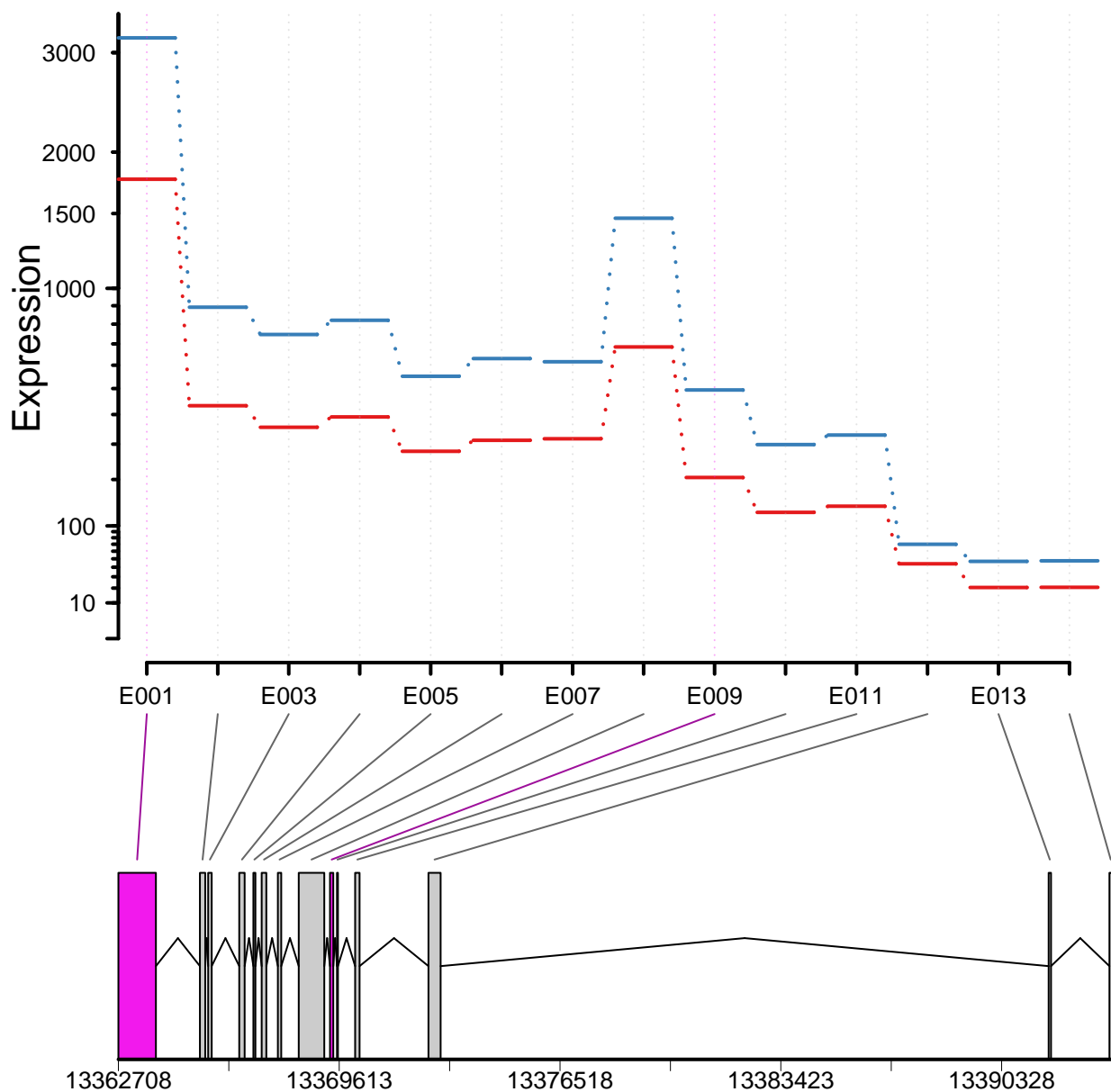

Supplement: Supplementary file 11 [file Data_Sheet_1.ZIP › Supplementary 17/gene-Nfkbiz.pdf]

gene-Nr2f2 +

C

S

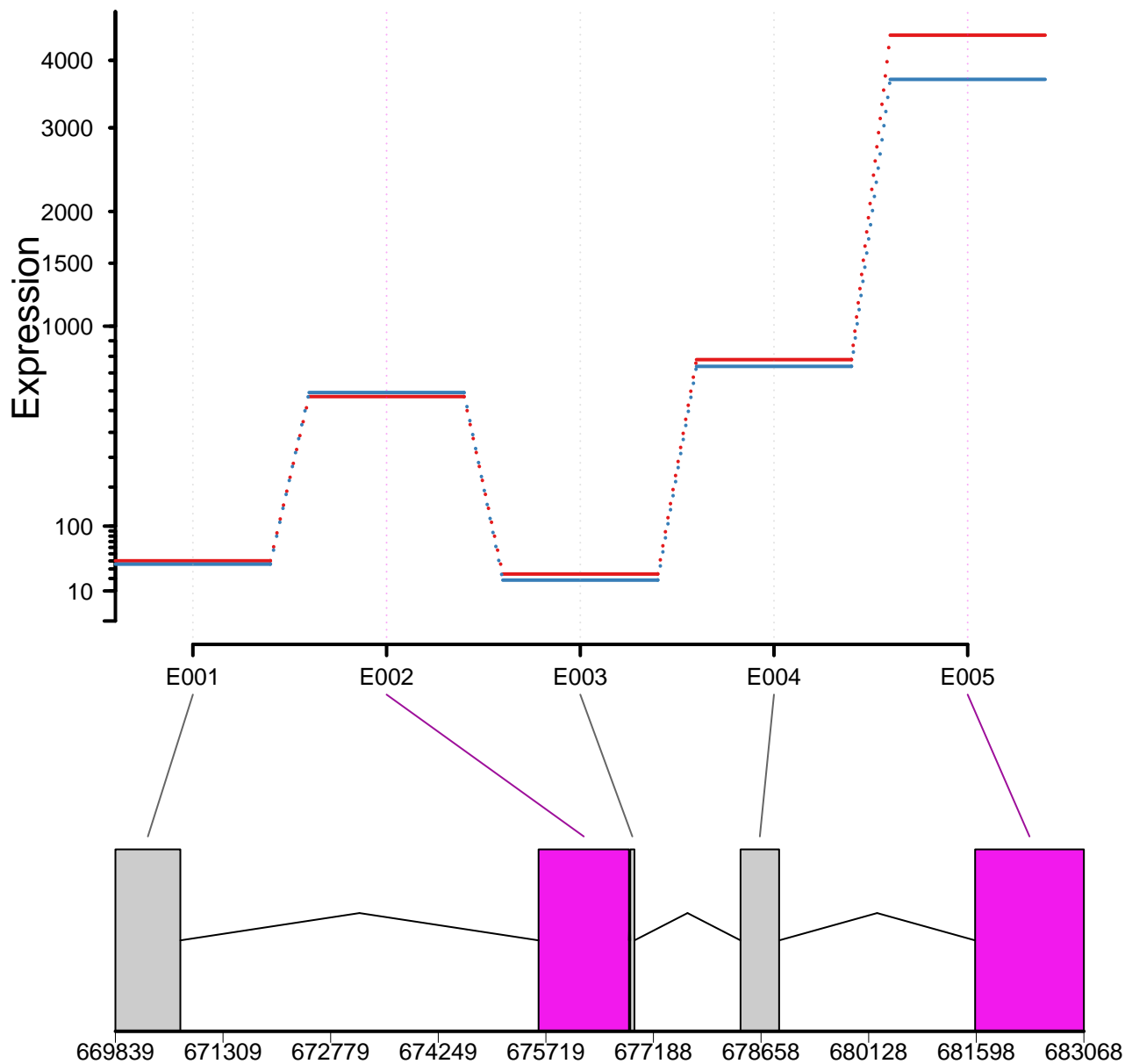

Supplement: Supplementary file 11 [file Data_Sheet_1.ZIP › Supplementary 17/gene-Nr2f2.pdf]

gene-Nr3c1 +

C

S

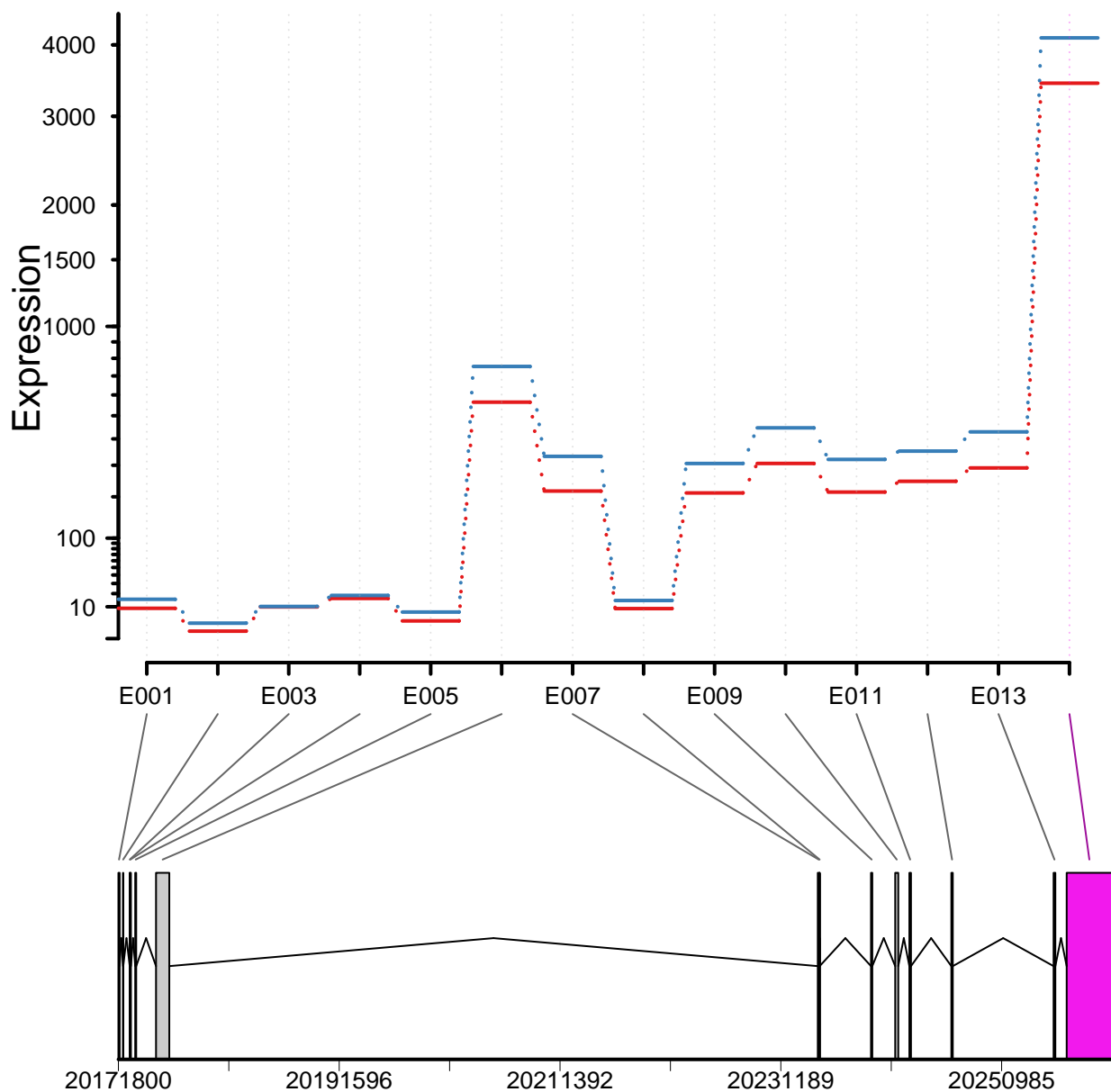

Supplement: Supplementary file 11 [file Data_Sheet_1.ZIP › Supplementary 17/gene-Nr3c1.pdf]

gene-Nrip1 +

C

S

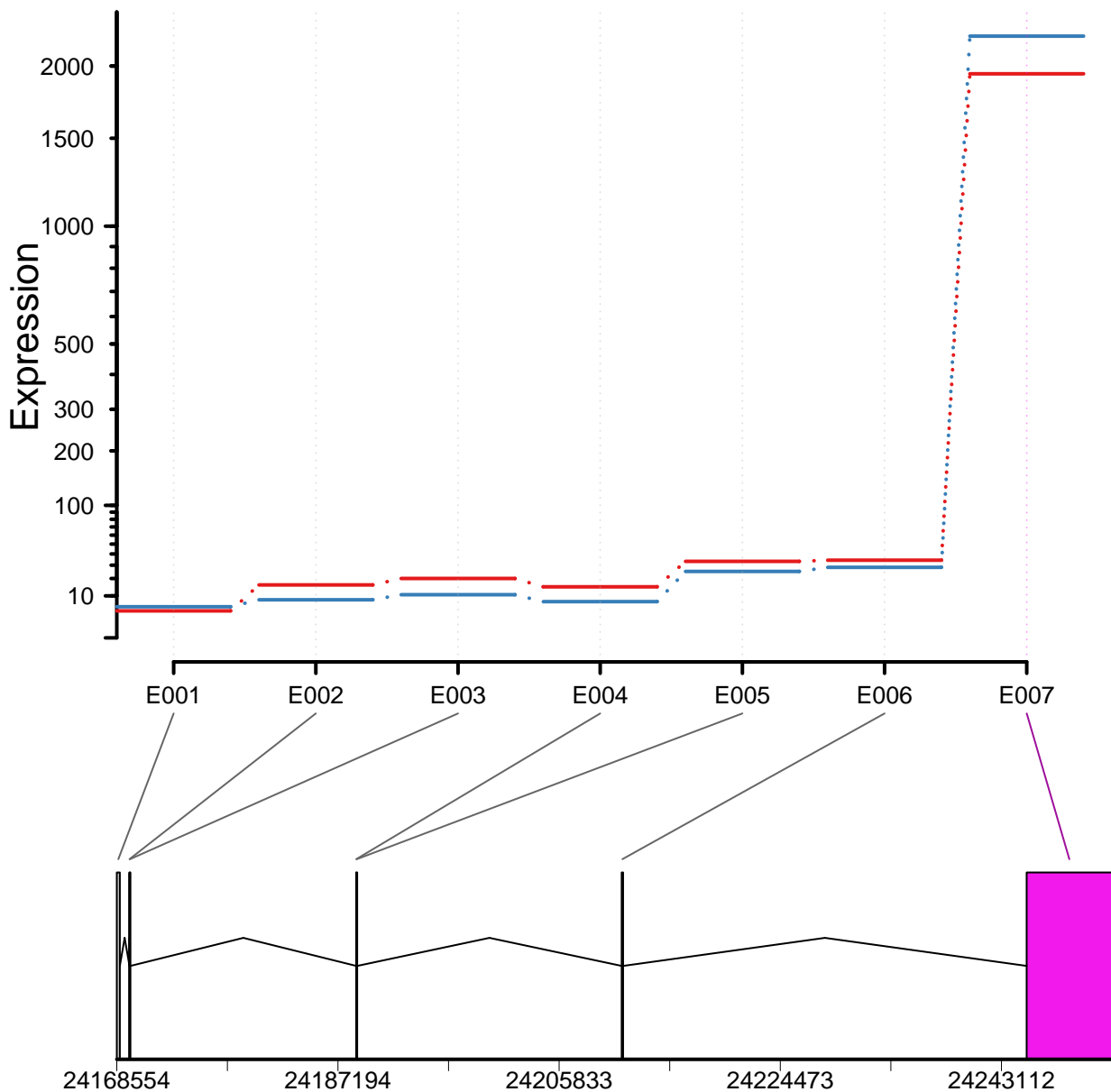

Supplement: Supplementary file 11 [file Data_Sheet_1.ZIP › Supplementary 17/gene-Nrip1.pdf]

gene-Nup58 +

C

S

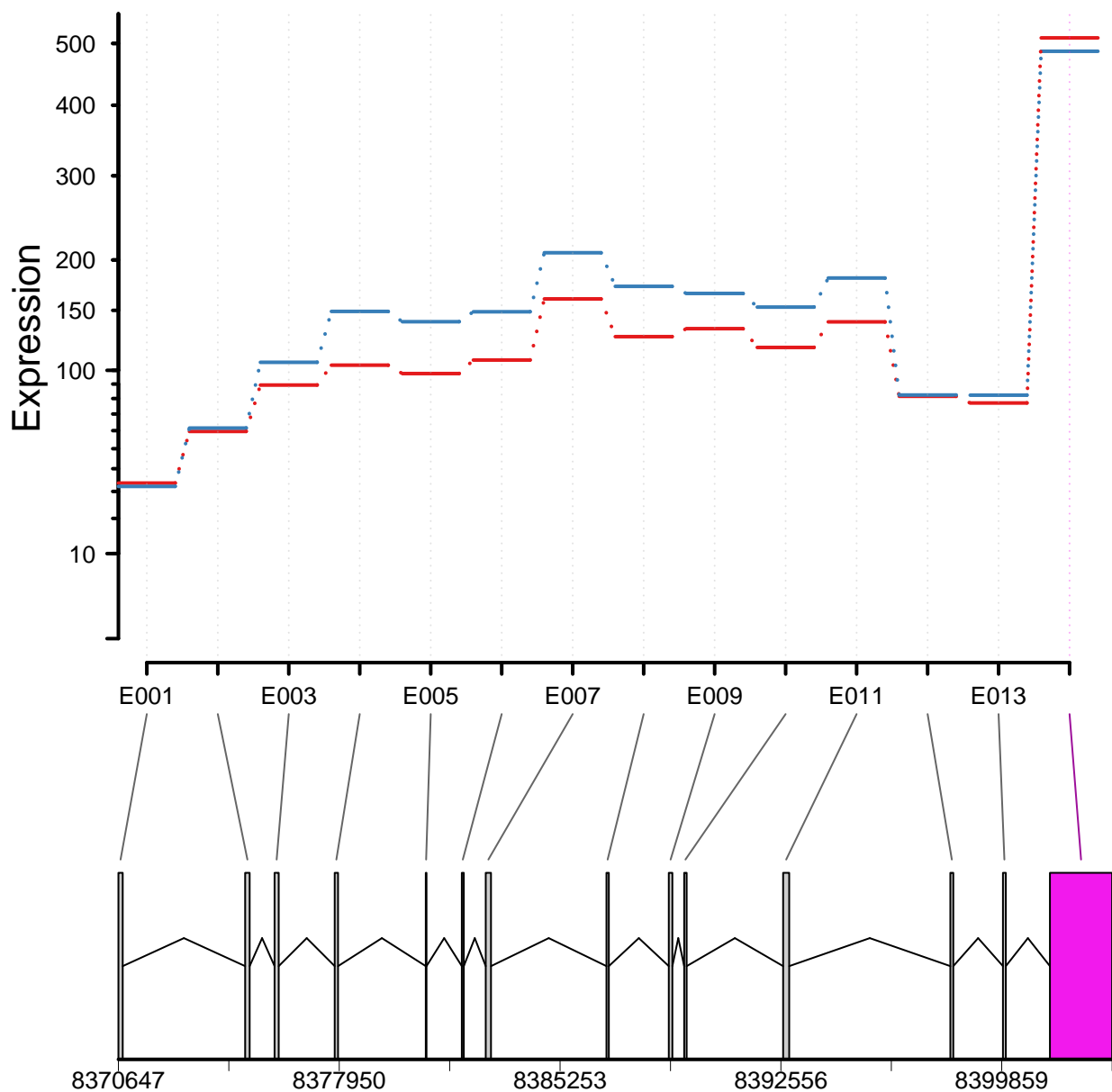

Supplement: Supplementary file 11 [file Data_Sheet_1.ZIP › Supplementary 17/gene-Nup58.pdf]

gene-Nup98 -

C

S

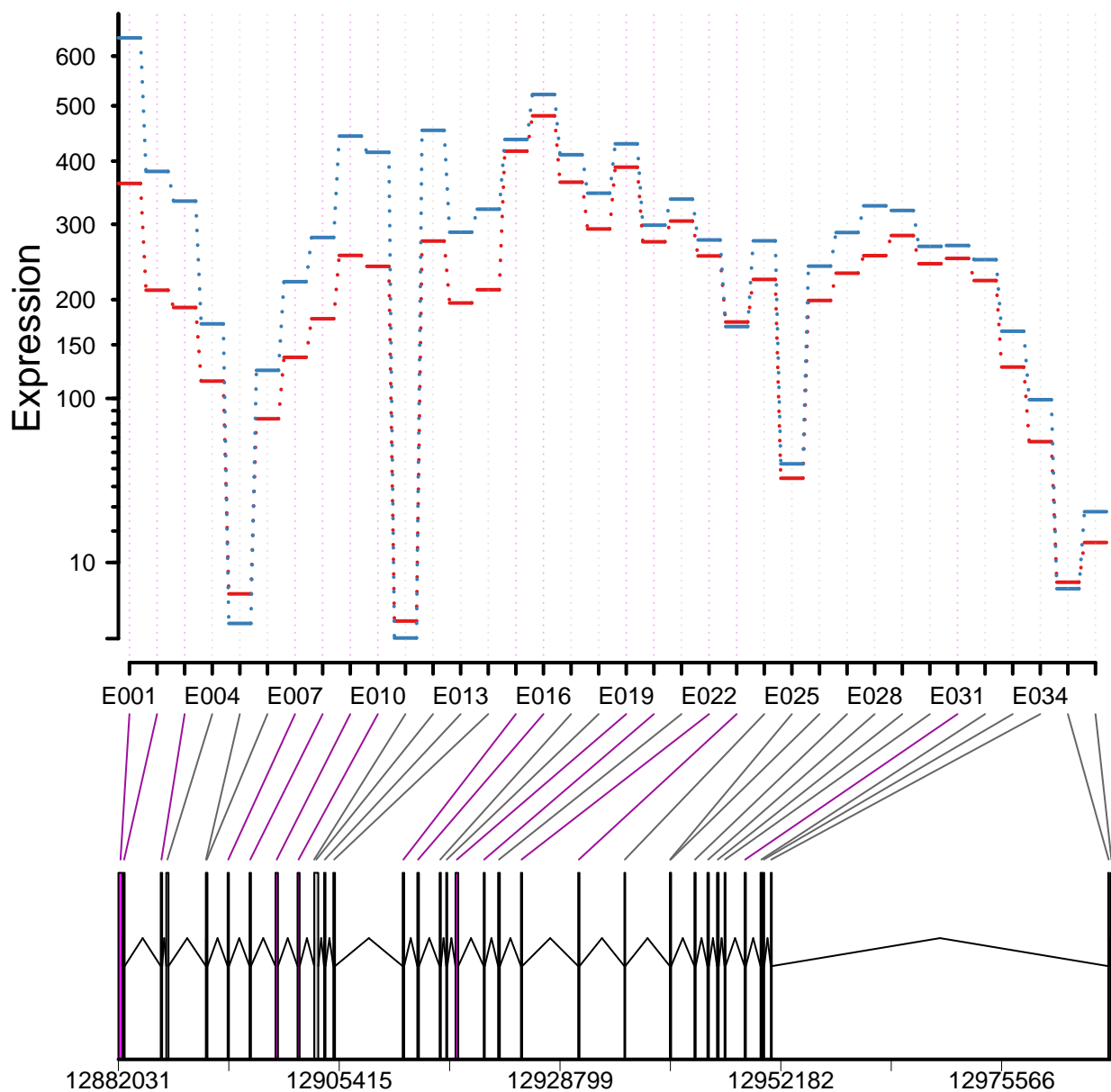

Supplement: Supplementary file 11 [file Data_Sheet_1.ZIP › Supplementary 17/gene-Nup98.pdf]

gene-Opa1 -

C

S

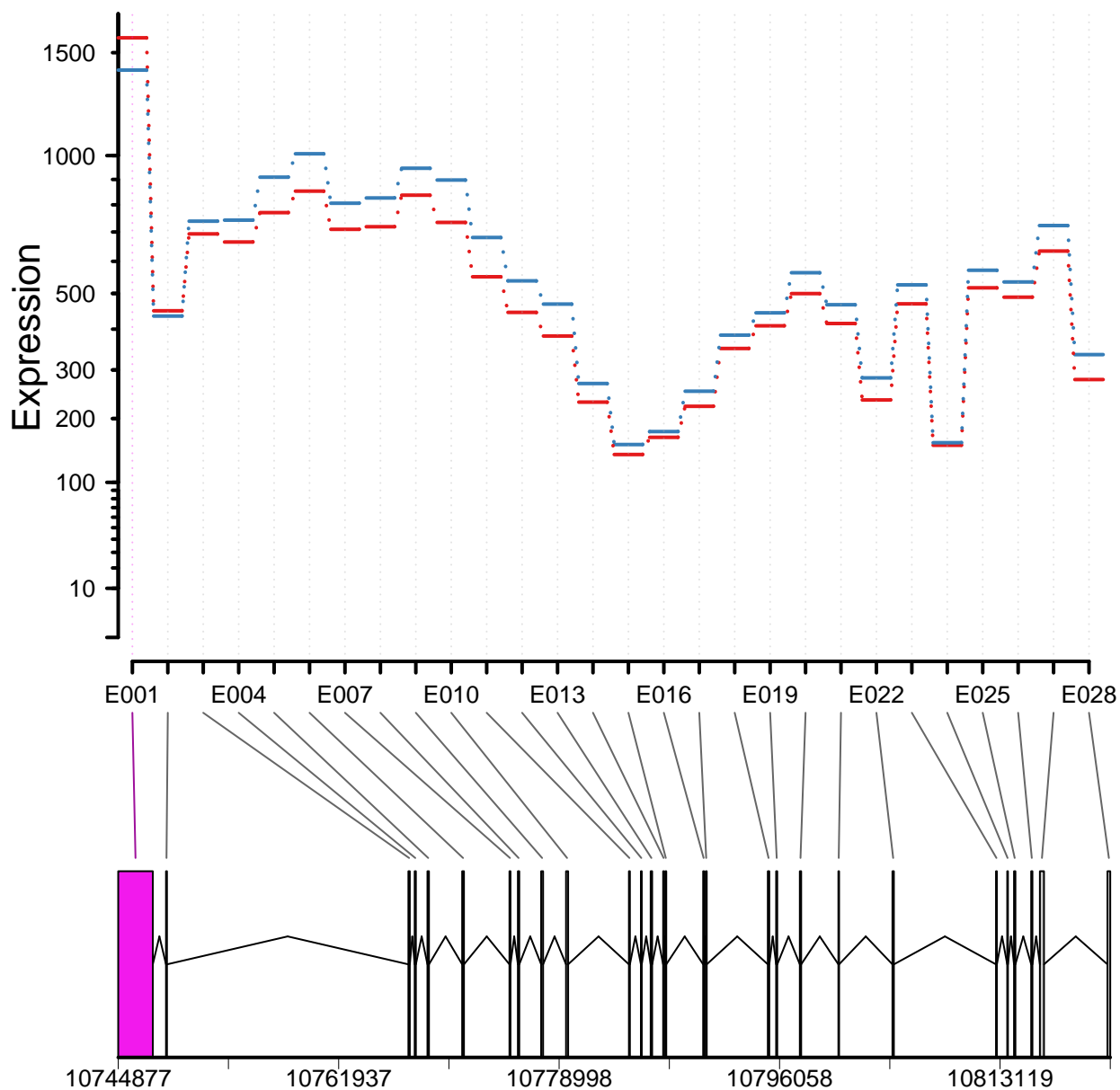

Supplement: Supplementary file 11 [file Data_Sheet_1.ZIP › Supplementary 17/gene-Opa1.pdf]

gene-Oxsr1 +

C

S

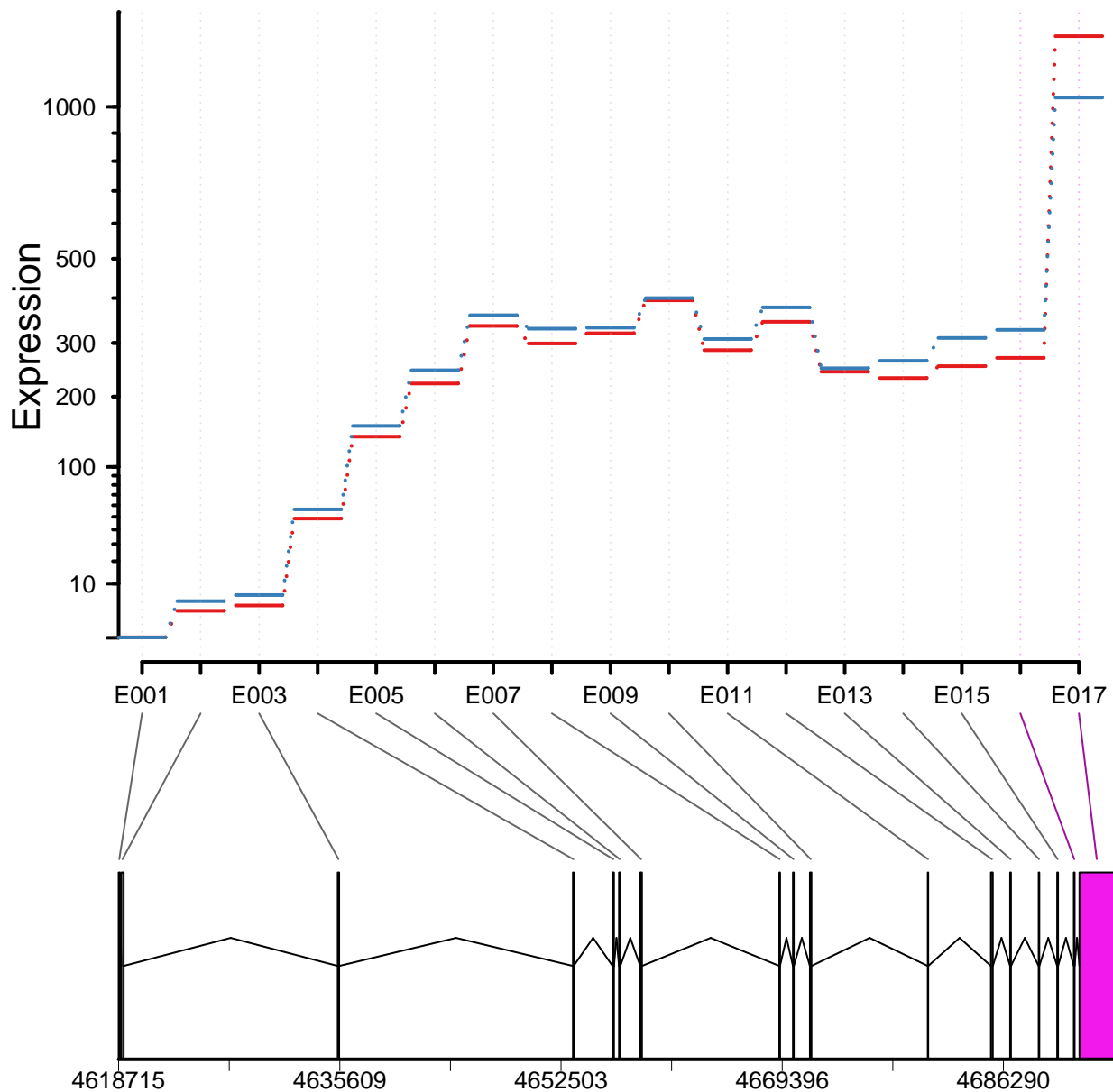

Supplement: Supplementary file 11 [file Data_Sheet_1.ZIP › Supplementary 17/gene-Oxsr1.pdf]

gene-Pafah1b1 +

C

S

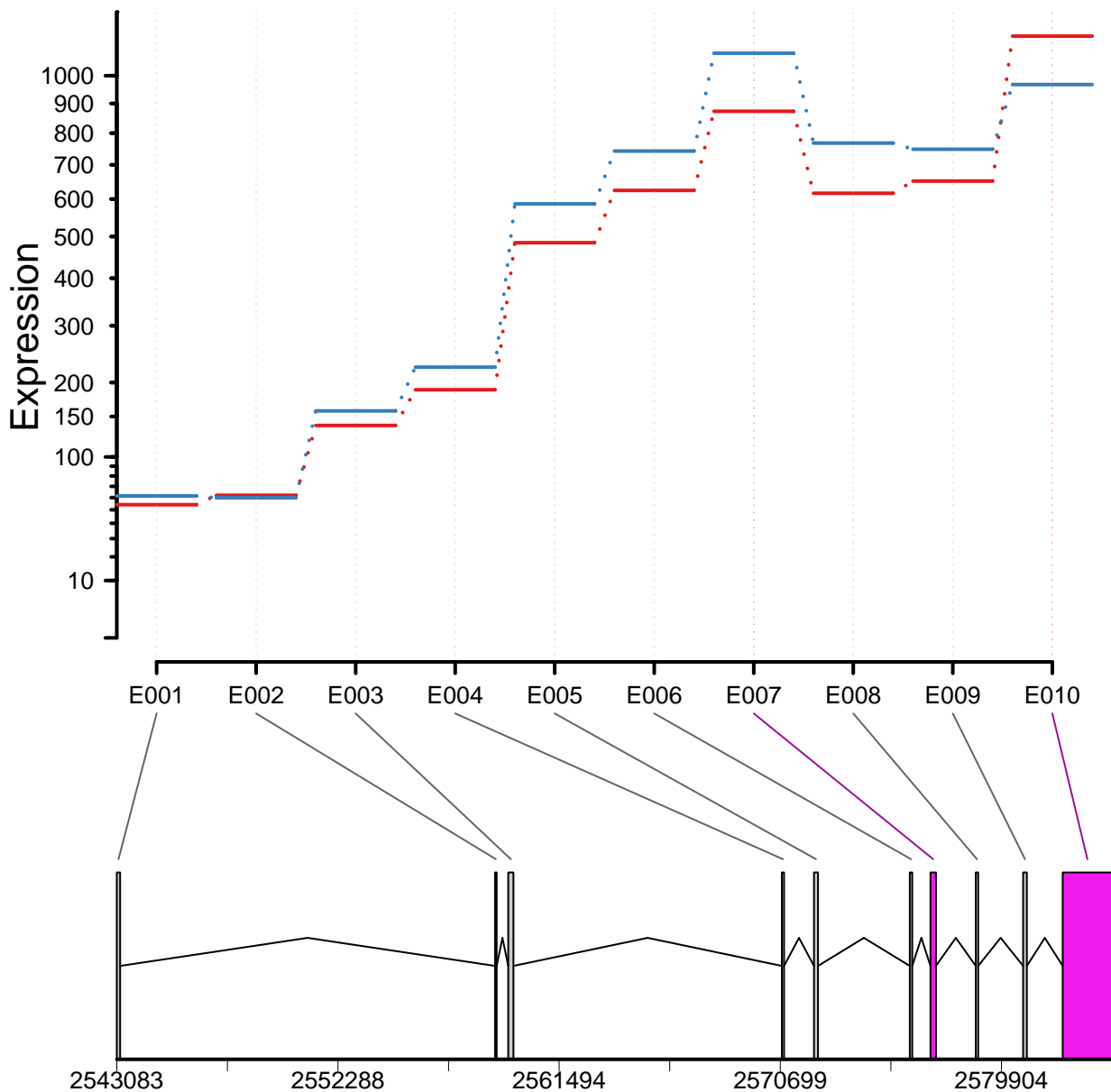

Supplement: Supplementary file 11 [file Data_Sheet_1.ZIP › Supplementary 17/gene-Pafah1b1.pdf]

gene-Pex11a -

C

S

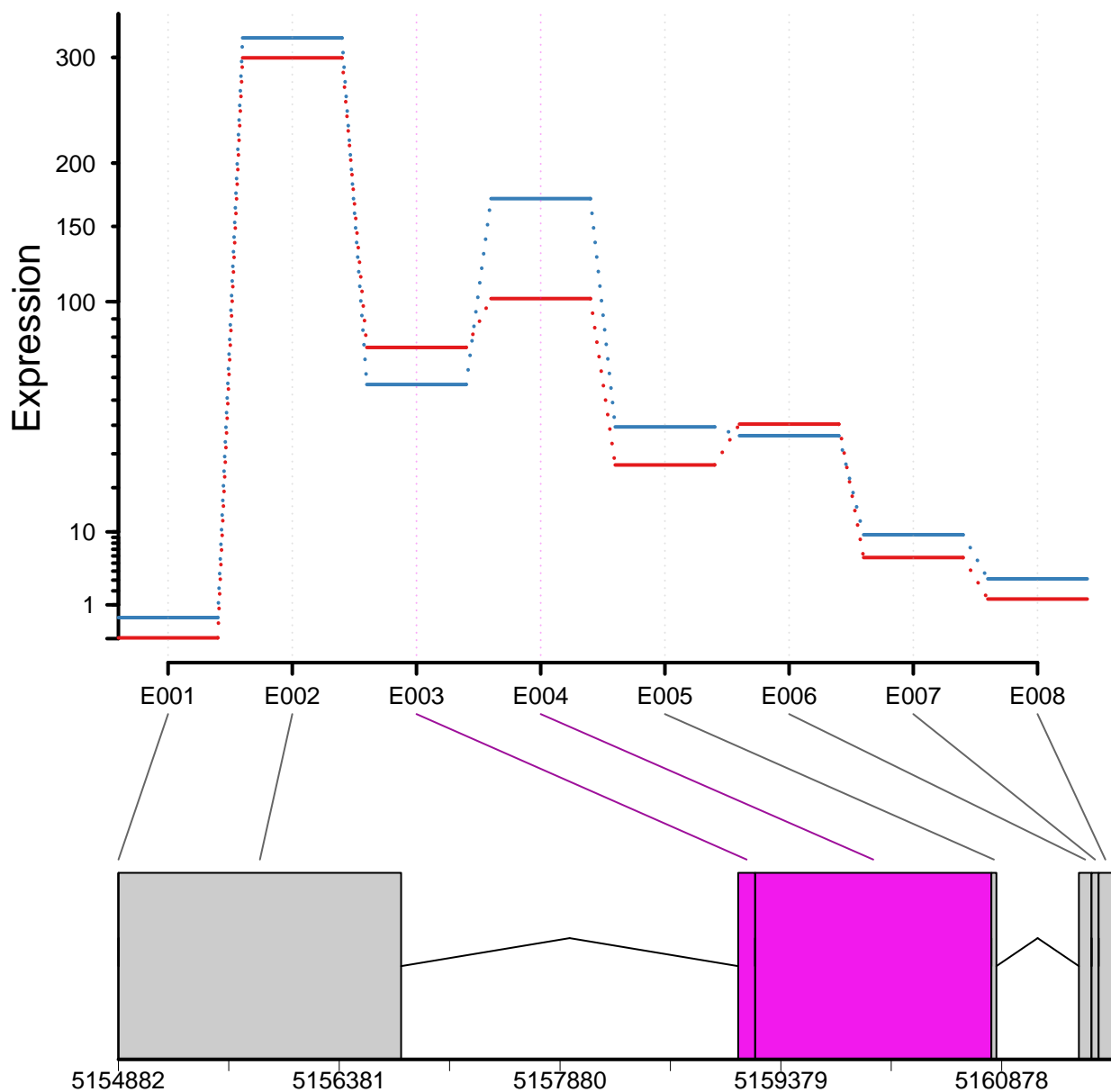

Supplement: Supplementary file 11 [file Data_Sheet_1.ZIP › Supplementary 17/gene-Pex11a.pdf]

gene-Pias1 +

C

S

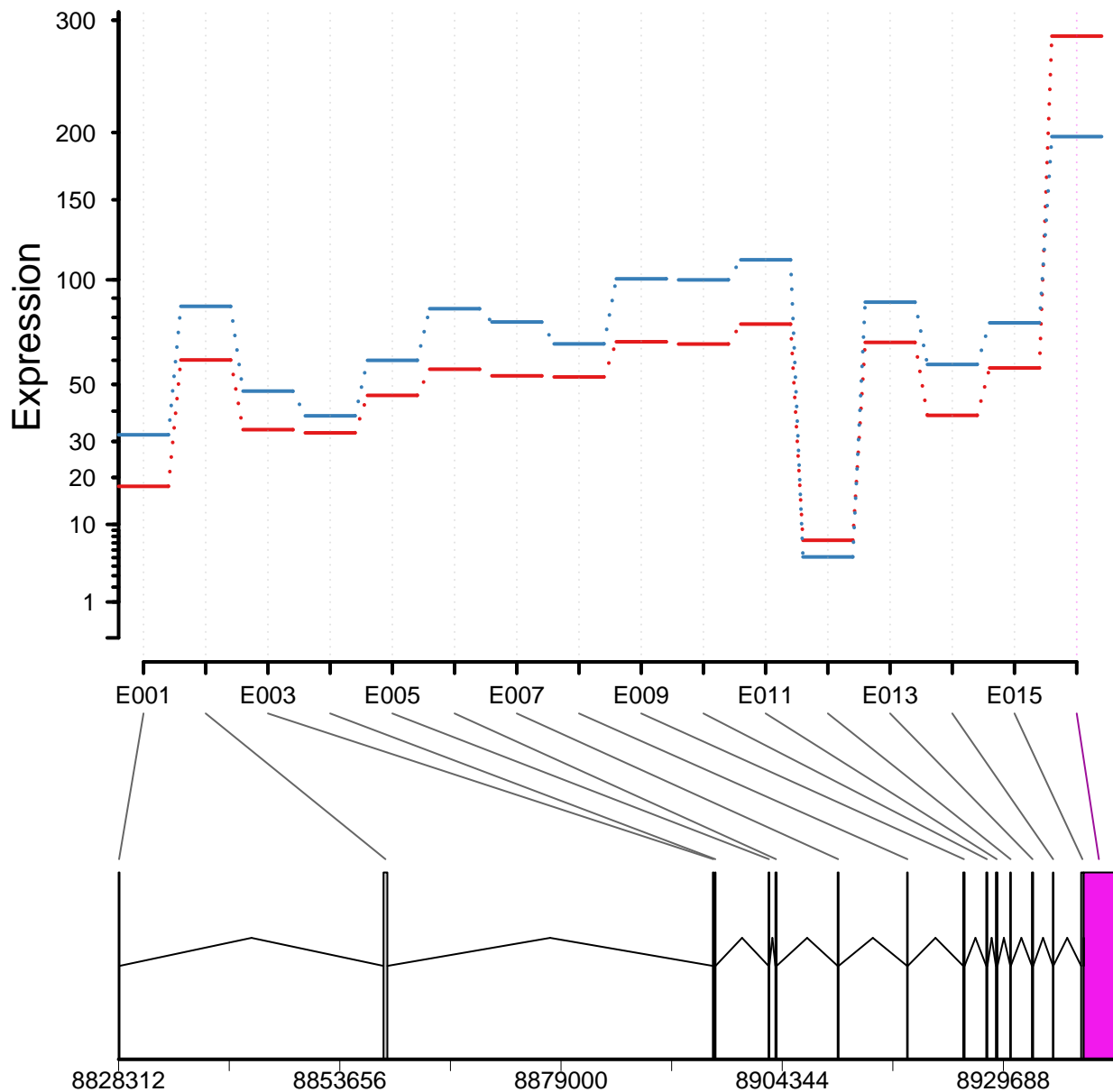

Supplement: Supplementary file 11 [file Data_Sheet_1.ZIP › Supplementary 17/gene-Pias1.pdf]

gene-Ppp1r2 +

C

S

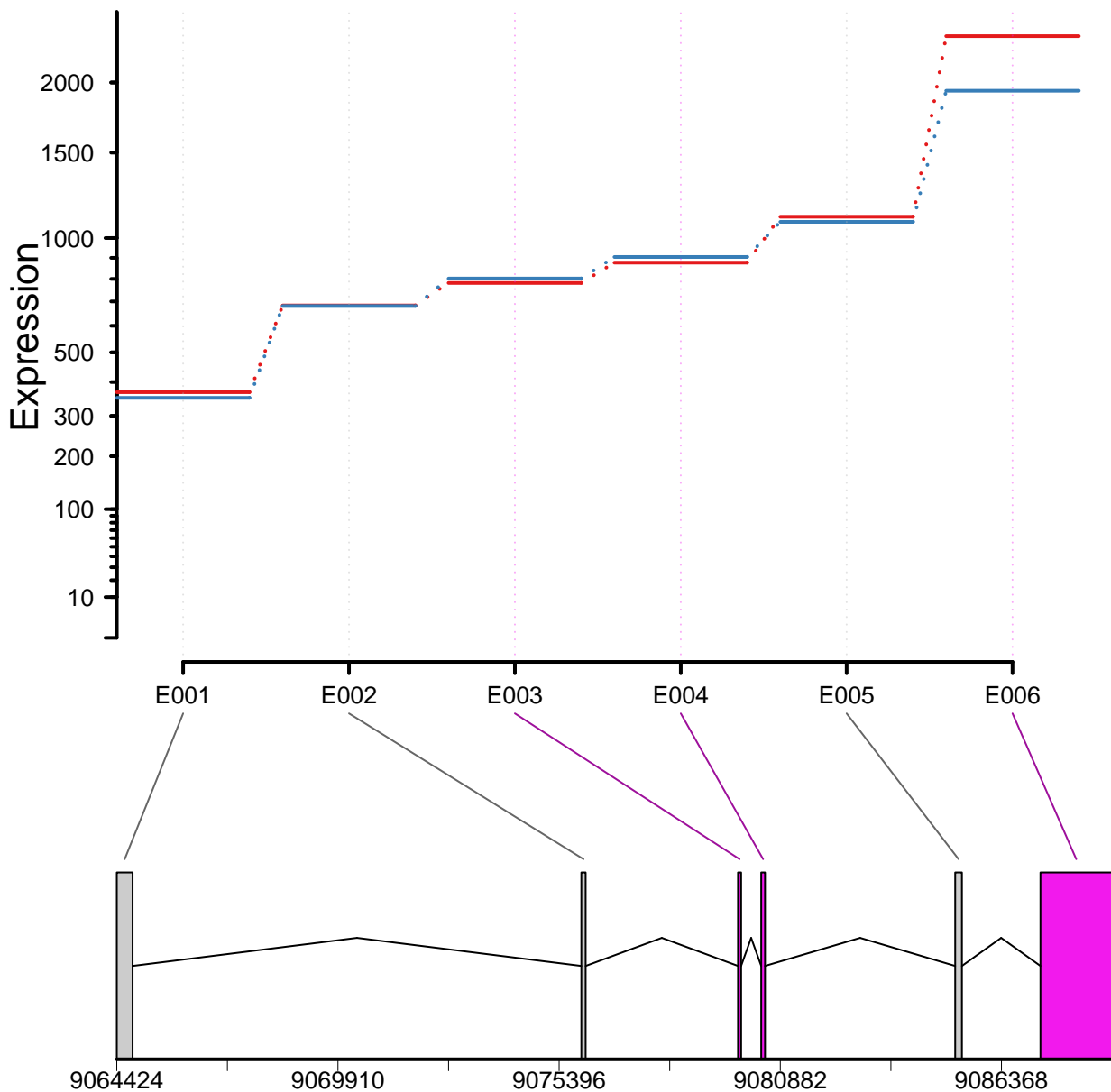

Supplement: Supplementary file 11 [file Data_Sheet_1.ZIP › Supplementary 17/gene-Ppp1r2.pdf]

gene-Ppp6r3 +

C

S

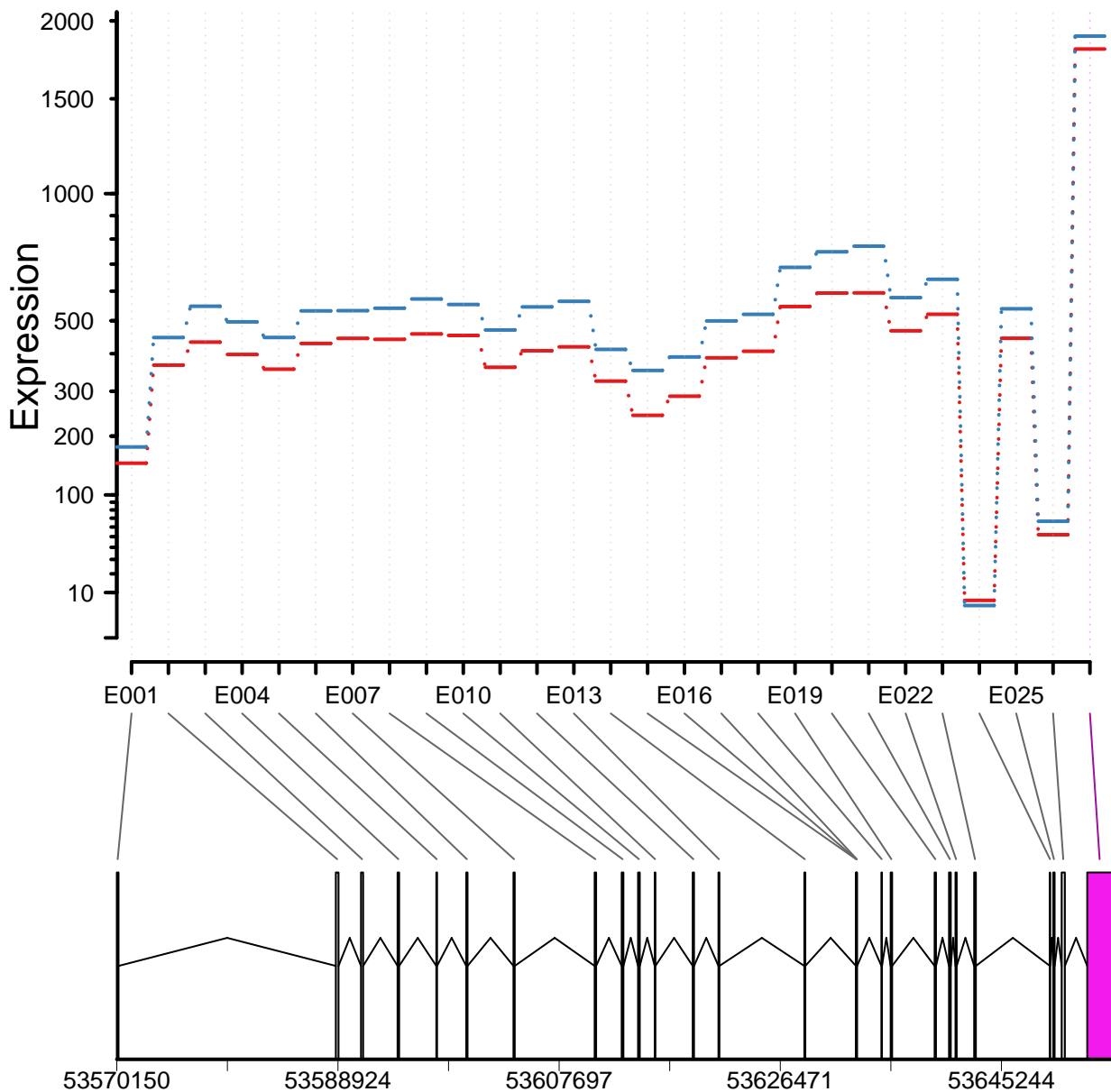

Supplement: Supplementary file 11 [file Data_Sheet_1.ZIP › Supplementary 17/gene-Ppp6r3.pdf]

gene-Prrc2c -

C

S

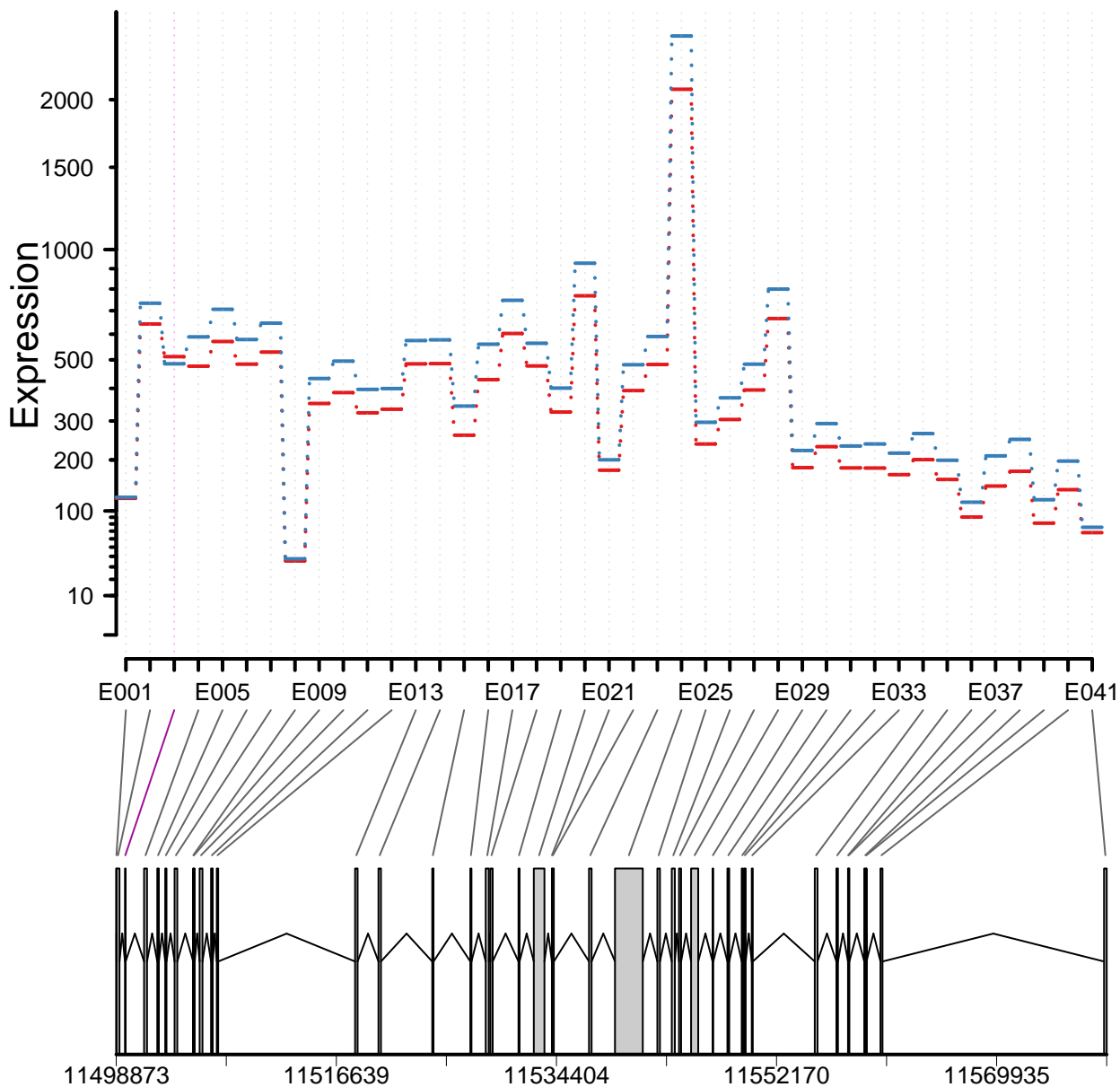

Supplement: Supplementary file 11 [file Data_Sheet_1.ZIP › Supplementary 17/gene-Prrc2c.pdf]

gene-Ptprb +

C

S

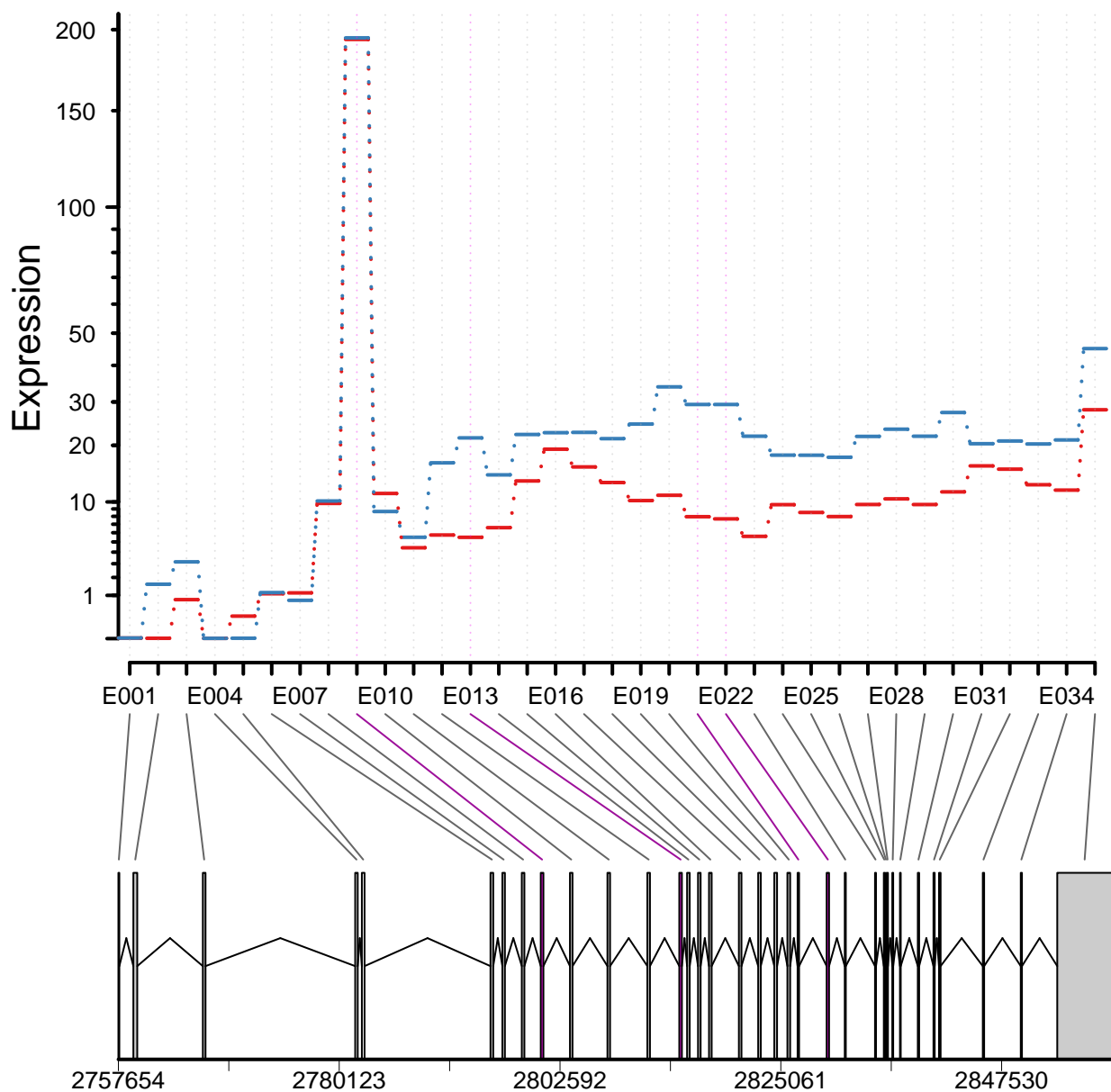

Supplement: Supplementary file 11 [file Data_Sheet_1.ZIP › Supplementary 17/gene-Ptprb.pdf]

gene-Pum2 -

C

S

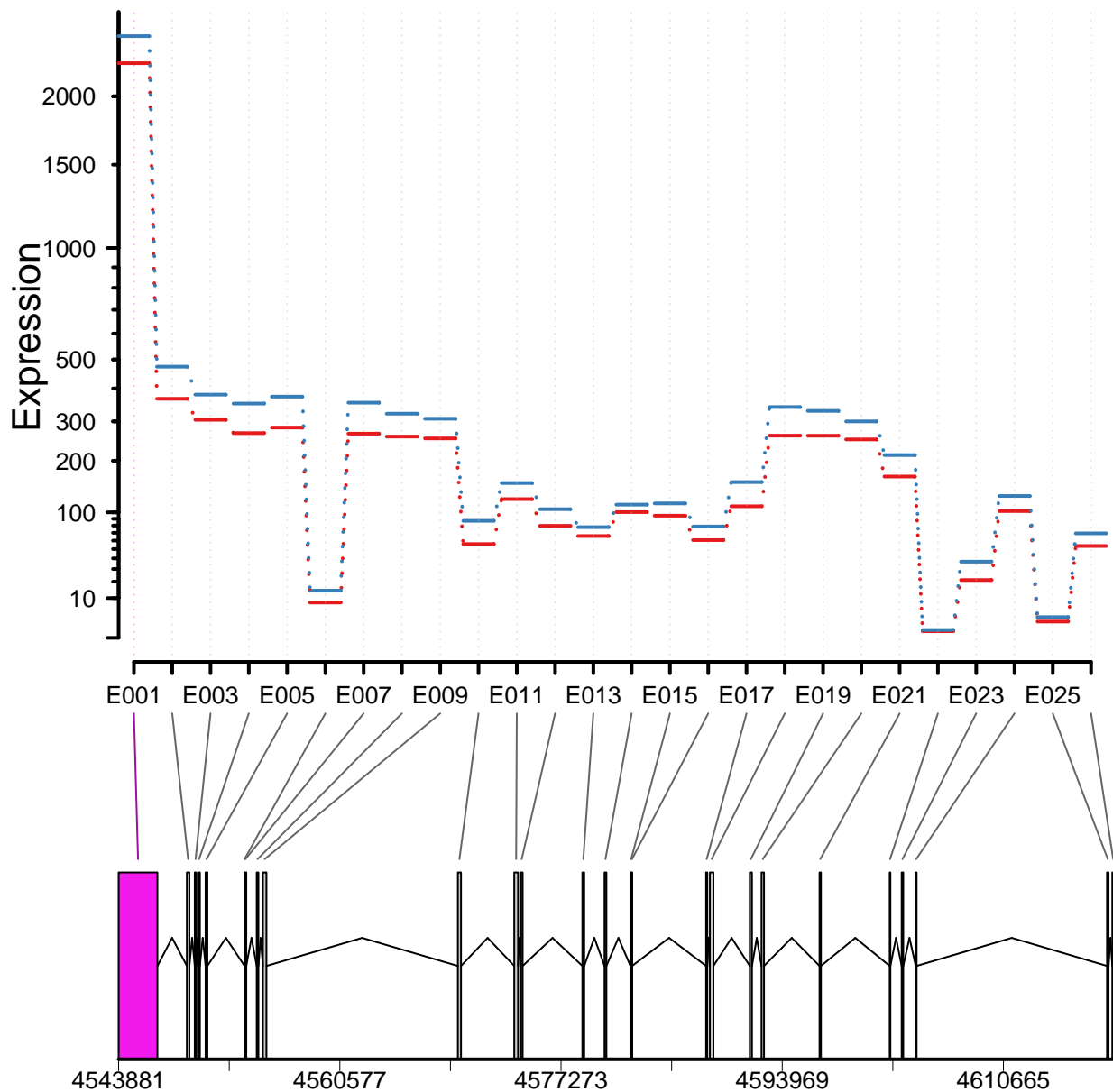

Supplement: Supplementary file 11 [file Data_Sheet_1.ZIP › Supplementary 17/gene-Pum2.pdf]

gene-Rab2a +

C

S

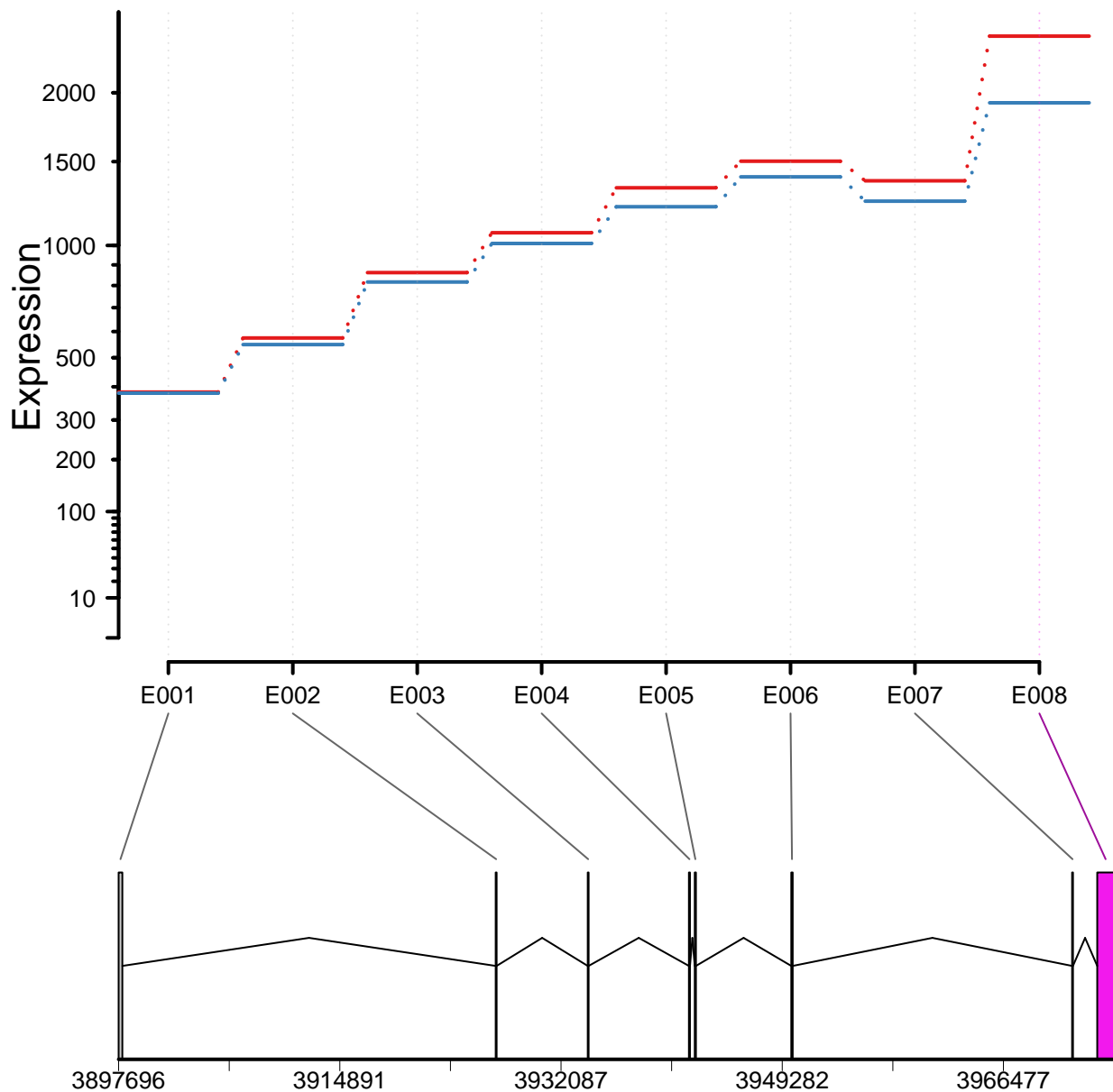

Supplement: Supplementary file 11 [file Data_Sheet_1.ZIP › Supplementary 17/gene-Rab2a.pdf]

gene-Rassf6 +

C

S

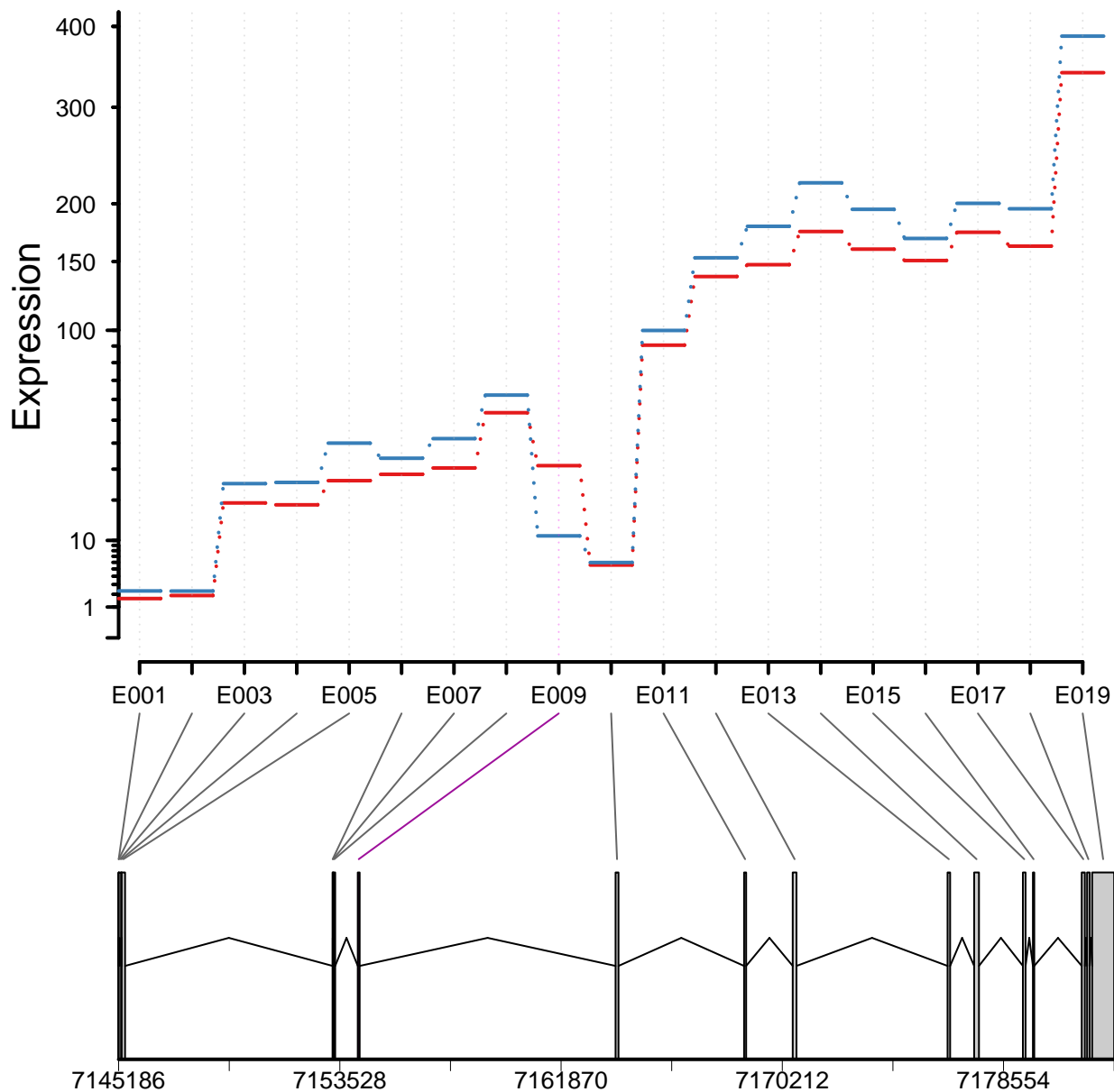

Supplement: Supplementary file 11 [file Data_Sheet_1.ZIP › Supplementary 17/gene-Rassf6.pdf]

gene-Rbm12b -

C

S

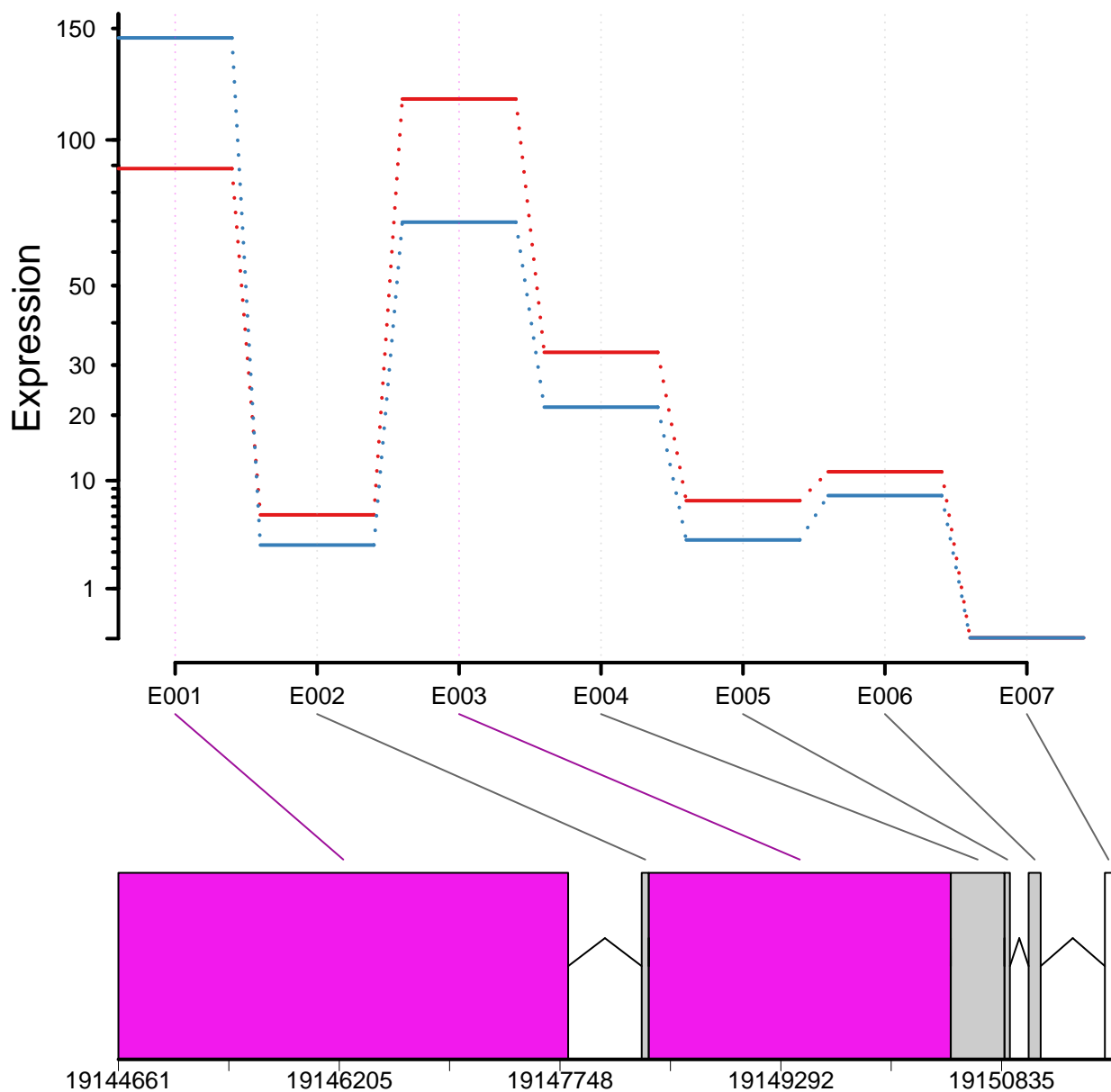

Supplement: Supplementary file 11 [file Data_Sheet_1.ZIP › Supplementary 17/gene-Rbm12b.pdf]

gene-Rbms1 -

C

S

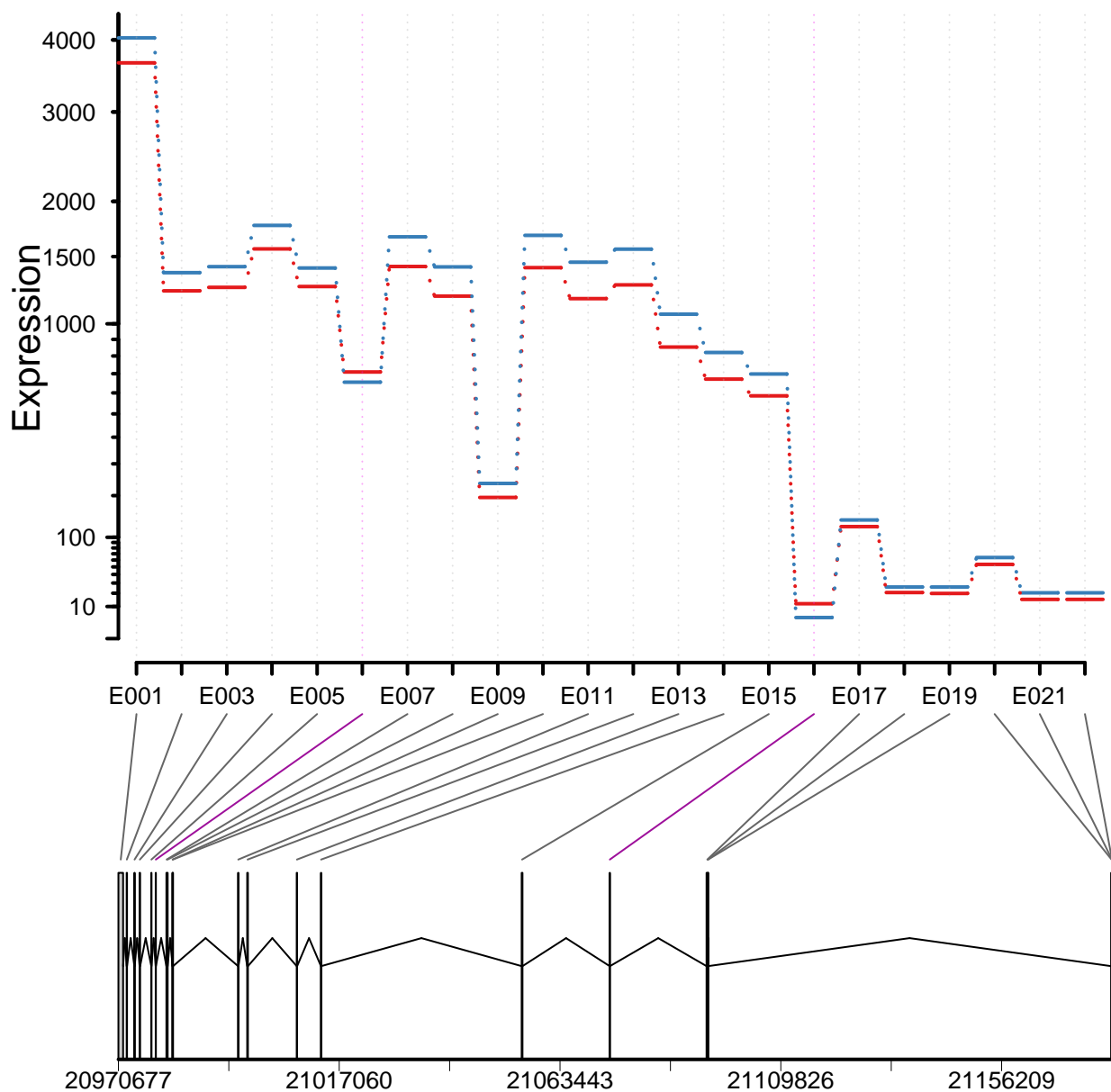

Supplement: Supplementary file 11 [file Data_Sheet_1.ZIP › Supplementary 17/gene-Rbms1.pdf]

gene-Rbpj -

C

S

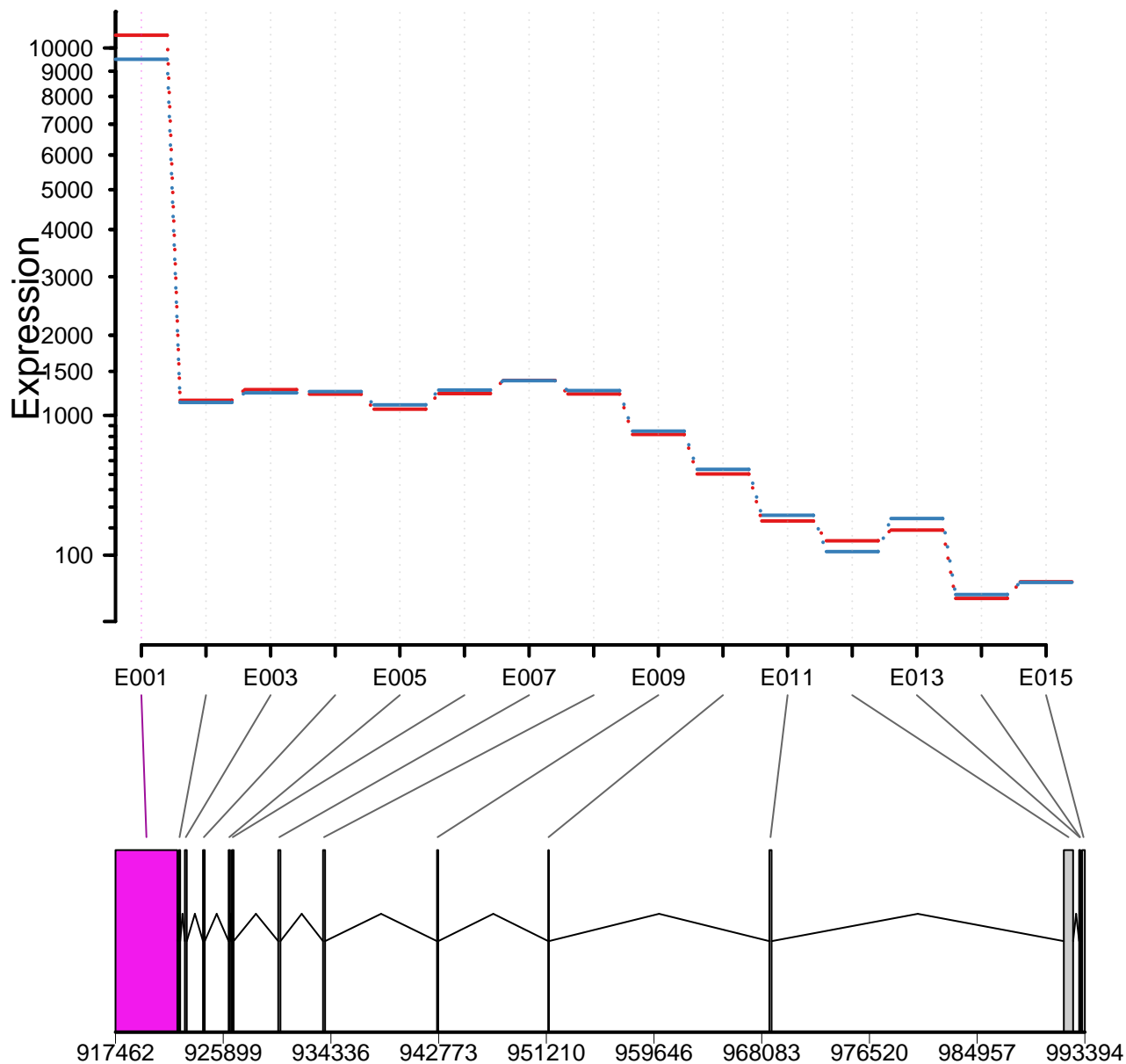

Supplement: Supplementary file 11 [file Data_Sheet_1.ZIP › Supplementary 17/gene-Rbpj.pdf]

gene-Sdhaf4 +

C

S

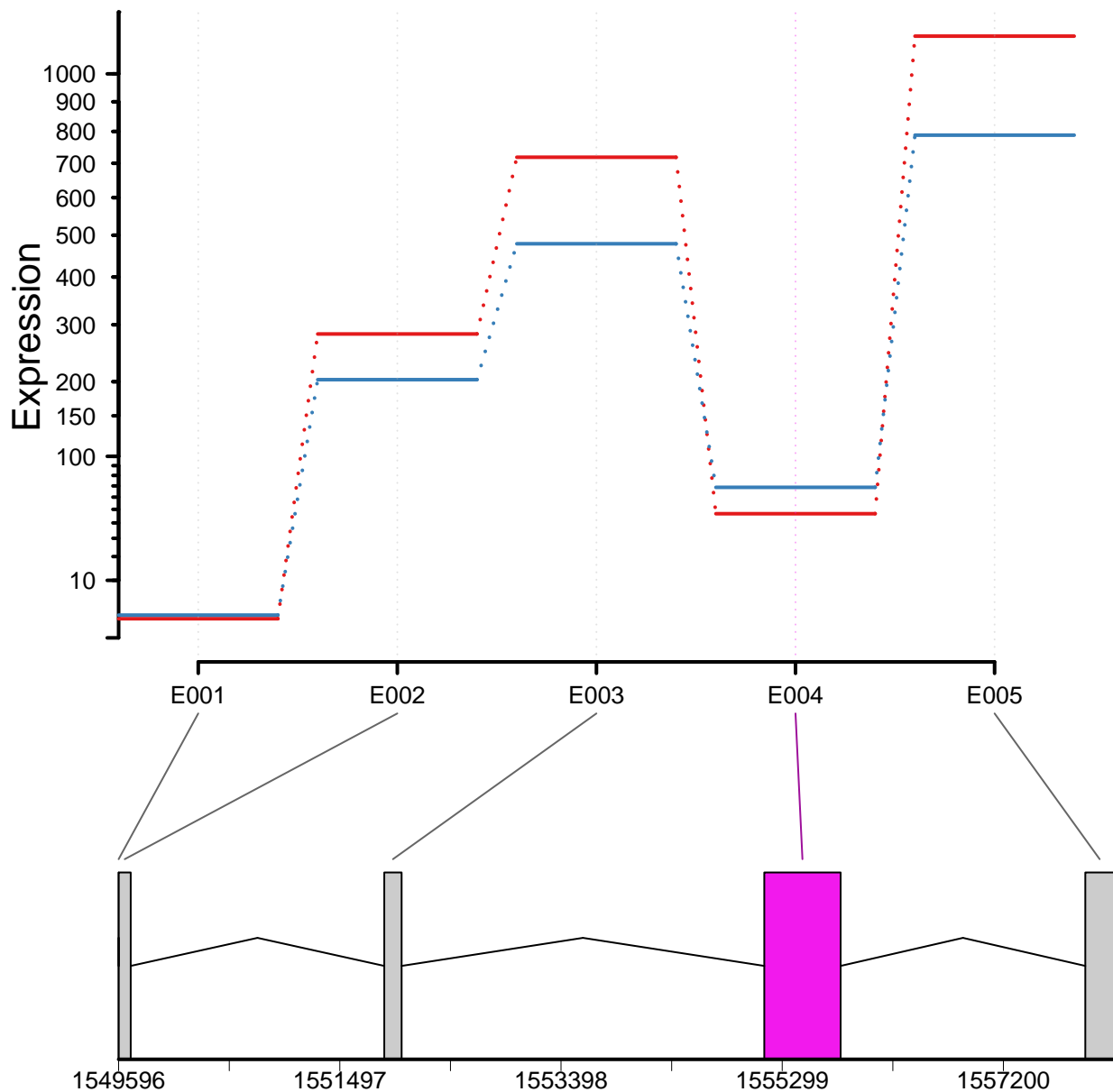

Supplement: Supplementary file 11 [file Data_Sheet_1.ZIP › Supplementary 17/gene-Sdhaf4.pdf]

gene-Sec24a +

C

S

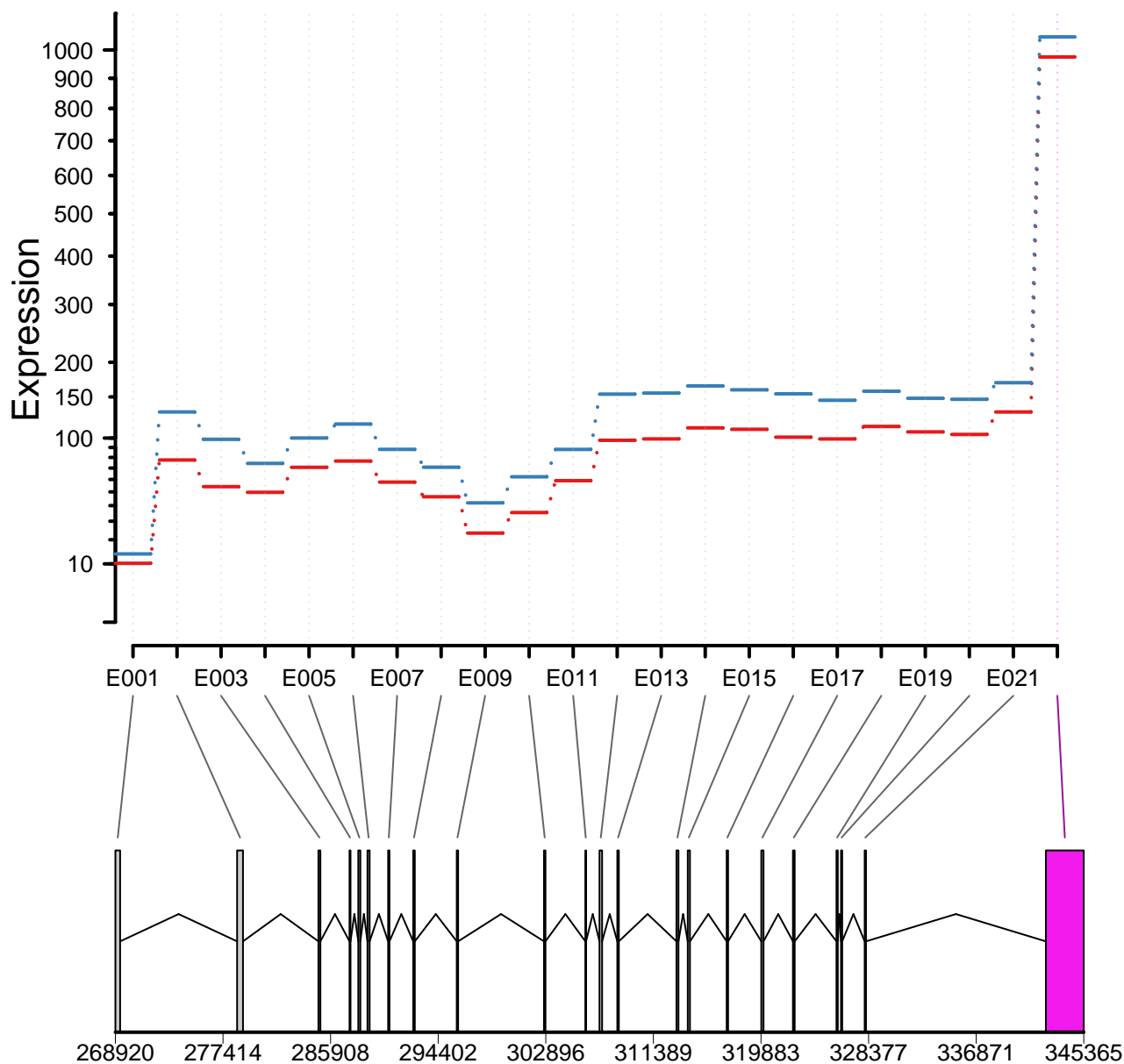

Supplement: Supplementary file 11 [file Data_Sheet_1.ZIP › Supplementary 17/gene-Sec24a.pdf]

gene-Selenoi +

C

S

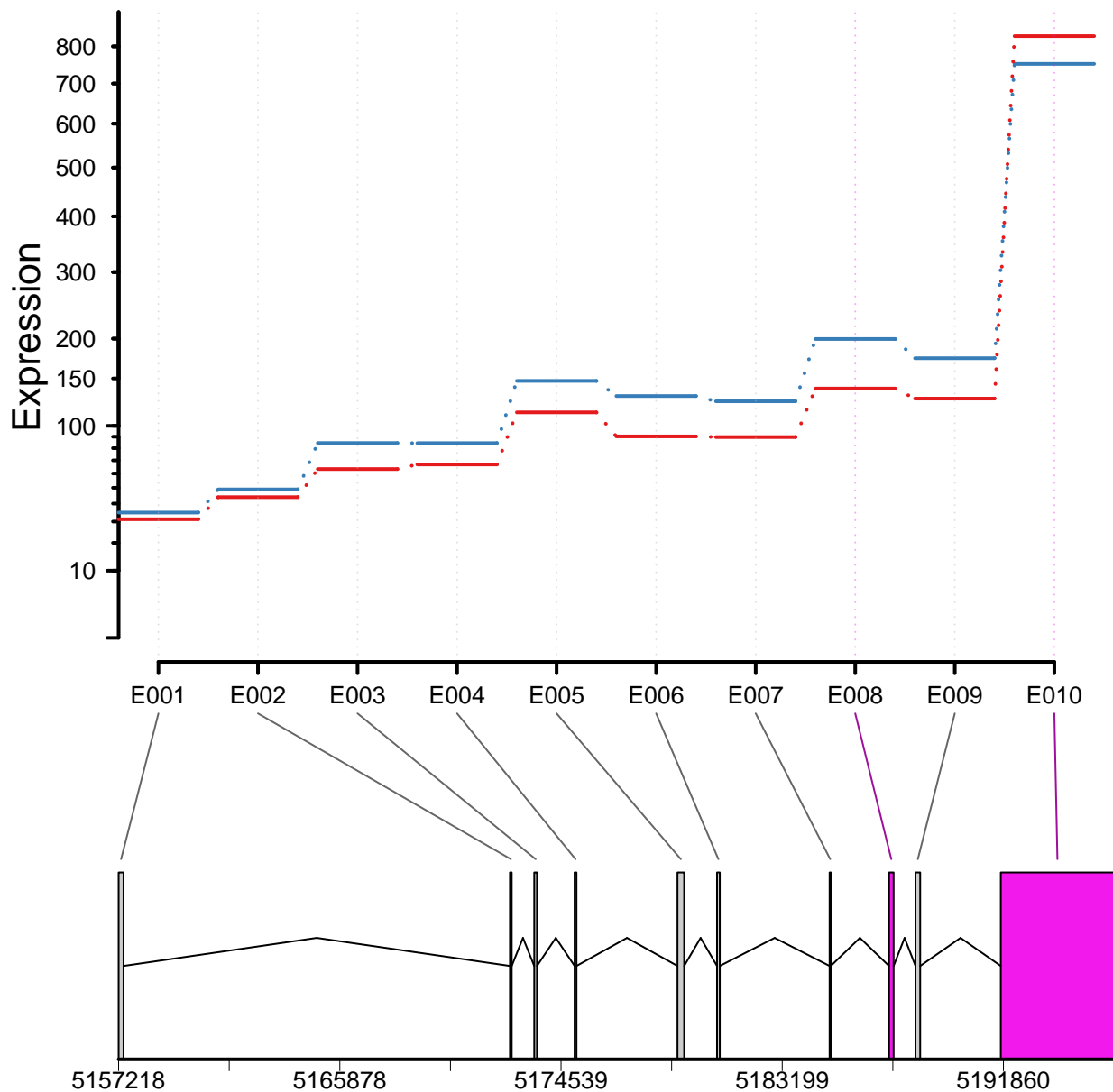

Supplement: Supplementary file 11 [file Data_Sheet_1.ZIP › Supplementary 17/gene-Selenoi.pdf]

gene-Serpinh1 -

C

S

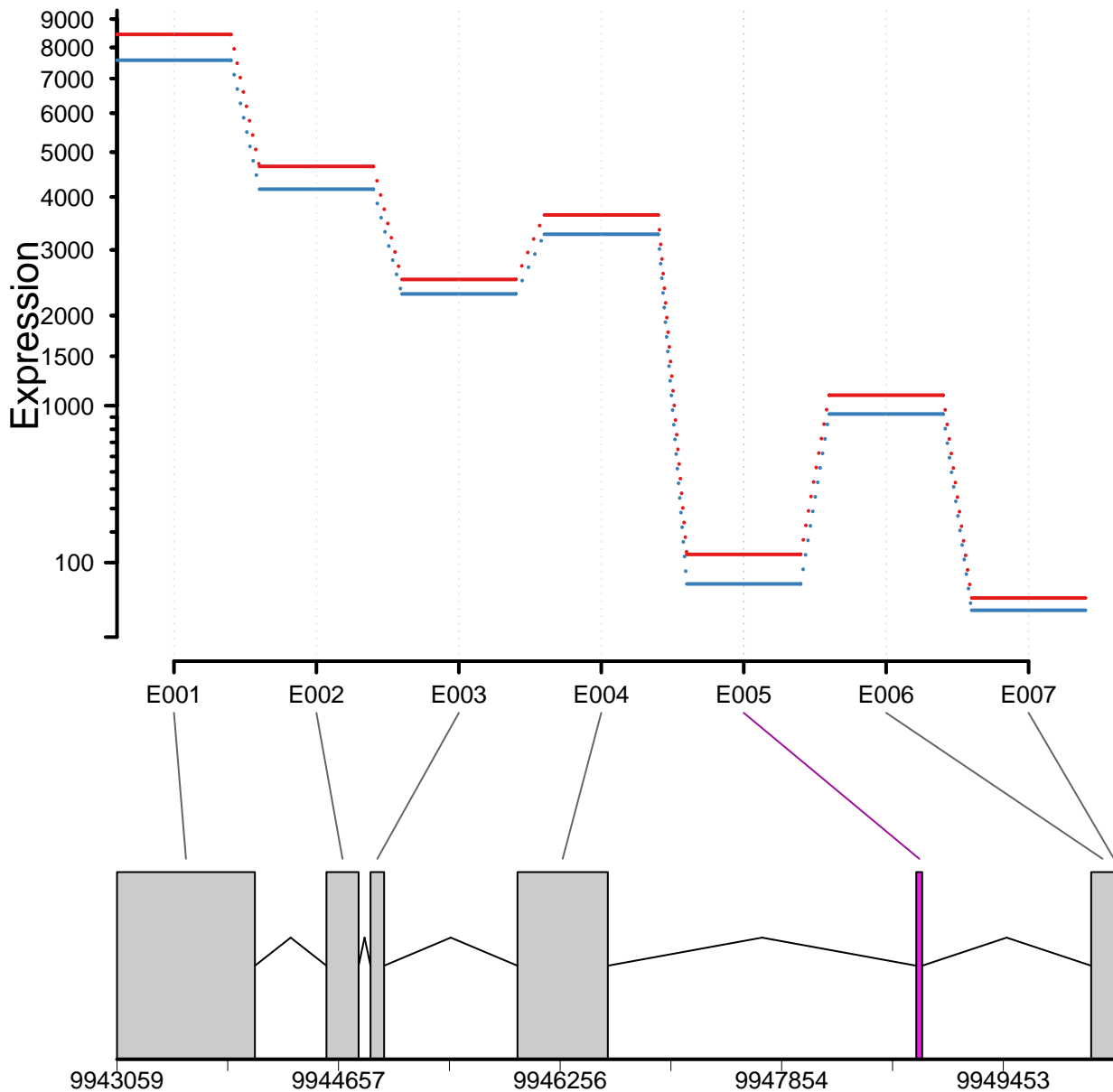

Supplement: Supplementary file 11 [file Data_Sheet_1.ZIP › Supplementary 17/gene-Serpinh1.pdf]

gene-Slc25a46 +

C

S

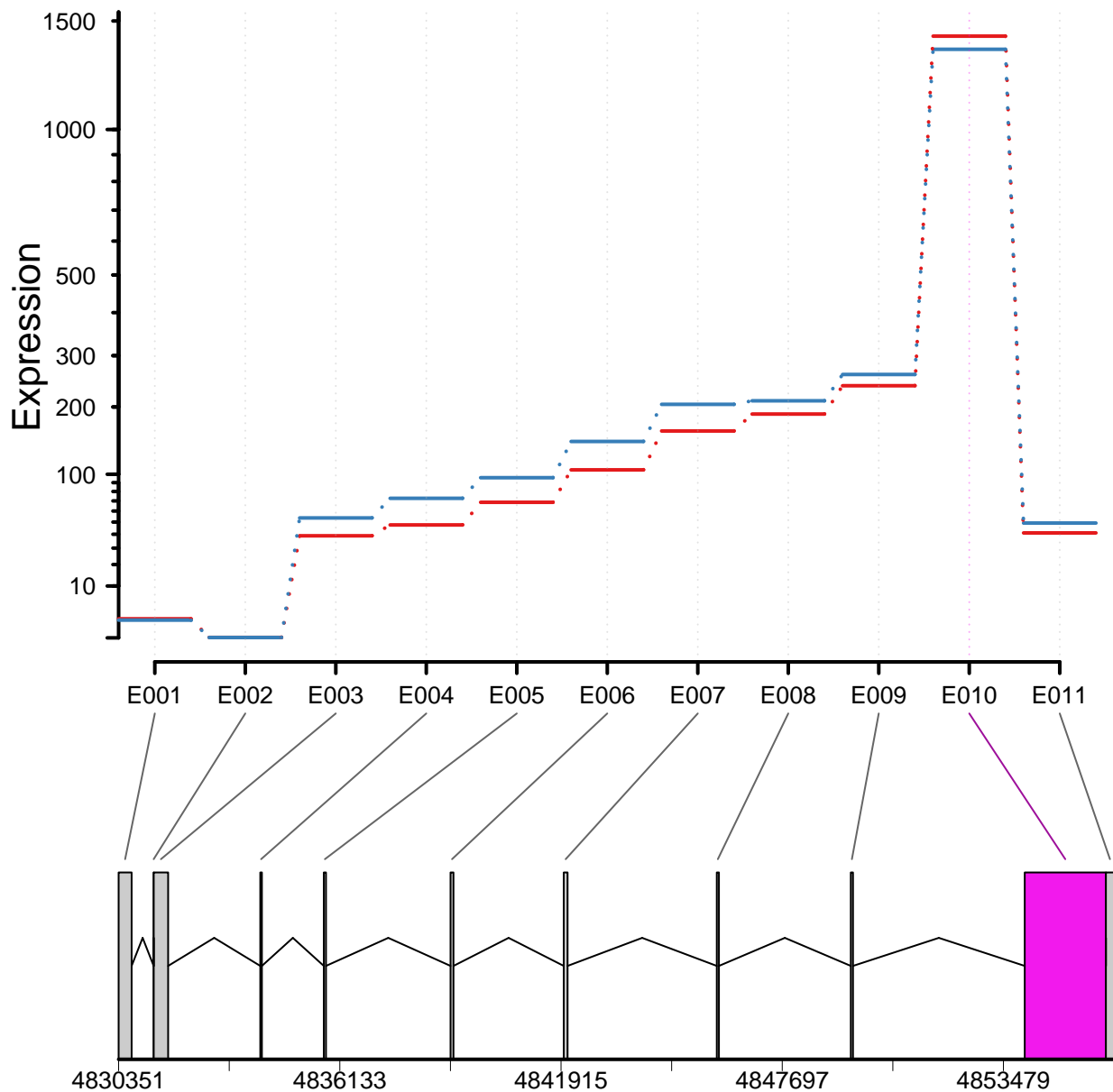

Supplement: Supplementary file 11 [file Data_Sheet_1.ZIP › Supplementary 17/gene-Slc25a46.pdf]

gene-Smc5 +

C

S

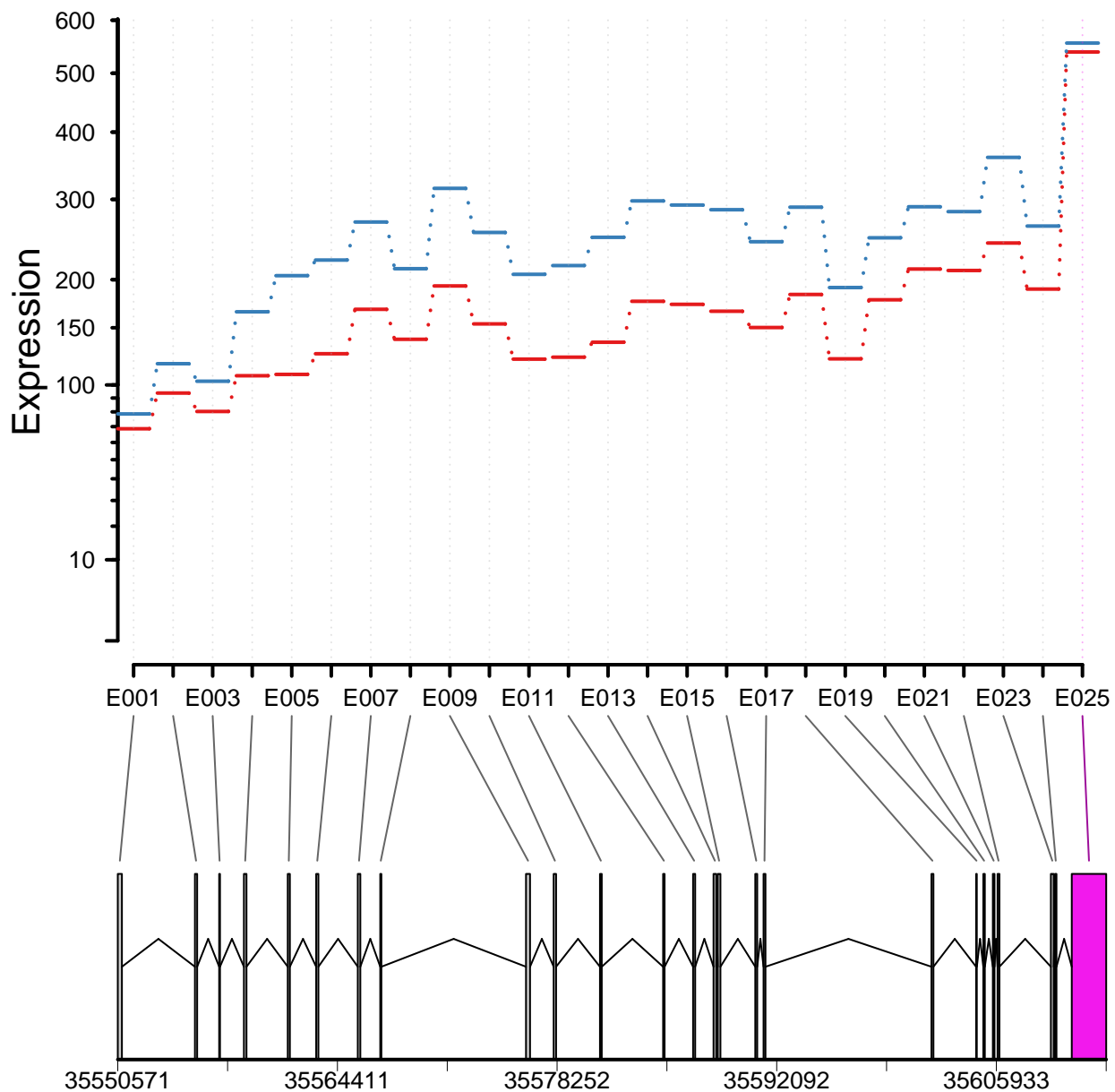

Supplement: Supplementary file 11 [file Data_Sheet_1.ZIP › Supplementary 17/gene-Smc5.pdf]

gene-Sptbn1 +

C

S

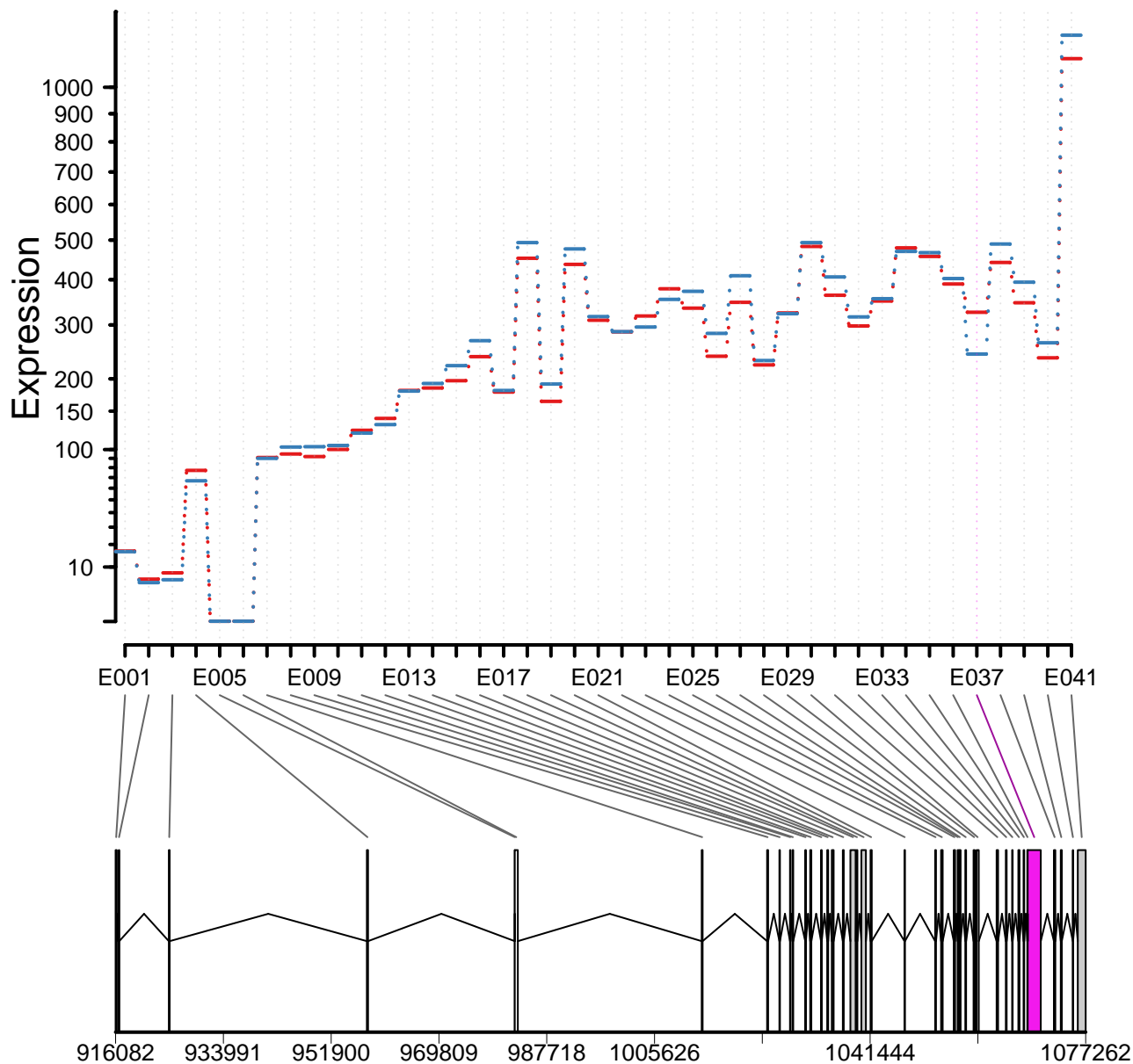

Supplement: Supplementary file 11 [file Data_Sheet_1.ZIP › Supplementary 17/gene-Sptbn1.pdf]

gene-Srsf11 +

C

S

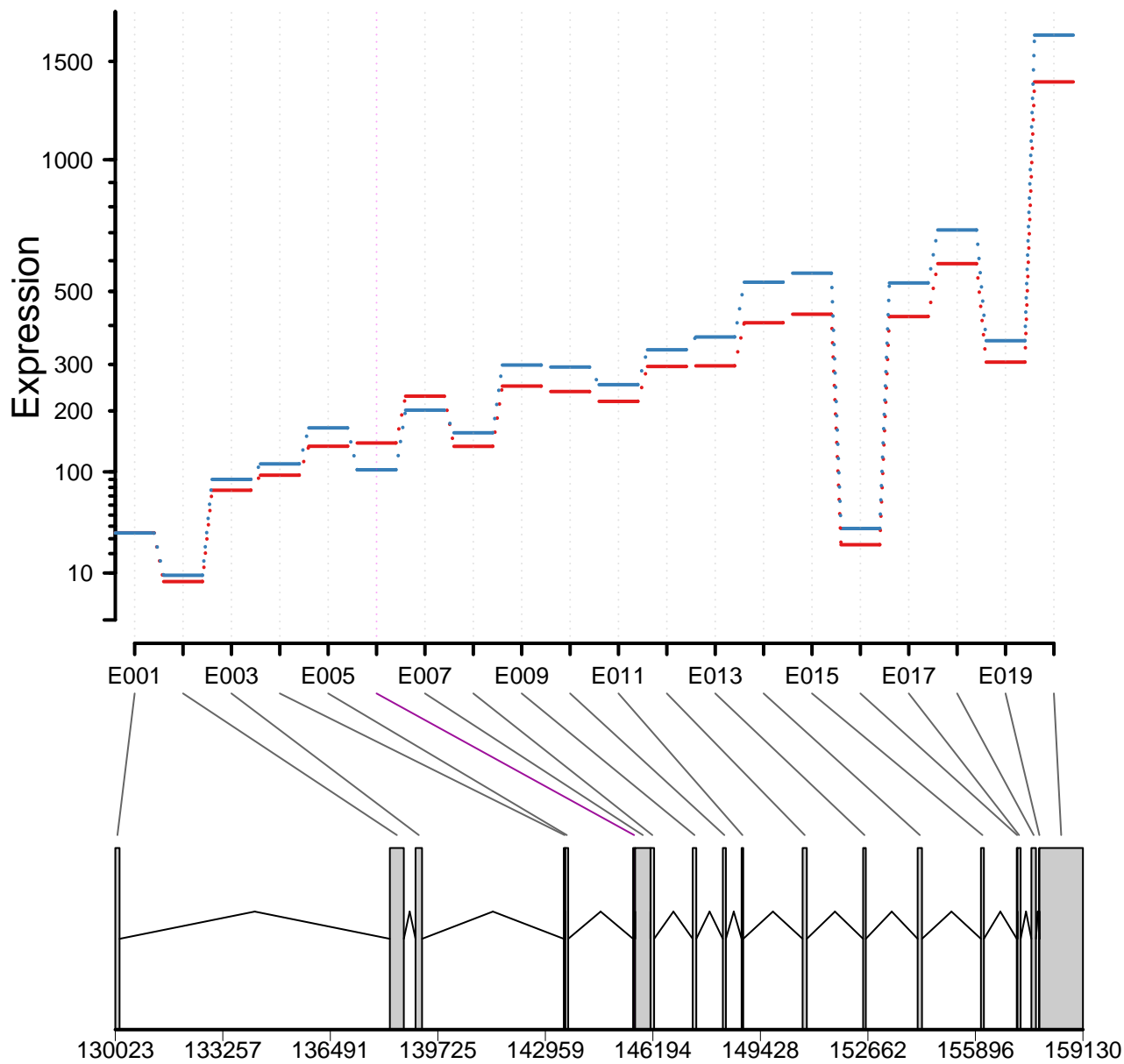

Supplement: Supplementary file 11 [file Data_Sheet_1.ZIP › Supplementary 17/gene-Srsf11.pdf]

gene-Srsf7 -

C

S

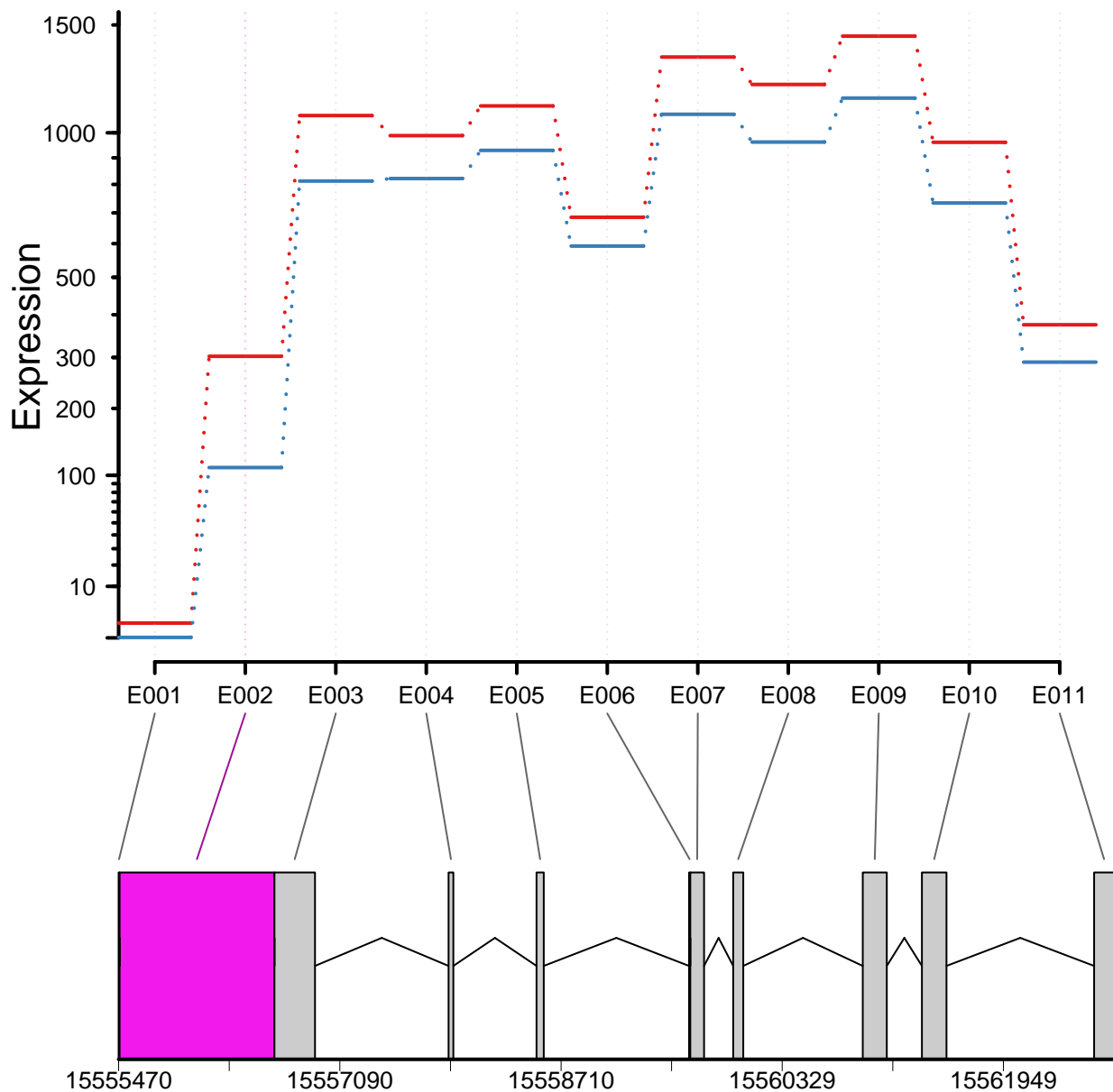

Supplement: Supplementary file 11 [file Data_Sheet_1.ZIP › Supplementary 17/gene-Srsf7.pdf]

gene-Ssr1 +

C

S

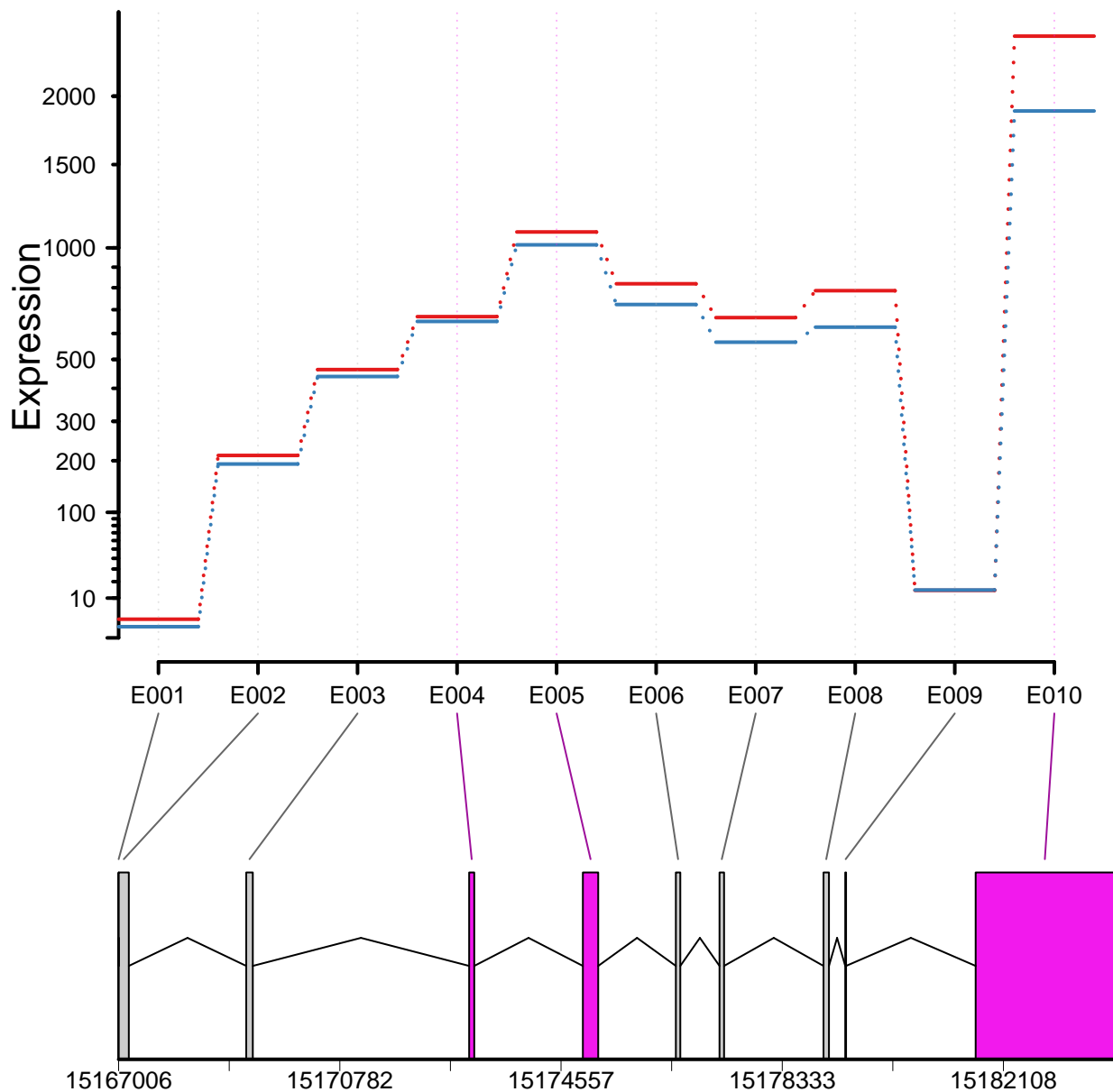

Supplement: Supplementary file 11 [file Data_Sheet_1.ZIP › Supplementary 17/gene-Ssr1.pdf]

gene-Syncrip +

C

S

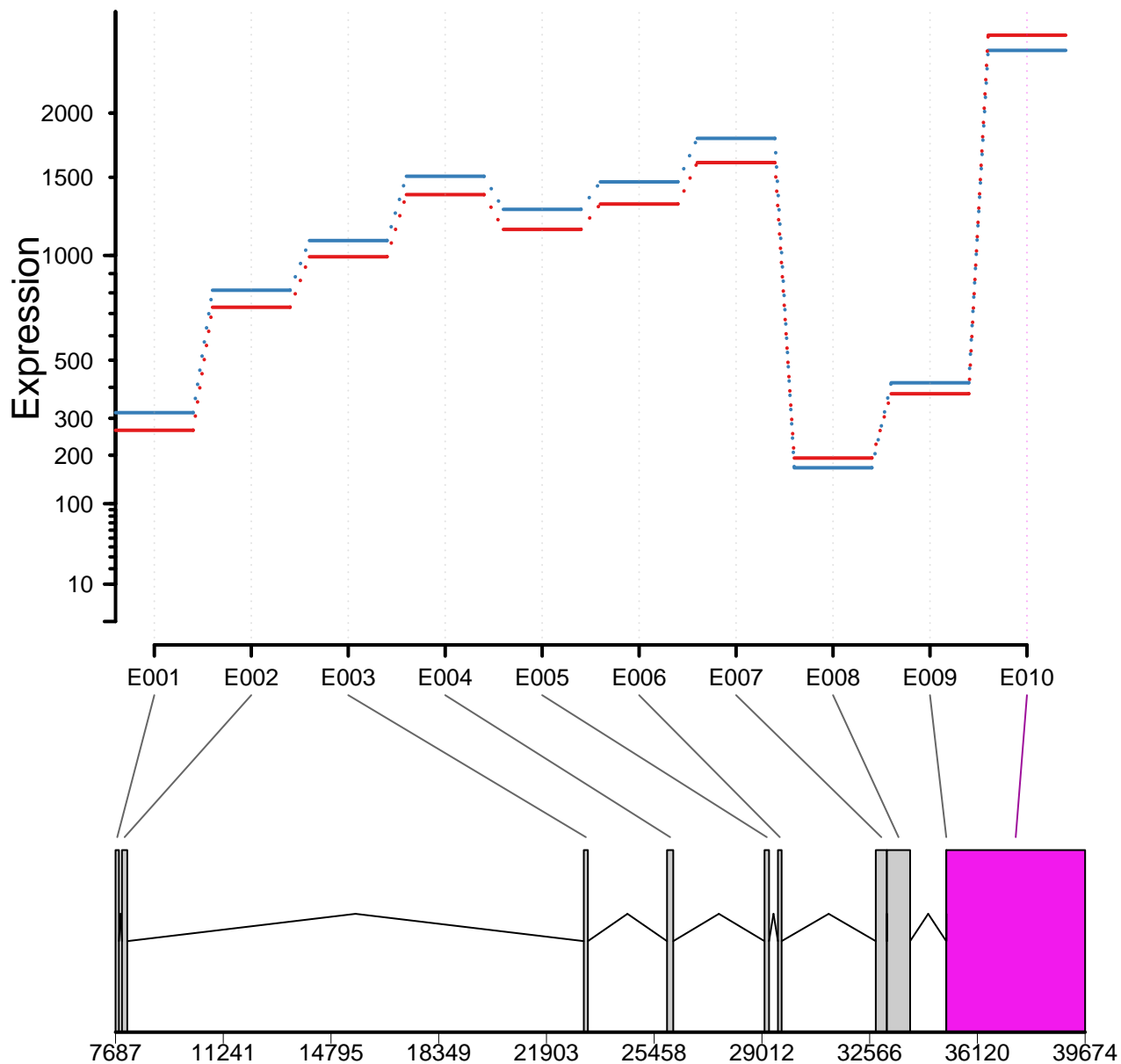

Supplement: Supplementary file 11 [file Data_Sheet_1.ZIP › Supplementary 17/gene-Syncrip.pdf]

gene-SytI2 +

C

S

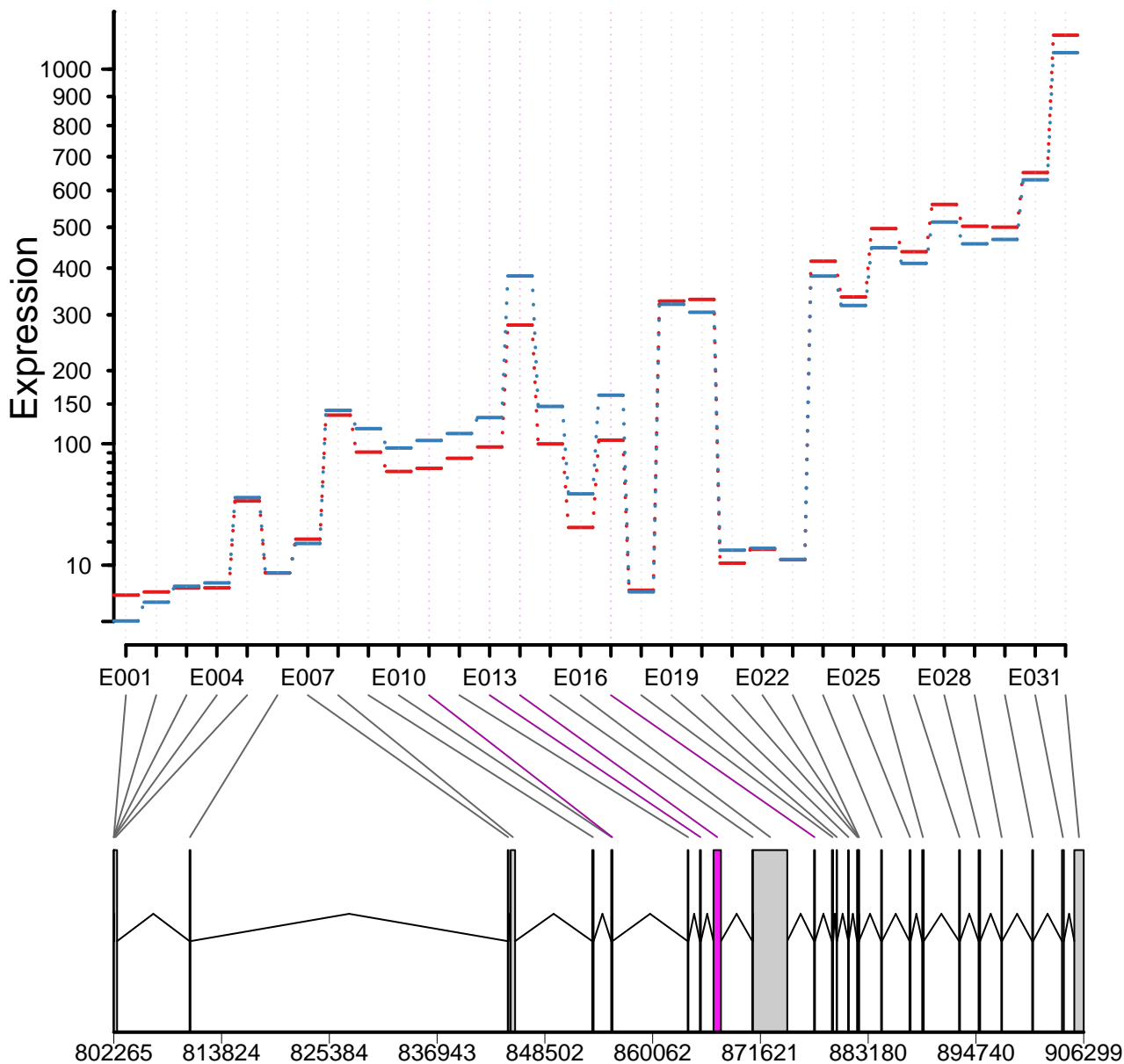

Supplement: Supplementary file 11 [file Data_Sheet_1.ZIP › Supplementary 17/gene-Sytl2.pdf]

gene-Tcf4 -

C

S

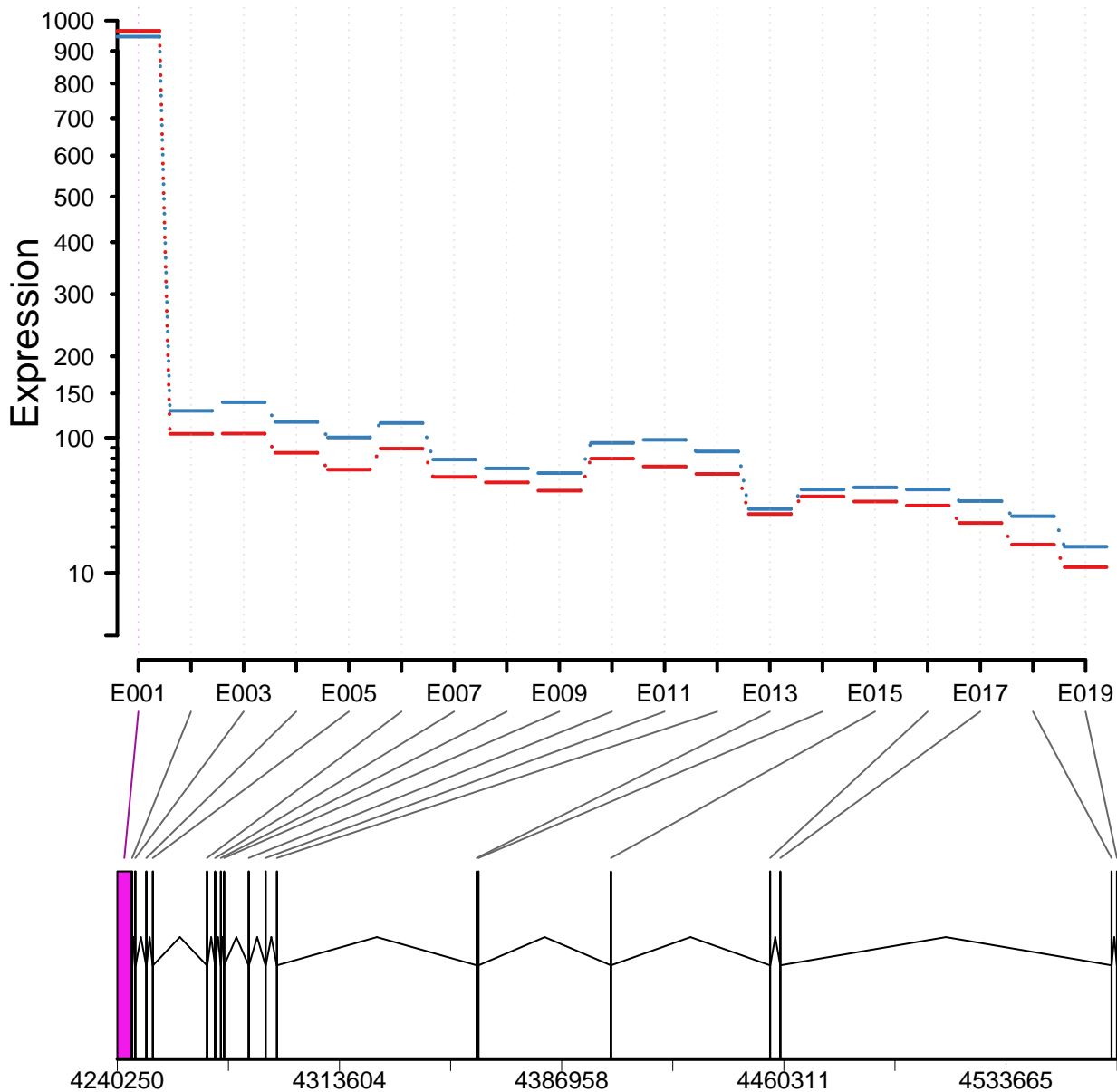

Supplement: Supplementary file 11 [file Data_Sheet_1.ZIP › Supplementary 17/gene-Tcf4.pdf]

gene-Tdrd3 -

C

S

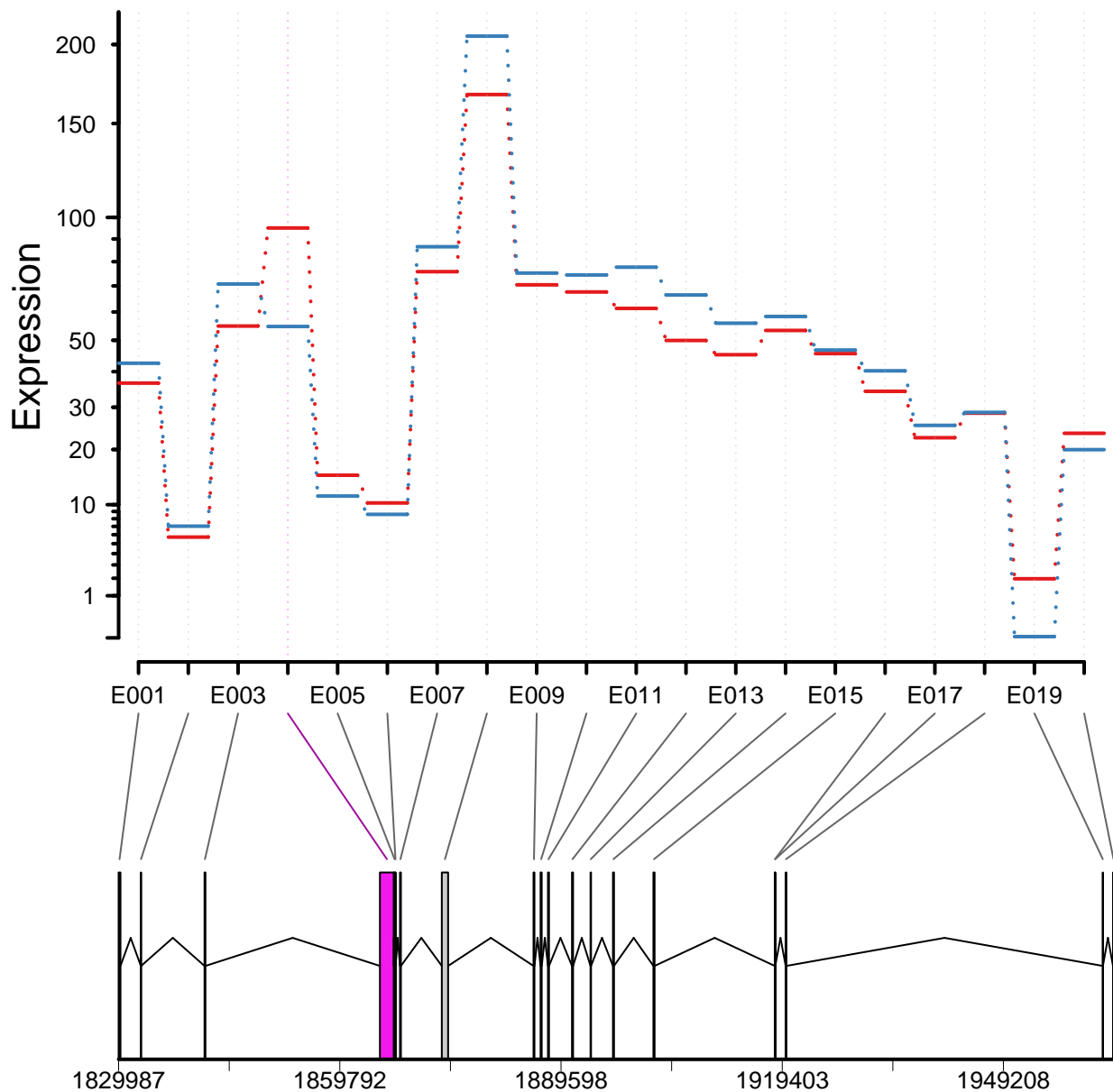

Supplement: Supplementary file 11 [file Data_Sheet_1.ZIP › Supplementary 17/gene-Tdrd3.pdf]

gene-Tmem11 +

C

S

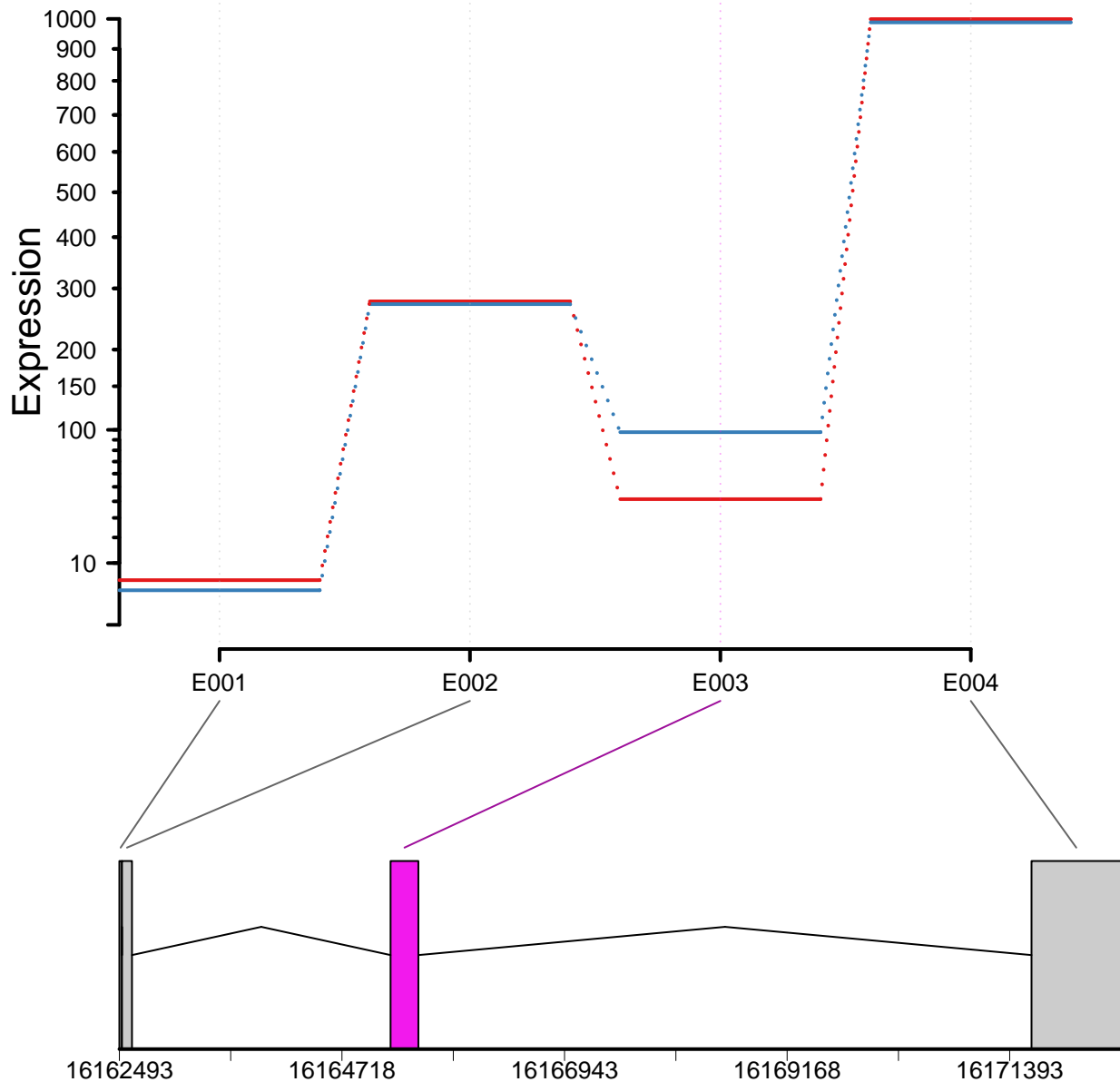

Supplement: Supplementary file 11 [file Data_Sheet_1.ZIP › Supplementary 17/gene-Tmem11.pdf]

gene-Tmem267 +

C

S

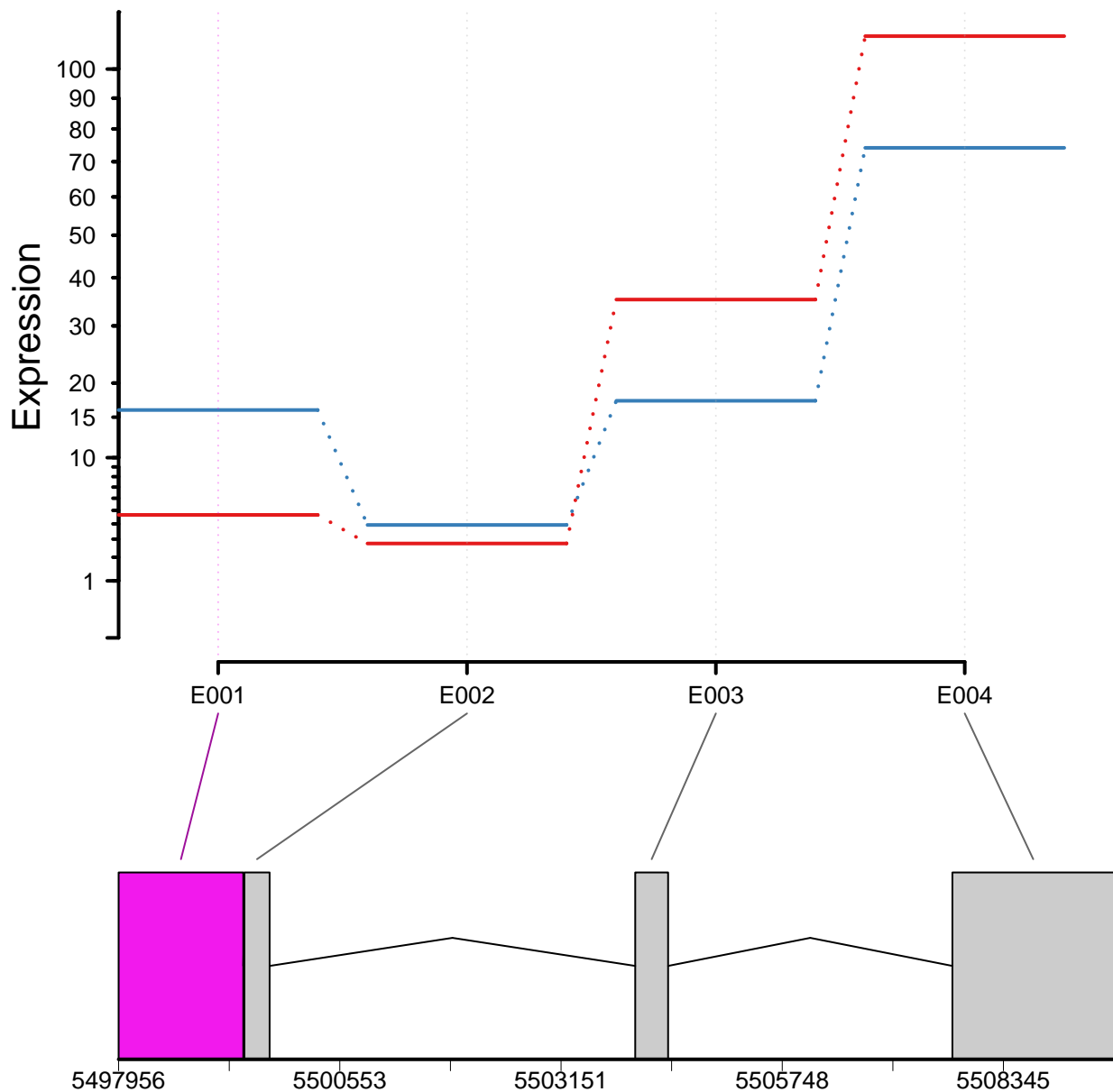

Supplement: Supplementary file 11 [file Data_Sheet_1.ZIP › Supplementary 17/gene-Tmem267.pdf]

gene-Tnrc6a -

C

S

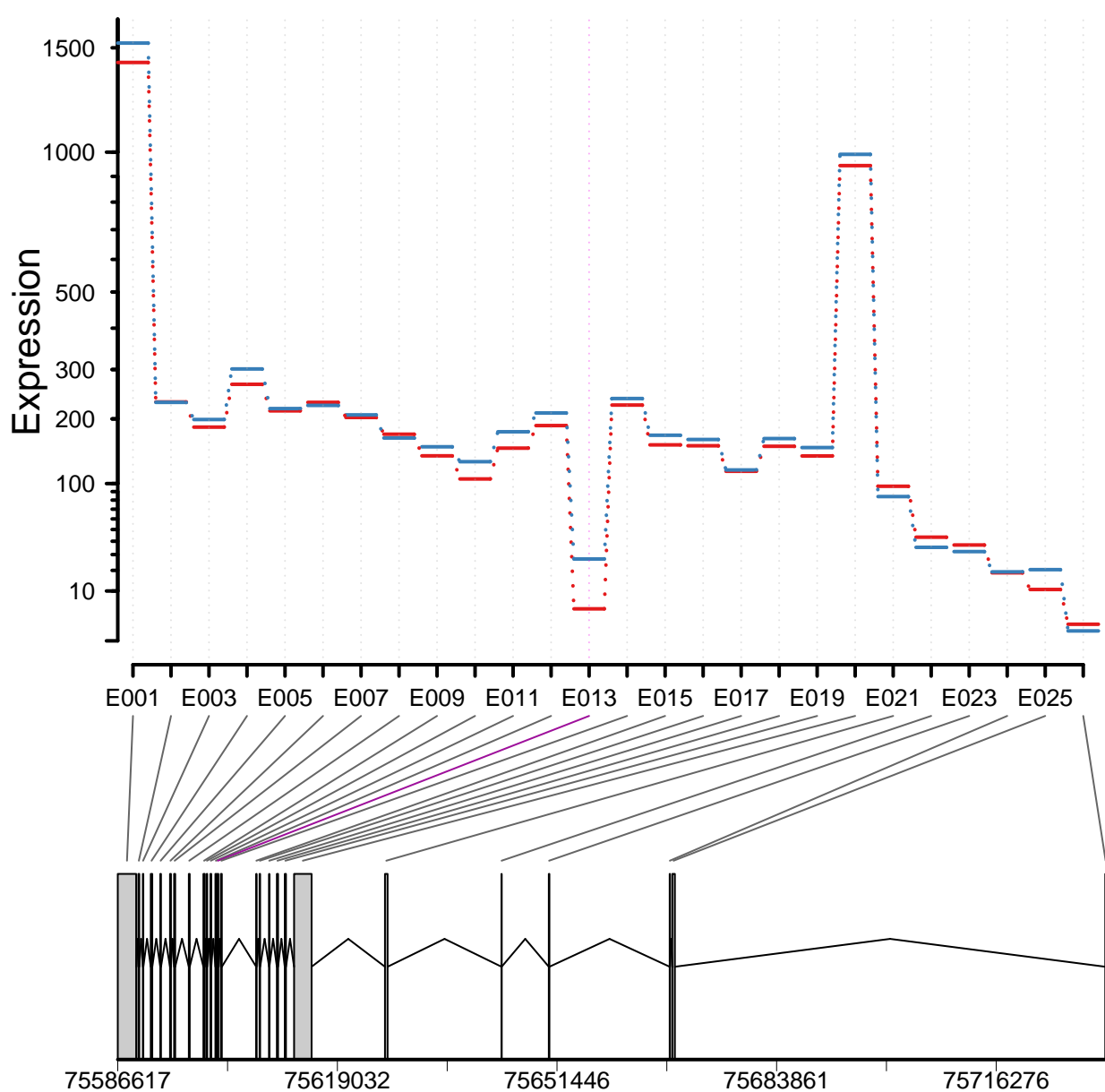

Supplement: Supplementary file 11 [file Data_Sheet_1.ZIP › Supplementary 17/gene-Tnrc6a.pdf]

gene-Tnrc6c -

C

S

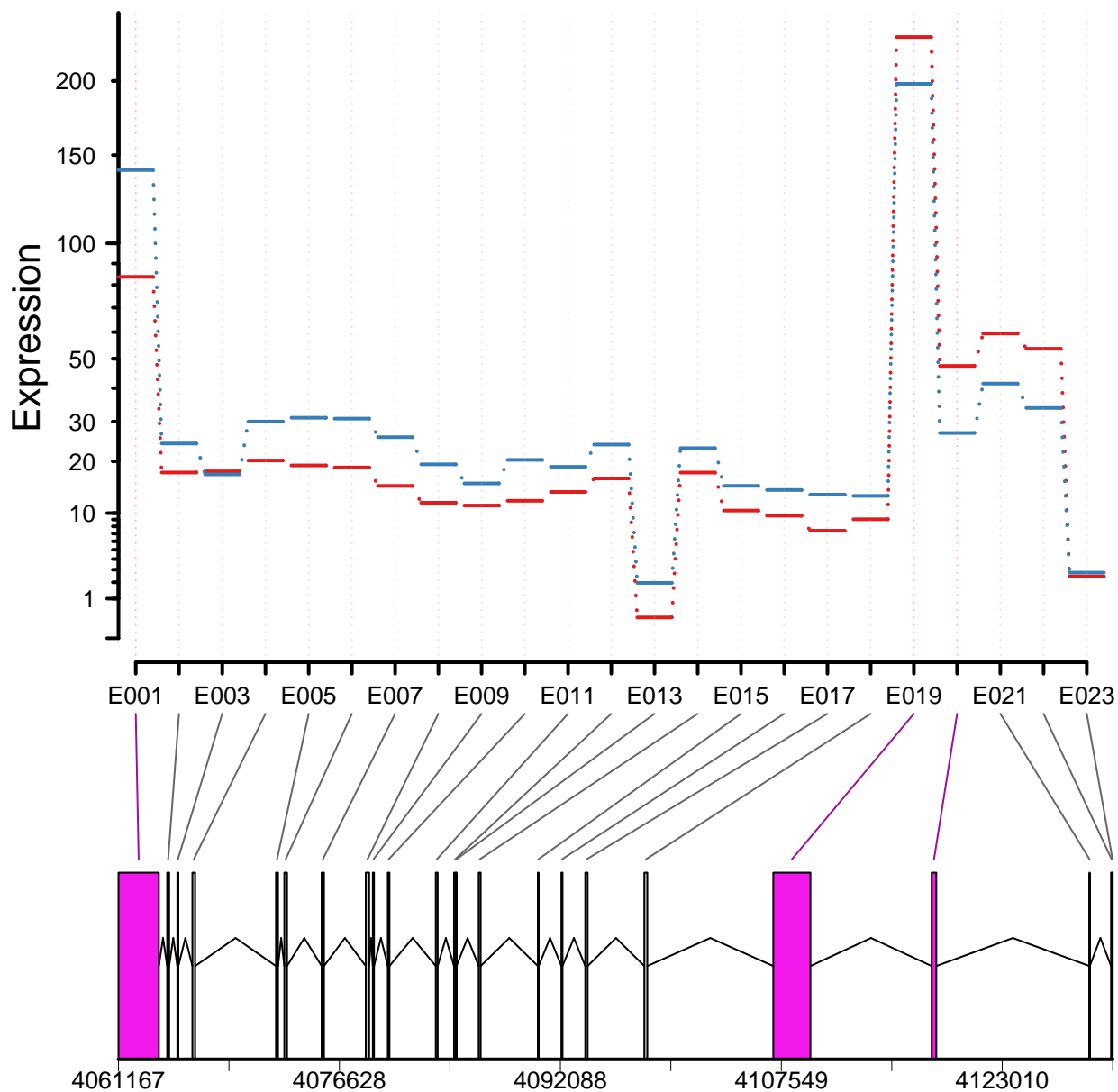

Supplement: Supplementary file 11 [file Data_Sheet_1.ZIP › Supplementary 17/gene-Tnrc6c.pdf]

gene-Tpcn2 +

C

S

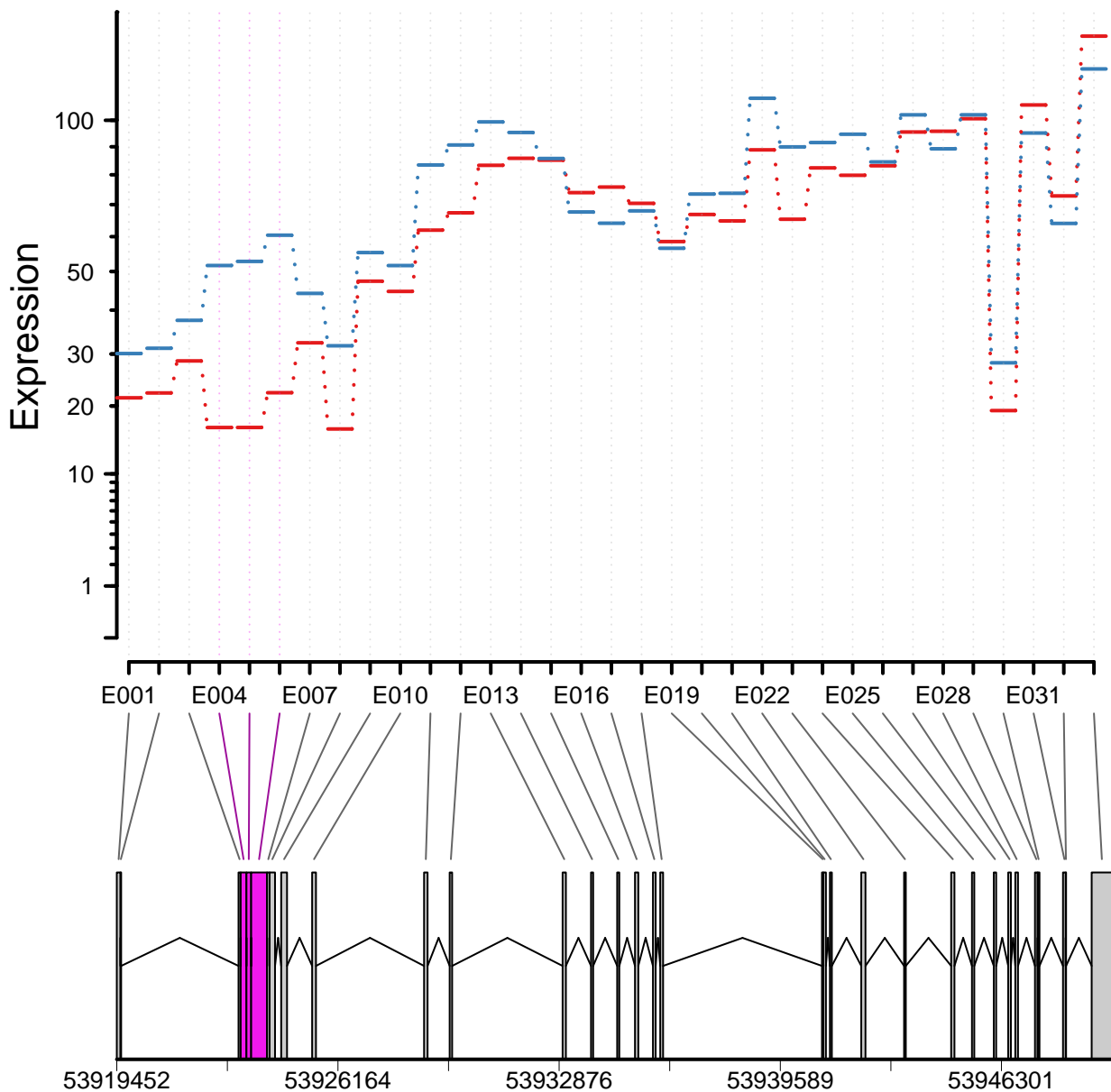

Supplement: Supplementary file 11 [file Data_Sheet_1.ZIP › Supplementary 17/gene-Tpcn2.pdf]

gene-Trim24 +

C

S

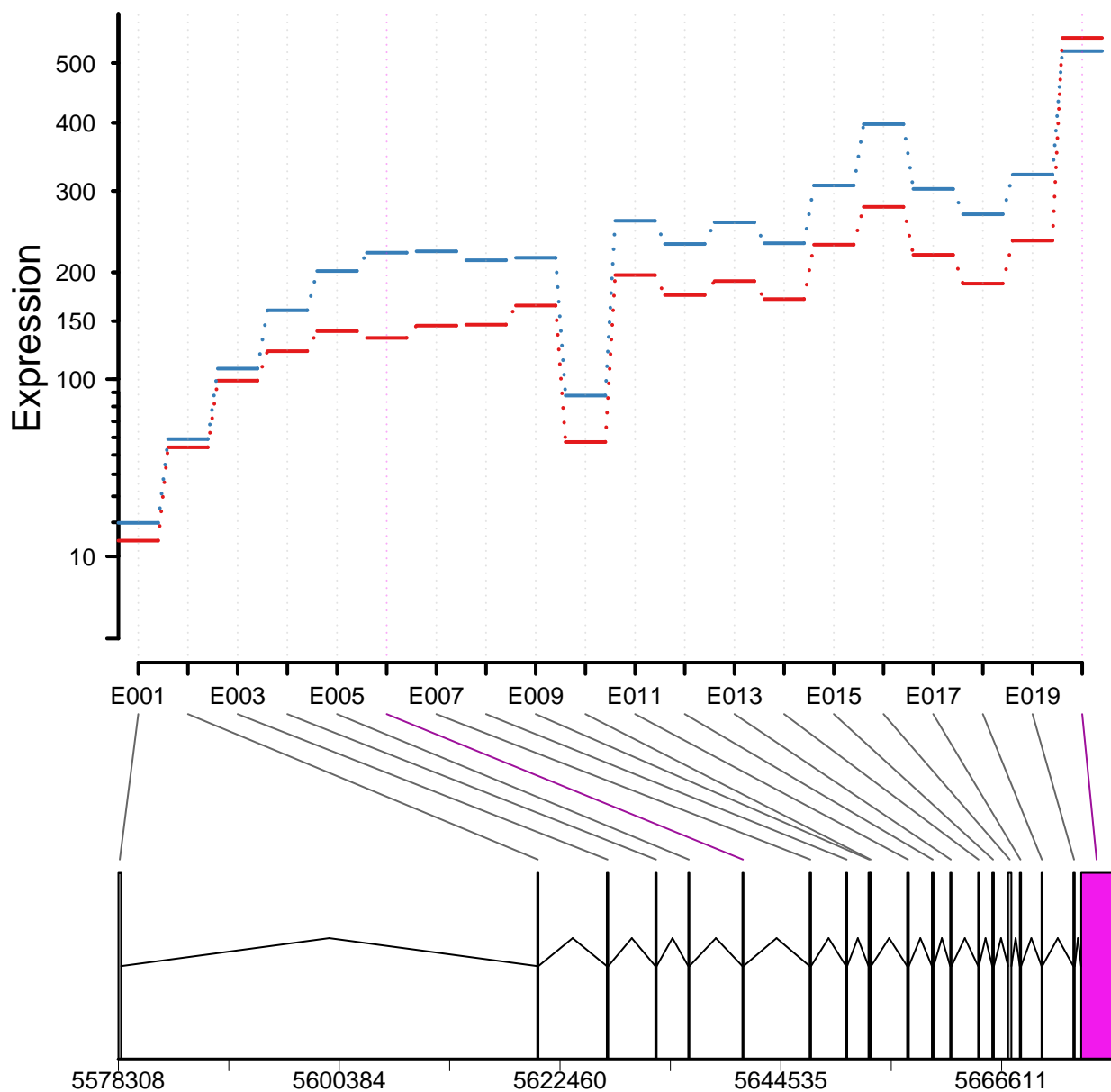

Supplement: Supplementary file 11 [file Data_Sheet_1.ZIP › Supplementary 17/gene-Trim24.pdf]
