# Supplementary figures and images for: Comparative transcriptomics analysis on Senecavirus A-infected and non-infected cells (part 2 of 2)
Source: Front Vet Sci. 2024 Jun 25;11:1431879. doi: 10.3389/fvets.2024.1431879 (PMC11231404; doi:10.3389/fvets.2024.1431879)

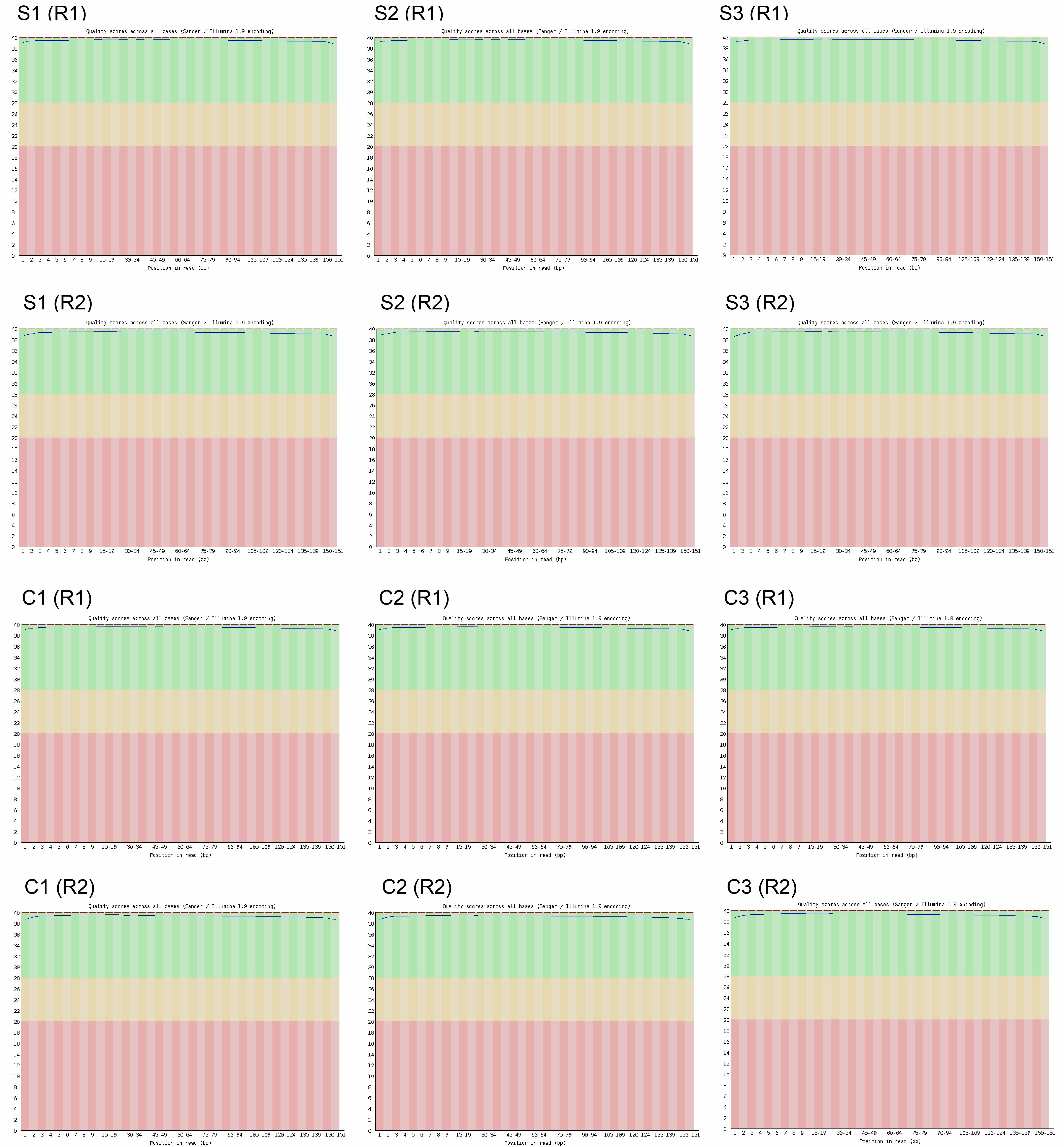

Supplement: Supplementary file 12 [file Image_1.JPEG]

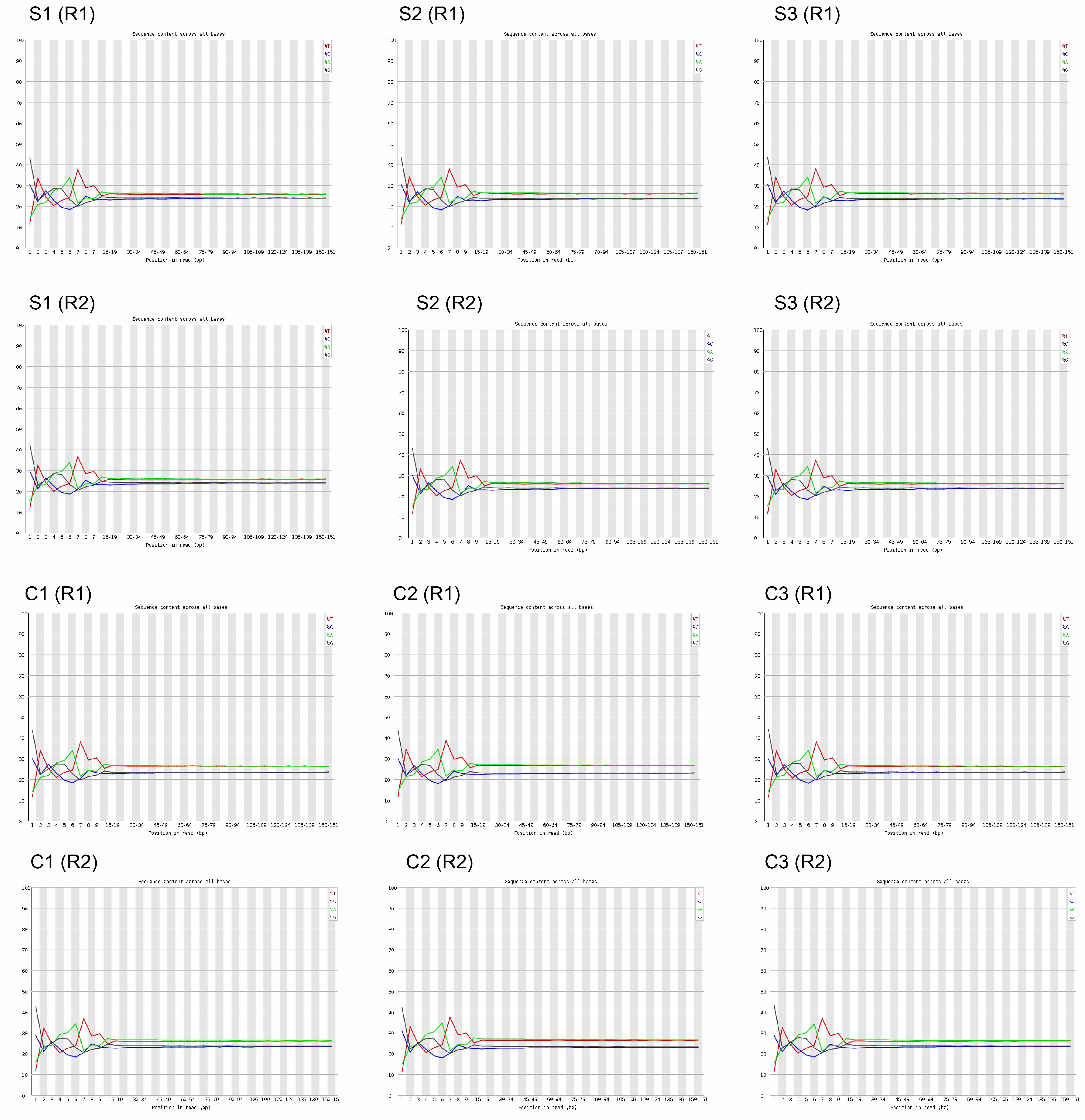

Supplement: Supplementary file 13 [file Image_2.JPEG]

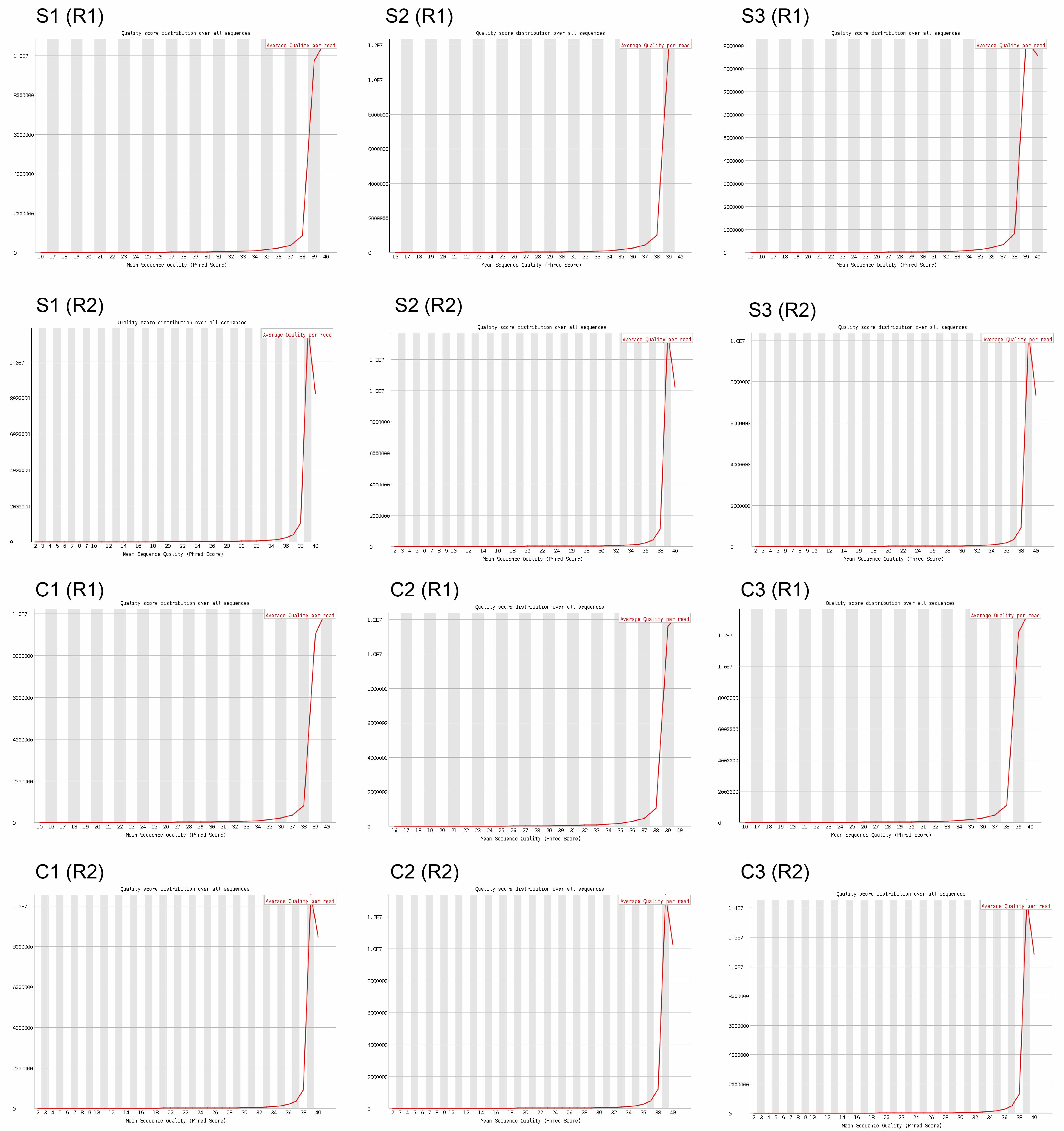

Supplement: Supplementary file 14 [file Image_3.JPEG]

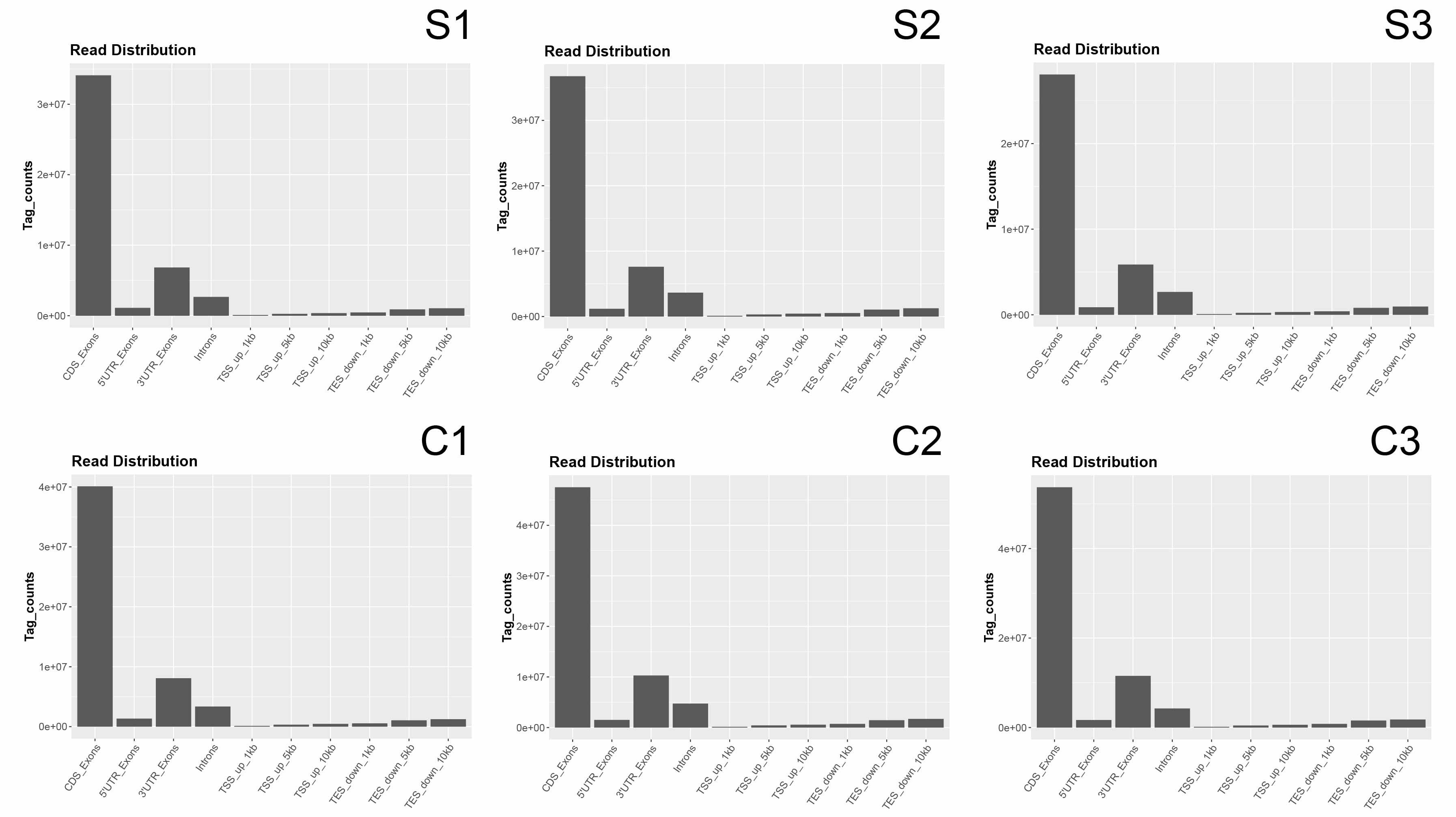

Supplement: Supplementary file 15 [file Image_4.JPEG]

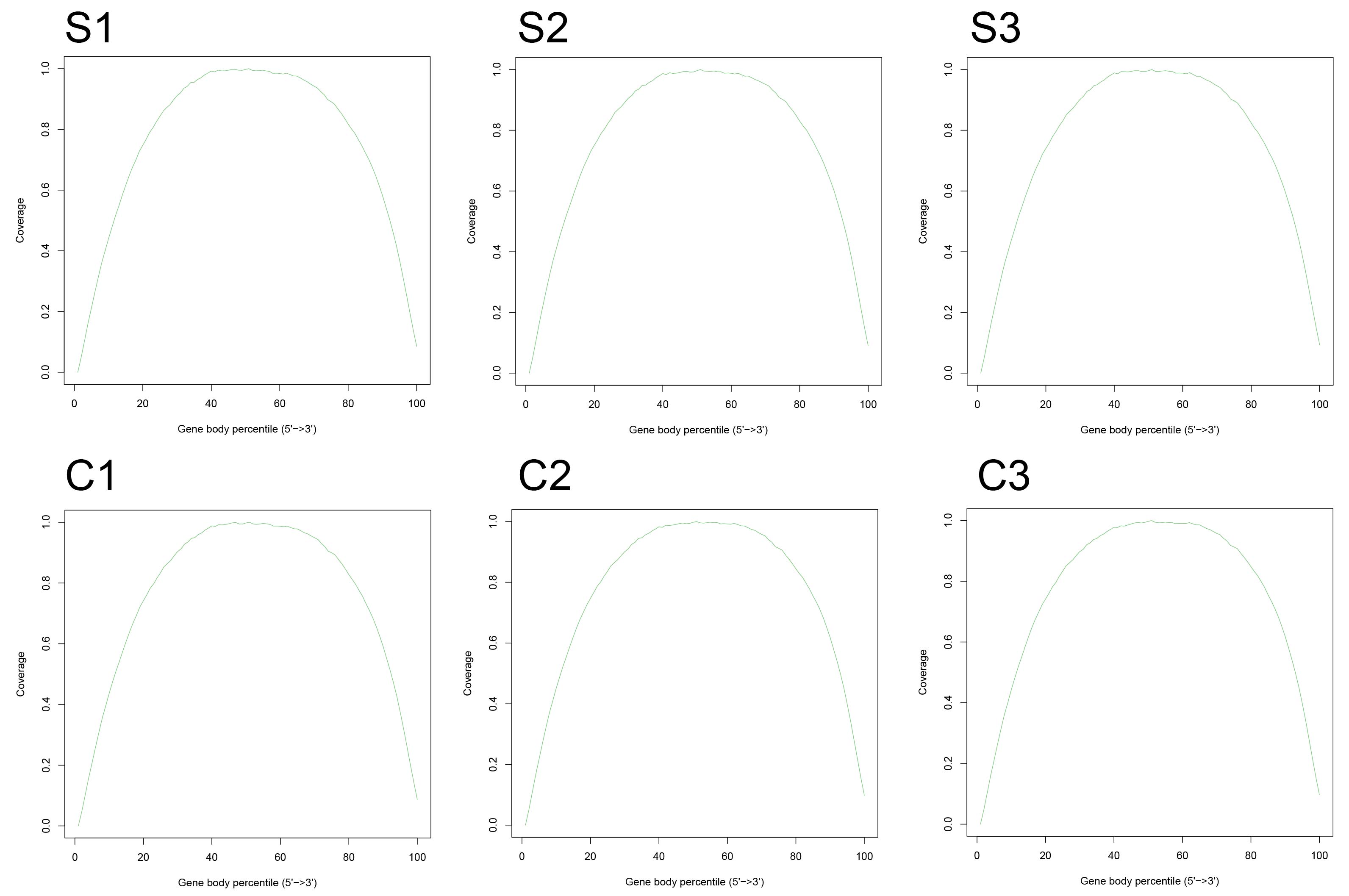

Supplement: Supplementary file 16 [file Image_5.JPEG]

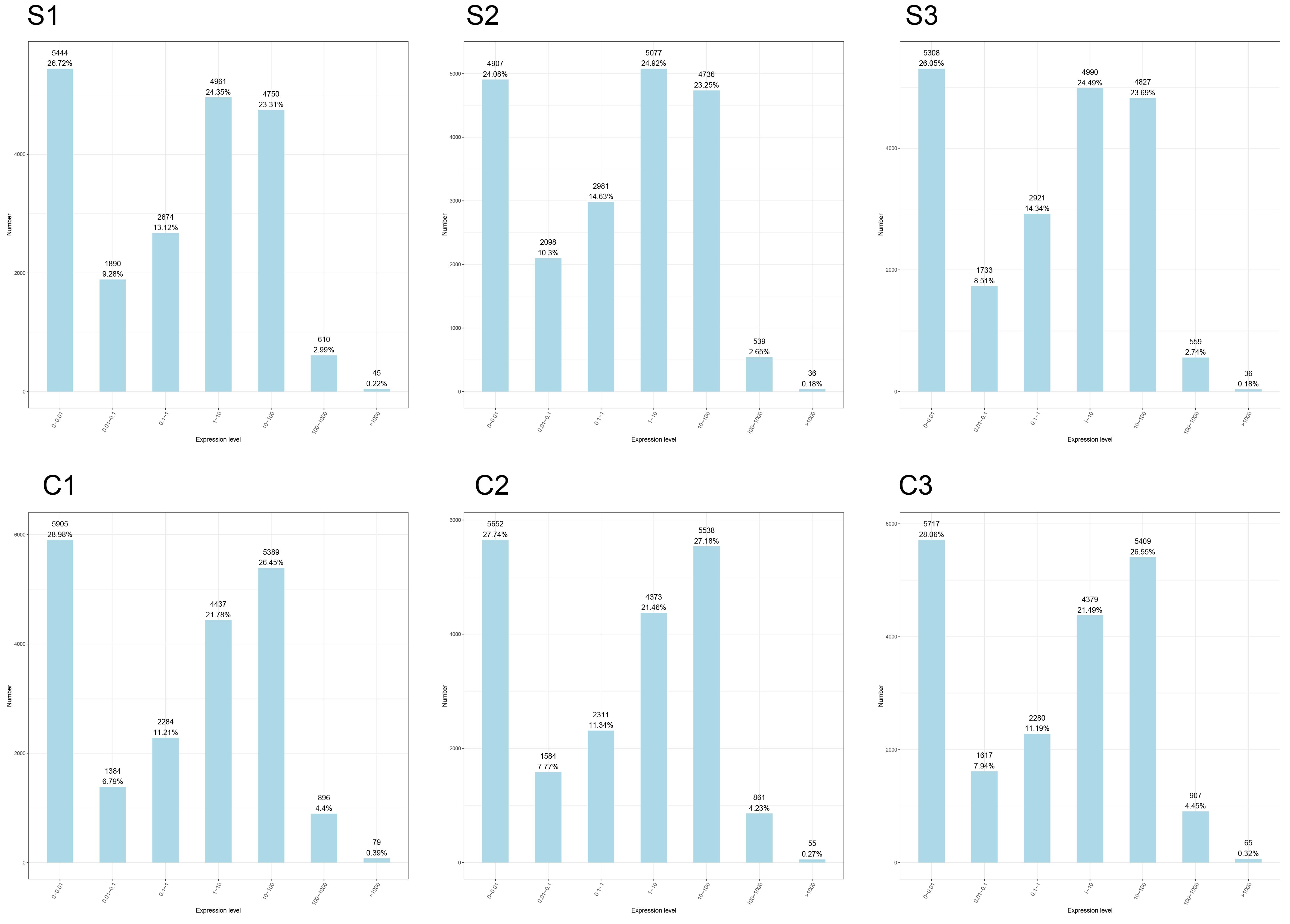

Supplement: Supplementary file 17 [file Image_6.JPEG]

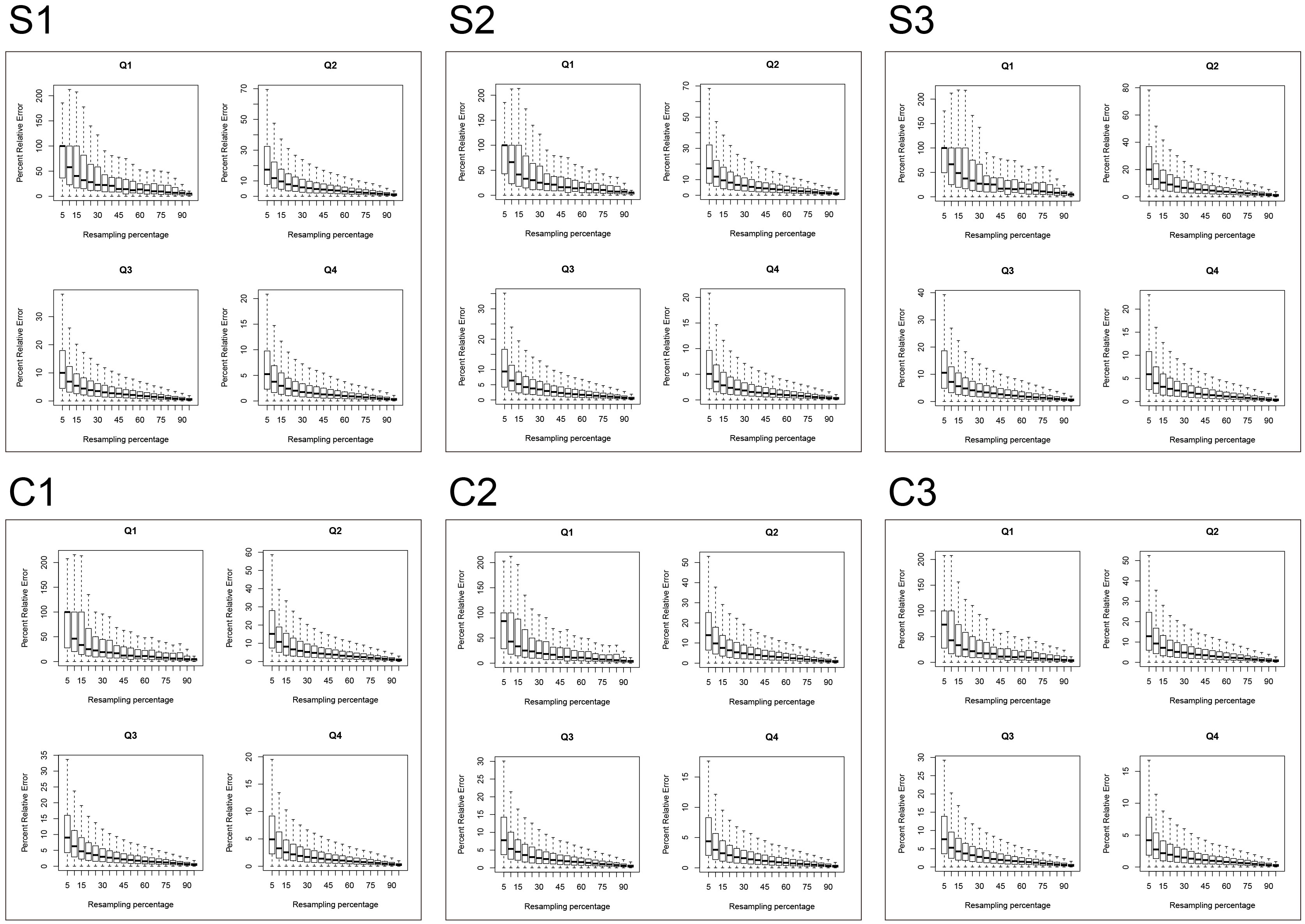

Supplement: Supplementary file 18 [file Image_7.JPEG]
